# Comparison of lower body joint kinematics during change of direction tasks estimated using a markerless and a markerbased method

**DOI:** 10.1038/s41598-025-21143-x

**Published:** 2025-09-30

**Authors:** Janina Helwig, Jos Vanrenterghem, Bastian Anedda, Daniel Koska, Markus Hipper, Yannick Denis, Luca Braun, Veerle Segers, Albert Gollhofer, Steffen Willwacher

**Affiliations:** 1 Institute for Advanced Biomechanics and Motion Studies, Offenburg University, Max-Planck Straße 1, 77652 Offenburg, Germany; 2 Department of Sport and Sport Science, Albert-Ludwigs-University Freiburg, Sandfangweg 4, 79102 Freiburg im Breisgau, Germany; 3 Department of Rehabilitation Sciences, KU Leuven, Tervuursevest 101, Leuven, 3001 Belgium; 4 Institute of Human Movement Science and Health, Technische Universität Chemnitz, Thüringer Weg 11, 09126 Chemnitz, Germany; 5 Department of Movement and Sports Sciences, Ghent University, Watersportlaan 2, Ghent, B9000 Belgium

**Keywords:** Markerless, Marker-based, 3D motion capturing, Physiology, Biophysics

## Abstract

Marker-based (MB) motion capture systems face challenges like marker loss, soft tissue artifacts, and manual processing. Markerless (ML) motion capture systems might be particularly advantageous for capturing dynamic, non-linear movements like change of direction (COD) movements. The agreement between MB and ML systems for lower extremity joint kinematics during COD tasks was evaluated. Nineteen athletes performed cutting movements in 5 directions at 3 intensities. Data was captured using infrared and video cameras. Joint angles were computed, and the agreement was assessed using prediction band and extended Bland-Altman (BA) methods. Knee joint angles showed the smallest random and systematic errors (bias = 4.34°, area = 3102.57 deg·stance%; BA: bias = 3.34°, limits of agreement (LoA) = ± 11.38°) compared to ankle and hip joint angles. Flexion/extension angles displayed a smaller random error (area = 2919.74 deg·stance%; LoA = ± 10.71°) compared to ab-/adduction (area = 3477.70 deg·stance%; LoA ± 12.16°) and internal/external rotation angles (area = 4630.68 deg·stance%; LoA = ± 15.75°). Slower and straight-line movements demonstrated stronger agreement than sharper, non-linear directions. These findings provide valuable insight for researchers and practitioners when placing ML data in the context of existing data, with particular care when considering highly dynamic, non-linear movements or internal/external rotation angles.

**Supplementary Information:**

The online version contains supplementary material available at 10.1038/s41598-025-21143-x.

## Introduction

Motion capturing is a principal technology to perform kinematic analyses in biomechanics for clinical or research purposes. Estimating three-dimensional segment position and orientation and derived kinematic parameters vary depending on the experimental and computational methods used^1,2^. Traditionally, biomechanical analyses are performed using marker-based (MB) motion capture systems. This approach is the current reference standard, and allows to capture a wide range of dynamic movements in a non-invasive manner. Yet, MB motion capturing also presents several limitations, including the need to attach markers to precise anatomical landmarks^3^, operator-dependent variability^3,4^, susceptibility to skin motion artifacts^3,5,6^, potential marker loss, and reliance on manual post-processing. Together, these factors make for a time-intensive procedure. Markerless (ML) systems, based on deep learning algorithms, are an alternative that overcomes some of the limitations of MB systems. Measurements can be performed outside the laboratory with little preparation time. Larger groups of participants can be measured in a short time frame. While both systems have their advantages and disadvantages, the ML approach provides benefits, especially when performing large, and in the field data collections.

However, to contextualize the study results based on ML data within the biomechanical research output of MB systems, it is necessary to thoroughly analyze the difference in results between ML- and MB-based calculations. This study focuses on Theia3D (Theia Markerless Inc., Kingston, Ontario, Canada), one of the most widely used ML systems in research settings at the moment. Several research groups have evaluated, validity, reliability and kinematic parameters of Theia3D^7–12^. Strutzenberger et al.^11^ evaluated lower limb joint angles during a countermovement jump. They found intraclass correlation coefficients from 0.885 to 0.988, with the lowest and highest coefficients observed for the hip and ankle flexion angle, respectively. Lower limb joint angles with a mean root mean square difference (RMSD) between 5–10° during treadmill running were calculated by Kanko et al.^13^ Song et al.^14^ further supported strong agreement between systems for ankle and knee joint angles across eight movements (heel raises, walking, stepping down from a box, countermovement jump, running, regular squat, sumo squat, and a COD movement; Pearson’s correlation coefficient *r* ≥ 0.877).

While the agreement of these two systems has been investigated during several movements mentioned above, little attention has been paid to change-of-direction (COD) movements. The only study that analyzed lower extremity kinematics during a COD task, which lacked further specification, found a higher RMSD but also a higher correlation coefficient (RMSD: 5.1° – 15.9°, *r* = 0.601–0.995) for the COD task when compared to overground running (RMSD: 4.5° – 12.8°, *r* = 0.016–0.995)^14^. In most team sports, however, COD tasks are crucial and may decide over winning or losing a game^15,16^. COD movements performed at higher speeds are desirable in terms of performance, but they are associated with a higher risk of injury^15^. In contrast, sharp-angled COD movements are performed at slower speeds since they require greater deceleration and re-acceleration. This increases the knee joint loading, and with that the injury risk^17,18^. Rapid braking and re-acceleration further induce increased wobbling mass. It is well documented that COD movements are associated with injuries such as ankle sprains and anterior-cruciate ligament ruptures^15,19^, which can lead to financial, emotional and health-related burdens^20^. Therefore, COD movements are frequently used in performance and rehabilitation assessments to identify individual strengths and deficits^21,22^, making them essential in training, rehabilitation, and diagnostic settings. Due to decreased setup and analysis times, ML systems may be preferable to MB systems for collecting COD tasks in large teams. This highlights the importance of analyzing the agreement of joint kinematics between MB and ML systems, specifically Theia3D, for COD tasks. To our knowledge, no study has evaluated this agreement during various COD tasks at different speeds. Therefore, the purpose of the study was to evaluate the agreement between MB and Theia3D lower limb joint angle estimates across a variety of COD movements.

Based on the findings of Kanko et al.^13^ and Song et al.^14^, we hypothesized that flexion/extension and abduction/adduction angles exhibit stronger agreement compared to internal/external rotation angles (or inversion/eversion angles at the ankle). Additionally, we hypothesized that ankle and knee angles demonstrate stronger agreement than hip angles, as suggested by the results of Kanko et al.^23^, Song et al.^14^, and Strutzenberger et al.^11^ Based on the findings of Song et al.^14^ and the increased wobbling mass movement with higher acceleration and decelerations we further hypothesized that the agreement during straight running and cutting movements with a smaller angle will show a stronger agreement than during cutting movements with a sharper angle. Lastly, we hypothesized that slower running speeds will exhibit stronger agreement than faster running speeds, based on the findings of Song et al.^14^

## Methods

### Experimental setup and procedure

**Fig. 1:**
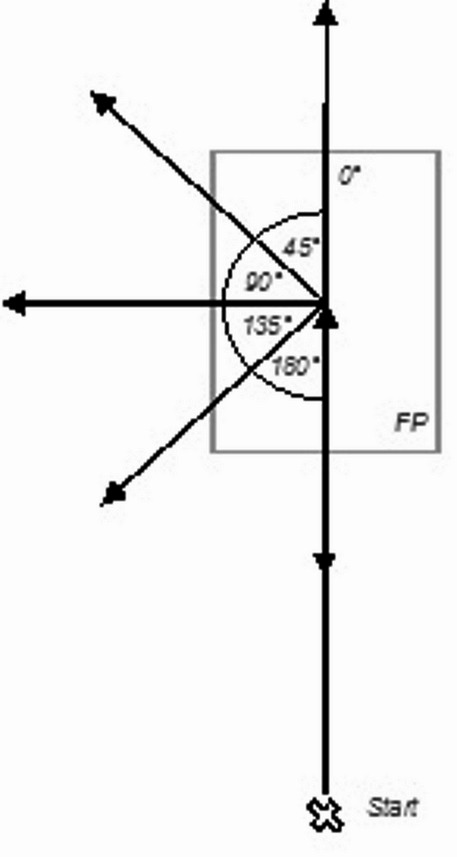
Displayed are the 5 movement directions performed by the participants. FP = force plate.

Two camera systems were installed around the measuring volume, which captured data simultaneously: video cameras for ML motion capturing and infrared cameras for MB motion capturing. Participants performed COD movements in 5 different directions (Fig. 1) at 3 different speeds and joint angles from the ultimate step, during which the direction was changed, were subsequently computed and analyzed.

#### Markerless motion capture

Eight video cameras (Miqus video cameras, Qualisys AB, Gothenburg, Sweden) were used for the ML motion capture. They were calibrated and synchronized using QTM. Data was captured at 85 Hz, which was the highest sampling frequency, allowing to capture with the highest resolution for this camera type (1080 px). The shutter speed was set to 300 µs. The video files were further processed using Theia3D v2023-1-0-3161. The software uses a deep learning algorithm for (anatomical) feature recognition, such as joint locations^9^. An individual subject-specific model is then fitted onto the subject in each video frame^9^. Degrees of freedom were six at the pelvis and three at the hip, knee, and ankle joints.

#### Marker-based motion capture

MB motion capturing was conducted using 21 infrared cameras (Miqus M3 cameras, Qualisys AB, Gothenburg, Sweden) calibrated and synchronized using Qualisys Track Manager (QTM, Qualisys AB, Gothenburg, Sweden). Data were acquired at 200 Hz with a shutter speed of 300 µs and downsampled to 85 Hz to match the Theia3D sampling frequency using a custom-written MATLAB (R2023b, The Mathworks Inc., Natick, USA) script. The data was then further processed in Visual3D (C-Motion Inc., Germantown, MD) using a model that most closely matched the Theia3D model. The model was defined as described in Kanko et al.^13^. Slight modifications to this model were made at the pelvis. Given the focus of the study on lower extremities, due to their critical role and elevated injury risk during COD movements, markers were placed only on the right leg and pelvis, unlike the study above, which employed a full-body marker set. The foot was also modified to allow for different marker placements. The metatarsal 2/3 marker was replaced with a landmark created at the midpoint between 1st and 5th metatarsal head. The modifications were done in order to accommodate the established marker set used in this study^24,25^. Twenty-two retroreflective markers (radius: 7 mm) were attached to the participants’ skin at the pelvis and the right leg at the following locations: right and left anterior and posterior superior iliac spines, right medial and lateral femoral condyles, right medial and lateral malleoli, right medial, lateral and posterior aspect of the heel, first and fifth metatarsal heads, and on top of the tip of the first toe. Four tracking markers per segment were attached to the right lateral thigh and shank. Further details of the marker set can be found in previous publications^24,26^.

### Participants

Nineteen healthy running-based team sport athletes were recruited (6 female/13 male, mean (SD) age: 23.7 (± 4.5) years, height: 178.2 (± 10.0) cm, mass: 74.7 (± 13.0) kg) to participate in the study. They agreed to participate on a voluntary basis and signed an informed consent document. The study was approved by the university’s ethics committee (approval number: 04/23) and all relevant ethical guidelines and regulations were followed. Injury or any impairments to the musculoskeletal system constituted the exclusion criteria. Participants wore minimal, tight clothing and indoor athletic shoes during data collection.

### Data collection

Participants carried out an individual, self-chosen warm-up. Subsequently, markers were attached to their right leg and pelvis. Instructions were then given for the following study procedures. The movements performed included a straight run and four different cutting directions: 45-, 90-, 135-, and a 180-degree cutting angles, as pictured in Fig. 1. The participants ran in each direction at three different intensities with the instructions “slow jogging or around 40% of your maximal speed” for the first intensity, “fast jogging or 60–70% of your maximal speed” for the second intensity, and “sprinting or around 90% of your maximal speed” for the third and last intensity. Each intensity was repeated 3 times, resulting in 45 trials for each participant if all were successful. The starting point was chosen to be 7 m from the force plate (2000 Hz; 0.9 × 0.6 m; AMTI, Watertown, Massachusetts, USA), which was to be contacted with the right leg for stance phase detection. Participants were instructed to “run naturally,” and the laboratory staff adjusted the starting point as needed so that participants could contact the force plate using their naturally chosen stride length. Tape marks on the ground at a 3 m distance from the force plate marked a target for each movement direction. Erroneous trials that did not present the whole foot on the force plate, or where the cutting angle was not achieved based on the failure to cross over the target, were repeated. The run-up speed was not controlled during data collection as the primary objective was to generate a broad range of lower extremity joint angle variations for the analysis. The speed of the center of the pelvis was calculated from marker data during post-processing before touch down. Trials showing marker tracking issues (*n* = 31) during post-processing were excluded from the analysis.

Figure 1*placed near here*.

### Data analysis

ML video data was processed in Theia3D and inverse kinematics calculations were performed in Visual3D. Theia’s built-in GCVSPL filter was set at a cut-off frequency of 20 Hz. MB data was also processed in Visual3D, where a Butterworth low-pass filter with the same cut-off frequency was applied to MB and force data. Further processing was done in MATLAB and RStudio (version 2023.12.1 build 402, RStudio Team). The extracted joint angles from hip, knee and ankle were time normalized to the stance phase (> 20 N) to 101 data points.

In the first step of the analysis, the agreement between ML and MB kinematics across hip, knee and ankle was evaluated, as well as across the three movement planes. In a second step, the agreement between joint kinematics across different running directions was analyzed. Finally, in step three, the agreement across different running intensities was compared. For this, the trials were categorized into slow, medium and fast. This was based on tertiles of the calculated approach speed. After initial analysis, we observed that this approach resulted in an unequal distribution of intensity categories across running directions, meaning no fast trials for 180-degree COD movements and no slow trials for 45-degree and straight running movements. For a more balanced analysis, direction-specific speed tertiles were subsequently defined.

In this context, agreement refers to the systematic and random differences between measurement systems. Two methods assessed the agreement between ML and MB joint kinematics. First, an extended Bland-Altman (BA) analysis^27^ was conducted to examine agreement in right lower limb joint kinematics at touchdown (TD) and toe-off (TO), as well as the peak minimal and peak maximal joint angles during the stance phase. To account for repeated measures per subject, the BA analysis was extended using a linear mixed-effects models (LMMs). The model uses a random intercept for the participants and fixed effects for the MB estimate. The regression line from this model represents the bias line. Limits of agreement (LoAs) were calculated as ± 1.96 times the adjusted standard deviation (SD), as described in Bland and Altman (1999)^27^ in their Eq. 5.3 for repeated measures. The confidence intervals were computed based on Eq. 5.10 from the same research article by Bland and Altman (1999)^27^. All bias estimates are reported at the mean MB estimate. The normality of model residuals was assessed using the Shapiro-Wilk test^28^ (violation: *p* < 0.05). Linearity was assessed using the Spearman rank correlation coefficient between fitted values and model residuals (*p* < 0.05 and absolute correlation coefficient > 0.3). Homoscedasticity was assessed through regression of the absolute residual values against fitted values (*p* < 0.05 and absolute slope > 0.1). The bias, as an indicator of the systematic difference, the limits of agreement (LoA), as an indicator of the random difference, and their respective confidence intervals, were extracted.

**Fig. 2:**
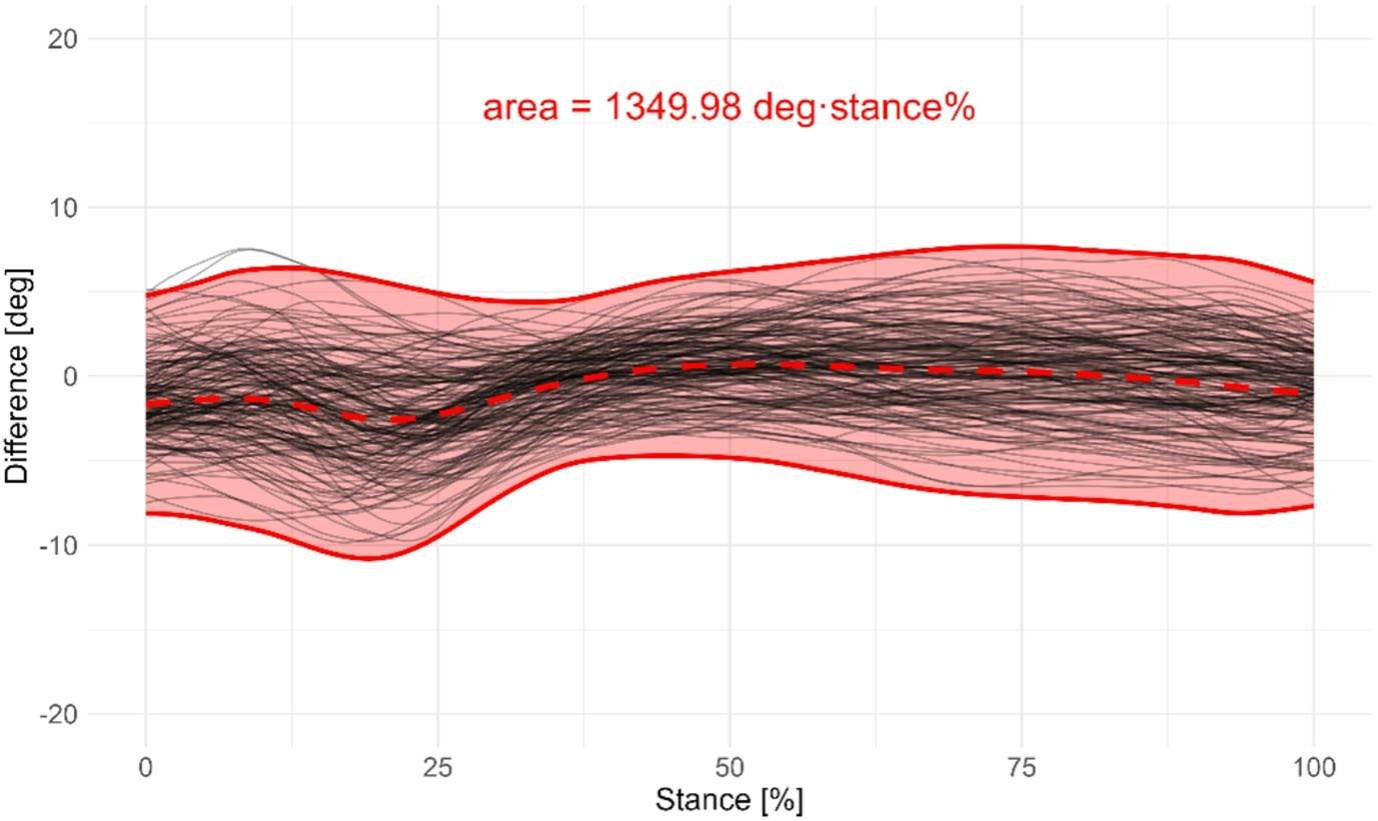
A 95% prediction band region (outer think, red lines) for the difference in knee flexion/extension angle (ML° - MB°) during stance phase of the 45-degree cutting movement. The shaded area in between the bands is the area calculated during analysis as a measure of the random error. The middle thick, dotted line represents the mean difference (bias) between systems.

Second, bootstrapped functional prediction bands for continuous differences were computed in RStudio using the FunBootBand function developed by Koska^29^. The area between the upper and lower bands over the stance phase is expressed in deg·stance% and represents the magnitude of the random difference between the systems, with larger areas indicating larger variability. Just like in the extended BA analysis, the bias represents the systematic difference. Positive values indicate that ML measurements yield larger joint angles compared to MB estimates, and negative values indicate the opposite. A narrow prediction band, or a small area between the bands, and a small bias suggest strong agreement between the systems, whereas wider bands, or a large area indicate larger divergence (Fig. 2). The functional prediction bands for the differences between systems for each variable were estimated using 400 bootstrap iterations per band, as described in the study by Lenhoff et al.^30^. The significance level α was set to 0.05. Trials were assumed as dependent. Subsequently, the maximum and minimum width, the area between the upper and lower prediction bands, and the mean bias for each joint, plane, cutting direction, and running intensity, were extracted.

Figure 2*placed near here*.

## Results

Of the 756 fitted models, 46% (348/756) met all statistical assumptions. Normality violations were observed in 53% (401/756), linearity violations in 0%, and heteroscedasticity in 5% (38/756). Individual BA plots with LMM analysis are depicted in the Supplementary Fig. 1-756.

### Agreement across joints and movement planes

The mean bias indicated that ML joint angles were estimated to be larger than the MB counterparts. Only the ML knee ab-/adduction and internal/external rotation were estimated to be smaller using the prediction band method and the ML knee joint angles in all movement planes when using the extended BA analysis. The results of the prediction band and extended BA analysis are summarized in Table 1. The regression analysis of the LMM revealed a mean slope of -0.32 indicating a 0.32° change in difference between systems for every 1° increase in MB joint angle, which was similar across joints (-0.31 – -0.35) but differed between planes, with the flexion/extension plane showing the smallest slope of -0.09, followed the ab-/adduction (-0.30), and rotation (-0.58). On average, the agreement between the ML and MB estimates was strongest for the knee joint kinematics: it exhibited the smallest random error (area = 3102.57 deg·stance%, LoA = ± 11.38°), compared to the hip (area: 3562.70 deg·stance%; LoA: ±13.05°) and the ankle (area: 4362.85 deg·stance%; LoA: ±14.19°) joints. The absolute biases were also smallest for the knee joint estimates, on average: 4.34°, and 3.34°, for the prediction band method and the extended BA analysis, respectively, compared to 6.13°, and 5.22° for the hip and 4.23°, and 5.27° for the ankle. On average, flexion/extension angles had an area of 2919.74 deg·stance% and LoA of ± 10.71° compared to 3477.70 deg·stance% and LoA of ± 12.16° for ab-/adduction angles and 4630.68 deg·stance% and LoA of ± 15.75° for rotation angles. For all three joints, the largest random and systematic errors were observed in the transverse plane, which corresponded to joint rotation for the knee and hip joints, and abduction/adduction movement for the ankle joint. Detailed results for each movement plane and each joint can be found in Supplementary Table 1 for the prediction band method and in Supplementary Table 2 for the extended BA analysis.

**Table 1:** Smallest and largest values and the corresponding joints for the random errors are displayed, as well as the mean values for the random and the systematic errors.

Ab-/Adduction	Prediction Band Analysis	Area (deg·stance%)	Smallest	2244.44	Hip
			to	3123.92	Knee
			Largest	5064.74	Ankle
			Mean	3477.70	
		Absolute Bias (°)	Mean	3.35	
	**Extended BA Analysis**	LoA (°), random error	Smallest	± 8.82	Hip
			to	± 8.96	Knee
			Largest	± 18.69	Ankle
			Mean	± 12.16	
		Absolute Bias (°)	Mean	3.00	
		Slope	Mean	-0.30	
**Flexion/Extension**	**Prediction Band Analysis**	Area (deg·stance%)	Smallest	1797.03	Knee
			to	3338.94	Hip
			Largest	3623.24	Ankle
			Mean	2919.74	
		Absolute Bias (°)	Mean	4.65	
	**Extended BA Analysis**	LoA (°), random error	Smallest	± 7.16	Knee
			to	11.13	Ankle
			Largest	± 13.85	Hip
			Mean	± 10.71	
		Absolute Bias (°)	Mean	4.63	
		Slope	Mean	-0.09	
**Rotation**	**Prediction Band Analysis**	Area (deg·stance%)	Smallest	4386.76	Knee
			to	4400.58	Ankle
			Largest	5104.7 1	Hip
			Mean	4630.68	
		Absolute Bias (°)	Mean	6.82	
	**Extended BA Analysis**	LoA, random error	Smallest	± 12.74	Ankle
			to	± 16.49	Hip
			Largest	± 18.02	Knee
			Mean	± 15.75	
		Absolute Bias (°)	Mean	6.20	
		Slope	Mean	-0.58	

Abbreviations: BA = Bland Altman, LoA = Limits of agreement.

Table 1*placed near here*.

### Agreement across running directions

The agreement between ML and MB estimates varied systematically with cutting direction with similar patterns across both analytical methods. Both methods showed an increase in random error with increasing cutting angle from straight to 180-degree cutting movement, which is displayed in Table 2. For the bias, both methods revealed that the 45-degree cutting movement exhibiting the smallest deviation between systems (4.48° for the prediction band and 4.34° for the extended BA method). The slopes of the bias of the extended BA analysis were similar for the five running directions (-0.41 - -0.48). On average, both methods identified ML estimates to be larger than MB estimates for all directions. The results of each movement plane and each joint separated by each running direction can be found in Supplementary Table 3 for the prediction band method and in Supplementary Table 4 for the extended BA analysis.

**Table 2:** The random and systematic errors sorted from smallest to largest values averaged across all three joints and all planes and the corresponding running directions are displayed.

Prediction Band Analysis	Area (deg·stance%)	Smallest to Largest	2530.8	Straight
			2773.7	45°
			3271.1	90°
			3720.7	135
			3762.9	180°
	Absolute Bias (°)	Smallest to Largest	4.48	45°
			5.05	90°
			6.04	Straight
			6.26	135°
			7.10	180°
**Extended BA Analysis**	LoA (°), random error	Smallest to Largest	± 9.51	Straight
			± 10.29	45°
			± 11.86	90°
			± 12.46	135°
			± 12.87	180°
	Absolute Bias (°)	Smallest to Largest	4.30	45°
			4.46	90°
			4.65	Straight
			5.47	135°
			6.17	180°
	Slope	Mean	-0.44	

Abbreviations: BA = Bland Altman, LoA = Limits of agreement.

Table 2*placed near here*.

### Agreement across running intensities

The approach speeds for the intensity categories for each direction is displayed in Table 3. Table 4 summarizes the agreement across running intensities, showing that, when averaged across directions, slow or medium movements resulted in the largest random and systematic error. Analyzing each running direction separately, indicates that the agreement depends on the direction: For straight running, both methods showed that slow movements had the smallest random error (area = 2279.58 deg·stance%, LoA = ± 8.67°) with larger random errors for medium (area = 2627.06 deg·stance%, LoA = ± 9.15°) and fast (area = 2454.66 deg·stance%, LoA = ± 10.40°) speeds. For the 45-degree cutting movement, the extended BA analysis revealed the same increasing pattern, though the prediction band method showed the opposite pattern with fast movements exhibiting the smallest random (2619.21 deg·stance%) and systematic error (4.20°). For 90-degree movements both methods revealed the medium intensity to have the smallest random and systematic error and for the remaining two movement directions the smallest error occurred for the fast movements for 135-degree cutting and fast or medium for the 180-degree cutting, depending on the method used.

**Table 3:** Displayed are the approach speed thresholds of each category for each direction.

Direction	Slow (m/s)	Medium (m/s)	Fast (m/s)
**Straight**	< 4.50	4.50–5.55	> 5.55
**45-degree**	< 4.36	4.36–5.04	> 5.04
**90-degree**	< 3.14	3.14–3.45	> 3.45
**135-degree**	< 2.47	2.47–2.90	> 2.90
**180-degree**	< 2.36	2.36–2.69	> 2.69

**Table 4:** A summary of the results of the level 3 analysis, displaying the random and systematic errors sorted from smallest to largest values averaged across all three joints and all planes and the corresponding running intensities are displayed.

Prediction Band Analysis	Area (deg·stance%)	Smallest to Largest	3120.91	Fast
			3186.73	Slow
			3189.05	Medium
	Absolute Bias (°)	Smallest to Largest	5.68	Fast
			5.76	Medium
			6.04	Slow
**Extended BA Analysis**	LoA (°), random error	Smallest to Largest	± 11.00	Medium
			± 11.14	Fast
			± 11.72	Slow
	Absolute Bias (°)	Smallest to Largest	4.93	Fast
			4.94	Medium
			5.21	Sow
	Slope	Mean	-0.46	

Abbreviations: BA = Bland Altman, LoA = Limits of agreement.

On average, ML estimates were larger than MB estimates for each running intensity using both methods. The regression analysis of the LMM revealed similar mean slopes for each running intensity: -0.41, -0.46, -0.41, for slow, medium and fast intensities, respectively.

The results of each movement plane and each joint separated by each running direction and further separated by each speed can be found in Supplementary Table 5 for the prediction band method and in Supplementary Table 6 for the extended BA analysis.

Table 3*placed near here.*

## Discussion

The current study’s biomechanical analysis reveals insights into the agreement of ML with MB lower extremity joint kinematics during COD tasks, evaluating the separate effects of cutting angle and running speed. Overall, the study demonstrated that the agreement between MB and ML systems for lower extremity joint kinematics was strongest for flexion/extension angles, especially at the knee while internal/external rotations seem most challenging. The agreement between systems tended to be poorer with larger cutting angles.

### Agreement across joints and movement planes

We hypothesized that flexion/extension and abduction/adduction angles would demonstrate a stronger agreement between measurement systems compared to the internal/external rotation angles (or inversion/eversion angles at the ankle). The results of our study support this hypothesis. Both methodologies identified the internal/external rotation and inversion/eversion angles to exhibit the largest bias and the widest random spread when averaged across all events and all joints. The strongest agreement was observed for the knee flexion/extension angle across all joint angles in the sagittal plane (extended BA analysis bias = 0.56°, LoA = ± 7.16°; prediction band method: bias = 1.17°, area = 1797.03 deg·stance%). These findings are consistent with the findings of Kanko et al.^23^, reporting the largest degree of similarity between systems for the knee angles in the sagittal plane with an average RMSD of 3.3°. Additionally, the findings align with those of Song et al.^14^, who identified weak correlations for the hip rotation angles between ML and MB estimates for all but one analyzed movement, and Kanko et al.^23^ reported that global thigh and shank segment angles exhibited larger differences in rotational angles than in other planes. Our findings are in line with those of Huang et al.^31^, who identified the strongest agreement in flexion/extension movements and the poorest agreement in internal/external/inversion/eversion rotation angles. Additionally, the slope for flexion/extension kinematics was the lowest (-0.09) indicating the smallest change in bias with increasing MB angle. The second-best agreement in the extended BA analysis, was present in the knee abduction/adduction angles (bias = 1.42°, LoA = ± 8.96°). Given the pivotal role of knee kinematics in assessing injury risk and loading patterns during landing and cutting movements^32–35^, it is advantageous that the strongest agreements was identified here.

Our second hypothesis proposed that knee and ankle kinematics would demonstrate a stronger agreement between measurement systems compared to hip joint angles. Our findings offer partial support for this hypothesis: The smallest bias and random error were observed at the knee joint (bias = 3.34°; LoA = ± 11.38°), which is consistent with previous findings^13^. The mean bias and random error for hip joint angles (bias = 5.27°; LoA = ± 13.05°), however, indicated a stronger agreement than for ankle joint angles (bias = 5.27°; LoA = ± 14.19°). As the most distal joint of the three analyzed joints, the ankle experiences the most rapid deceleration and acceleration with each step^36^, which may induce motion artifacts. This may be a reason for the increased discrepancy between estimates.

A measurement error of up to 5° is considered as clinically acceptable^37^. Even though errors for flexion/extension angles and knee joint kinematics were generally lower than those for abduction/adduction and internal/external rotation angles and hip and ankle joint kinematics, respectively, the average values still exceeded this threshold. However, an absolute error of 3.3° (± 1.8°) for internal/external rotation at toe-off to 13.1° (± 9.8°) for abduction/adduction has been reported for knee joint kinematics during a dynamic task when comparing MB data against data from bone pins^38^. As such, our findings fall within this range. In another study comparing MB data against data from bone pins, conducted by Reinschmidt et al.^39^, the RMSD for individual subjects was found to range from 3.0° to 6.7 ° for knee flexion/extension, from 1.7° to 7.2° for knee abduction/adduction and from 2.0° to 6.7° for internal/external rotation during the stance phase in running. This resulted in kinematic data derived from MB that differed from the data derived from bone pins by 21% for knee flexion/extension, 70% for abduction/adduction, and 63% for internal/external rotation angles^39^. The challenges in measuring angles about the longitudinal axis of a segment align with limitations affecting both MB and ML motion capture systems as reported across different studies^39–41^ with different ML technologies, such as OpenPose, not reporting those angles at all due to insufficient information for calculation^42^. Therefore, the observed discrepancies between systems should be interpreted within the context that both measurement technologies have inherent limitations in tracking skeletal motion. Prior work showed, however, that Theia3D kinematics yield high intra- and inter-session reliability for lower limb kinematics^8,10,43^ supporting its use for comparison with Theia3D estimates only without comparing it to MB or other estimates.

The difficulty of accurately capturing rotational angles has implications for multiplanar motions, such as dynamic knee valgus (DKV), which is a critical consideration for practitioners evaluating COD tasks, as it plays a key role in assessing knee injury risk. We suggest to interpret absolute DKV angles with great caution and that they not bed referenced to MB values. Due to the systems’ high reliability^8,10,43^, it can still be useful for practitioners to assess relative changes rather than absolute values. It may be possible to investigate changes in DKV angles throughout the stance phase or pre- and post-interventions.

### Agreement across running directions

We hypothesized that the agreement during straight running and movements with small cutting angles would show a stronger agreement than during movements with a sharper cutting angle. Our results support this hypothesis for the random error. The results of both methodologies show a wider random spread in the data with a sharper cutting angle (see Table 2) It seems that the measuring technologies face greater challenges the more the movement differs from straight running. This may be caused by increased positive and negative accelerations during sharper angles when compared to smaller COD angles. Which may be supported by the fact that the random error at TD, where rapid limb deceleration occurs, was larger than at other time points (LoA = ± 13.59° vs. a mean of 12.59° for the other time points). Additionally, the ML system may be best trained for more common activities such as straight walking and running. Lastly, Benoit et al.^38^ showed that MB-derived knee joint kinematics exhibit a larger deviation from bone pin derived kinematics when performing a cutting movement than when walking. This indicates that cutting movements present measurement challenges that likely affect both systems, potentially explaining the increased discrepancies during movements performed at sharper cutting angles. This means that larger random deviations from MB estimates are expected when measuring sharper cutting angles.

### Agreement across running intensities

Our last hypothesis proposed that slower running speeds would demonstrate a stronger agreement than faster running speeds. Our findings provide mixed support for this hypothesis. When averaged across all directions, as it can be seen in Table 4, movements at slower speed do not show a higher agreement than movements performed at faster speeds. Defining the speed categories for each movement direction individually, however, revealed that there was only minimal change between slow and the fast categories for cutting directions above 45 degrees: 0.31 m/s for 90-degree, 0.43 m/s for 135 degree, and 0.33 m/s for 180-degree COD movements. Since those movements require substantial deceleration, this limited speed range is a logical consequence when measuring approach speed just before the ultimate foot contact. In contrast, the 45-degree and straight running directions showed larger differences between slow and fast intensities (0.68 m/s and 1.05 m/s, respectively). The results for these two movements support our hypothesis, demonstrating an increase in random error from slow through medium to fast speeds when using the extended BA analysis.

The speed-related increases in measurement discrepancies likely reflect different challenges faced by both systems. For the ML system, we observed slight image blurring at fast movement speeds, which may impair accurate landmark detection. This could potentially be improved using a fast shutter speed. For MB systems, Benoit et al.^38^ observed that cutting movements result in larger measurement errors compared to walking when compared to estimates derived from bone pin data, likely due to soft tissue artifacts occurring at dynamics movements. These system-specific challenges may both contribute to reduced agreement between systems at higher speeds. This means that speed-related reductions in agreement are most dominant when speed varies considerably. Ensuring a high frame rate and shutter speed is likely beneficial to improve the estimates.

## Limitations

The study has some limitations that must be acknowledged: 54% (408/756) of the fitted LMM models did not meet al.l the statistical assumptions, primarily violating the normality assumption for residuals. LMM models, however, have been shown to be relatively robust to this violation^44,45^. The sampling frequency was chosen to be 85 Hz as it offered the highest resolution for the available system. While 85 Hz is relatively high for a ML system, it may be insufficient to capture rapid movements accurately. A higher frame rate potentially leads to a stronger agreement between systems in general and especially during highly dynamic movements. Furthermore, the shutter speed should be selected with precision in order to minimize the occurrence of image blurring.

The exact anatomical landmarks used by Theia3D to create segments, as well as the segment coordinate definitions, are not available. The MB model we used is, therefore, not perfectly identical to the ML model. This may mean that, next to comparing different measurement technologies, we were comparing different anatomical models, which may be one cause for bias between kinematic estimates. Kanko et al.^13^ found that joint center positions between MB and ML systems had mean differences of up to 3.0 cm and 3.6 cm, respectively. This could be a cause for systematic bias between joint angle estimates.

Additionally, it is important to note that MB estimates vary from the motion measured with e.g., invasive technologies, as mentioned previously. In order to understand the discrepancies between ML estimates and the true underlying skeletal motion a comparison with other technologies is necessary. Nevertheless, given the widespread use of MB technology in biomechanics research, our results provide a valuable reference point for contextualizing ML findings within the framework of MB kinematic estimates.

## Conclusion

In summary, this study offers valuable insight into the agreement of lower body kinematics between ML and MB measurement systems during cutting movements. Practitioners can use our findings to determine if an ML system is suitable for their observed outcome parameters, depending on the context in which it is used. In general, lower limb flexion/extension angles demonstrate stronger agreement than lower limb abduction/adduction, which in turn demonstrate stronger agreement than angles about the long axis of a segment. Discrepancies between systems can be substantial even in the sagittal plane. Therefore, practitioners should avoid directly comparing absolute ML values to MB values and instead focus on relative changes in joint kinematics. We also observed larger discrepancies during sharper cutting angles. Taken together, these findings suggest that Theia3D estimates should not be used interchangeably with MB estimates. Due to the fact that prior studies have demonstrated a high reliability for the system, it may be a practical and time-efficient solution when a comparison to previous collected MB data is not of interest. To gain a deeper understanding of the agreement with skeletal motion, future studies should compare estimates with e.g., dynamic biplanar fluoroscopy.

## Supplementary Information

Below is the link to the electronic supplementary material.

Supplementary Material 1

## Data Availability

The datasets generated during and/or analyzed during the current study are available from the corresponding author on reasonable request.
