# Supplementary material for: Comparison of lower body joint kinematics during change of direction tasks estimated using a markerless and a markerbased method
Source: Sci Rep. 2025 Sep 30;15:34119. doi: 10.1038/s41598-025-21143-x (PMC12484822; doi:10.1038/s41598-025-21143-x)
Supplement: Supplementary file 1 — Supplementary Material 1 [file 41598_2025_21143_MOESM1_ESM.pdf]

Supplementary Table 1: Displayed are the results of the prediction band analysis for the agreement across lower extremity joints and movement planes.

Joint	Plane	Min Width (°)	Max Width (°)	Area (°·%stance)	Mean Bias (°)	Min Bias (°)	Max Bias (°)
Ankle	Frontal	33.81	48.59	4400.58	2.60	0.00	5.68
	Sagittal	24.71	46.60	3623.24	2.00	0.00	7.41
	Transverse	47.12	55.58	5064.74	8.43	4.21	10.84
Hip	Frontal	18.29	25.74	2244.44	0.68	0.01	1.60
	Sagittal	27.43	36.81	3338.94	10.77	7.77	11.82
	Transverse	46.20	56.68	5104.71	6.93	3.21	8.15
Knee	Frontal	16.09	38.48	3123.92	-0.92	-0.02	-2.28
	Sagittal	15.13	20.64	1797.03	1.17	0.05	1.99
	Transverse	41.00	55.52	4386.76	-10.93	-4.10	-13.12

Abbreviations: Max = maximal, Min = minimal

Supplementary Table 2: Displayed are the results of the extended BA analysis for the agreement across lower extremity joints and movement planes.

Event	Joint	Plane	Bias at mean MB angle (°)	±CI Bias (°)	Slope	±LoA (°)	±CI LoA (°)
TD	Ankle	Frontal	5.80	1.09	-0.51	14.52	3.98
		Sagittal	1.67	1.23	-0.07	9.99	2.68
		Transverse	6.93	1.96	-0.80	18.49	5.21
	Knee	Frontal	-0.52	1.11	-0.19	9.54	2.61
		Sagittal	-0.23	0.90	0.05	8.77	2.24
		Transverse	-9.48	1.03	-0.75	18.84	6.26
	Hip	Frontal	-0.20	1.23	-0.04	10.03	2.68
		Sagittal	10.11	1.50	-0.43	14.07	3.93
		Transverse	7.53	1.52	-0.62	18.07	6.22
Min	Ankle	Frontal	7.02	1.37	-0.33	13.23	3.81
		Sagittal	6.77	1.29	-0.24	11.16	3.09
		Transverse	10.53	2.38	-0.32	18.05	5.44
	Knee	Frontal	2.17	1.53	-0.41	11.42	3.61
		Sagittal	-0.05	0.94	0.11	6.73	1.79
		Transverse	-3.91	1.35	-0.59	18.11	5.76
	Hip	Frontal	1.05	1.05	-0.08	9.31	2.46

		Sagittal	7.96	2.12	0.05	12.62	3.75
		Transverse	10.24	0.80	-0.82	15.38	5.04
Max	Ankle	Frontal	-5.53	0.88	-0.41	10.46	2.92
		Sagittal	-1.45	1.42	-0.45	12.12	3.59
		Transverse	5.90	2.13	-0.37	18.25	5.16
	Knee	Frontal	-0.71	1.11	-0.48	9.21	2.51
		Sagittal	1.52	1.11	0.20	6.78	1.88
		Transverse	-14.55	1.41	-0.51	17.03	5.34
	Hip	Frontal	0.07	0.62	-0.17	7.97	2.04
		Sagittal	10.12	1.93	-0.20	15.96	4.50
		Transverse	2.76	1.46	-0.57	16.64	5.20
TO	Ankle	Frontal	0.09	1.32	-0.41	11.58	3.37
		Sagittal	7.34	1.27	-0.23	11.25	3.07
		Transverse	4.27	2.72	-0.01	19.98	5.44
	Knee	Frontal	-2.27	0.76	-0.61	5.65	1.52
		Sagittal	0.45	1.05	0.11	6.35	1.81
		Transverse	-4.25	1.35	-0.58	18.07	5.66
	Hip	Frontal	1.45	0.79	-0.11	7.95	2.06
		Sagittal	7.89	2.13	0.04	12.72	3.78
		Transverse	3.27	0.71	-0.85	15.87	4.98

Abbreviations: CI Confidence Interval, MB Markerbased, LoA Limit of Agreement, TD Touchdown, TO Toe-off.

Supplementary Table 3: Displayed are the results of the prediction band analysis for the agreement across lower extremity joints and movement planes and movement directions.

Joint	Plane	Movement Direction (°)	Min Width (°)	Max Width (°)	Area (°·%stance)	Mean Bias (°)	Min Bias (°)	Max Bias (°)
Ankle	Frontal	135	33.72	43.33	3856.96	-7.25	0.76	-9.89
		180	33.39	41.13	3823.46	-8.83	0.30	-11.89
		45	20.02	30.84	2429.41	1.01	-0.01	7.51
		90	26.25	40.62	2932.48	5.30	-0.74	8.81
		Straight	12.19	18.68	1618.59	8.53	1.61	11.21
	Sagittal	135	23.85	59.12	4249.01	3.58	0.15	8.72
		180	31.03	56.84	4478.71	3.14	-0.07	9.47
		45	20.40	27.22	2307.89	1.81	0.01	6.32
		90	25.00	46.80	3509.84	2.90	-0.02	8.55

		Straight	17.78	27.77	2263.47	3.14	1.61	5.54
	Transverse	135	44.88	71.29	5322.77	9.47	2.49	13.42
		180	46.22	66.50	5224.05	7.41	0.19	12.93
		45	33.17	41.75	3804.26	6.72	0.00	10.60
		90	40.44	56.76	4683.89	7.84	3.16	12.32
		Straight	30.62	42.25	3411.77	13.37	4.13	18.05
Hip	Frontal	135	18.94	27.71	2357.19	1.13	0.06	2.39
		180	17.36	25.36	2227.80	3.14	1.16	3.79
		45	15.52	20.85	1821.98	-1.21	-0.08	-1.96
		90	14.17	26.41	1979.94	-0.57	0.00	-0.96
		Straight	10.17	19.15	1388.01	1.01	0.02	2.28
	Sagittal	135	29.42	39.19	3411.91	12.66	7.29	14.95
		180	29.44	40.54	3549.59	14.21	10.06	16.43
		45	23.09	28.36	2593.01	9.21	6.00	11.34
		90	21.87	32.29	2882.61	11.20	7.06	13.18
		Straight	21.20	27.57	2607.47	7.16	5.60	8.28
	Transverse	135	43.40	64.52	4938.87	7.61	4.60	10.32
		180	44.76	72.53	5168.35	11.69	7.15	14.35
		45	35.30	45.06	4012.81	6.76	0.57	10.79
		90	33.80	55.51	4562.19	5.10	0.83	8.49
		Straight	37.50	44.45	4116.61	4.26	0.02	9.79
Knee	Frontal	135	15.77	41.85	3248.10	1.33	0.01	3.00
		180	13.83	37.92	3096.56	2.35	0.01	3.87
		45	12.87	33.86	2615.97	1.47	-0.03	2.92
		90	15.14	44.01	2922.61	-1.23	0.00	-4.46
		Straight	14.79	31.98	2549.21	-2.37	0.02	-4.62
	Sagittal	135	12.96	18.48	1503.11	2.56	-0.01	3.80
		180	14.57	21.60	1692.66	2.63	1.03	3.49
		45	9.67	16.69	1349.98	-0.91	0.03	-2.48
		90	14.10	21.19	1670.18	1.44	0.02	2.57
		Straight	11.33	17.25	1378.98	-1.79	-0.12	-3.70
	Transverse	135	41.65	63.98	4598.05	-10.71	-5.91	-12.49
		180	38.00	64.79	4604.97	-10.54	-6.32	-13.28
		45	36.75	48.83	4028.27	-11.21	-4.23	-14.62
		90	37.80	54.95	4295.69	-9.82	-3.90	-12.18

		Straight	31.60	37.47	3443.48	-12.70	-1.76	-17.38

Abbreviations: Max = maximal, Min = minimal

Supplementary Table 4: Displayed are the results of the extended BA analysis for the agreement across lower extremity joints and movement planes and movement directions.

Event	Joint	Plane	Movement Direction (°)	Bias at mean MB angle (°)	±CI Bias (°)	Slope	±LoA (°)	±CI LoA (°)
TD	Ankle	Frontal	Straight	1.61	0.87	-0.51	6.55	2.18
			45	7.03	1.31	-0.72	10.20	3.66
			90	8.50	1.12	-0.69	13.86	5.03
			135	6.29	1.42	-0.73	14.36	5.06
			180	5.52	1.48	-0.59	13.51	4.59
		Sagittal	Straight	1.73	1.08	-0.26	7.59	2.51
			45	3.11	1.08	-0.18	8.17	2.64
			90	2.39	1.56	-0.17	8.99	2.88
			135	0.80	1.51	-0.11	8.65	2.64
			180	0.16	1.74	-0.13	9.71	3.32
		Transverse	Straight	3.73	1.64	-0.77	14.40	5.58
			45	1.46	2.35	-0.59	15.98	5.90
			90	7.16	2.65	-0.91	15.73	5.13
			135	10.33	2.63	-0.83	15.94	5.28
			180	12.42	2.67	-0.46	17.09	5.60
	Knee	Frontal	Straight	0.53	1.17	-0.59	7.95	2.24
			45	0.80	1.01	-0.55	7.33	2.67
			90	-2.24	0.98	-0.78	8.46	2.94
			135	-1.56	1.09	-0.49	7.70	2.83
			180	-0.29	0.94	-0.69	8.00	2.85
		Sagittal	Straight	-3.56	0.77	0.07	4.43	1.28
			45	-1.45	0.88	0.13	5.43	1.60
			90	0.91	1.14	0.15	6.89	2.04
			135	1.29	1.16	0.11	5.72	1.91
			180	1.81	1.53	0.01	7.52	2.65
		Transverse	Straight	-8.59	1.09	-0.80	15.37	5.19
			45	-8.03	1.40	-0.78	18.74	7.09

			90	-7.54	1.74	-0.84	19.36	7.31
			135	-10.59	1.47	-0.78	21.69	8.25
			180	-12.93	1.77	-0.74	20.48	7.52
	Hip	Frontal	Straight	0.69	1.22	-0.41	7.28	2.67
			45	-1.35	1.13	-0.24	7.78	2.52
			90	-0.97	1.62	-0.26	10.89	3.41
			135	-0.61	0.86	-0.46	8.85	2.79
			180	1.22	0.73	-0.44	7.12	2.20
		Sagittal	Straight	5.18	1.96	0.11	8.89	3.08
			45	6.46	1.65	-0.12	9.47	3.28
			90	9.86	1.92	-0.41	12.96	4.57
			135	13.46	2.09	-0.18	11.82	4.08
			180	15.98	2.24	-0.26	11.93	4.32
		Transverse	Straight	9.36	0.91	-0.84	15.86	5.98
			45	8.38	1.34	-0.77	14.53	5.63
			90	5.04	1.53	-0.89	17.32	6.62
			135	6.48	1.52	-0.84	19.59	7.88
			180	8.69	1.64	-0.79	21.99	8.93
Min	Ankle	Frontal	Straight	9.46	0.70	-0.58	5.24	1.82
			45	6.21	1.32	-0.68	10.24	3.61
			90	8.09	1.01	-0.82	13.86	5.21
			135	5.97	1.40	-0.79	14.51	5.13
			180	5.12	1.40	-0.65	13.14	4.53
		Sagittal	Straight	5.69	1.92	-0.04	11.10	3.48
			45	6.21	1.39	-0.17	10.22	2.95
			90	7.62	1.23	-0.30	9.74	2.89
			135	7.05	1.88	-0.47	11.24	3.51
			180	7.32	1.08	-0.37	10.48	3.36
		Transverse	Straight	14.19	1.46	-0.85	11.77	4.55
			45	8.73	2.08	-0.74	13.49	5.16
			90	9.76	2.12	-0.78	16.97	6.52
			135	11.48	2.58	-0.70	18.06	6.59
			180	8.30	2.31	-0.60	17.58	6.55
	Knee	Frontal	Straight	-0.86	1.17	-0.88	8.46	2.91
			45	3.39	1.40	-0.61	10.03	3.81
			90	1.98	1.77	-0.52	11.23	3.93
			135	2.53	1.94	-0.51	12.95	4.61

			180	3.95	1.44	-0.57	11.07	4.14
		Sagittal	Straight	-1.16	0.94	0.12	6.02	1.70
			45	-1.10	0.92	0.03	5.59	1.66
			90	0.34	0.98	0.06	6.06	1.77
			135	1.14	1.18	0.12	5.87	1.91
			180	0.63	1.20	0.02	6.12	2.07
		Transverse	Straight	-2.16	1.11	-0.75	14.29	4.67
			45	-4.12	1.44	-0.76	14.47	5.41
			90	-2.39	1.67	-0.70	18.68	7.03
			135	-6.06	2.03	-0.59	19.09	7.07
			180	-5.09	2.09	-0.57	18.96	7.01
	Hip	Frontal	Straight	2.04	0.74	0.00	4.47	1.35
			45	-0.93	1.09	-0.17	7.12	2.19
			90	-0.39	1.14	-0.26	9.38	2.78
			135	1.36	0.85	-0.45	8.85	3.00
			180	3.24	1.03	-0.31	7.90	2.67
		Sagittal	Straight	6.88	1.53	-0.52	9.92	3.56
			45	7.97	1.85	-0.17	9.54	3.34
			90	7.06	1.99	-0.03	10.12	3.49
			135	7.85	2.57	0.06	12.92	4.36
			180	10.28	2.83	0.09	14.17	4.77
		Transverse	Straight	7.18	0.76	-0.92	15.44	5.91
			45	9.34	1.01	-0.89	15.21	6.09
			90	9.34	1.01	-0.74	14.89	5.51
			135	10.08	1.75	-0.70	15.40	5.65
			180	15.73	1.81	-0.63	15.95	5.87
Max	Ankle	Frontal	Straight	3.17	0.69	-0.93	5.94	2.02
			45	-1.86	1.11	-0.48	7.79	2.62
			90	-7.42	0.91	-0.45	9.32	3.22
			135	-10.49	1.32	-0.55	12.65	4.15
			180	-11.48	1.35	-0.48	11.77	4.13
		Sagittal	Straight	2.88	1.70	-0.16	8.69	3.36
			45	-1.51	1.44	-0.15	7.60	2.72
			90	-2.03	1.70	-0.32	11.72	4.32
			135	-3.67	1.98	-0.34	13.37	4.82
			180	-2.96	2.59	-0.15	14.15	5.43
		Transverse	Straight	10.82	1.20	-0.77	10.85	4.20

			45	5.02	2.39	-0.32	14.31	5.16
			90	3.54	2.76	-0.36	17.45	5.87
			135	3.93	2.65	-0.60	16.71	5.62
			180	6.22	2.71	-0.65	18.12	5.90
	Knee	Frontal	Straight	-0.29	1.39	-0.84	9.18	2.75
			45	-1.32	1.13	-0.73	7.21	2.30
			90	-2.55	1.33	-0.68	7.46	2.45
			135	-0.78	1.40	-0.47	8.59	2.88
			180	1.53	1.16	-0.72	8.05	2.63
		Sagittal	Straight	-2.31	1.02	0.14	4.75	1.77
			45	0.31	0.86	0.13	4.05	1.40
			90	2.85	1.14	0.01	5.50	1.97
			135	3.59	1.08	0.02	5.34	1.85
			180	3.36	1.20	-0.01	5.91	2.11
		Transverse	Straight	-17.51	1.39	-0.75	14.18	4.96
			45	-14.57	1.61	-0.71	15.93	6.16
			90	-12.58	1.63	-0.76	15.69	5.95
			135	-14.27	1.30	-0.75	15.54	5.91
			180	-13.77	1.83	-0.80	15.80	5.68
	Hip	Frontal	Straight	-1.39	0.84	-0.44	5.36	1.90
			45	-1.76	0.72	-0.38	7.18	2.39
			90	-0.24	0.41	-0.25	7.31	1.93
			135	1.52	0.85	-0.33	7.56	2.31
			180	2.33	0.95	-0.37	7.69	2.46
		Sagittal	Straight	5.68	1.93	0.02	9.30	3.30
			45	6.69	1.64	-0.17	9.48	3.41
			90	10.85	1.97	-0.33	12.92	4.68
			135	13.35	2.14	-0.30	12.99	4.88
			180	14.44	2.08	-0.32	15.35	5.71
		Transverse	Straight	0.62	0.82	-0.95	15.49	5.84
			45	4.89	1.53	-0.66	13.31	5.29
			90	1.02	1.54	-0.82	15.23	5.78
			135	2.79	1.54	-0.81	14.32	5.48
			180	4.81	1.69	-0.72	20.26	8.20
TO	Ankle	Frontal	Straight	5.40	0.77	-0.56	5.59	1.72
			45	1.44	1.38	-0.39	8.46	2.80
			90	-0.49	1.26	-0.75	11.44	3.84

			135	-2.96	1.79	-0.56	14.07	4.93
			180	-3.17	1.70	-0.56	14.13	5.03
		Sagittal	Straight	5.69	1.92	-0.04	11.10	3.48
			45	6.33	1.45	-0.13	10.36	2.99
			90	8.30	1.24	-0.29	10.07	2.90
			135	8.06	1.57	-0.26	10.63	3.15
			180	8.44	1.58	-0.17	10.62	3.43
		Transverse	Straight	10.89	1.24	-0.71	10.78	4.16
			45	5.43	2.40	-0.35	14.59	5.31
			90	2.57	2.94	-0.17	16.34	5.58
			135	2.05	3.12	-0.40	17.89	5.82
			180	-0.08	3.24	-0.16	18.46	6.04
	Knee	Frontal	Straight	-4.10	1.05	-0.78	5.45	1.68
			45	-2.63	0.76	-0.68	4.55	1.52
			90	-2.52	0.95	-0.67	5.64	1.80
			135	-1.03	1.03	-0.36	5.92	1.88
			180	-0.99	0.74	-0.52	4.98	1.59
		Sagittal	Straight	0.07	1.09	0.18	5.84	1.88
			45	-0.79	0.98	0.08	5.57	1.70
			90	0.52	1.16	0.12	5.71	1.88
			135	1.42	1.15	0.11	5.61	1.81
			180	1.12	1.27	0.08	6.58	2.16
		Transverse	Straight	-1.57	1.18	-0.71	14.35	4.58
			45	-4.46	1.53	-0.83	14.69	5.46
			90	-3.32	1.65	-0.63	18.23	6.73
			135	-6.91	1.97	-0.58	18.71	6.73
			180	-5.25	1.99	-0.62	19.07	6.99
	Hip	Frontal	Straight	2.17	0.72	0.04	4.53	1.35
			45	-0.26	1.10	-0.19	6.72	2.01
			90	-0.02	0.82	-0.15	7.15	1.96
			135	2.45	0.82	-0.29	7.29	2.33
			180	3.01	1.12	-0.15	7.02	2.23
		Sagittal	Straight	6.88	1.53	-0.52	9.92	3.56
			45	7.97	1.85	-0.17	9.54	3.34
			90	7.06	1.98	-0.02	10.16	3.48
			135	7.69	2.63	0.08	12.81	4.41
			180	10.08	2.92	0.06	14.59	4.98

		Transverse	Straight	-1.40	1.13	-1.02	15.90	5.95
			45	1.57	1.06	-0.84	15.72	6.18
			90	1.27	1.15	-0.76	14.17	5.06
			135	4.92	1.69	-0.63	15.23	5.84
			180	10.55	1.51	-0.69	17.90	6.52

Abbreviations: CI = Confidence Interval, LoA = Limit of Agreement, TD = Touchdown, TO = Toe-off.

Supplementary Table 5: Displayed are the results of the prediction band analysis for the agreement across lower extremity joints, movement planes, movement directions and movement intensities.

Joint	Plane	Movement Direction (°)	Speed	Min Width (°)	Max Width (°)	Area (°·%stance)	Mean Bias (°)	Min Bias (°)	Max Bias (°)
Ankle	Frontal	Straight	Slow	12.04	18.56	1575.41	8.02	1.46	11.04
			Medium	13.78	21.14	1767.00	8.89	1.58	11.71
			Fast	9.16	22.93	1513.14	8.38	1.62	10.59
		45	Slow	15.50	27.77	2210.56	1.54	0.54	7.02
			Medium	18.23	31.33	2312.71	1.28	-0.04	4.39
			Fast	18.23	31.77	2244.08	1.74	0.03	9.84
		90	Slow	23.53	55.21	3065.51	4.12	0.16	8.16
			Medium	25.08	40.18	2898.98	4.50	0.74	10.34
			Fast	20.18	48.10	2684.24	6.26	-0.82	8.42
		135	Slow	28.21	42.40	3507.77	7.55	-0.36	-10.37
			Medium	31.03	39.54	3567.37	8.07	-0.17	-11.03
			Fast	35.47	52.31	4552.82	6.85	0.16	-10.17
		180	Slow	23.30	41.66	3622.05	8.77	-0.54	-12.17
			Medium	34.38	46.17	4180.05	10.72	-0.75	-13.88
			Fast	33.69	45.34	3926.45	8.07	1.10	-11.10
	Sagittal	Straight	Slow	17.64	24.95	2347.40	2.46	0.67	4.41
			Medium	16.40	27.77	2167.31	2.78	1.56	5.02
			Fast	19.35	34.05	2454.02	1.70	0.55	4.32
		45	Slow	14.96	24.49	1906.83	1.68	-0.01	6.71
			Medium	20.41	27.13	2313.55	2.03	0.00	5.60
			Fast	18.82	29.19	2535.36	2.49	-0.11	5.72
		90	Slow	28.39	38.29	3347.85	3.04	-0.03	8.67
			Medium	20.47	39.92	3017.80	2.60	-0.07	8.18
			Fast	17.41	61.54	3652.86	3.21	0.16	6.97

		135	Slow	18.52	43.67	3362.25	3.34	0.00	10.69
			Medium	21.96	53.67	3932.42	4.09	0.05	8.38
			Fast	21.46	70.09	4520.55	4.30	-0.02	-7.74
		180	Slow	23.84	57.59	4128.39	3.00	0.06	9.69
			Medium	26.84	51.01	4060.79	2.88	-0.04	10.32
			Fast	36.58	75.05	5523.84	2.75	-0.04	9.00
	Transverse	Straight	Slow	29.22	39.74	3382.44	14.59	5.57	20.26
			Medium	31.26	44.95	3660.53	13.21	2.64	18.29
			Fast	20.73	32.43	2577.93	12.28	3.18	17.57
		45	Slow	31.54	46.68	4062.10	8.63	3.51	12.56
			Medium	29.83	42.29	3725.85	6.71	0.02	10.86
			Fast	29.85	43.43	3572.24	6.60	-0.10	10.09
		90	Slow	43.13	61.53	5248.61	5.36	0.07	8.92
			Medium	32.15	50.47	3955.03	6.43	2.13	10.47
			Fast	37.55	77.81	5261.48	7.34	3.26	11.53
		135	Slow	47.42	72.89	5541.88	10.29	3.26	14.46
			Medium	42.81	74.62	5785.49	8.27	2.14	12.38
			Fast	37.52	77.32	5448.75	12.08	4.28	16.10
		180	Slow	44.57	72.94	5544.65	7.61	0.08	13.38
			Medium	40.55	62.23	4866.34	5.75	-0.03	11.55
			Fast	48.87	74.63	5600.11	6.00	-0.31	13.43
Knee	Frontal	Straight	Slow	10.08	25.79	1805.30	1.92	-0.02	-3.30
			Medium	14.42	32.66	2491.73	2.44	-0.02	-5.00
			Fast	15.15	31.64	2557.46	2.63	0.10	-4.89
		45	Slow	11.48	34.41	2310.22	2.82	0.08	6.33
			Medium	11.50	33.95	2557.99	1.75	0.06	2.70
			Fast	14.87	34.72	2666.41	1.50	0.03	3.04
		90	Slow	14.62	44.46	3013.49	1.40	0.00	3.45
			Medium	15.62	43.19	2897.75	1.25	-0.02	-3.88
			Fast	15.30	47.76	3022.19	2.14	-0.66	-4.38
		135	Slow	13.61	44.62	3250.73	1.81	0.02	3.81
			Medium	15.27	53.26	3739.76	1.29	0.00	-3.47
			Fast	18.46	43.06	3083.67	1.10	0.01	-2.76
		180	Slow	13.46	46.12	3454.76	1.89	-0.05	4.12
			Medium	13.98	41.25	3179.12	1.95	-0.02	3.34
			Fast	14.12	38.21	2948.41	2.53	0.00	4.69

	Sagittal	Straight	Slow	7.84	15.54	1162.03	2.36	-0.59	-4.33
			Medium	11.39	17.78	1412.41	1.77	0.00	-3.85
			Fast	10.36	13.41	1228.43	1.81	0.01	-3.91
		45	Slow	10.27	16.99	1447.40	1.12	0.01	-3.46
			Medium	10.83	18.19	1419.21	0.84	0.00	-2.12
			Fast	8.72	16.68	1216.67	0.63	0.02	-1.71
		90	Slow	14.52	23.27	1949.91	1.46	-0.03	2.79
			Medium	13.35	20.43	1571.69	1.64	-0.01	2.74
			Fast	11.31	19.72	1352.36	1.74	0.00	3.10
		135	Slow	14.29	18.47	1637.54	2.53	0.00	3.97
			Medium	12.40	18.82	1488.78	3.29	0.52	4.63
			Fast	8.63	17.45	1167.46	1.73	0.05	2.87
		180	Slow	16.69	23.97	1997.25	2.24	0.67	3.29
			Medium	13.05	21.76	1525.92	2.94	1.54	3.73
			Fast	13.98	21.34	1657.03	2.58	0.41	3.52
	Transverse	Straight	Slow	29.60	38.55	3444.44	12.34	-1.42	-16.43
			Medium	30.61	36.92	3365.61	11.72	0.10	-15.65
			Fast	30.59	38.71	3537.03	12.88	-1.42	-18.45
		45	Slow	30.62	42.05	3853.65	14.04	-5.34	-17.18
			Medium	35.87	43.92	3999.06	11.03	-4.45	-15.53
			Fast	36.91	53.02	4055.83	8.09	-2.55	-11.18
		90	Slow	39.81	59.47	4604.04	9.97	-1.93	-12.19
			Medium	29.89	51.67	3631.29	7.84	-1.80	-10.70
			Fast	35.08	47.97	4301.87	9.01	-3.66	-11.71
		135	Slow	40.98	71.55	5040.14	10.21	-5.11	-12.41
			Medium	36.46	57.30	4328.82	14.20	-10.68	-16.49
			Fast	42.93	60.73	4928.08	10.36	-6.11	-13.00
		180	Slow	45.19	70.56	5137.16	10.36	-4.60	-14.77
			Medium	37.38	60.91	4536.68	10.80	-6.09	-14.87
			Fast	28.39	59.27	3835.54	10.08	-4.34	-13.61
Hip	Frontal	Straight	Slow	8.20	14.59	1130.34	1.36	-0.06	2.57
			Medium	10.67	21.02	1502.55	1.18	0.00	-2.42
			Fast	9.69	21.75	1434.54	1.02	0.00	2.00
		45	Slow	12.78	23.64	1802.14	1.56	0.02	-3.08
			Medium	14.20	23.65	1895.26	1.62	-0.26	-2.82
			Fast	16.17	22.84	1846.73	2.06	-0.84	-2.80

		90	Slow	13.27	27.08	2060.77	1.55	-0.27	-2.84
			Medium	14.05	24.94	1869.94	0.49	0.00	0.89
			Fast	13.42	30.04	2056.10	2.00	-0.70	-2.93
		135	Slow	13.48	24.18	1928.22	0.82	0.01	2.26
			Medium	20.03	31.98	2627.93	1.94	-0.01	3.26
			Fast	15.06	21.83	1754.13	0.93	0.00	-2.30
		180	Slow	20.01	25.35	2286.23	2.10	0.37	3.29
			Medium	15.43	25.95	2124.19	4.07	2.53	6.10
			Fast	17.06	27.86	2375.77	3.91	1.56	4.96
	Sagittal	Straight	Slow	19.70	27.51	2470.58	7.19	5.13	7.77
			Medium	21.71	28.65	2676.06	5.88	4.34	7.20
			Fast	19.88	25.95	2396.44	5.96	4.37	7.59
		45	Slow	22.75	29.91	2705.06	8.57	5.61	10.48
			Medium	20.99	29.16	2682.95	9.82	7.21	11.71
			Fast	17.33	20.74	1932.55	9.76	6.16	11.96
		90	Slow	28.76	37.83	3449.35	11.22	7.43	13.77
			Medium	21.97	38.23	3102.53	9.12	5.71	10.69
			Fast	16.38	29.45	2385.40	11.99	7.23	14.45
		135	Slow	30.41	36.24	3328.13	14.44	9.67	17.17
			Medium	27.68	38.27	3273.10	12.77	7.69	15.12
			Fast	18.95	28.04	2367.70	10.96	4.81	13.93
		180	Slow	31.14	40.10	3603.26	14.80	11.47	16.71
			Medium	28.04	38.42	3308.07	14.97	10.38	17.96
			Fast	23.81	40.09	3466.00	12.86	8.53	16.12
	Transverse	Straight	Slow	27.51	35.64	3198.29	3.80	0.10	7.09
			Medium	41.84	48.11	4600.33	3.72	0.09	7.58
			Fast	37.23	52.28	4392.95	6.39	1.91	11.19
		45	Slow	30.39	44.33	3635.70	9.47	4.00	13.79
			Medium	36.14	46.91	4249.58	7.62	2.30	10.11
			Fast	28.89	40.00	3503.02	5.72	-0.03	10.56
		90	Slow	38.00	62.59	5202.34	4.74	0.18	8.11
			Medium	34.25	57.86	4424.62	5.60	1.41	8.49
			Fast	27.36	60.58	4283.46	4.56	0.11	7.55
		135	Slow	37.11	59.44	4447.00	10.64	7.98	13.56
			Medium	48.13	75.73	5599.22	8.06	4.81	10.51
			Fast	21.54	56.22	3496.45	7.26	3.48	10.70

		180	Slow	42.33	64.60	5193.67	13.23	9.76	16.65
			Medium	41.08	71.69	5213.69	9.20	3.29	12.64
			Fast	40.79	74.21	5123.29	11.38	6.08	15.75

Abbreviations: Max = maximal, Min = minimal

Supplementary Table 6: Displayed are the results of the extended BA analysis for the agreement across lower extremity joints, movement planes, movement directions and movement intensities.

Event	Joint	Plane	Movement Direction (°)	Speed	Bias at mean MB angle (°)	±CI Bias (°)	Slope	±LoA (°)	±CI LoA (°)
TD	Ankle	Frontal	Straight	Slow	0.96	1.17	-0.38	5.91	2.36
				Medium	1.36	1.07	-0.35	7.64	2.88
				Fast	2.55	1.31	-0.25	8.07	2.71
			45	Slow	1.98	1.11	-0.16	7.50	2.73
				Medium	3.58	1.41	-0.22	8.37	3.10
				Fast	3.83	1.30	-0.20	8.55	3.21
			90	Slow	3.01	2.18	-0.15	10.61	4.07
				Medium	2.28	1.50	-0.13	7.95	2.91
				Fast	1.13	1.47	-0.15	7.34	2.82
			135	Slow	1.16	1.69	-0.16	8.07	3.03
				Medium	1.12	1.79	-0.03	9.26	3.22
				Fast	-0.25	1.32	0.02	8.07	3.02
			180	Slow	0.13	1.80	-0.23	11.04	4.67
				Medium	1.45	1.91	-0.04	8.92	3.40
				Fast	-0.46	2.24	-0.09	9.96	4.06
		Sagittal	Straight	Slow	5.02	1.83	-0.04	8.85	3.23
				Medium	5.62	1.97	-0.06	9.96	3.47
				Fast	6.25	2.17	-0.26	11.85	4.09
			45	Slow	6.93	1.07	-0.40	8.31	2.75
				Medium	5.72	1.39	-0.28	8.93	2.86
				Fast	6.56	2.14	-0.13	12.21	4.27
			90	Slow	8.45	1.62	-0.41	10.70	4.03
				Medium	7.74	0.94	-0.35	10.86	3.75
				Fast	6.92	1.95	-0.22	9.65	4.14
			135	Slow	8.66	2.07	-0.52	11.81	4.26
				Medium	7.50	2.20	-0.45	11.08	3.83
				Fast	5.41	2.63	-0.37	11.04	4.63

			180	Slow	7.67	1.33	-0.38	10.40	3.97
				Medium	7.33	1.26	-0.54	11.08	4.18
				Fast	6.80	1.51	-0.34	10.68	4.21
		Transverse	Straight	Slow	3.14	1.32	-0.60	8.58	3.66
				Medium	2.86	1.17	-0.66	8.64	3.44
				Fast	2.67	1.73	-0.30	9.20	3.78
			45	Slow	-0.78	1.42	-0.18	6.60	2.67
				Medium	-1.78	1.39	-0.27	6.94	2.71
				Fast	-1.97	1.76	-0.20	8.36	3.46
			90	Slow	-1.19	2.06	-0.32	13.04	5.69
				Medium	-1.84	1.62	-0.44	11.04	4.42
				Fast	-2.81	2.53	-0.29	12.95	6.34
			135	Slow	-2.59	1.67	-0.43	12.94	5.19
				Medium	-3.73	2.37	-0.35	13.48	5.63
				Fast	-4.65	2.78	-0.31	13.70	6.19
			180	Slow	-3.20	3.80	-0.11	16.49	7.71
				Medium	-2.50	2.12	-0.28	12.78	5.24
				Fast	-3.14	2.75	-0.30	15.07	6.51
	Knee	Frontal	Straight	Slow	5.02	1.83	-0.04	8.85	3.23
				Medium	5.62	1.97	-0.06	9.96	3.47
				Fast	6.25	2.17	-0.26	11.85	4.09
			45	Slow	7.04	1.16	-0.38	8.59	2.84
				Medium	5.84	1.44	-0.22	9.01	2.89
				Fast	6.66	2.10	-0.10	12.30	4.22
			90	Slow	9.10	1.58	-0.51	11.80	4.49
				Medium	8.81	1.31	-0.22	10.25	3.50
				Fast	7.72	2.06	-0.13	9.14	4.01
			135	Slow	9.59	1.52	-0.29	10.83	3.61
				Medium	8.19	2.02	-0.38	10.93	3.74
				Fast	6.46	1.96	-0.12	9.34	3.61
			180	Slow	8.91	1.90	-0.23	11.70	4.65
				Medium	8.15	1.75	-0.39	11.41	4.37
				Fast	7.81	1.91	-0.17	10.40	4.06
		Sagittal	Straight	Slow	1.55	0.79	-0.61	4.78	1.84
				Medium	1.66	0.94	-0.38	5.82	2.11
				Fast	1.73	1.06	-0.54	8.28	3.06
			45	Slow	6.73	1.37	-0.89	9.09	3.66

				Medium	5.91	1.36	-0.68	10.53	3.92
				Fast	8.28	1.22	-0.68	10.38	4.33
			90	Slow	8.04	1.65	-0.64	14.11	5.79
				Medium	9.13	1.43	-0.68	13.25	5.36
				Fast	8.30	1.07	-0.76	15.71	7.73
			135	Slow	6.04	1.79	-0.75	14.52	6.06
				Medium	5.61	2.10	-0.65	14.56	5.83
				Fast	6.85	1.22	-0.74	14.73	6.60
			180	Slow	4.54	2.02	-0.41	13.86	5.67
				Medium	5.43	1.99	-0.66	12.72	4.68
				Fast	6.48	1.80	-0.70	13.26	5.82
		Transverse	Straight	Slow	9.08	0.70	-0.70	4.87	2.07
				Medium	9.98	0.86	-0.57	5.78	2.15
				Fast	9.41	0.85	-0.47	5.29	1.97
			45	Slow	6.00	1.38	-0.75	8.66	3.41
				Medium	5.33	1.52	-0.76	10.69	4.03
				Fast	7.63	1.39	-0.69	9.88	4.05
			90	Slow	8.47	1.56	-0.82	16.21	7.08
				Medium	8.65	1.22	-0.79	13.16	5.38
				Fast	7.58	0.94	-0.79	15.41	7.76
			135	Slow	5.56	1.69	-0.78	14.54	6.09
				Medium	5.14	2.10	-0.73	15.07	6.05
				Fast	6.62	1.26	-0.76	15.04	6.73
			180	Slow	4.13	2.00	-0.46	13.99	5.62
				Medium	4.82	1.72	-0.71	11.86	4.37
				Fast	6.22	1.47	-0.76	13.46	5.94
	Hip	Frontal	Straight	Slow	2.78	0.65	-0.98	4.43	1.70
				Medium	2.85	0.75	-0.63	5.41	2.07
				Fast	3.84	0.86	-0.95	8.07	3.27
			45	Slow	-0.63	1.30	-0.56	7.91	3.18
				Medium	-2.02	1.12	-0.49	7.44	2.79
				Fast	-3.22	1.39	-0.74	9.81	4.15
			90	Slow	-6.74	1.38	-0.37	10.14	4.18
				Medium	-6.24	1.11	-0.48	8.57	3.39
				Fast	-9.54	1.04	-0.59	9.36	4.24
			135	Slow	-10.73	1.61	-0.56	11.93	4.37
				Medium	-10.06	2.01	-0.57	12.33	4.99

				Fast	-11.49	1.72	-0.52	11.94	5.25
			180	Slow	-10.86	1.46	-0.46	12.29	5.06
				Medium	-11.65	1.78	-0.58	12.47	4.77
				Fast	-12.13	1.51	-0.58	11.49	4.84
		Sagittal	Straight	Slow	5.48	0.84	-0.75	5.84	2.24
				Medium	5.01	1.00	-0.77	4.92	1.77
				Fast	5.88	0.82	-0.43	4.78	1.70
			45	Slow	1.51	1.62	-0.49	7.46	2.88
				Medium	1.54	1.50	-0.45	8.77	3.20
				Fast	1.33	1.58	-0.51	9.41	3.52
			90	Slow	0.26	1.99	-0.82	16.15	6.86
				Medium	0.66	1.35	-0.72	9.66	3.66
				Fast	-1.81	1.02	-0.62	10.47	4.24
			135	Slow	-2.30	1.88	-0.82	13.64	5.44
				Medium	-3.54	2.54	-0.42	13.57	5.59
				Fast	-4.24	2.16	-0.50	13.92	6.40
			180	Slow	-3.63	2.36	-0.56	15.30	6.76
				Medium	-3.96	1.94	-0.69	14.59	5.46
				Fast	-2.78	1.66	-0.62	12.47	5.18
		Transverse	Straight	Slow	4.75	1.59	-0.89	13.83	6.00
				Medium	3.41	1.51	-1.10	16.37	6.81
				Fast	3.40	1.50	-0.86	14.19	5.89
			45	Slow	3.19	2.83	-0.55	15.23	6.36
				Medium	0.12	2.32	-0.65	15.00	5.93
				Fast	0.77	1.74	-0.87	16.09	6.79
			90	Slow	8.35	3.34	-0.76	17.39	7.08
				Medium	6.53	2.87	-0.84	14.43	5.41
				Fast	6.68	3.14	-1.28	17.95	8.39
			135	Slow	11.00	2.69	-0.85	15.74	6.52
				Medium	11.09	3.15	-0.80	14.49	5.39
				Fast	8.21	3.50	-0.73	17.54	7.80
			180	Slow	14.20	2.67	-0.70	20.11	7.82
				Medium	11.25	3.02	-0.22	14.77	5.68
				Fast	12.05	3.23	-0.65	15.47	6.08
Min	Ankle	Frontal	Straight	Slow	14.30	1.65	-0.86	11.96	5.33
				Medium	14.89	1.68	-0.92	12.33	5.06
				Fast	13.29	1.53	-0.96	12.56	5.16

			45	Slow	9.37	2.74	-0.82	14.55	6.37
				Medium	8.14	2.15	-0.65	13.56	5.31
				Fast	8.20	1.77	-0.84	13.26	5.60
			90	Slow	9.26	2.79	-0.81	17.79	7.74
				Medium	9.33	2.36	-0.72	14.35	6.05
				Fast	11.03	2.37	-0.85	19.34	9.82
			135	Slow	10.33	2.65	-0.77	19.38	7.60
				Medium	11.87	2.99	-0.58	17.26	7.14
				Fast	12.62	3.21	-0.70	18.55	8.77
			180	Slow	7.99	3.00	-0.68	21.79	9.62
				Medium	8.34	2.55	-0.59	17.17	7.01
				Fast	8.80	2.26	-0.60	14.87	6.11
		Sagittal	Straight	Slow	10.96	1.35	-0.76	10.75	4.68
				Medium	11.51	1.18	-0.88	11.13	4.44
				Fast	10.38	1.47	-0.78	12.55	5.23
			45	Slow	4.99	2.64	-0.42	14.53	6.12
				Medium	3.87	2.56	-0.39	14.84	5.91
				Fast	5.50	2.67	-0.18	13.46	5.45
			90	Slow	2.62	3.49	-0.48	19.52	8.18
				Medium	3.00	2.41	-0.71	14.00	5.01
				Fast	3.44	3.54	-0.21	17.20	7.52
			135	Slow	3.97	2.98	-0.74	18.41	7.05
				Medium	4.28	3.04	-0.67	17.41	6.57
				Fast	3.97	2.62	-0.44	13.53	5.67
			180	Slow	8.62	2.90	-0.91	19.87	7.70
				Medium	5.25	3.19	-0.68	19.21	7.39
				Fast	5.73	3.59	-0.39	16.15	6.69
		Transverse	Straight	Slow	11.05	1.30	-0.74	10.25	4.44
				Medium	11.55	1.17	-0.86	11.04	4.42
				Fast	10.48	1.57	-0.72	12.54	5.21
			45	Slow	5.22	2.37	-0.63	15.43	6.39
				Medium	4.90	2.54	-0.30	14.40	5.71
				Fast	5.66	2.23	-0.39	13.93	5.39
			90	Slow	0.26	3.65	-0.30	18.33	7.59
				Medium	2.37	2.43	-0.49	13.16	4.83
				Fast	2.94	3.42	-0.09	15.04	6.67
			135	Slow	0.96	3.86	-0.38	19.49	7.20

				Medium	3.07	3.46	-0.58	18.71	6.94
				Fast	2.86	3.34	-0.24	14.20	6.31
			180	Slow	0.45	3.76	-0.41	18.54	7.46
				Medium	0.76	3.22	-0.70	20.07	7.54
				Fast	-0.32	3.45	-0.04	15.43	6.09
	Knee	Frontal	Straight	Slow	-4.07	0.73	0.00	4.06	1.46
				Medium	-3.76	0.83	-0.03	4.58	1.49
				Fast	-2.82	0.93	0.03	4.46	1.54
			45	Slow	-2.14	0.93	0.08	4.95	1.63
				Medium	-1.51	1.04	0.13	5.71	1.78
				Fast	-0.85	0.84	0.05	4.76	1.60
			90	Slow	1.42	1.36	0.18	6.54	2.32
				Medium	0.51	1.28	0.08	7.17	2.38
				Fast	0.31	1.00	0.07	5.85	2.16
			135	Slow	1.55	1.53	0.14	6.01	2.39
				Medium	1.45	1.27	0.09	5.36	2.10
				Fast	0.33	0.87	0.03	4.96	1.86
			180	Slow	2.31	1.91	0.15	7.79	3.33
				Medium	1.79	1.55	0.05	7.06	2.71
				Fast	0.92	1.56	-0.13	6.64	2.72
		Sagittal	Straight	Slow	-2.42	0.83	-0.24	4.88	1.76
				Medium	-1.29	1.08	-0.18	6.01	1.97
				Fast	0.45	0.93	-0.03	4.92	1.77
			45	Slow	-1.08	1.11	0.02	5.18	1.89
				Medium	-1.40	0.92	0.00	5.18	1.63
				Fast	-0.73	0.80	0.03	5.19	1.64
			90	Slow	0.57	1.21	0.08	5.86	2.01
				Medium	-0.05	1.22	0.02	6.81	2.30
				Fast	0.39	0.83	0.02	4.50	1.62
			135	Slow	1.40	1.32	0.15	5.72	2.17
				Medium	1.07	1.35	0.09	5.93	2.22
				Fast	0.27	0.80	0.00	4.88	1.81
			180	Slow	0.65	1.56	0.00	6.32	2.75
				Medium	0.86	1.40	0.06	6.12	2.47
				Fast	0.18	1.11	-0.05	5.87	2.14
		Transverse	Straight	Slow	-2.61	1.04	0.24	4.44	1.91
				Medium	-2.56	1.18	0.16	5.16	2.12

				Fast	-1.95	1.09	0.11	4.78	1.89
			45	Slow	0.18	1.06	0.11	4.43	1.76
				Medium	0.35	0.92	0.20	3.48	1.36
				Fast	0.69	0.85	0.17	3.82	1.47
			90	Slow	3.06	1.25	-0.11	5.53	2.28
				Medium	2.41	1.25	-0.02	5.54	2.20
				Fast	3.22	1.11	0.08	4.36	1.93
			135	Slow	3.82	1.24	-0.09	5.80	2.32
				Medium	3.63	1.10	-0.07	5.05	1.89
				Fast	2.71	0.95	0.06	3.76	1.61
			180	Slow	3.37	1.35	-0.08	5.76	2.50
				Medium	3.50	1.14	-0.11	5.58	2.20
				Fast	3.03	1.34	0.07	5.39	2.30
	Hip	Frontal	Straight	Slow	-0.85	1.29	0.24	5.51	2.16
				Medium	0.28	1.26	0.06	6.24	2.19
				Fast	0.94	1.16	0.00	5.71	2.18
			45	Slow	-0.71	1.15	0.00	5.25	1.95
				Medium	-0.86	1.02	0.06	4.93	1.68
				Fast	-0.50	0.88	0.10	5.37	1.71
			90	Slow	0.38	1.31	0.14	5.54	2.09
				Medium	-0.04	1.35	0.08	5.86	2.20
				Fast	1.28	0.99	0.14	4.40	1.66
			135	Slow	1.31	1.30	0.13	5.65	2.19
				Medium	1.19	1.33	0.07	6.02	2.34
				Fast	1.06	0.84	0.09	5.11	1.95
			180	Slow	1.37	1.88	0.10	7.30	3.24
				Medium	1.37	1.43	0.07	6.43	2.59
				Fast	0.59	1.08	0.00	5.76	1.99
		Sagittal	Straight	Slow	-0.86	0.87	-0.71	4.01	1.68
				Medium	-0.16	0.88	-0.29	5.18	1.74
				Fast	2.20	1.90	-0.37	10.42	3.72
			45	Slow	0.81	0.97	-0.63	6.49	2.82
				Medium	1.10	1.18	-0.45	7.04	2.69
				Fast	0.82	0.95	-0.74	7.91	3.12
			90	Slow	-1.81	1.19	-0.73	9.46	3.94
				Medium	-2.02	1.07	-0.88	9.02	3.56
				Fast	-2.17	1.35	-0.75	6.79	2.78

			135	Slow	-1.33	1.20	-0.53	7.91	3.30
				Medium	-1.59	1.09	-0.50	7.43	2.92
				Fast	-1.99	1.25	-0.61	7.98	3.84
			180	Slow	0.46	1.18	-0.62	8.68	3.64
				Medium	-0.23	1.15	-0.84	8.42	3.43
				Fast	-0.89	0.97	-0.68	6.30	2.57
		Transverse	Straight	Slow	-1.06	1.30	-0.95	9.02	3.86
				Medium	-0.94	1.10	-0.76	8.35	2.87
				Fast	-0.65	1.53	-0.77	9.13	3.47
			45	Slow	3.86	1.08	-0.88	10.28	4.54
				Medium	3.37	1.62	-0.56	9.82	3.87
				Fast	3.57	1.25	-0.73	11.32	4.70
			90	Slow	1.85	2.26	-0.41	11.29	4.68
				Medium	2.37	2.08	-0.68	12.44	4.94
				Fast	2.53	2.25	-0.48	10.80	4.92
			135	Slow	2.88	2.43	-0.45	13.96	5.82
				Medium	2.66	1.94	-0.62	12.39	4.79
				Fast	2.07	1.79	-0.44	9.64	4.00
			180	Slow	4.24	2.00	-0.58	14.02	6.53
				Medium	4.21	1.44	-0.67	10.14	4.04
				Fast	3.50	1.56	-0.59	9.95	4.25
Max	Ankle	Frontal	Straight	Slow	-0.87	1.37	-0.80	6.64	2.82
				Medium	-1.12	1.23	-0.69	7.65	2.45
				Fast	0.86	1.98	-0.62	11.80	4.28
			45	Slow	-0.70	1.30	-0.84	7.68	3.19
				Medium	-0.85	1.37	-0.62	7.18	2.61
				Fast	-2.13	1.02	-0.67	6.70	2.31
			90	Slow	-1.62	1.82	-0.53	7.67	3.21
				Medium	-2.22	1.56	-0.62	7.21	2.86
				Fast	-3.44	1.58	-0.72	6.70	2.63
			135	Slow	0.27	1.47	-0.36	7.54	2.83
				Medium	-0.81	1.57	-0.47	7.89	3.15
				Fast	-1.80	1.90	-0.57	8.91	3.65
			180	Slow	1.80	1.69	-0.66	10.18	4.05
				Medium	1.04	1.24	-0.83	7.41	2.71
				Fast	1.30	1.13	-0.58	6.71	2.70
		Sagittal	Straight	Slow	-3.51	1.03	-0.60	3.78	1.52

				Medium	-4.64	0.90	-0.44	5.22	1.69
				Fast	-4.36	1.38	-0.55	7.48	2.91
			45	Slow	-2.60	0.85	-0.47	4.60	1.84
				Medium	-2.28	0.80	-0.56	4.82	1.84
				Fast	-2.72	0.81	-0.69	4.93	1.79
			90	Slow	-1.96	1.22	-0.55	5.42	2.12
				Medium	-2.29	1.15	-0.65	5.76	2.21
				Fast	-3.10	1.14	-0.70	5.19	1.93
			135	Slow	-0.29	1.07	-0.27	4.96	1.97
				Medium	-1.06	0.92	-0.21	4.55	1.57
				Fast	-1.18	1.55	-0.42	6.77	2.66
			180	Slow	-0.81	0.94	-0.49	5.50	2.20
				Medium	-1.21	0.75	-0.47	4.60	1.55
				Fast	-1.27	0.80	-0.56	4.92	1.87
		Transverse	Straight	Slow	-8.31	1.17	-0.86	16.39	7.06
				Medium	-8.50	1.26	-0.63	15.46	5.46
				Fast	-9.11	1.42	-0.81	17.21	6.16
			45	Slow	-11.06	1.77	-0.75	18.67	7.91
				Medium	-7.18	1.62	-0.75	19.35	7.77
				Fast	-6.60	1.61	-0.82	20.97	9.13
			90	Slow	-10.26	2.31	-0.78	23.76	10.61
				Medium	-6.68	1.77	-0.83	18.27	7.56
				Fast	-6.10	2.26	-0.85	17.59	8.12
			135	Slow	-12.77	2.08	-0.71	22.93	9.54
				Medium	-11.20	1.74	-0.78	21.24	8.82
				Fast	-6.99	1.91	-0.76	21.57	10.71
			180	Slow	-15.56	2.11	-0.85	25.49	11.48
				Medium	-11.19	1.93	-0.72	18.54	7.15
				Fast	-11.45	2.21	-0.74	15.82	6.48
	Knee	Frontal	Straight	Slow	-2.21	1.57	-0.83	13.40	5.68
				Medium	-1.79	1.23	-0.69	13.57	4.71
				Fast	-2.66	1.47	-0.77	16.13	5.67
			45	Slow	-4.19	1.83	-0.85	14.93	6.43
				Medium	-4.04	1.42	-0.68	15.16	5.87
				Fast	-4.36	1.68	-0.75	15.04	6.25
			90	Slow	-2.76	2.43	-0.70	22.39	10.14
				Medium	-2.43	1.70	-0.84	15.92	6.52

				Fast	-2.28	1.94	-0.72	16.37	7.45
			135	Slow	-5.54	2.28	-0.57	19.95	8.54
				Medium	-6.60	2.15	-0.70	17.21	7.01
				Fast	-4.82	2.60	-0.51	16.32	7.50
			180	Slow	-6.11	1.98	-0.89	20.85	8.87
				Medium	-4.21	1.87	-0.65	18.06	7.16
				Fast	-4.64	2.28	-0.62	15.56	6.68
		Sagittal	Straight	Slow	-17.57	1.71	-0.76	15.25	6.67
				Medium	-17.63	1.53	-0.64	12.91	4.86
				Fast	-17.77	1.54	-0.68	15.83	5.74
			45	Slow	-16.52	1.93	-0.73	17.87	7.66
				Medium	-13.39	1.72	-0.66	15.92	6.43
				Fast	-13.62	1.77	-0.69	16.17	7.05
			90	Slow	-14.58	2.27	-0.75	17.86	7.99
				Medium	-11.34	1.55	-0.72	12.98	5.43
				Fast	-11.48	1.84	-0.68	16.51	7.76
			135	Slow	-14.34	1.38	-0.71	14.54	6.06
				Medium	-14.49	1.75	-0.81	15.44	6.43
				Fast	-13.72	1.90	-0.69	16.65	8.31
			180	Slow	-15.92	2.12	-0.86	18.91	8.20
				Medium	-12.13	1.94	-0.61	13.86	5.46
				Fast	-12.42	1.99	-0.58	12.52	5.05
		Transverse	Straight	Slow	-1.69	1.83	-0.72	13.97	5.84
				Medium	-0.80	1.21	-0.61	12.52	4.30
				Fast	-2.33	1.58	-0.75	16.37	5.77
			45	Slow	-4.42	1.93	-0.91	15.31	6.61
				Medium	-4.26	1.42	-0.68	15.21	5.85
				Fast	-4.95	1.79	-0.83	15.05	6.22
			90	Slow	-2.76	2.42	-0.70	22.52	10.19
				Medium	-3.62	1.66	-0.83	15.06	6.04
				Fast	-3.54	1.97	-0.55	15.62	7.02
			135	Slow	-5.68	2.39	-0.54	19.77	8.48
				Medium	-7.37	2.22	-0.81	17.80	7.10
				Fast	-6.31	2.41	-0.39	15.08	7.00
			180	Slow	-5.99	2.06	-0.86	20.50	8.69
				Medium	-4.37	1.83	-0.68	18.46	7.30
				Fast	-4.98	2.28	-0.63	16.39	6.90

	Hip	Frontal	Straight	Slow	4.66	1.64	-0.09	7.31	3.11
				Medium	4.85	1.80	-0.18	8.98	3.61
				Fast	6.37	1.73	-0.28	9.52	3.59
			45	Slow	6.31	1.81	-0.18	9.48	3.83
				Medium	6.75	1.63	-0.31	10.19	3.69
				Fast	6.17	1.58	-0.35	8.68	3.53
			90	Slow	10.87	2.51	-0.34	13.00	5.11
				Medium	7.58	1.33	-0.47	11.42	4.19
				Fast	10.01	2.26	-0.48	10.76	4.88
			135	Slow	12.75	2.77	-0.12	12.86	5.14
				Medium	13.35	2.43	-0.34	12.68	5.17
				Fast	12.14	1.70	-0.17	7.86	3.06
			180	Slow	14.99	3.03	-0.20	13.31	5.76
				Medium	16.52	2.57	-0.32	12.26	4.94
				Fast	15.23	1.86	-0.40	9.39	3.97
		Sagittal	Straight	Slow	5.95	1.57	-0.56	8.61	3.56
				Medium	7.24	1.65	-0.52	10.83	4.23
				Fast	8.38	2.05	-0.28	10.36	4.22
			45	Slow	8.02	2.27	-0.20	10.04	4.33
				Medium	8.10	1.75	-0.21	8.49	3.13
				Fast	8.24	2.08	-0.16	9.92	3.81
			90	Slow	7.68	2.47	-0.10	10.09	4.17
				Medium	6.16	1.77	-0.08	8.67	3.13
				Fast	6.98	1.47	0.00	7.49	2.75
			135	Slow	8.70	3.10	0.11	12.95	5.05
				Medium	8.22	2.44	0.01	11.20	4.23
				Fast	5.69	2.16	0.04	9.75	3.76
			180	Slow	10.00	3.96	0.03	15.78	6.90
				Medium	10.36	3.14	0.07	14.35	5.57
				Fast	9.68	2.91	0.02	12.44	5.03
		Transverse	Straight	Slow	6.06	1.85	-0.17	8.29	3.61
				Medium	4.94	1.80	-0.22	9.04	3.69
				Fast	6.36	1.73	-0.28	9.52	3.59
			45	Slow	6.83	1.86	-0.18	9.39	3.92
				Medium	6.72	1.78	-0.27	10.44	3.96
				Fast	6.17	1.63	-0.35	8.70	3.54
			90	Slow	12.04	2.43	-0.30	12.46	5.12

				Medium	8.67	2.05	-0.35	12.82	5.04
				Fast	11.69	2.10	-0.35	10.67	4.88
			135	Slow	13.25	2.64	-0.33	14.26	6.04
				Medium	13.59	2.30	-0.36	12.68	5.23
				Fast	11.02	1.74	-0.28	10.10	4.40
			180	Slow	14.81	2.57	-0.37	15.47	7.03
				Medium	14.78	2.23	-0.42	15.11	6.08
				Fast	13.07	2.39	-0.37	14.58	6.31
TO	Ankle	Frontal	Straight	Slow	5.95	1.57	-0.56	8.61	3.56
				Medium	7.24	1.65	-0.52	10.83	4.23
				Fast	8.38	2.05	-0.28	10.36	4.22
			45	Slow	8.02	2.27	-0.20	10.04	4.33
				Medium	8.10	1.76	-0.21	8.50	3.13
				Fast	8.24	2.08	-0.16	9.92	3.81
			90	Slow	7.66	2.47	-0.07	10.18	4.16
				Medium	6.11	1.77	-0.08	8.73	3.15
				Fast	7.03	1.42	0.01	7.43	2.70
			135	Slow	8.50	3.17	0.14	12.87	5.13
				Medium	8.03	2.53	0.01	11.46	4.38
				Fast	5.61	2.32	0.09	9.40	3.77
			180	Slow	9.88	4.10	0.03	16.30	7.15
				Medium	10.27	3.19	0.07	14.43	5.66
				Fast	9.23	3.06	0.00	12.99	5.37
		Sagittal	Straight	Slow	-0.52	1.25	-0.39	6.07	2.69
				Medium	0.87	1.33	-0.48	6.71	2.72
				Fast	1.66	1.42	-0.43	8.05	3.22
			45	Slow	-0.90	1.29	-0.26	7.92	3.16
				Medium	-1.28	1.11	-0.28	6.94	2.49
				Fast	-1.92	1.74	-0.36	8.95	3.71
			90	Slow	-1.20	1.58	-0.37	9.08	3.55
				Medium	-0.08	1.60	-0.37	11.46	4.08
				Fast	-1.80	2.60	-0.27	13.29	5.51
			135	Slow	-0.43	0.91	-0.48	8.59	3.31
				Medium	0.09	0.79	-0.57	8.32	2.94
				Fast	-1.11	1.49	-0.43	9.95	3.96
			180	Slow	0.24	1.02	-0.47	8.70	3.38
				Medium	1.84	0.86	-0.38	6.52	2.35

				Fast	1.25	0.88	-0.44	6.26	2.28
		Transverse	Straight	Slow	2.54	0.85	-0.07	3.79	1.46
				Medium	1.90	0.77	-0.04	3.80	1.34
				Fast	1.52	0.82	0.13	4.08	1.47
			45	Slow	0.18	1.10	-0.10	5.37	2.00
				Medium	-1.49	1.42	-0.21	7.78	2.98
				Fast	-1.64	1.30	-0.29	7.30	2.80
			90	Slow	-0.45	0.99	-0.47	8.64	3.42
				Medium	0.35	0.79	-0.33	7.84	2.53
				Fast	-0.88	2.06	-0.23	12.51	5.07
			135	Slow	1.61	0.75	-0.55	9.44	3.91
				Medium	2.45	1.10	-0.48	9.07	3.46
				Fast	0.26	1.51	-0.24	8.27	3.38
			180	Slow	2.52	1.25	-0.40	8.64	3.75
				Medium	3.37	1.04	-0.32	7.83	2.76
				Fast	4.04	1.19	-0.28	7.17	2.93
	Knee	Frontal	Straight	Slow	-2.02	0.96	-0.35	4.73	2.06
				Medium	-1.69	0.89	-0.37	5.11	2.05
				Fast	-0.55	0.98	-0.36	6.01	2.30
			45	Slow	-1.40	0.95	-0.36	7.37	2.92
				Medium	-2.14	1.02	-0.45	7.40	2.63
				Fast	-1.89	0.88	-0.35	6.84	2.81
			90	Slow	0.33	1.08	-0.20	6.66	2.40
				Medium	-0.30	0.63	-0.29	7.29	2.43
				Fast	-0.73	0.81	-0.24	7.52	2.61
			135	Slow	1.90	1.02	-0.31	7.54	2.70
				Medium	2.35	0.85	-0.37	8.54	2.77
				Fast	0.18	1.29	-0.27	6.11	2.38
			180	Slow	1.74	1.15	-0.42	9.18	3.68
				Medium	2.59	1.00	-0.36	7.35	2.68
				Fast	2.53	1.22	-0.40	7.26	2.73
		Sagittal	Straight	Slow	2.74	0.85	0.09	3.74	1.47
				Medium	1.97	0.79	-0.06	3.88	1.35
				Fast	1.66	0.77	0.19	4.08	1.42
			45	Slow	0.97	1.01	-0.04	5.18	1.76
				Medium	-0.64	1.23	-0.12	6.80	2.32
				Fast	-0.95	1.40	-0.24	6.45	2.52

			90	Slow	0.61	1.08	-0.16	6.30	2.29
				Medium	0.12	1.05	-0.19	6.42	2.12
				Fast	-0.60	0.93	-0.25	8.88	3.10
			135	Slow	2.65	0.98	-0.24	6.24	2.27
				Medium	3.21	0.98	-0.28	7.74	2.87
				Fast	0.81	1.25	-0.28	6.98	2.77
			180	Slow	2.44	1.54	-0.26	8.35	3.41
				Medium	2.84	1.17	-0.11	6.14	2.19
				Fast	3.65	1.16	-0.15	6.46	2.38
		Transverse	Straight	Slow	6.20	1.11	-0.97	16.54	7.31
				Medium	9.83	1.00	-0.95	14.75	5.72
				Fast	11.96	0.86	-0.99	15.81	6.19
			45	Slow	9.79	1.43	-0.74	13.97	6.07
				Medium	9.19	1.43	-0.80	15.38	6.17
				Fast	6.19	1.57	-0.81	15.68	6.52
			90	Slow	7.71	1.85	-0.85	17.95	7.96
				Medium	4.32	1.49	-0.88	18.08	7.95
				Fast	4.50	2.48	-0.94	19.01	9.21
			135	Slow	8.57	1.61	-0.89	22.01	9.69
				Medium	6.76	1.65	-0.77	18.15	7.75
				Fast	4.43	2.52	-0.88	18.01	9.07
			180	Slow	9.37	1.59	-0.81	25.92	12.48
				Medium	8.20	1.59	-0.82	21.43	8.96
				Fast	7.16	2.02	-0.79	21.29	9.74
	Hip	Frontal	Straight	Slow	3.64	1.12	-0.96	15.68	6.92
				Medium	6.91	1.01	-0.94	14.53	5.43
				Fast	10.98	0.74	-1.00	15.87	6.44
			45	Slow	10.46	1.22	-0.96	14.50	6.41
				Medium	9.03	1.09	-0.91	16.16	6.58
				Fast	8.58	1.25	-0.86	16.79	7.34
			90	Slow	10.73	1.18	-0.76	14.98	6.49
				Medium	8.14	1.12	-0.87	15.49	6.51
				Fast	9.00	1.74	-0.72	13.16	6.02
			135	Slow	10.82	2.10	-0.58	15.56	6.33
				Medium	10.65	2.08	-0.82	14.67	5.61
				Fast	9.44	1.63	-0.83	12.51	5.84
			180	Slow	15.26	2.02	-0.58	15.39	6.83

				Medium	15.15	1.57	-0.70	13.13	4.89
				Fast	16.14	2.00	-0.77	16.96	7.42
		Sagittal	Straight	Slow	-0.15	1.09	-0.95	14.84	6.57
				Medium	0.37	1.02	-1.00	14.77	5.79
				Fast	1.62	0.99	-1.02	17.30	7.15
			45	Slow	5.94	1.46	-0.68	12.73	5.57
				Medium	5.27	1.29	-0.85	13.80	5.54
				Fast	3.48	1.94	-0.66	14.71	6.45
			90	Slow	4.32	1.91	-0.76	16.95	7.63
				Medium	-0.48	1.42	-0.82	13.86	5.73
				Fast	0.01	2.65	-0.94	18.14	9.21
			135	Slow	4.72	1.72	-0.86	15.86	6.77
				Medium	2.63	1.52	-0.76	13.24	5.38
				Fast	1.21	2.42	-0.84	14.43	7.16
			180	Slow	4.71	1.73	-0.77	24.99	12.02
				Medium	4.81	1.37	-0.77	19.80	8.28
				Fast	3.21	1.97	-0.79	19.44	8.83
		Transverse	Straight	Slow	-2.88	1.23	-0.84	13.77	5.88
				Medium	-2.95	1.27	-0.87	14.38	5.28
				Fast	1.59	1.40	-1.12	19.81	8.26
			45	Slow	3.22	1.08	-0.88	15.17	6.58
				Medium	1.06	1.12	-0.96	17.48	6.97
				Fast	0.43	1.21	-0.88	15.07	6.48
			90	Slow	3.04	1.27	-0.75	15.41	6.61
				Medium	0.70	1.19	-0.92	12.82	5.05
				Fast	-0.83	2.07	-0.72	12.34	5.50
			135	Slow	7.13	1.96	-0.56	14.82	6.18
				Medium	5.99	1.91	-0.67	13.78	5.47
				Fast	2.36	1.36	-0.70	10.54	4.80
			180	Slow	9.63	1.79	-0.63	17.50	7.78
				Medium	10.49	1.84	-0.68	14.37	5.37
				Fast	10.36	1.65	-0.79	19.88	8.41

Abbreviations: CI = Confidence Interval, LoA = Limit of Agreement, Lower = Lower Limit, Upper = Upper Limit, TD = Touchdown, TO = Toe-off.

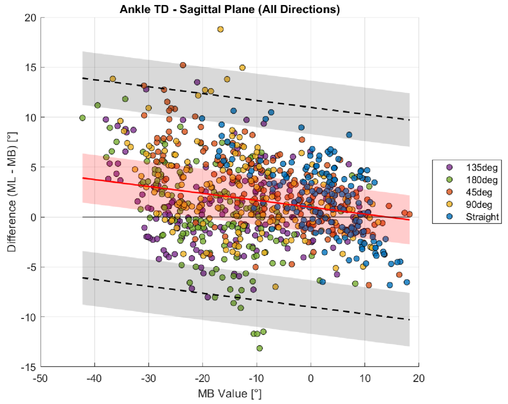

Supplementary Figure 1: Extended BA Plot for TD ankle angle - Sagittal Plane

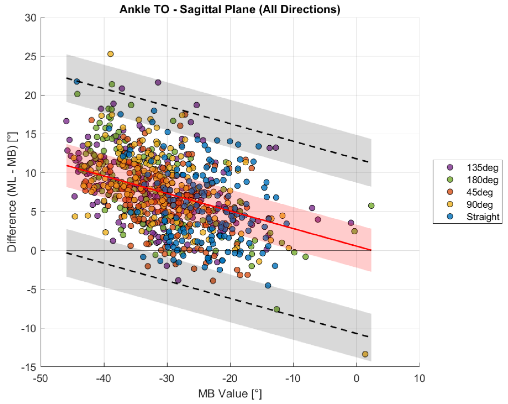

Supplementary Figure 2: Extended BA Plot for TO ankle angle - Sagittal Plane

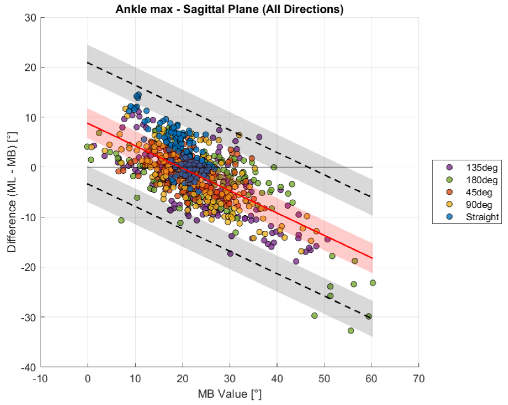

Supplementary Figure 3: Extended BA Plot for maximal ankle angle - Sagittal Plane

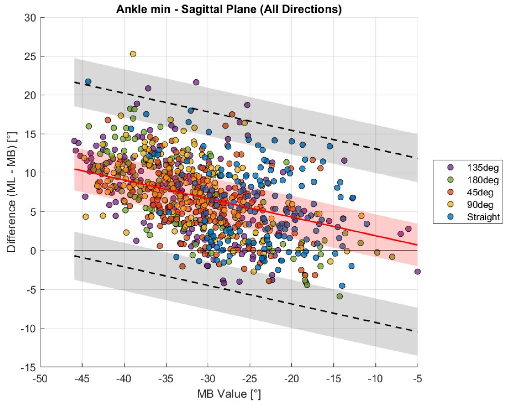

Supplementary Figure 4: Extended BA Plot for minimal ankle angle - Sagittal Plane

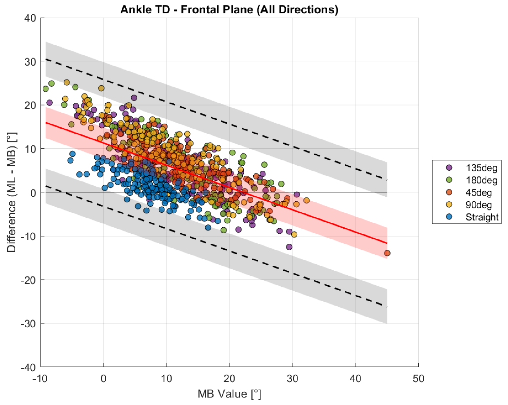

Supplementary Figure 5: Extended BA Plot for TD ankle angle - Frontal Plane

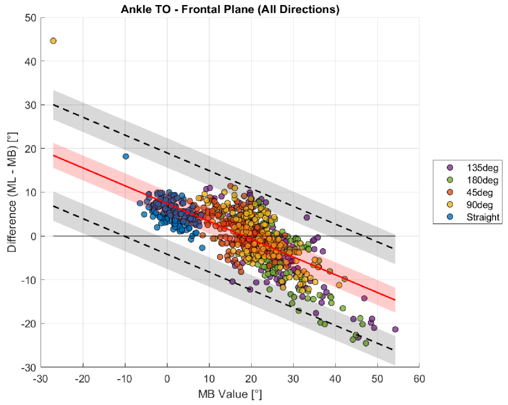

Supplementary Figure 6: Extended BA Plot for TO ankle angle - Frontal Plane

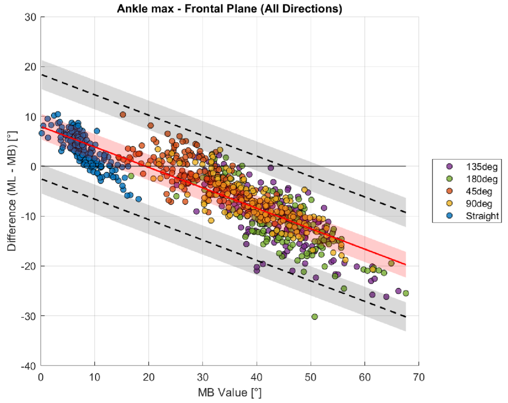

Supplementary Figure 7: Extended BA Plot for maximal ankle angle - Frontal Plane

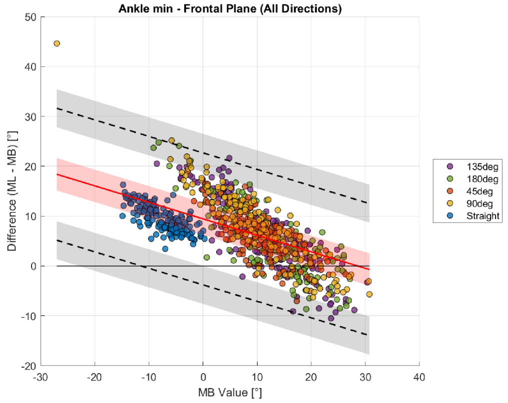

Supplementary Figure 8: Extended BA Plot for minimal ankle angle - Frontal Plane

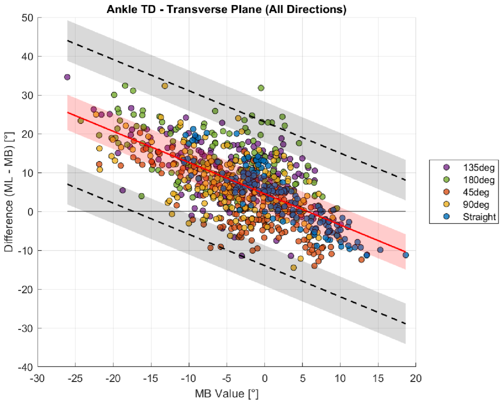

Supplementary Figure 9: Extended BA Plot for TD ankle angle - Transverse Plane

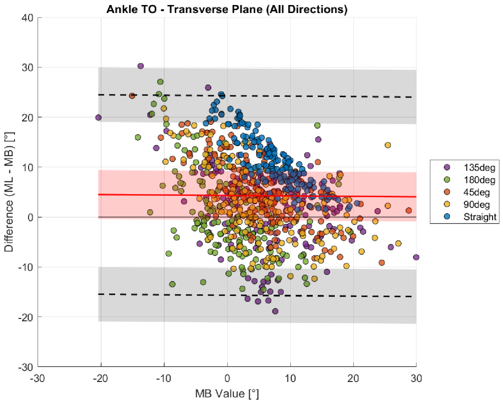

Supplementary Figure 10: Extended BA Plot for TO ankle angle - Transverse Plane

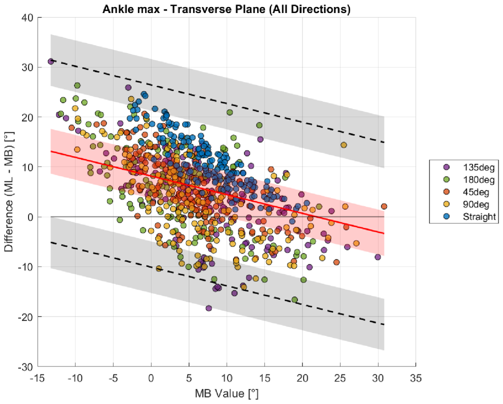

Supplementary Figure 11: Extended BA Plot for maximal ankle angle - Transverse Plane

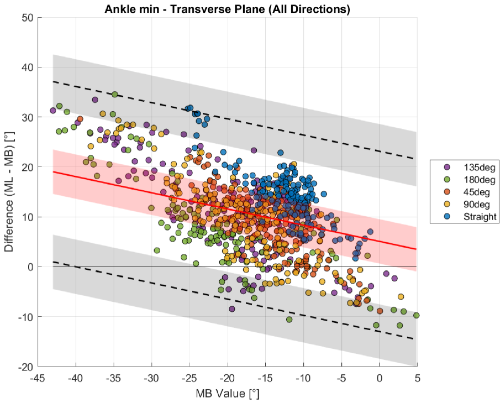

Supplementary Figure 12: Extended BA Plot for minimal ankle angle - Transverse Plane

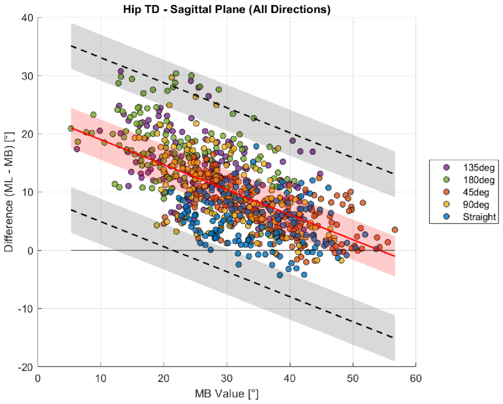

Supplementary Figure 13: Extended BA Plot for TD hip angle - Sagittal Plane

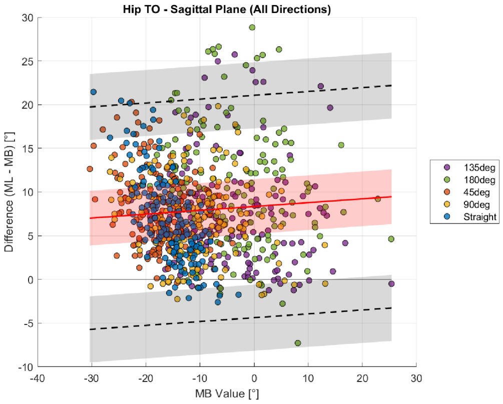

Supplementary Figure 14: Extended BA Plot for TO hip angle - Sagittal Plane

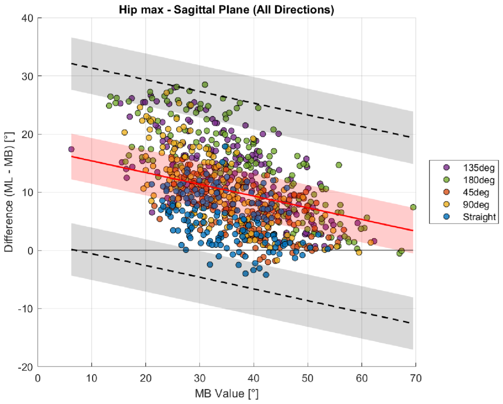

Supplementary Figure 15: Extended BA Plot for maximal hip angle - Sagittal Plane

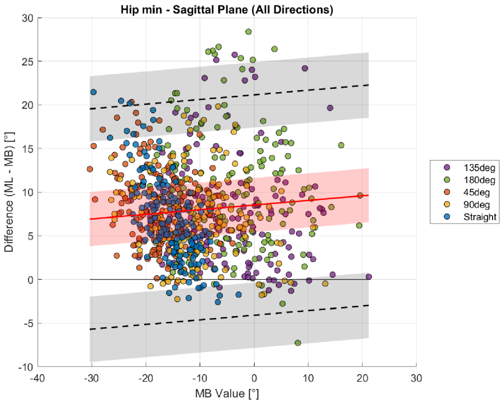

Supplementary Figure 16: Extended BA Plot for minimal hip angle - Sagittal Plane

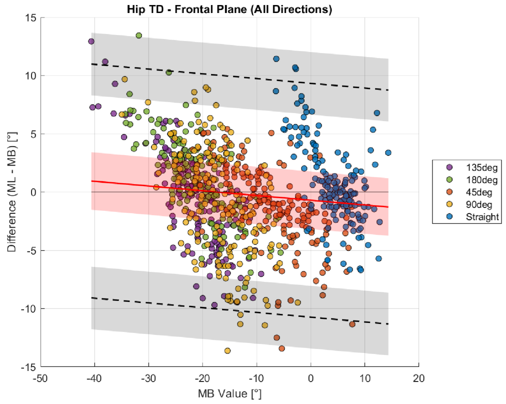

Supplementary Figure 17: Extended BA Plot for TD hip angle - Frontal Plane

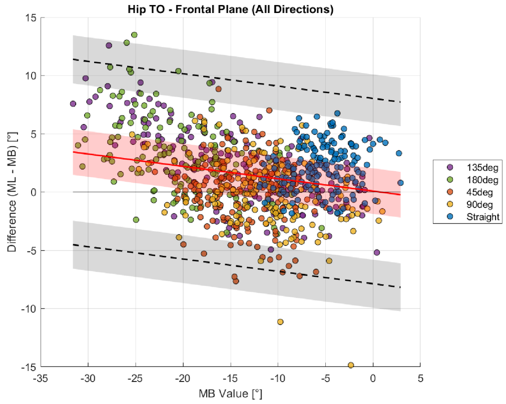

Supplementary Figure 18: Extended BA Plot for TO hip angle - Frontal Plane

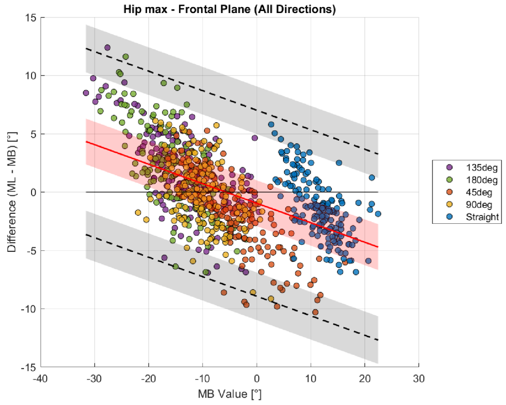

Supplementary Figure 19: Extended BA Plot for maximal hip angle - Frontal Plane

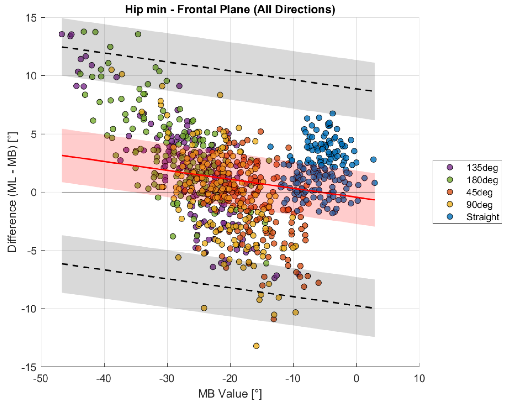

Supplementary Figure 20: Extended BA Plot for minimal hip angle - Frontal Plane

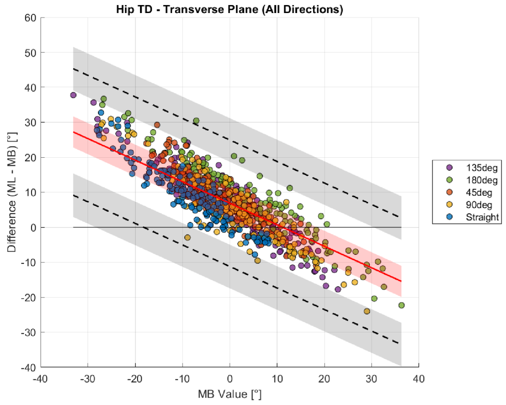

Supplementary Figure 21: Extended BA Plot for TD hip angle - Transverse Plane

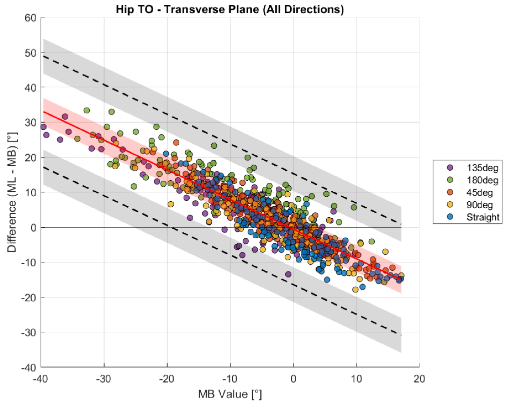

Supplementary Figure 22: Extended BA Plot for TO hip angle - Transverse Plane

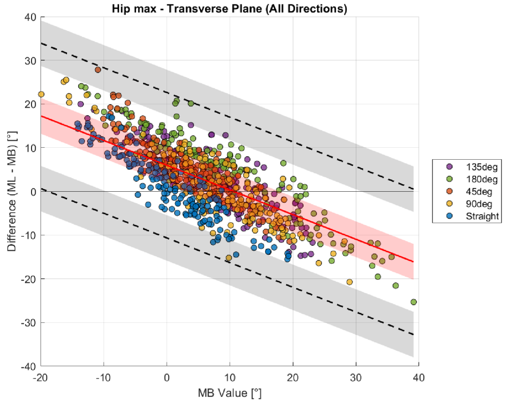

Supplementary Figure 23: Extended BA Plot for maximal hip angle - Transverse Plane

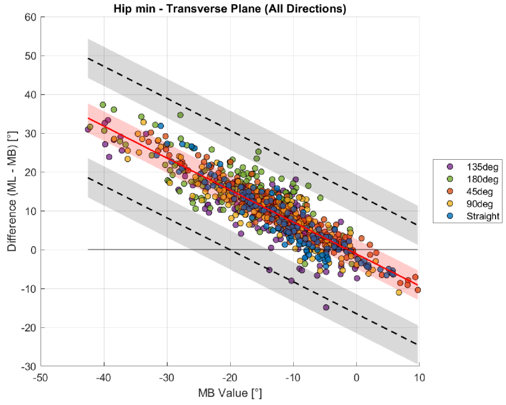

Supplementary Figure 24: Extended BA Plot for minimal hip angle - Transverse Plane

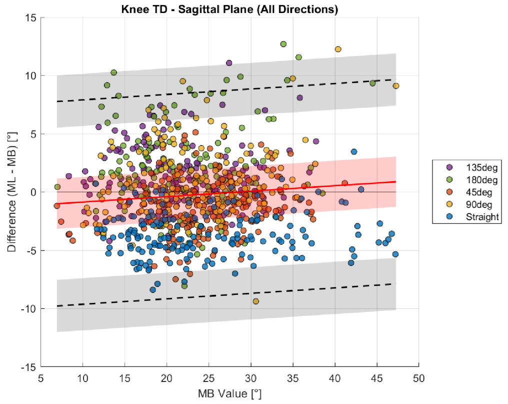

Supplementary Figure 25: Extended BA Plot for TD knee angle - Sagittal Plane

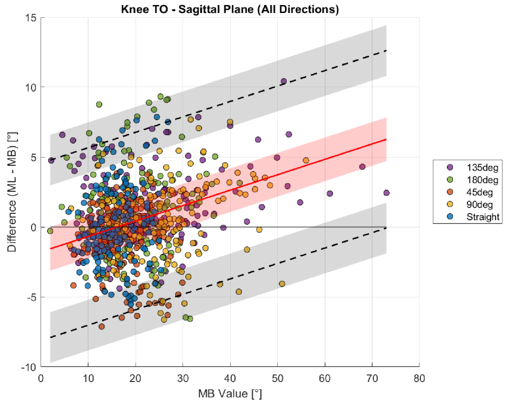

Supplementary Figure 26: Extended BA Plot for TO knee angle - Sagittal Plane

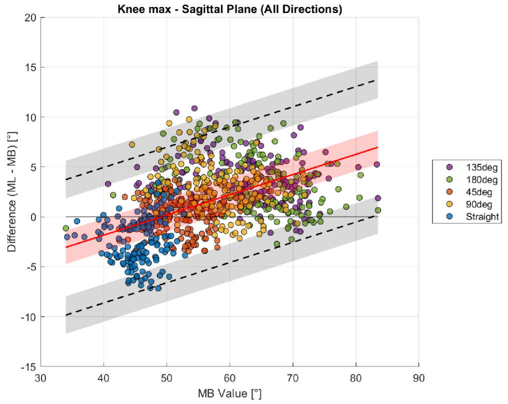

Supplementary Figure 27: Extended BA Plot for maximal knee angle - Sagittal Plane

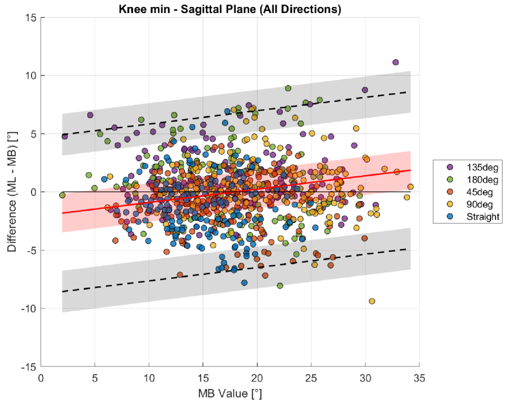

Supplementary Figure 28: Extended BA Plot for minimal knee angle - Sagittal Plane

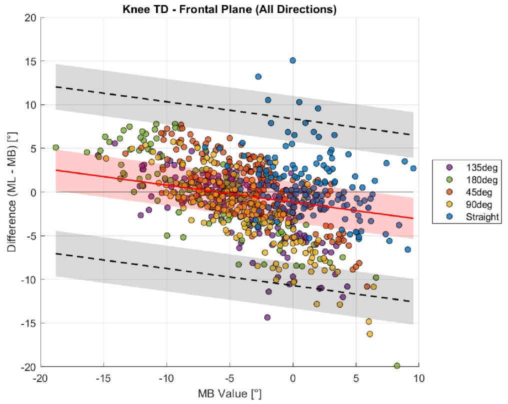

Supplementary Figure 29: Extended BA Plot for TD knee angle - Frontal Plane

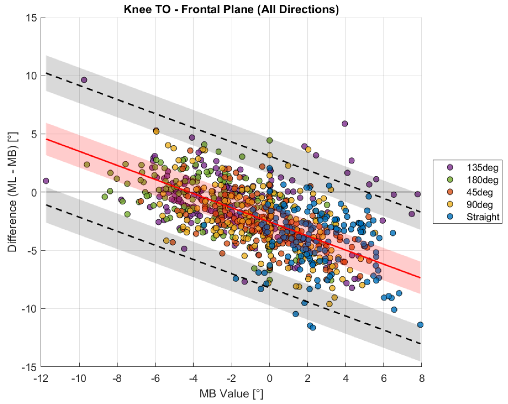

Supplementary Figure 30: Extended BA Plot for TO knee angle - Frontal Plane

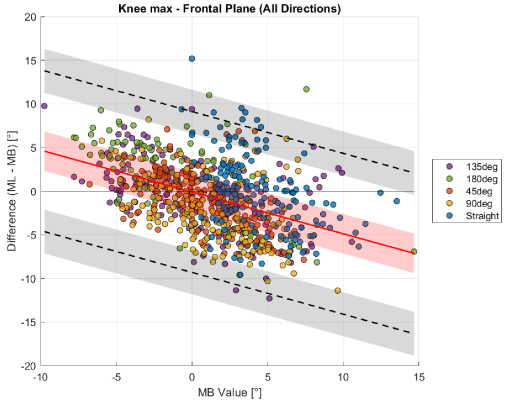

Supplementary Figure 31: Extended BA Plot for maximal knee angle - Frontal Plane

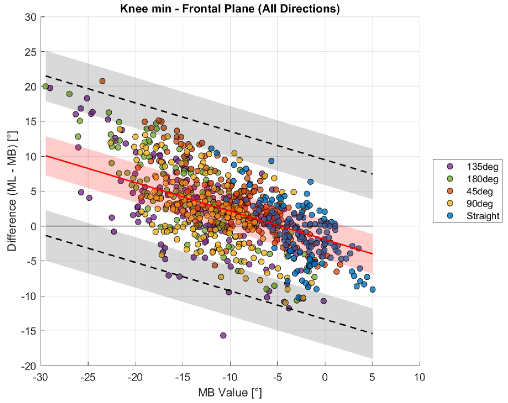

Supplementary Figure 32: Extended BA Plot for minimal knee angle - Frontal Plane

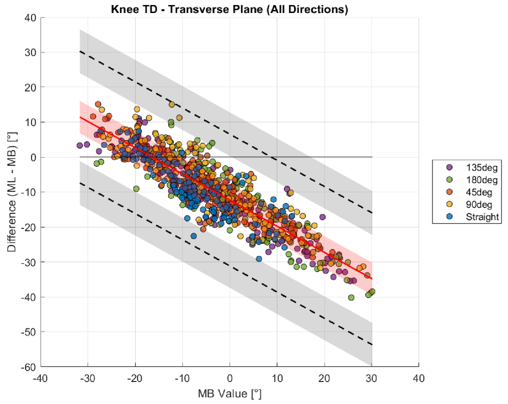

Supplementary Figure 33: Extended BA Plot for TD knee angle - Transverse Plane

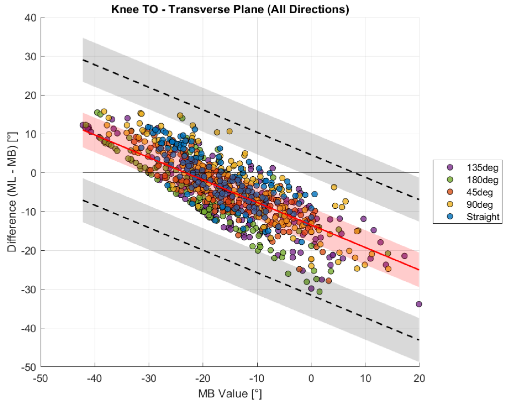

Supplementary Figure 34: Extended BA Plot for TO knee angle - Transverse Plane

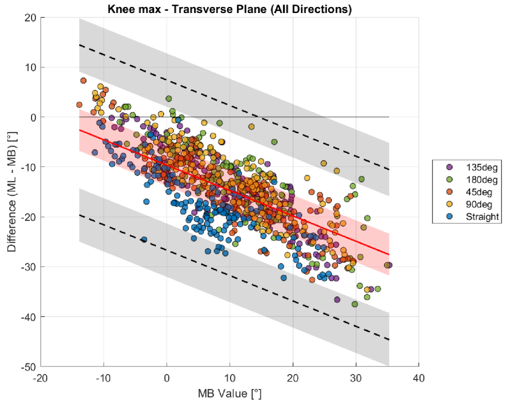

Supplementary Figure 35: Extended BA Plot for maximal knee angle - Transverse Plane

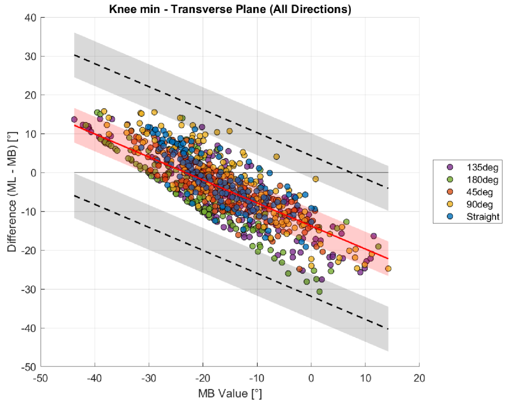

Supplementary Figure 36: Extended BA Plot for minimal knee angle - Transverse Plane

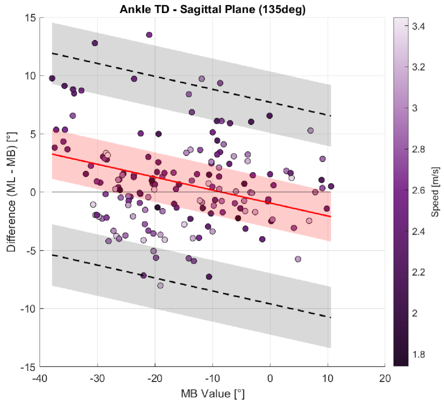

Supplementary Figure 37: Extended BA Plot for TD ankle angle - Sagittal Plane (135deg)

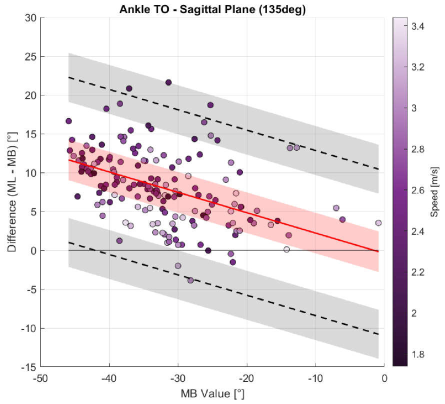

Supplementary Figure 38: Extended BA Plot for TO ankle angle - Sagittal Plane (135deg)

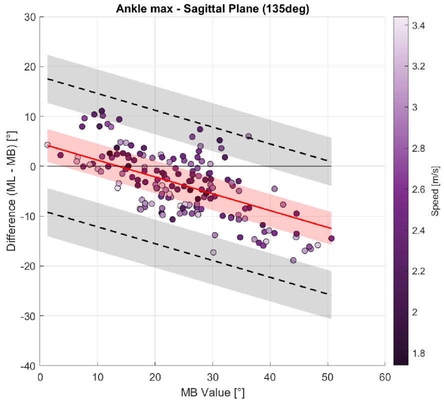

Supplementary Figure 39: Extended BA Plot for maximal ankle angle - Sagittal Plane (135deg)

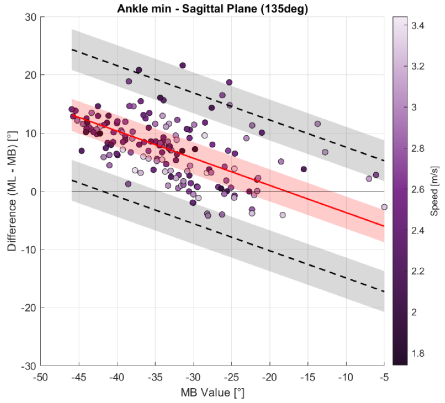

Supplementary Figure 40: Extended BA Plot for minimal ankle angle - Sagittal Plane (135deg)

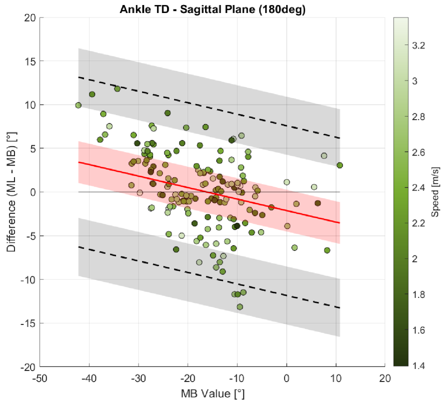

Supplementary Figure 41: Extended BA Plot for TD ankle angle - Sagittal Plane (180deg)

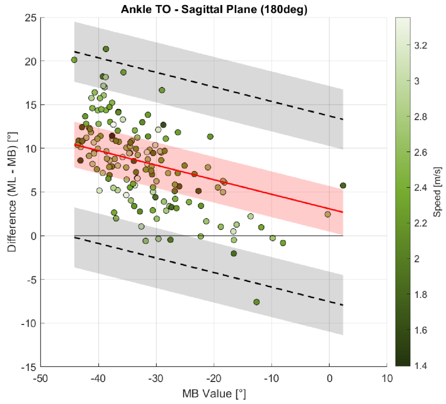

Supplementary Figure 42: Extended BA Plot for TO ankle angle - Sagittal Plane (180deg)

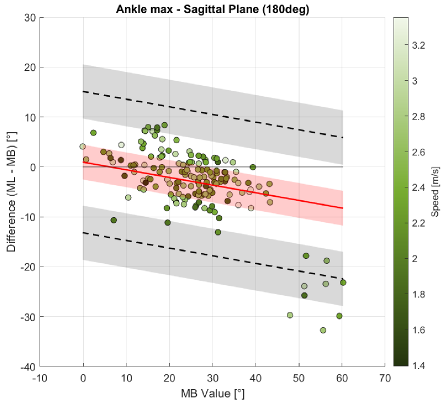

Supplementary Figure 43: Extended BA Plot for maximal ankle angle - Sagittal Plane (180deg)

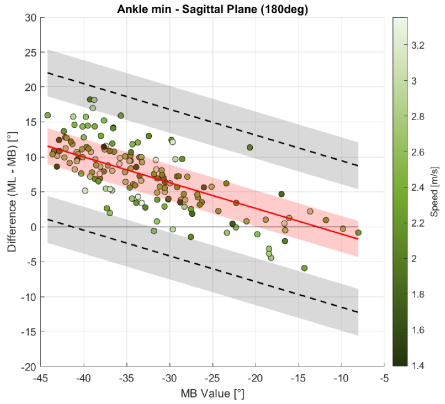

Supplementary Figure 44: Extended BA Plot for minimal ankle angle - Sagittal Plane (180deg)

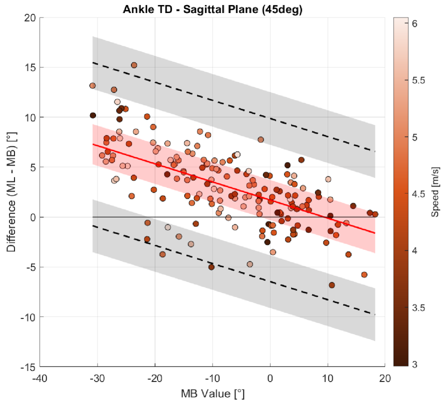

Supplementary Figure 45: Extended BA Plot for TD ankle angle - Sagittal Plane (45deg)

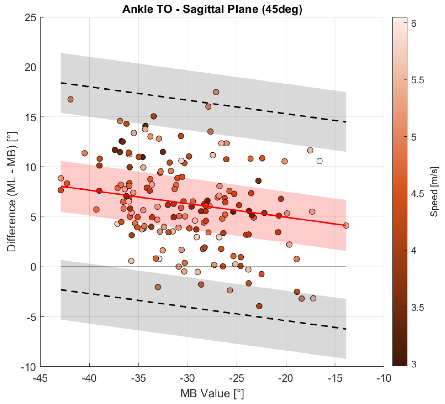

Supplementary Figure 46: Extended BA Plot for TO ankle angle - Sagittal Plane (45deg)

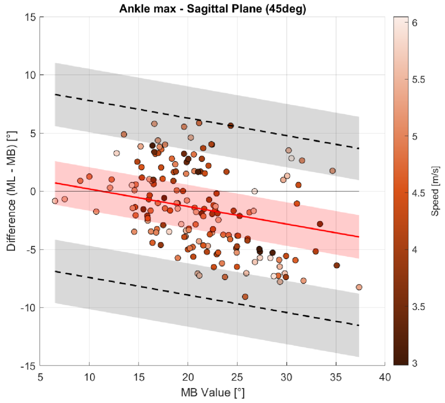

Supplementary Figure 47: Extended BA Plot for maximal ankle angle - Sagittal Plane (45deg)

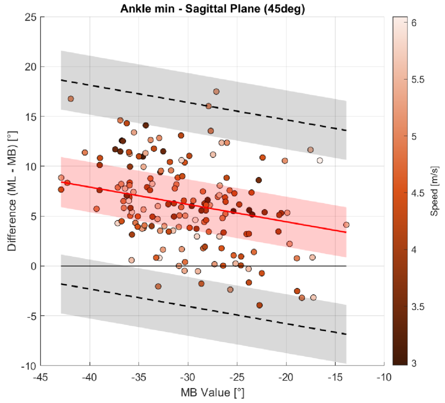

Supplementary Figure 48: Extended BA Plot for minimal ankle angle - Sagittal Plane (45deg)

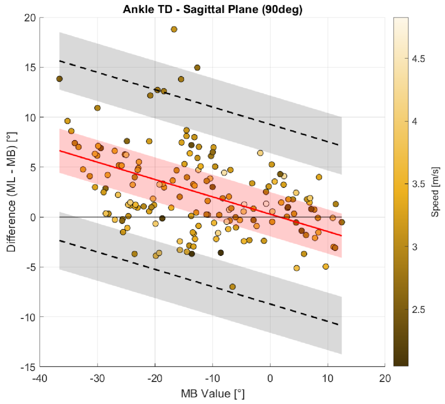

Supplementary Figure 49: Extended BA Plot for TD ankle angle - Sagittal Plane (90deg)

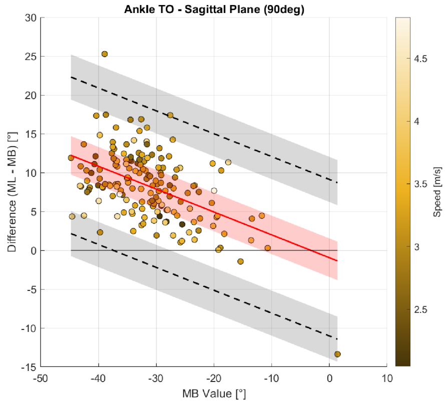

Supplementary Figure 50: Extended BA Plot for TO ankle angle - Sagittal Plane (90deg)

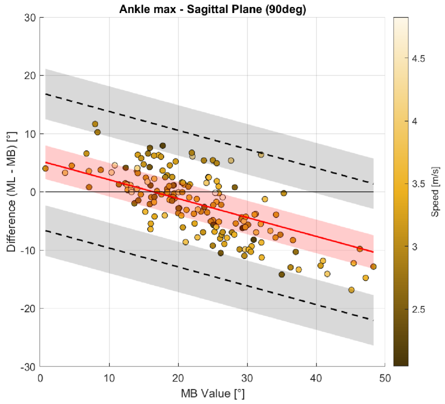

Supplementary Figure 51: Extended BA Plot for maximal ankle angle - Sagittal Plane (90deg)

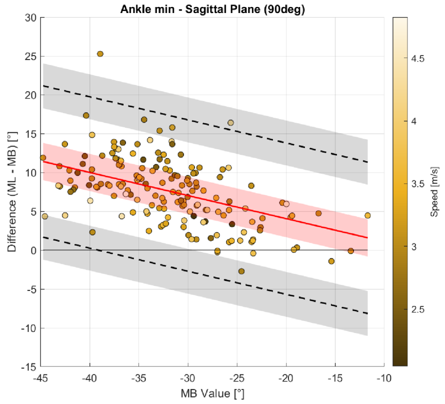

Supplementary Figure 52: Extended BA Plot for minimal ankle angle - Sagittal Plane (90deg)

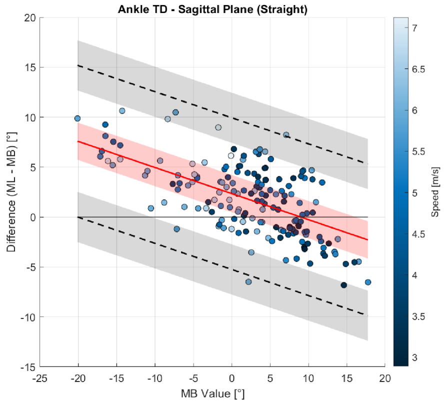

Supplementary Figure 53: Extended BA Plot for TD ankle angle - Sagittal Plane (Straight)

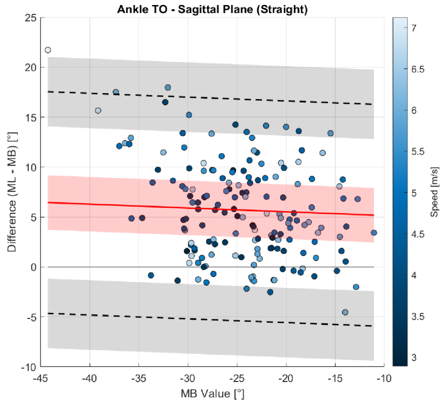

Supplementary Figure 54: Extended BA Plot for TO ankle angle - Sagittal Plane (Straight)

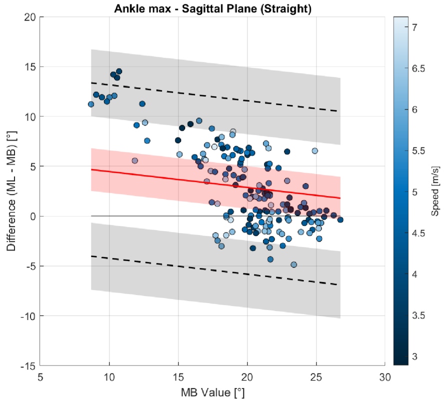

Supplementary Figure 55: Extended BA Plot for maximal ankle angle - Sagittal Plane (Straight)

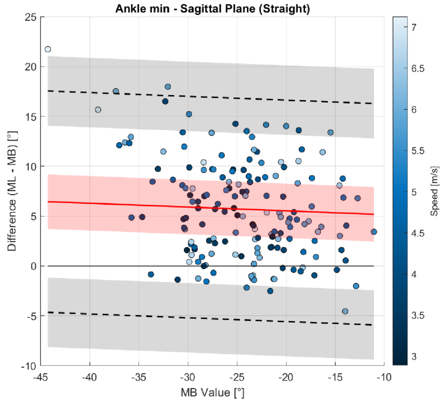

Supplementary Figure 56: Extended BA Plot for minimal ankle angle - Sagittal Plane (Straight)

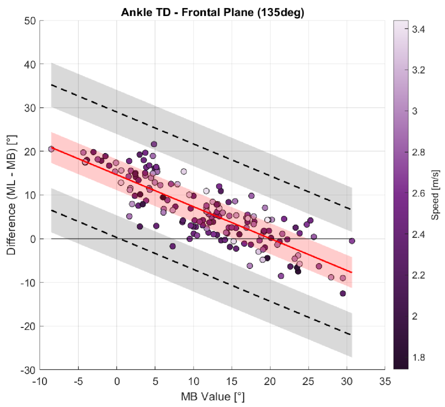

Supplementary Figure 57: Extended BA Plot for TD ankle angle - Frontal Plane (135deg)

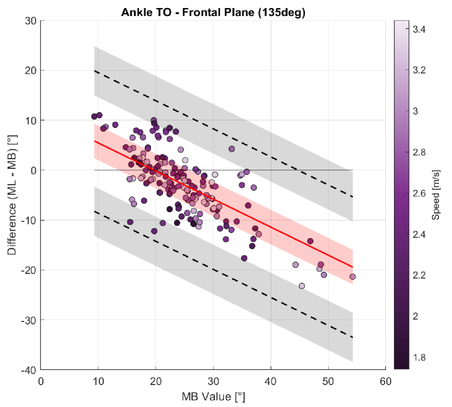

Supplementary Figure 58: Extended BA Plot for TO ankle angle - Frontal Plane (135deg)

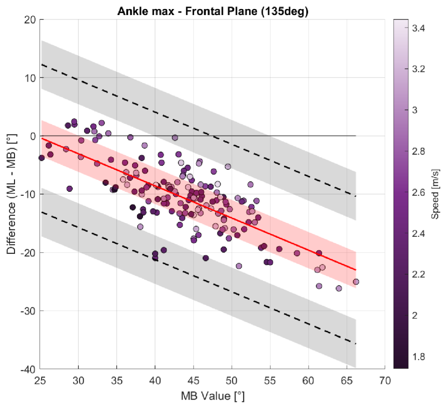

Supplementary Figure 59: Extended BA Plot for maximal ankle angle - Frontal Plane (135deg)

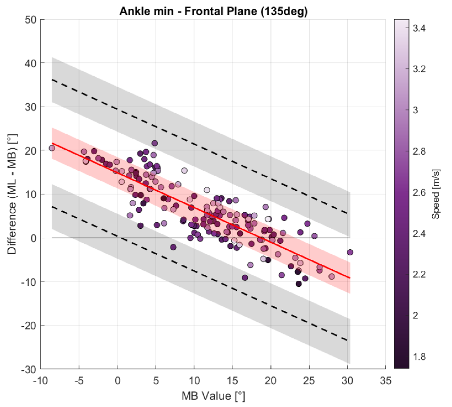

Supplementary Figure 60: Extended BA Plot for minimal ankle angle - Frontal Plane (135deg)

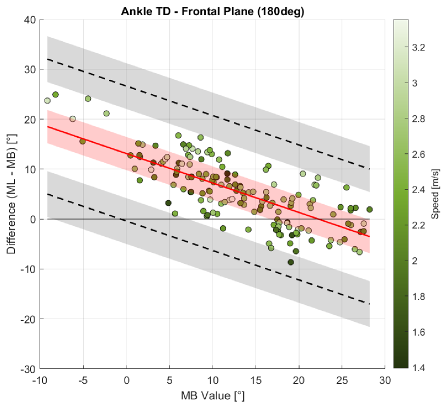

Supplementary Figure 61: Extended BA Plot for TD ankle angle - Frontal Plane (180deg)

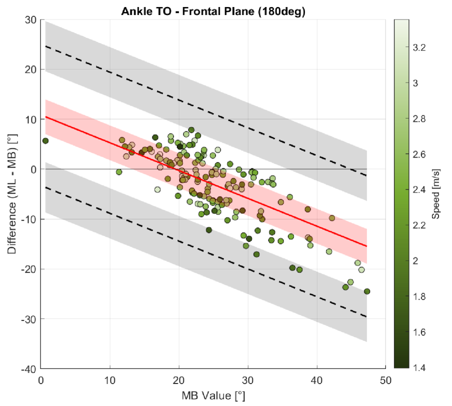

Supplementary Figure 62: Extended BA Plot for TO ankle angle - Frontal Plane (180deg)

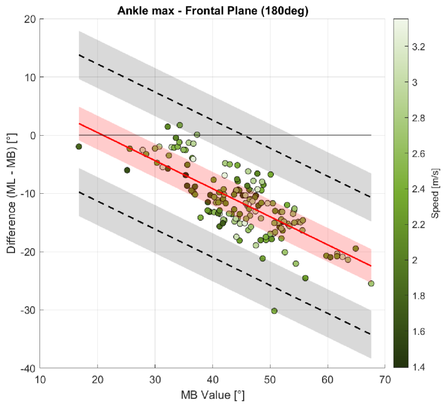

Supplementary Figure 63: Extended BA Plot for maximal ankle angle - Frontal Plane (180deg)

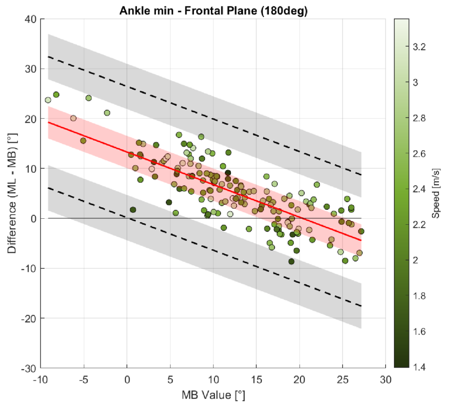

Supplementary Figure 64: Extended BA Plot for minimal ankle angle - Frontal Plane (180deg)

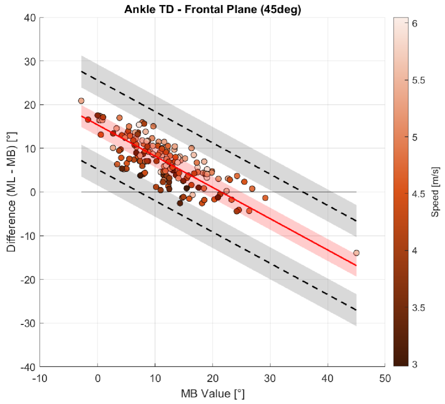

Supplementary Figure 65: Extended BA Plot for TD ankle angle - Frontal Plane (45deg)

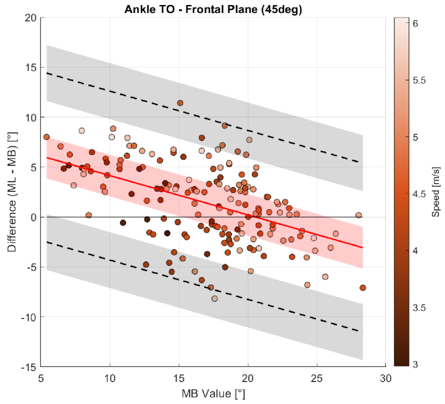

Supplementary Figure 66: Extended BA Plot for TO ankle angle - Frontal Plane (45deg)

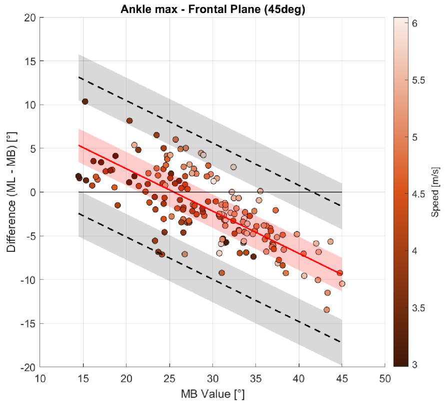

Supplementary Figure 67: Extended BA Plot for maximal ankle angle - Frontal Plane (45deg)

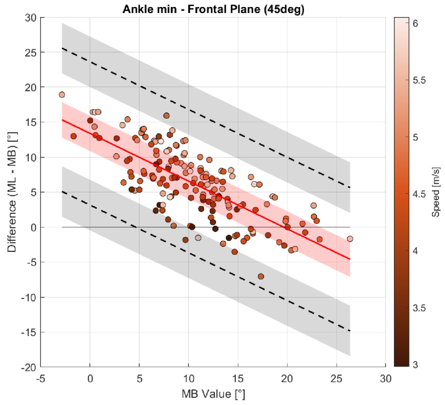

Supplementary Figure 68: Extended BA Plot for minimal ankle angle - Frontal Plane (45deg)

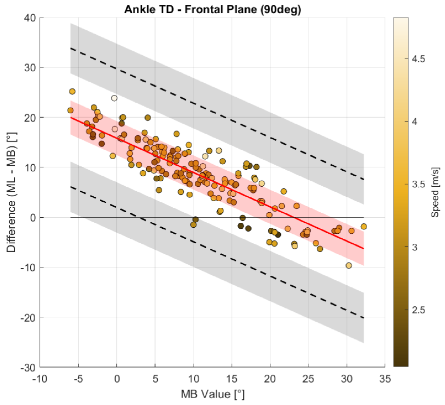

Supplementary Figure 69: Extended BA Plot for TD ankle angle - Frontal Plane (90deg)

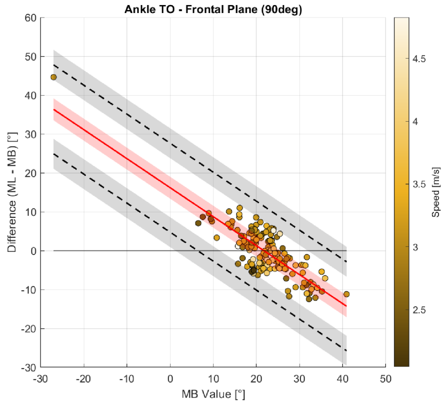

Supplementary Figure 70: Extended BA Plot for TO ankle angle - Frontal Plane (90deg)

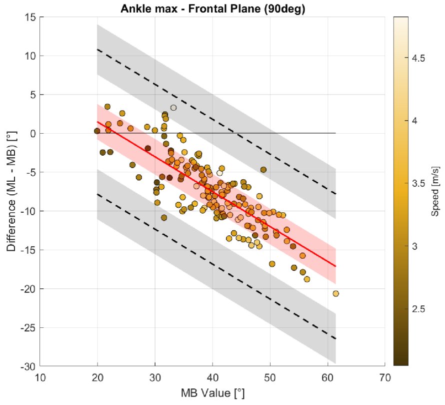

Supplementary Figure 71: Extended BA Plot for maximal ankle angle - Frontal Plane (90deg)

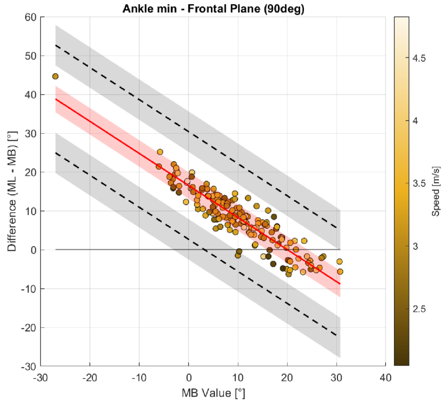

Supplementary Figure 72: Extended BA Plot for minimal ankle angle - Frontal Plane (90deg)

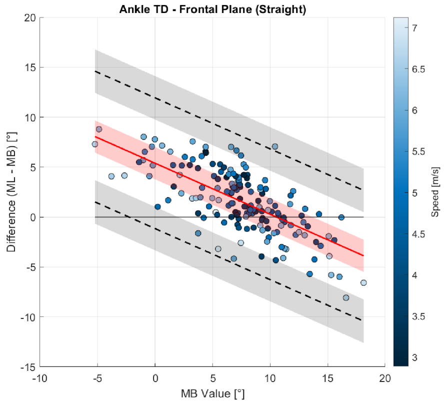

Supplementary Figure 73: Extended BA Plot for TD ankle angle - Frontal Plane (Straight)

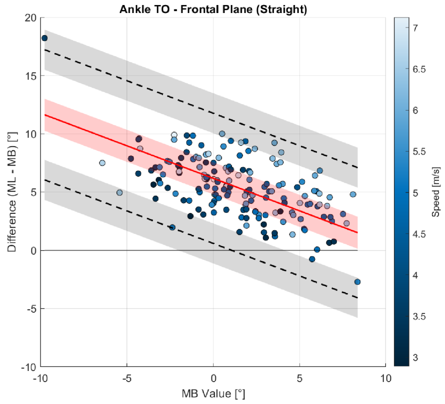

Supplementary Figure 74: Extended BA Plot for TO ankle angle - Frontal Plane (Straight)

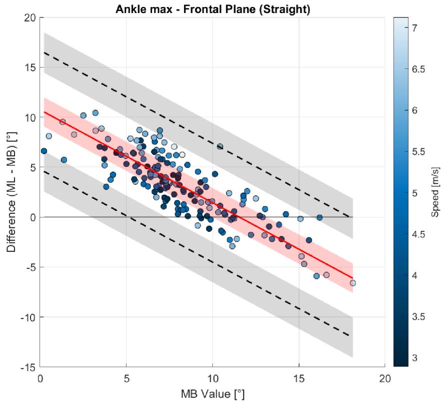

Supplementary Figure 75: Extended BA Plot for maximal ankle angle - Frontal Plane (Straight)

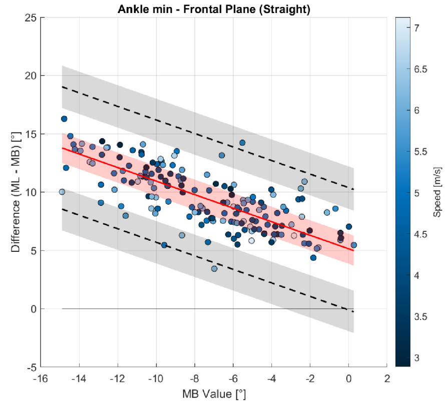

Supplementary Figure 76: Extended BA Plot for minimal ankle angle - Frontal Plane (Straight)

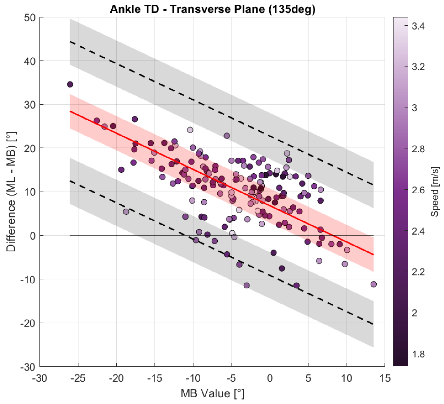

Supplementary Figure 77: Extended BA Plot for TD ankle angle - Transverse Plane (135deg)

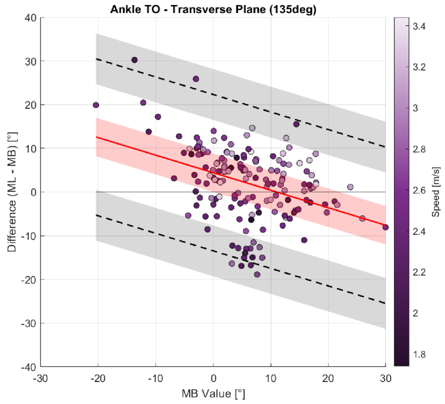

Supplementary Figure 78: Extended BA Plot for TO ankle angle - Transverse Plane (135deg)

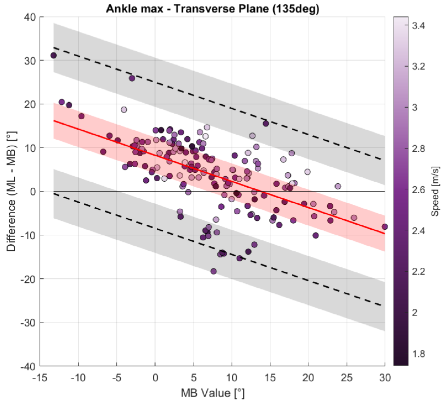

Supplementary Figure 79: Extended BA Plot for maximal ankle angle - Transverse Plane (135deg)

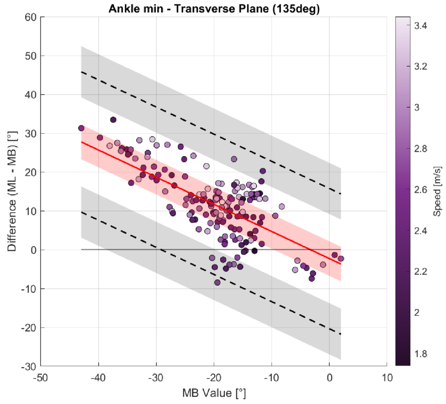

Supplementary Figure 80: Extended BA Plot for minimal ankle angle - Transverse Plane (135deg)

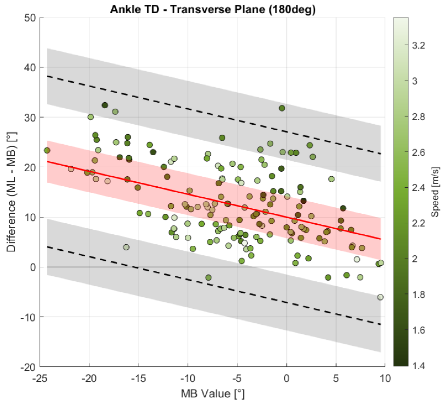

Supplementary Figure 81: Extended BA Plot for TD ankle angle - Transverse Plane (180deg)

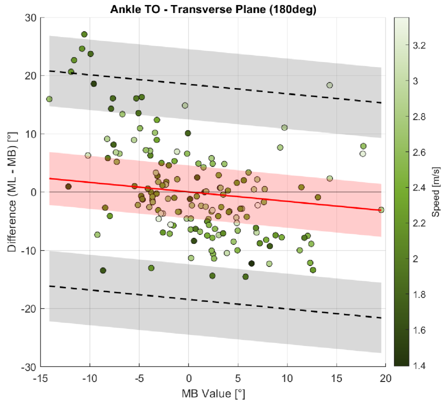

Supplementary Figure 82: Extended BA Plot for TO ankle angle - Transverse Plane (180deg)

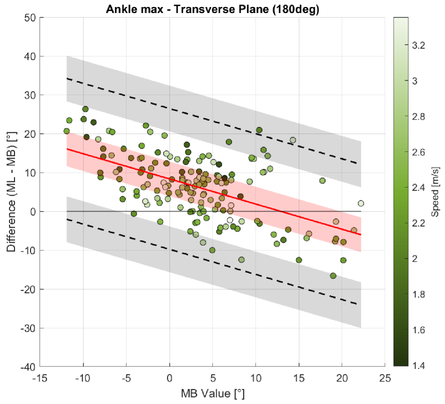

Supplementary Figure 83: Extended BA Plot for maximal ankle angle - Transverse Plane (180deg)

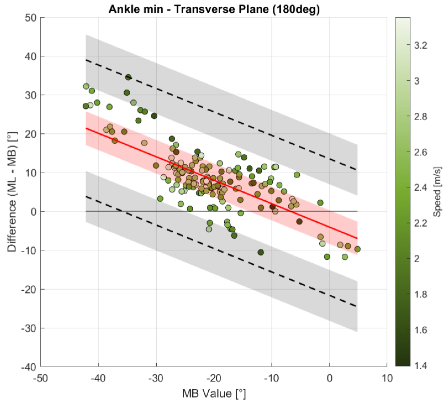

Supplementary Figure 84: Extended BA Plot for minimal ankle angle - Transverse Plane (180deg)

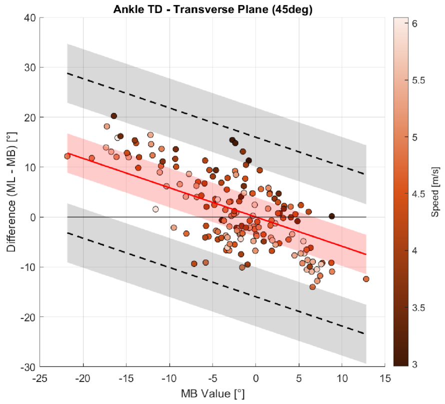

Supplementary Figure 85: Extended BA Plot for TD ankle angle - Transverse Plane (45deg)

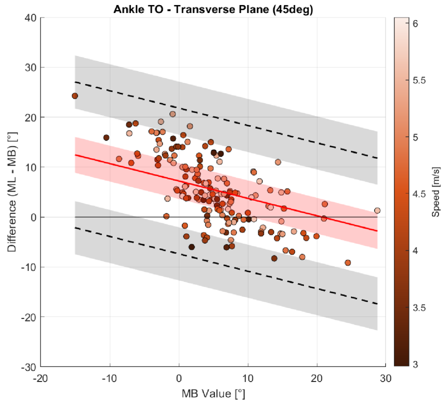

Supplementary Figure 86: Extended BA Plot for TO ankle angle - Transverse Plane (45deg)

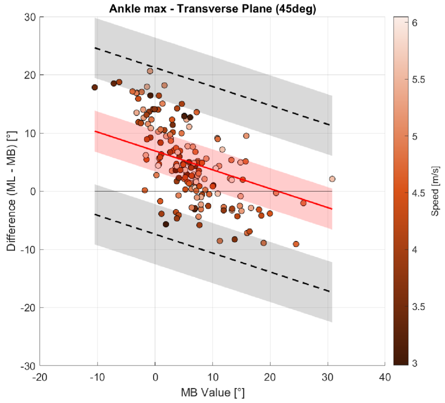

Supplementary Figure 87: Extended BA Plot for maximal ankle angle - Transverse Plane (45deg)

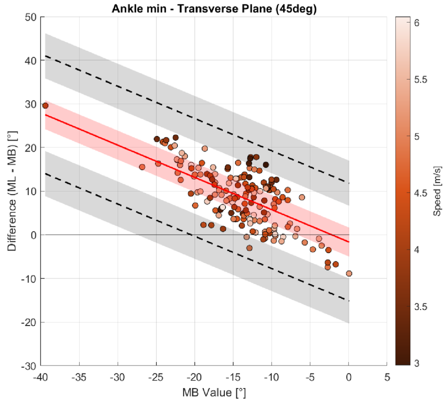

Supplementary Figure 88: Extended BA Plot for minimal ankle angle - Transverse Plane (45deg)

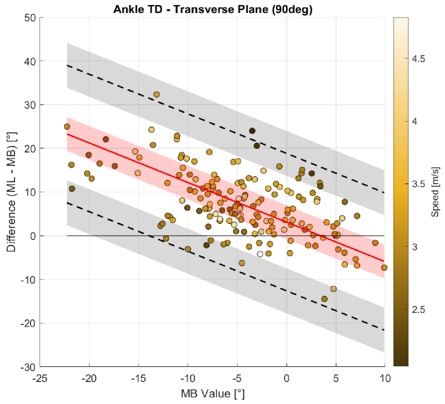

Supplementary Figure 89: Extended BA Plot for TD ankle angle - Transverse Plane (90deg)

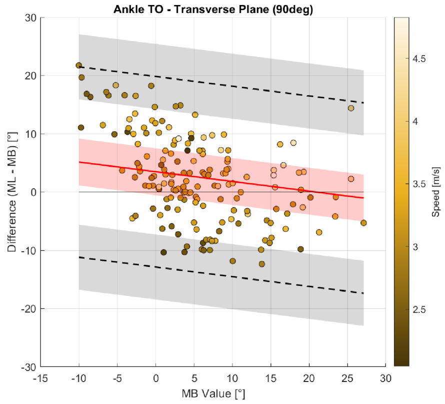

Supplementary Figure 90: Extended BA Plot for TO ankle angle - Transverse Plane (90deg)

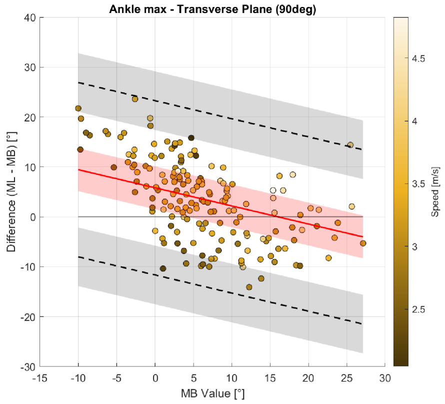

Supplementary Figure 91: Extended BA Plot for maximal ankle angle - Transverse Plane (90deg)

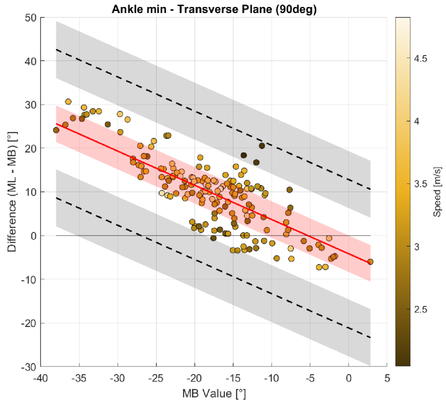

Supplementary Figure 92: Extended BA Plot for minimal ankle angle - Transverse Plane (90deg)

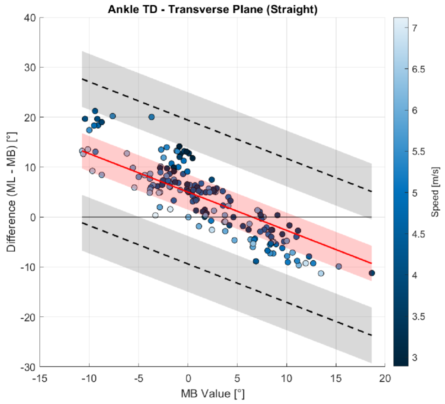

Supplementary Figure 93: Extended BA Plot for TD ankle angle - Transverse Plane (Straight)

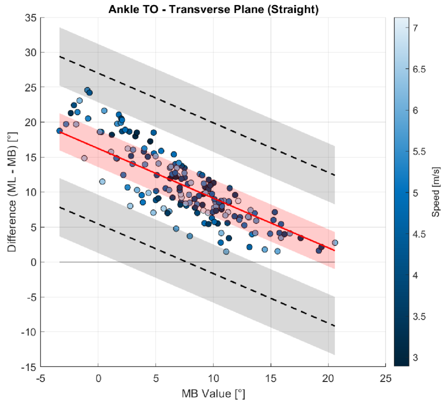

Supplementary Figure 94: Extended BA Plot for TO ankle angle - Transverse Plane (Straight)

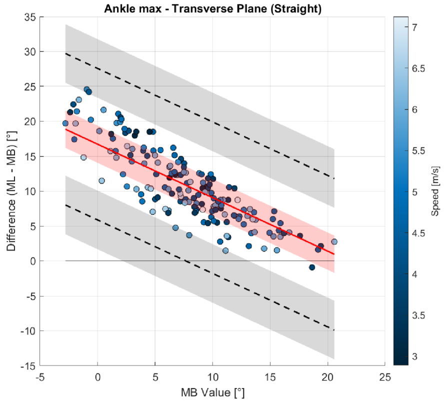

Supplementary Figure 95: Extended BA Plot for maximal ankle angle - Transverse Plane (Straight)

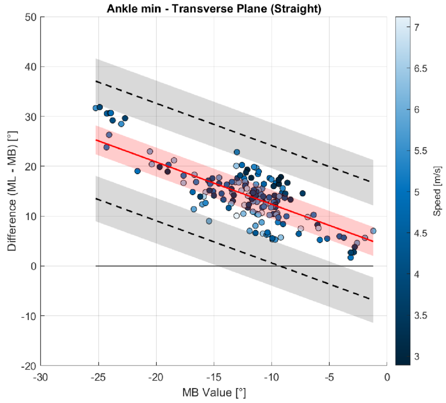

Supplementary Figure 96: Extended BA Plot for minimal ankle angle - Transverse Plane (Straight)

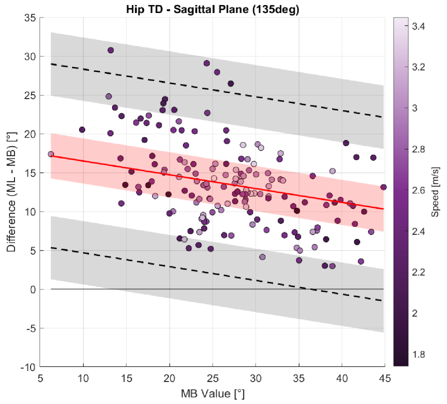

Supplementary Figure 97: Extended BA Plot for TD hip angle - Sagittal Plane (135deg)

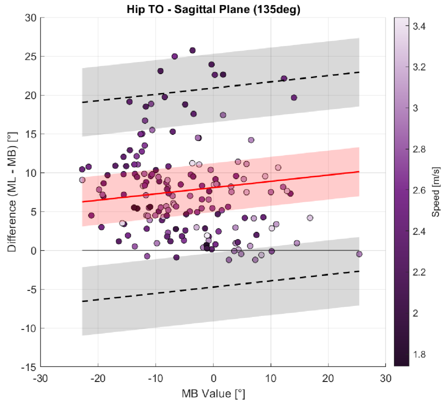

Supplementary Figure 98: Extended BA Plot for TO hip angle - Sagittal Plane (135deg)

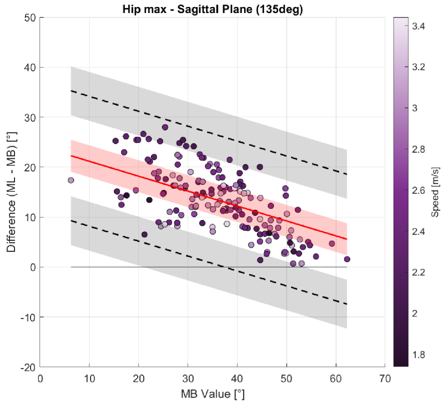

Supplementary Figure 99: Extended BA Plot for maximal hip angle - Sagittal Plane (135deg)

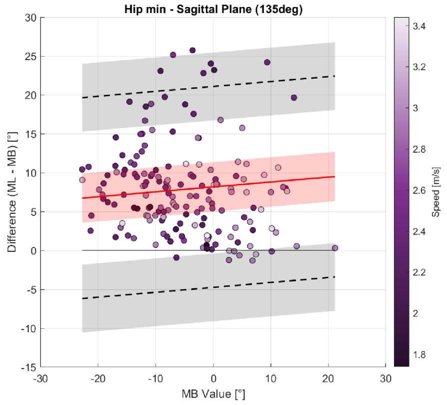

Supplementary Figure 100: Extended BA Plot for minimal hip angle - Sagittal Plane (135deg)

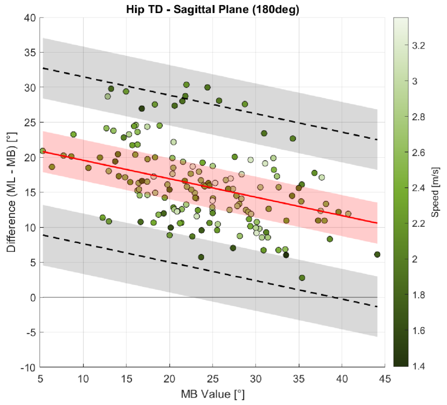

Supplementary Figure 101: Extended BA Plot for TD hip angle - Sagittal Plane (180deg)

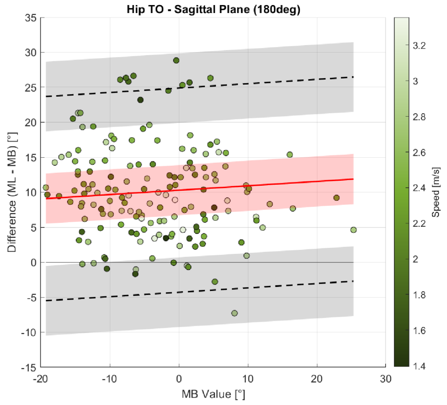

Supplementary Figure 102: Extended BA Plot for TO hip angle - Sagittal Plane (180deg)

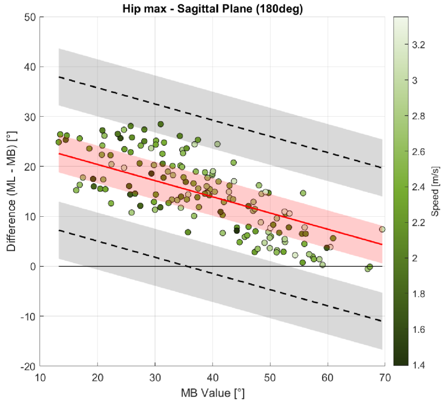

Supplementary Figure 103: Extended BA Plot for maximal hip angle - Sagittal Plane (180deg)

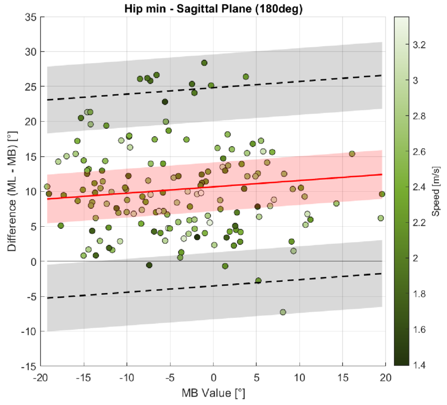

Supplementary Figure 104: Extended BA Plot for minimal hip angle - Sagittal Plane (180deg)

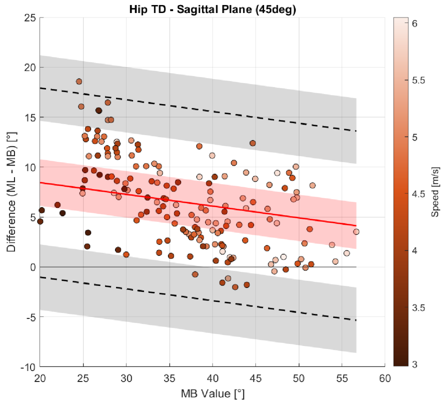

Supplementary Figure 105: Extended BA Plot for TD hip angle - Sagittal Plane (45deg)

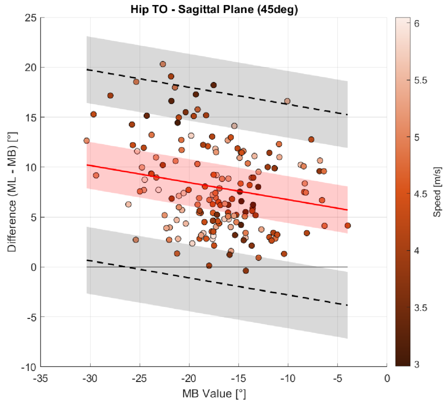

Supplementary Figure 106: Extended BA Plot for TO hip angle - Sagittal Plane (45deg)

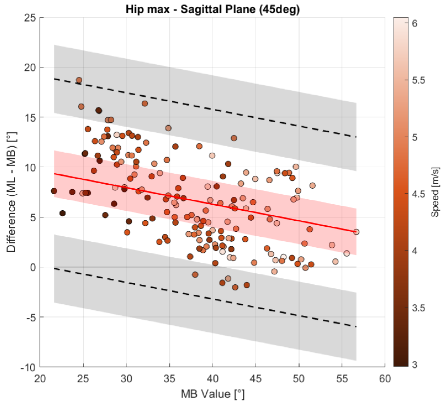

Supplementary Figure 107: Extended BA Plot for maximal hip angle - Sagittal Plane (45deg)

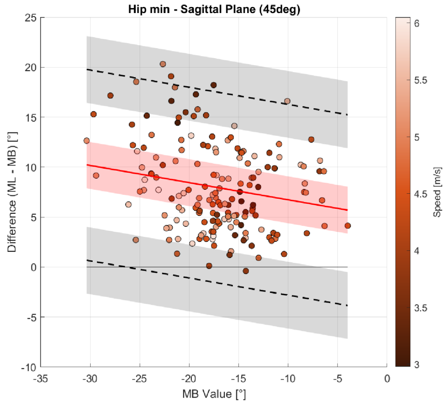

Supplementary Figure 108: Extended BA Plot for minimal hip angle - Sagittal Plane (45deg)

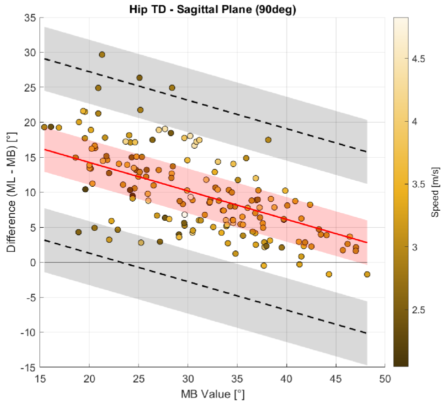

Supplementary Figure 109: Extended BA Plot for TD hip angle - Sagittal Plane (90deg)

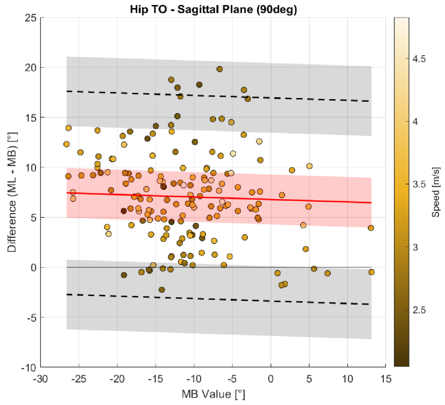

Supplementary Figure 110: Extended BA Plot for TO hip angle - Sagittal Plane (90deg)

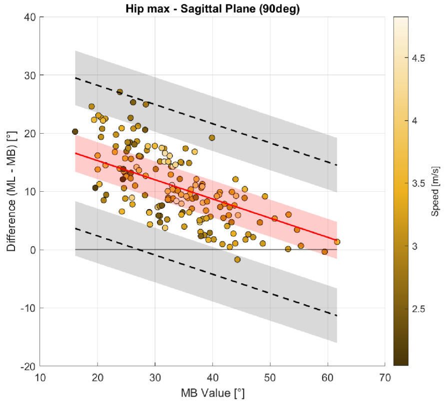

Supplementary Figure 111: Extended BA Plot for maximal hip angle - Sagittal Plane (90deg)

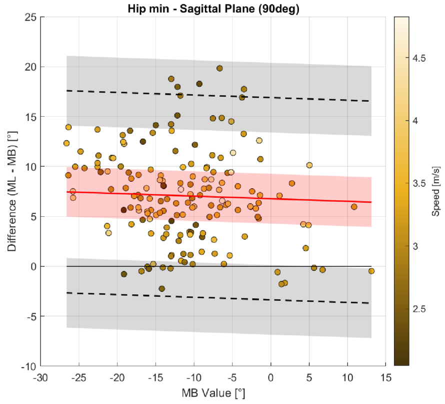

Supplementary Figure 112: Extended BA Plot for minimal hip angle - Sagittal Plane (90deg)

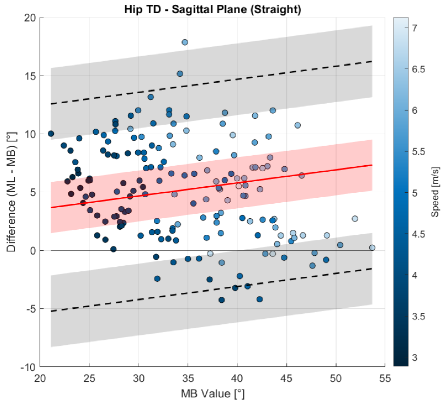

Supplementary Figure 113: Extended BA Plot for TD hip angle - Sagittal Plane (Straight)

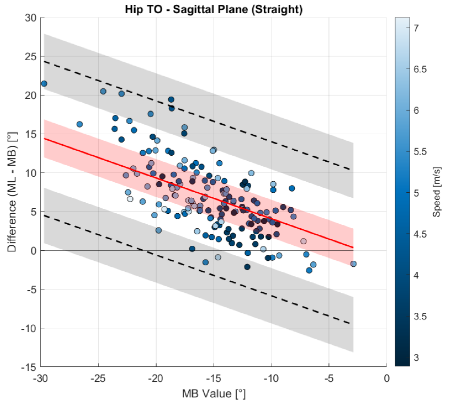

Supplementary Figure 114: Extended BA Plot for TO hip angle - Sagittal Plane (Straight)

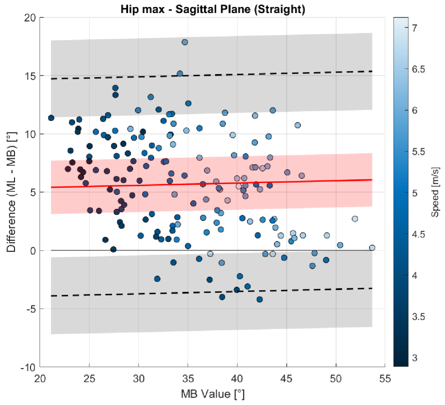

Supplementary Figure 115: Extended BA Plot for maximal hip angle - Sagittal Plane (Straight)

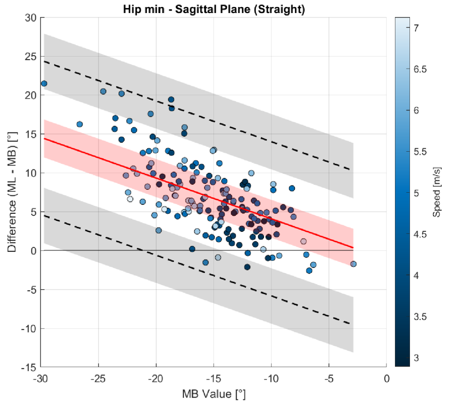

Supplementary Figure 116: Extended BA Plot for minimal hip angle - Sagittal Plane (Straight)

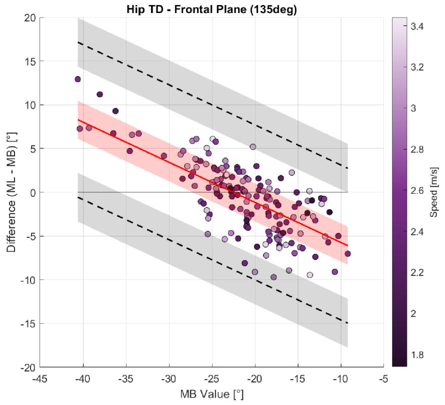

Supplementary Figure 117: Extended BA Plot for TD hip angle - Frontal Plane (135deg)

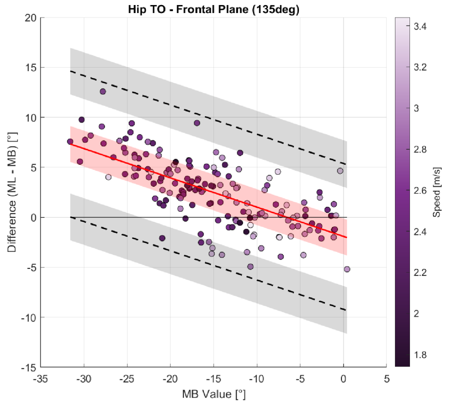

Supplementary Figure 118: Extended BA Plot for TO hip angle - Frontal Plane (135deg)

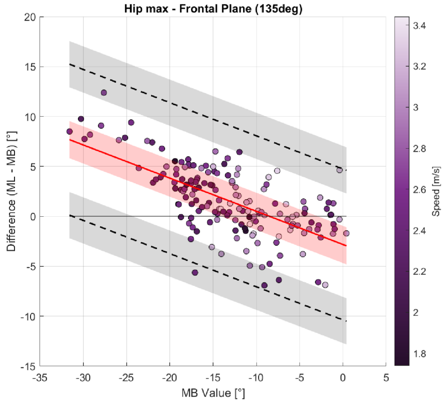

Supplementary Figure 119: Extended BA Plot for maximal hip angle - Frontal Plane (135deg)

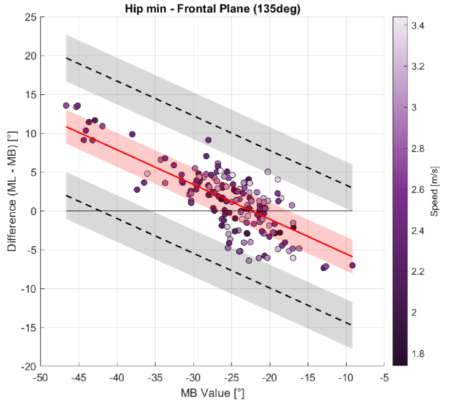

Supplementary Figure 120: Extended BA Plot for minimal hip angle - Frontal Plane (135deg)

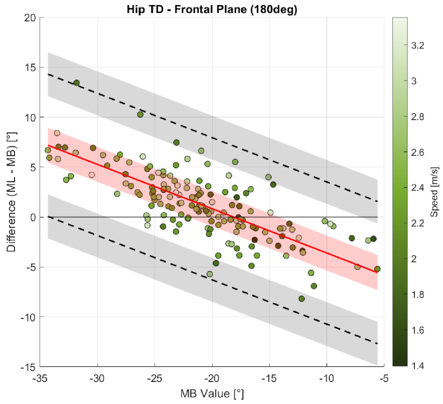

Supplementary Figure 121: Extended BA Plot for TD hip angle - Frontal Plane (180deg)

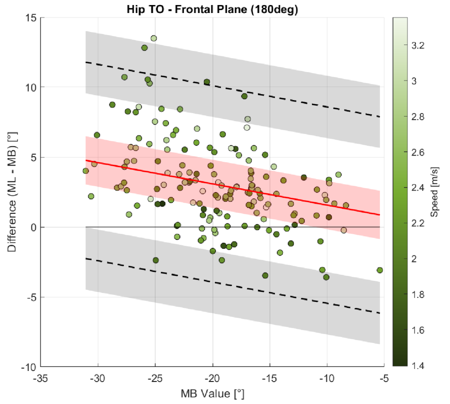

Supplementary Figure 122: Extended BA Plot for TO hip angle - Frontal Plane (180deg)

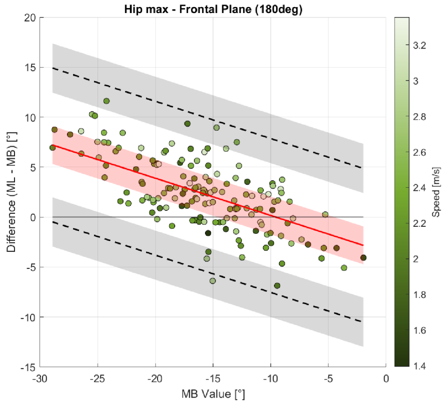

Supplementary Figure 123: Extended BA Plot for maximal hip angle - Frontal Plane (180deg)

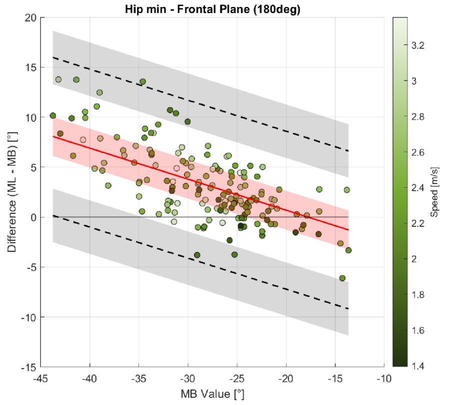

Supplementary Figure 124: Extended BA Plot for minimal hip angle - Frontal Plane (180deg)

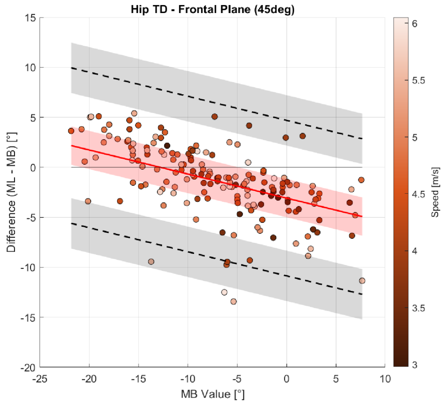

Supplementary Figure 125: Extended BA Plot for TD hip angle - Frontal Plane (45deg)

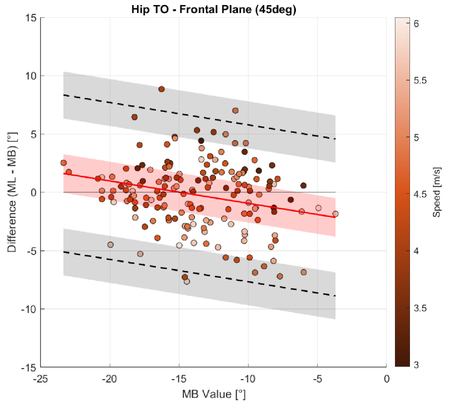

Supplementary Figure 126: Extended BA Plot for TO hip angle - Frontal Plane (45deg)

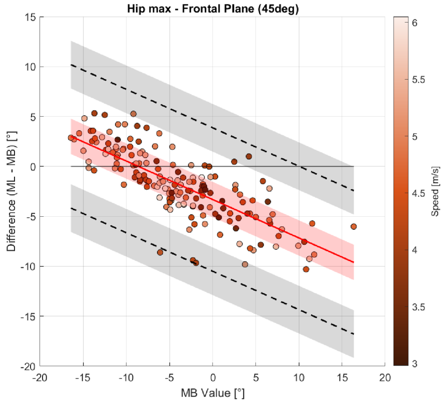

Supplementary Figure 127: Extended BA Plot for maximal hip angle - Frontal Plane (45deg)

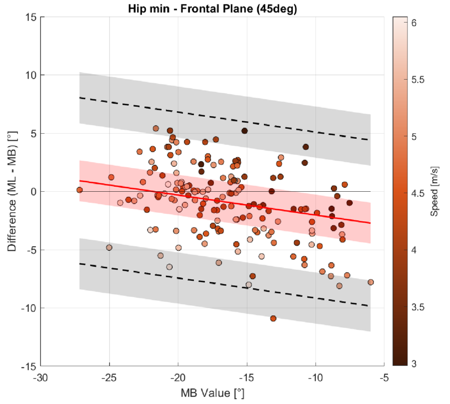

Supplementary Figure 128: Extended BA Plot for minimal hip angle - Frontal Plane (45deg)

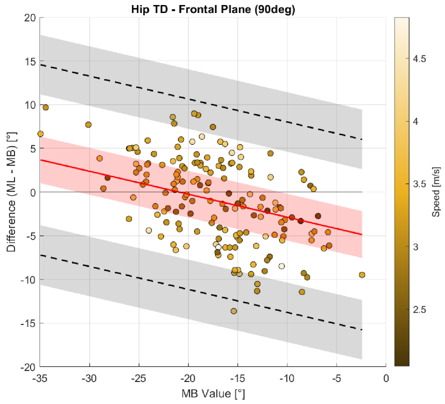

Supplementary Figure 129: Extended BA Plot for TD hip angle - Frontal Plane (90deg)

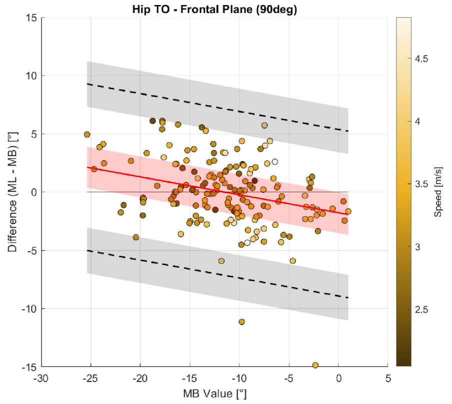

Supplementary Figure 130: Extended BA Plot for TO hip angle - Frontal Plane (90deg)

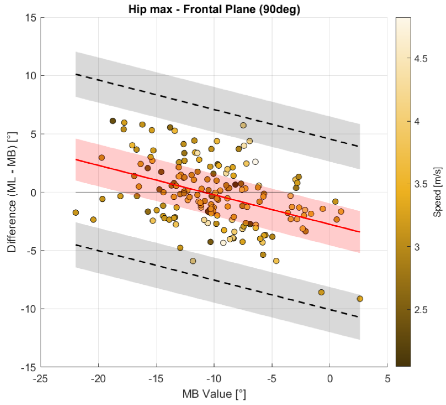

Supplementary Figure 131: Extended BA Plot for maximal hip angle - Frontal Plane (90deg)

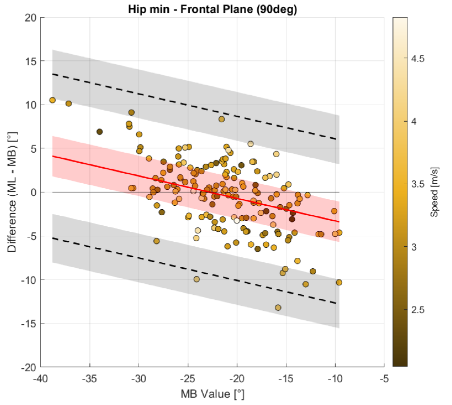

Supplementary Figure 132: Extended BA Plot for minimal hip angle - Frontal Plane (90deg)

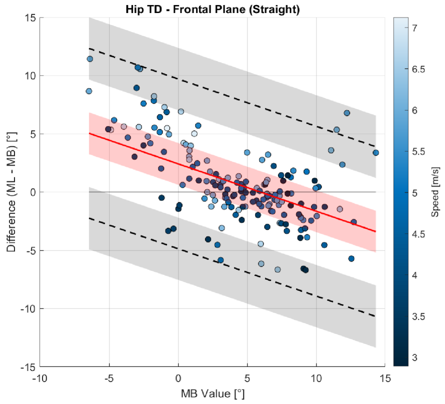

Supplementary Figure 133: Extended BA Plot for TD hip angle - Frontal Plane (Straight)

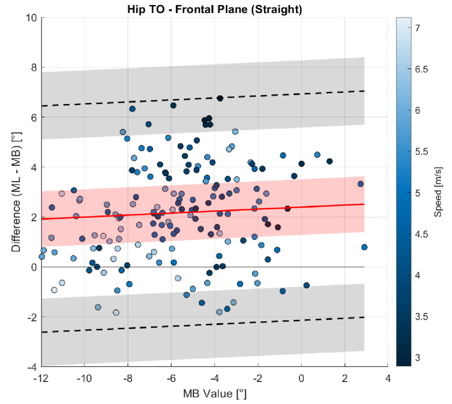

Supplementary Figure 134: Extended BA Plot for TO hip angle - Frontal Plane (Straight)

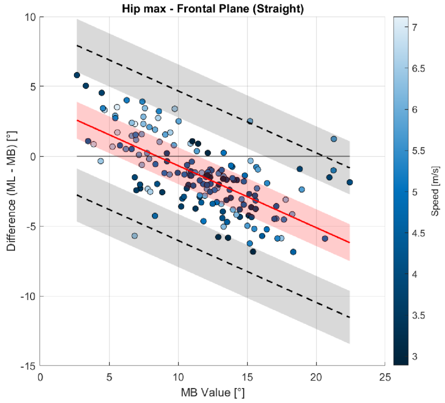

Supplementary Figure 135: Extended BA Plot for maximal hip angle - Frontal Plane (Straight)

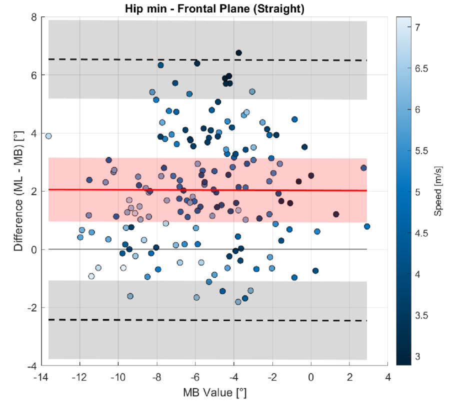

Supplementary Figure 136: Extended BA Plot for minimal hip angle - Frontal Plane (Straight)

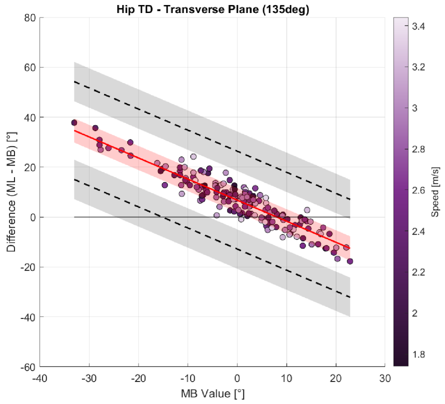

Supplementary Figure 137: Extended BA Plot for TD hip angle - Transverse Plane (135deg)

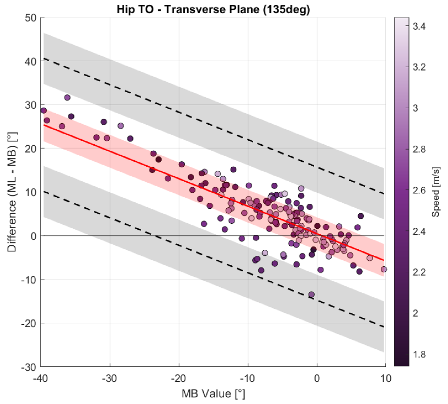

Supplementary Figure 138: Extended BA Plot for TO hip angle - Transverse Plane (135deg)

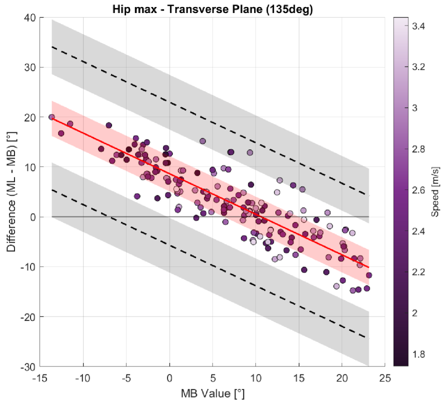

Supplementary Figure 139: Extended BA Plot for maximal hip angle - Transverse Plane (135deg)

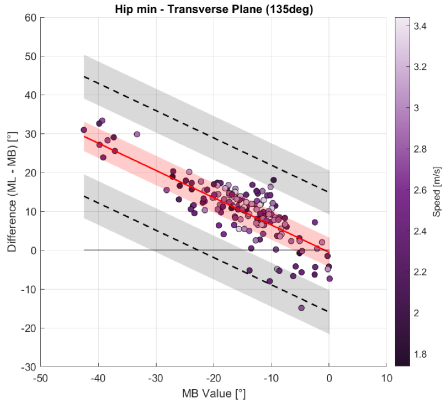

Supplementary Figure 140: Extended BA Plot for minimal hip angle - Transverse Plane (135deg)

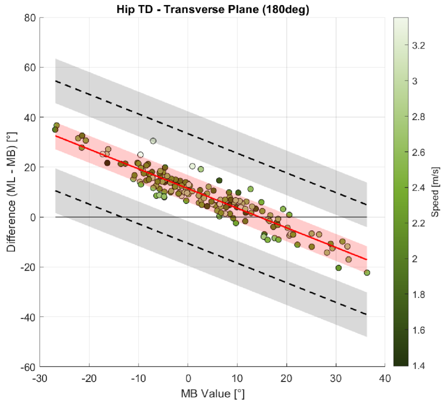

Supplementary Figure 141: Extended BA Plot for TD hip angle - Transverse Plane (180deg)

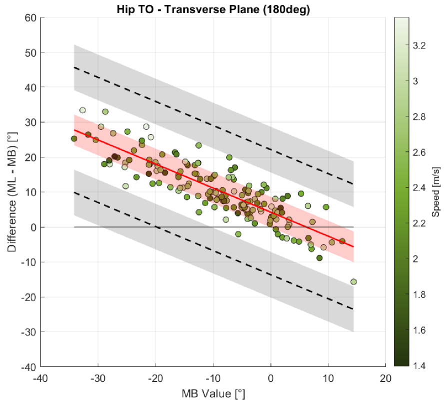

Supplementary Figure 142: Extended BA Plot for TO hip angle - Transverse Plane (180deg)

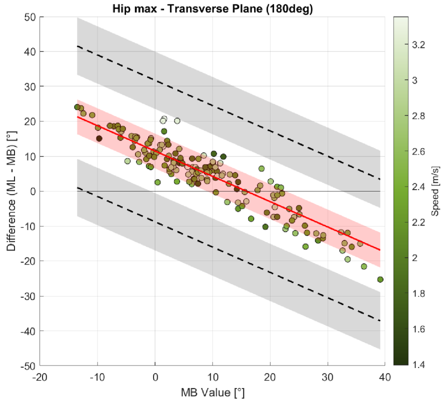

Supplementary Figure 143: Extended BA Plot for maximal hip angle - Transverse Plane (180deg)

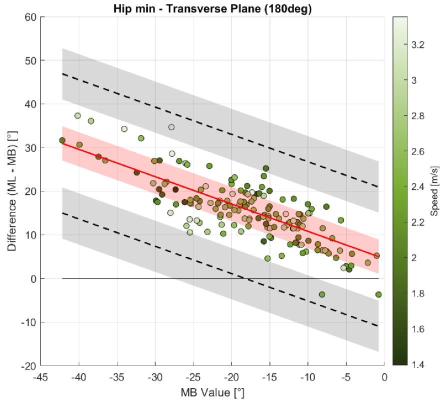

Supplementary Figure 144: Extended BA Plot for minimal hip angle - Transverse Plane (180deg)

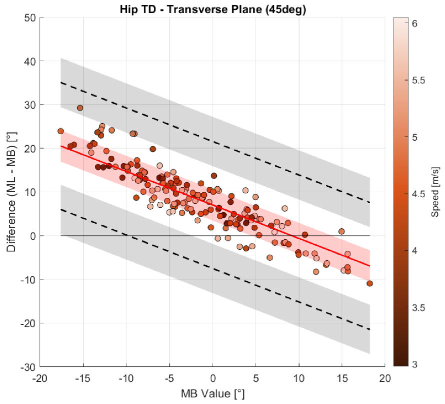

Supplementary Figure 145: Extended BA Plot for TD hip angle - Transverse Plane (45deg)

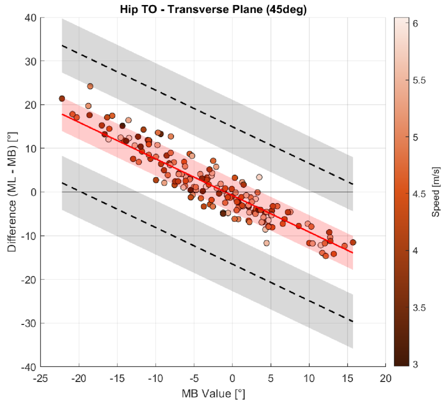

Supplementary Figure 146: Extended BA Plot for TO hip angle - Transverse Plane (45deg)

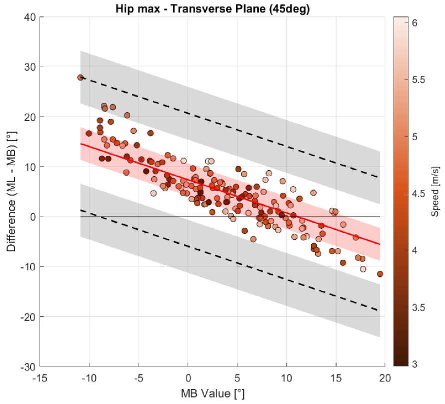

Supplementary Figure 147: Extended BA Plot for maximal hip angle - Transverse Plane (45deg)

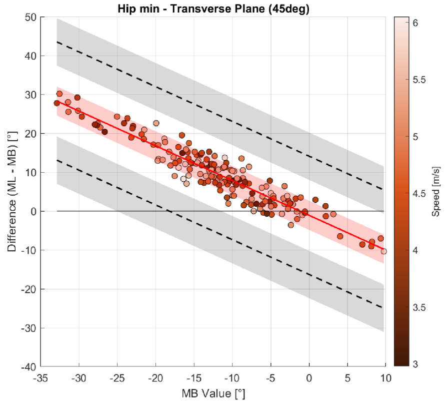

Supplementary Figure 148: Extended BA Plot for minimal hip angle - Transverse Plane (45deg)

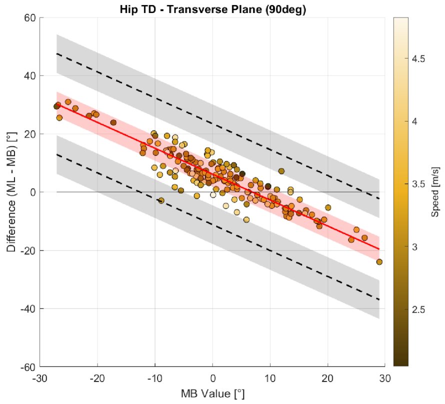

Supplementary Figure 149: Extended BA Plot for TD hip angle - Transverse Plane (90deg)

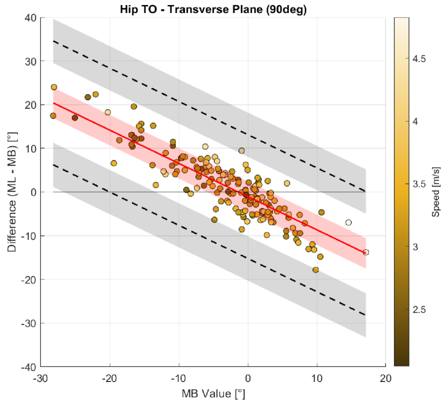

Supplementary Figure 150: Extended BA Plot for TO hip angle - Transverse Plane (90deg)

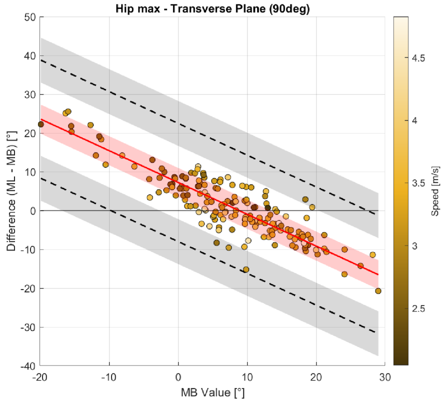

Supplementary Figure 151: Extended BA Plot for maximal hip angle - Transverse Plane (90deg)

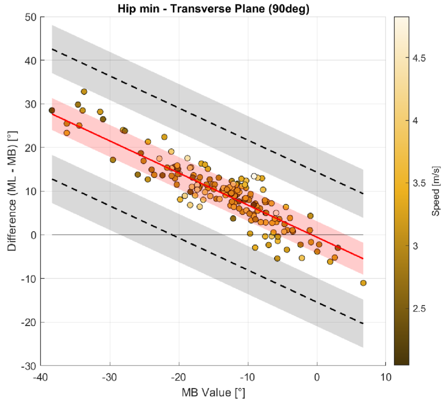

Supplementary Figure 152: Extended BA Plot for minimal hip angle - Transverse Plane (90deg)

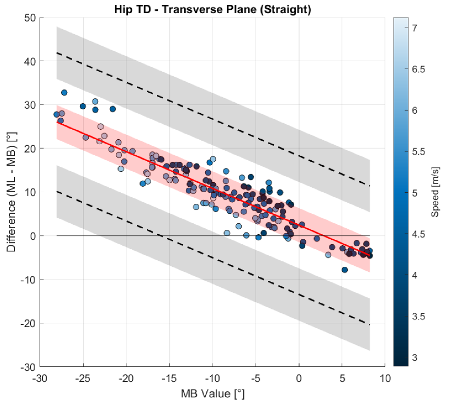

Supplementary Figure 153: Extended BA Plot for TD hip angle - Transverse Plane (Straight)

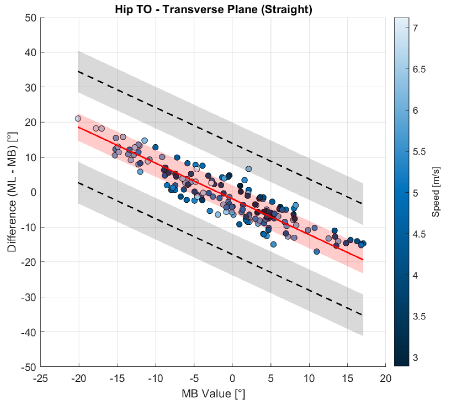

Supplementary Figure 154: Extended BA Plot for TO hip angle - Transverse Plane (Straight)

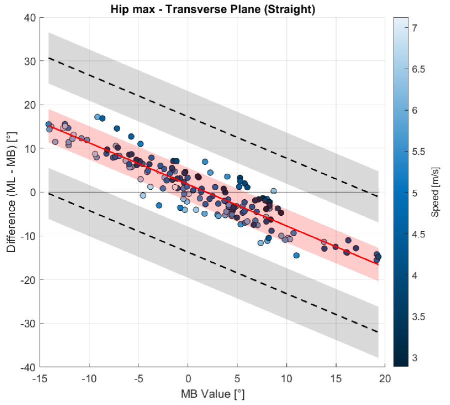

Supplementary Figure 155: Extended BA Plot for maximal hip angle - Transverse Plane (Straight)

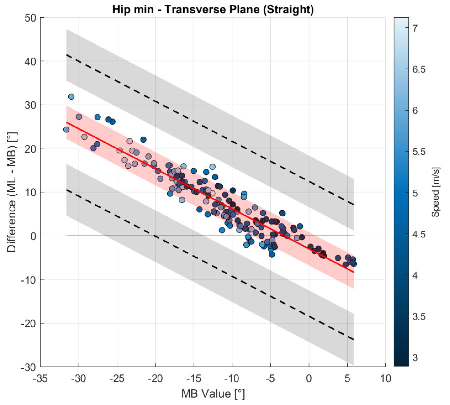

Supplementary Figure 156: Extended BA Plot for minimal hip angle - Transverse Plane (Straight)

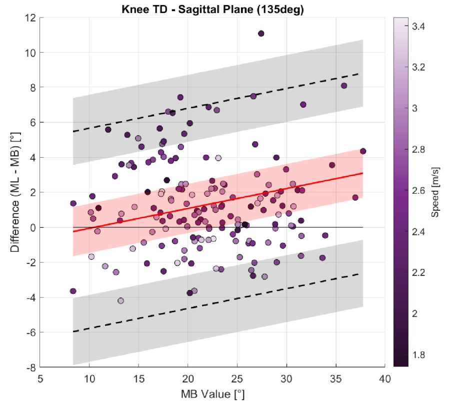

Supplementary Figure 157: Extended BA Plot for TD knee angle - Sagittal Plane (135deg)

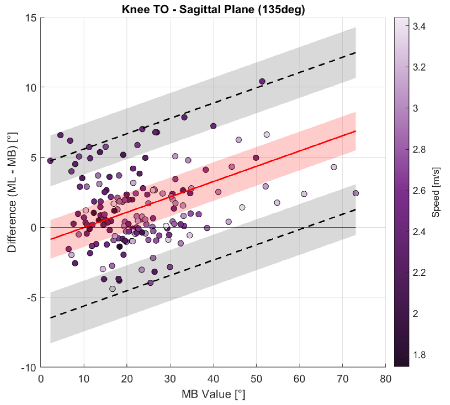

Supplementary Figure 158: Extended BA Plot for TO knee angle - Sagittal Plane (135deg)

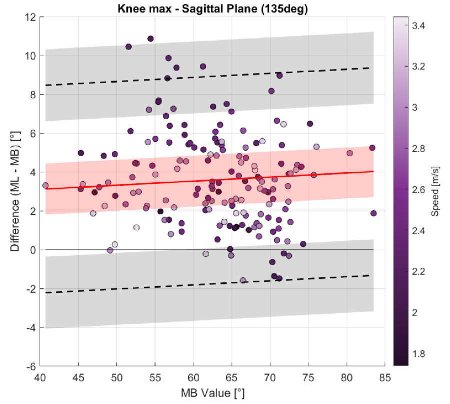

Supplementary Figure 159: Extended BA Plot for maximal knee angle - Sagittal Plane (135deg)

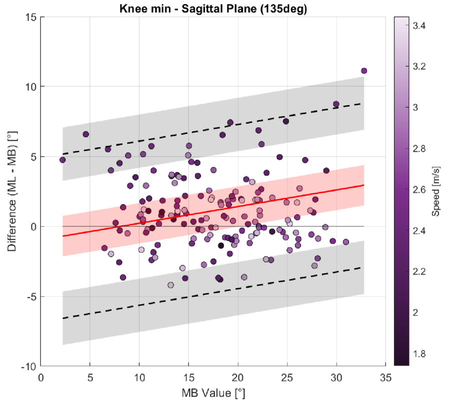

Supplementary Figure 160: Extended BA Plot for minimal knee angle - Sagittal Plane (135deg)

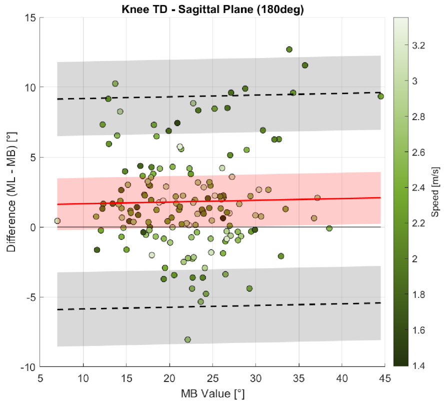

Supplementary Figure 161: Extended BA Plot for TD knee angle - Sagittal Plane (180deg)

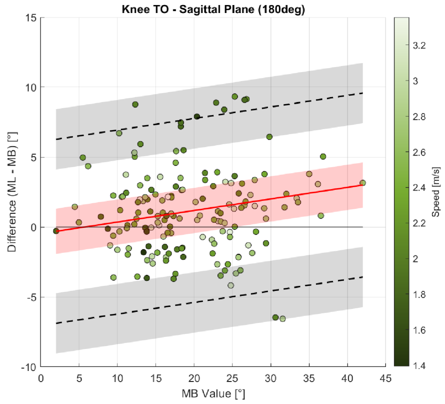

Supplementary Figure 162: Extended BA Plot for TO knee angle - Sagittal Plane (180deg)

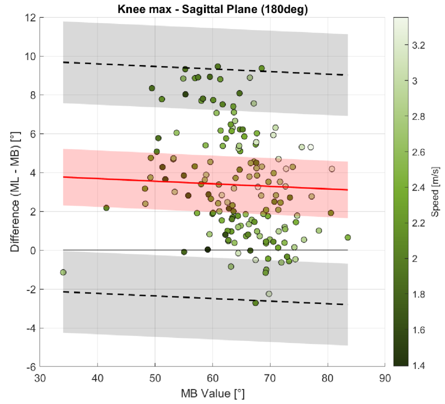

Supplementary Figure 163: Extended BA Plot for maximal knee angle - Sagittal Plane (180deg)

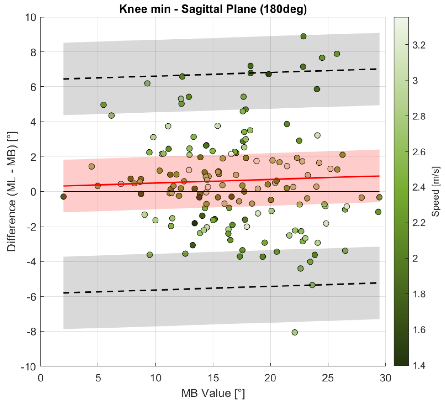

Supplementary Figure 164: Extended BA Plot for minimal knee angle - Sagittal Plane (180deg)

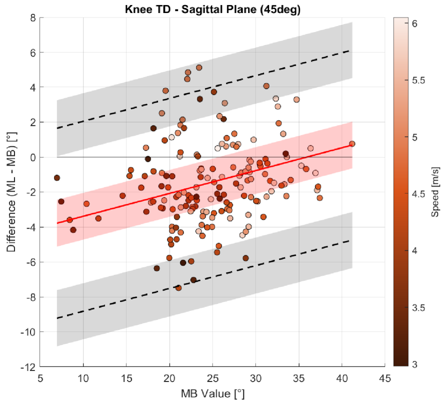

Supplementary Figure 165: Extended BA Plot for TD knee angle - Sagittal Plane (45deg)

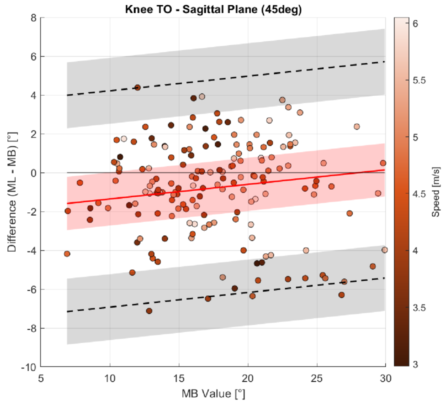

Supplementary Figure 166: Extended BA Plot for TO knee angle - Sagittal Plane (45deg)

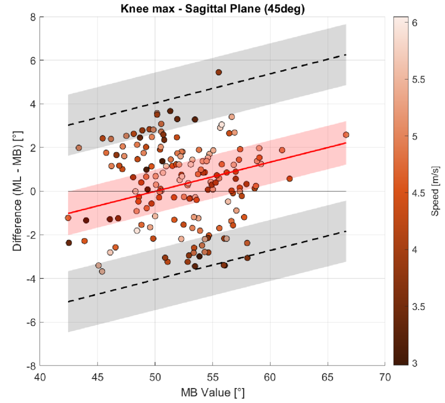

Supplementary Figure 167: Extended BA Plot for maximal knee angle - Sagittal Plane (45deg)

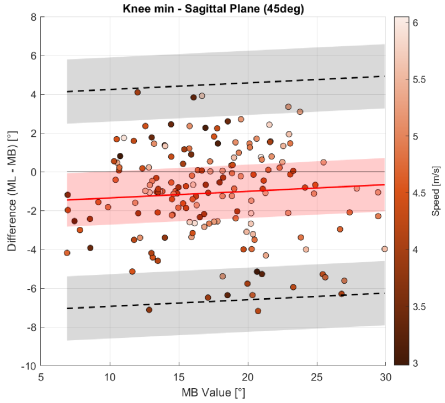

Supplementary Figure 168: Extended BA Plot for minimal knee angle - Sagittal Plane (45deg)

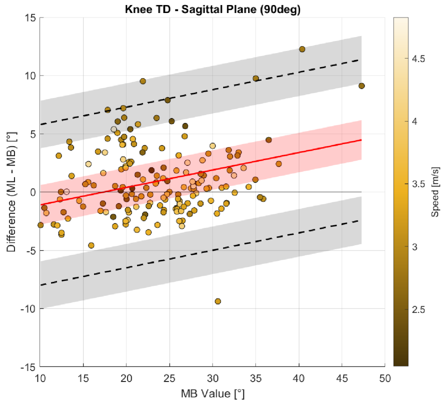

Supplementary Figure 169: Extended BA Plot for TD knee angle - Sagittal Plane (90deg)

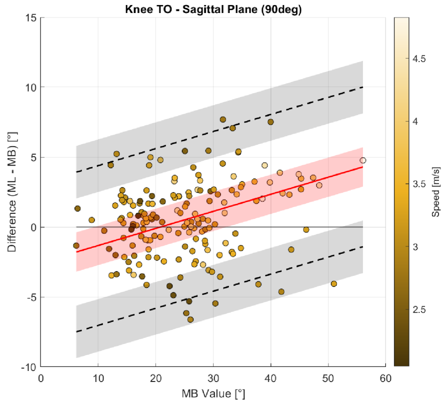

Supplementary Figure 170: Extended BA Plot for TO knee angle - Sagittal Plane (90deg)

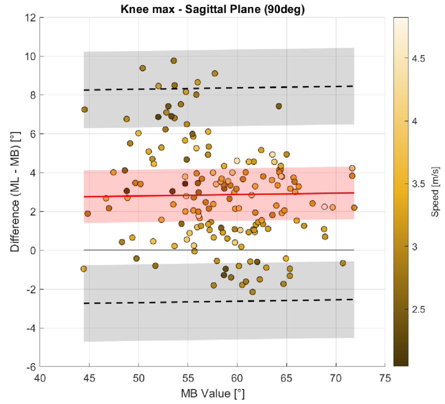

Supplementary Figure 171: Extended BA Plot for maximal knee angle - Sagittal Plane (90deg)

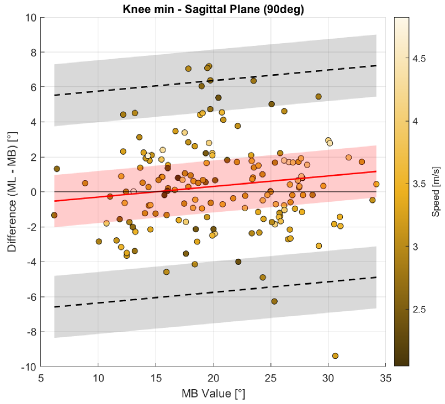

Supplementary Figure 172: Extended BA Plot for minimal knee angle - Sagittal Plane (90deg)

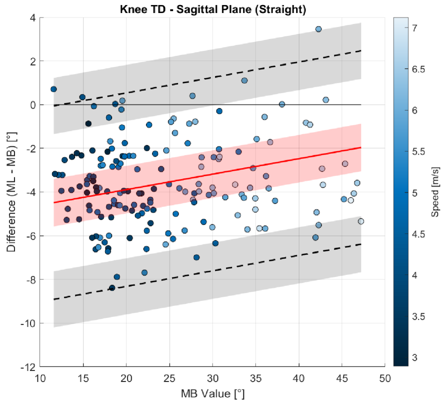

Supplementary Figure 173: Extended BA Plot for TD knee angle - Sagittal Plane (Straight)

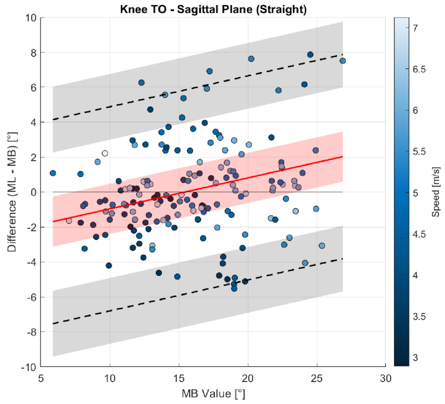

Supplementary Figure 174: Extended BA Plot for TO knee angle - Sagittal Plane (Straight)

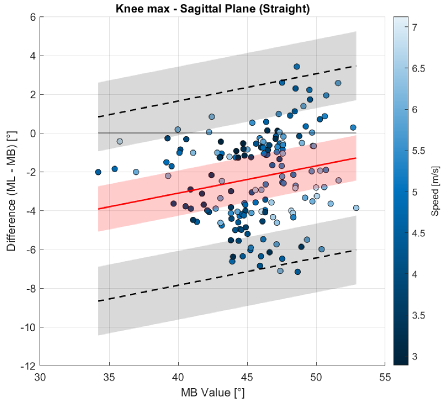

Supplementary Figure 175: Extended BA Plot for maximal knee angle - Sagittal Plane (Straight)

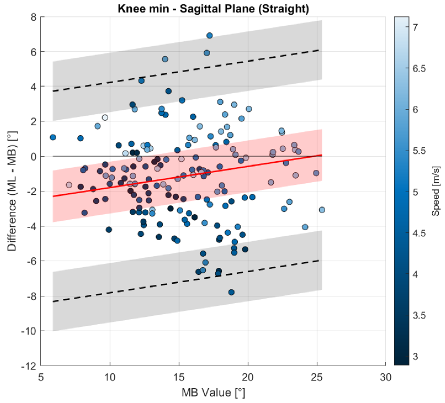

Supplementary Figure 176: Extended BA Plot for minimal knee angle - Sagittal Plane (Straight)

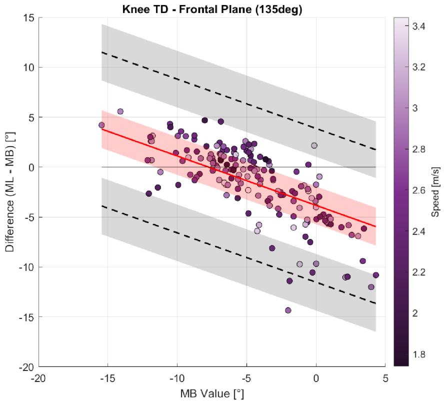

Supplementary Figure 177: Extended BA Plot for TD knee angle - Frontal Plane (135deg)

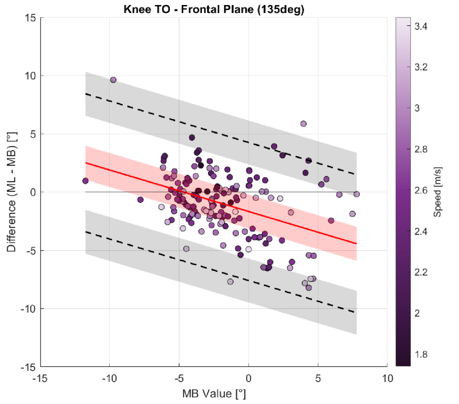

Supplementary Figure 178: Extended BA Plot for TO knee angle - Frontal Plane (135deg)

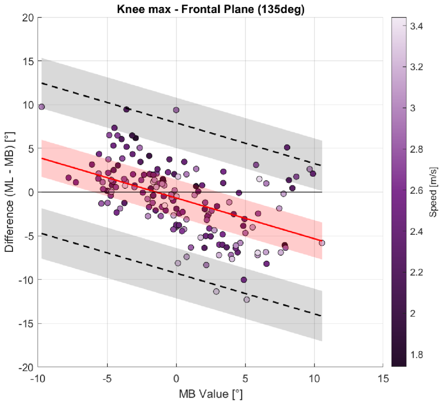

Supplementary Figure 179: Extended BA Plot for maximal knee angle - Frontal Plane (135deg)

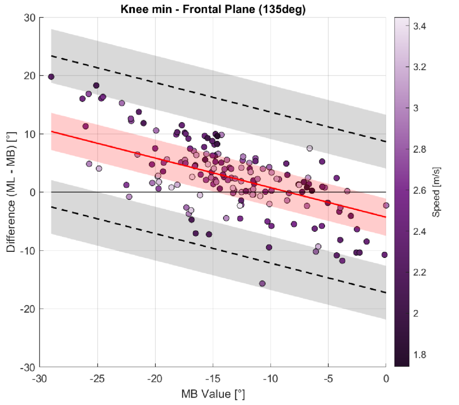

Supplementary Figure 180: Extended BA Plot for minimal knee angle - Frontal Plane (135deg)

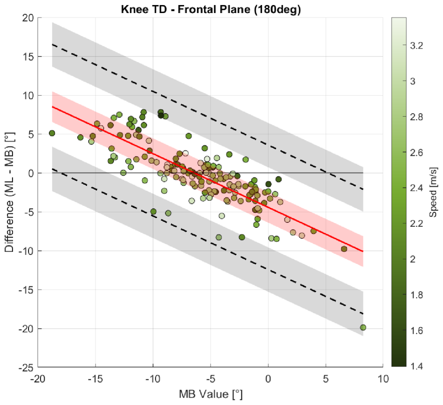

Supplementary Figure 181: Extended BA Plot for TD knee angle - Frontal Plane (180deg)

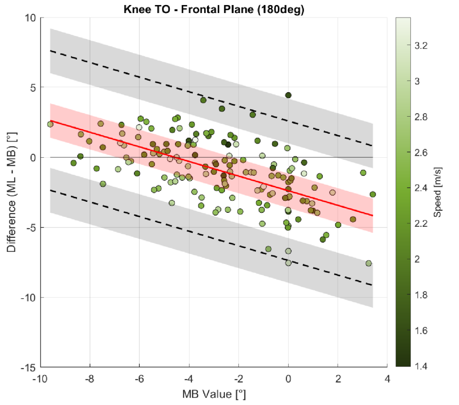

Supplementary Figure 182: Extended BA Plot for TO knee angle - Frontal Plane (180deg)

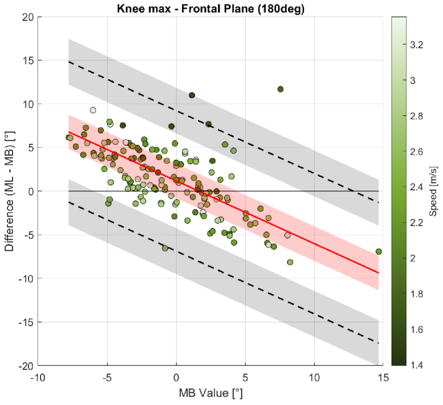

Supplementary Figure 183: Extended BA Plot for maximal knee angle - Frontal Plane (180deg)

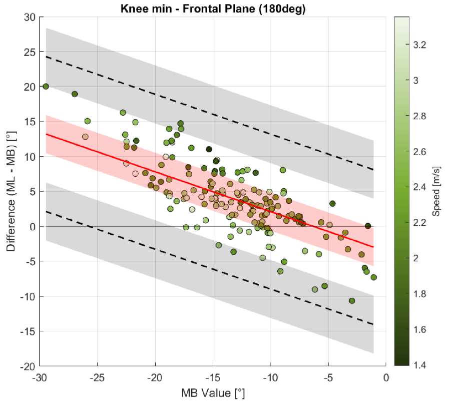

Supplementary Figure 184: Extended BA Plot for minimal knee angle - Frontal Plane (180deg)

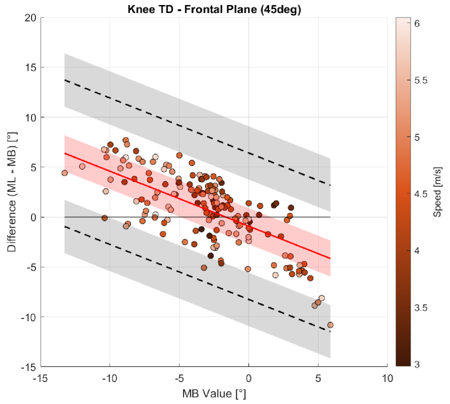

Supplementary Figure 185: Extended BA Plot for TD knee angle - Frontal Plane (45deg)

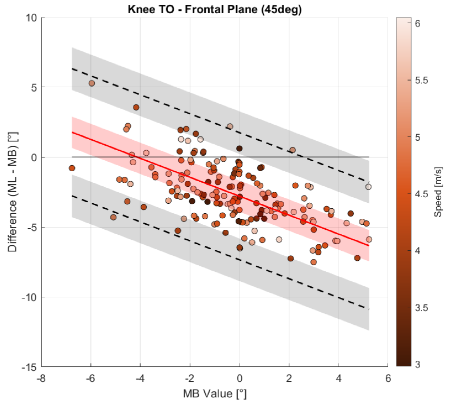

Supplementary Figure 186: Extended BA Plot for TO knee angle - Frontal Plane (45deg)

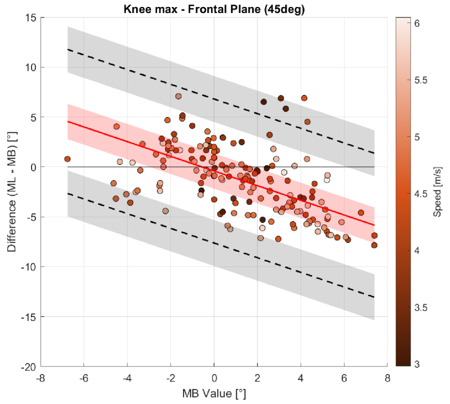

Supplementary Figure 187: Extended BA Plot for maximal knee angle - Frontal Plane (45deg)

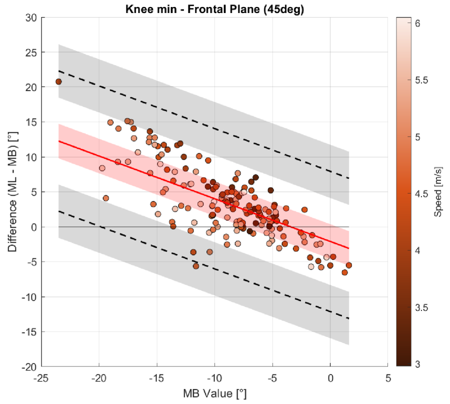

Supplementary Figure 188: Extended BA Plot for minimal knee angle - Frontal Plane (45deg)

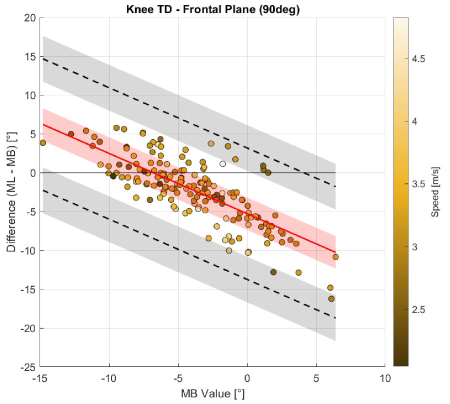

Supplementary Figure 189: Extended BA Plot for TD knee angle - Frontal Plane (90deg)

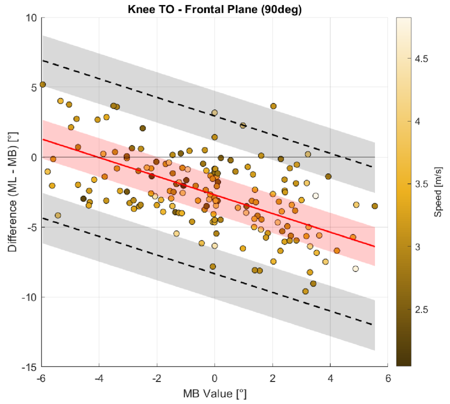

Supplementary Figure 190: Extended BA Plot for TO knee angle - Frontal Plane (90deg)

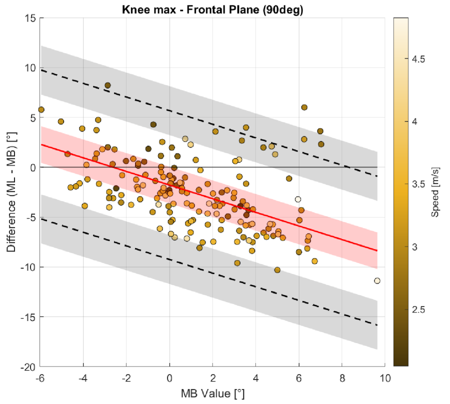

Supplementary Figure 191: Extended BA Plot for maximal knee angle - Frontal Plane (90deg)

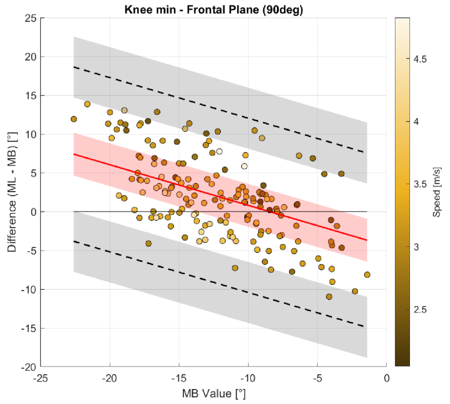

Supplementary Figure 192: Extended BA Plot for minimal knee angle - Frontal Plane (90deg)

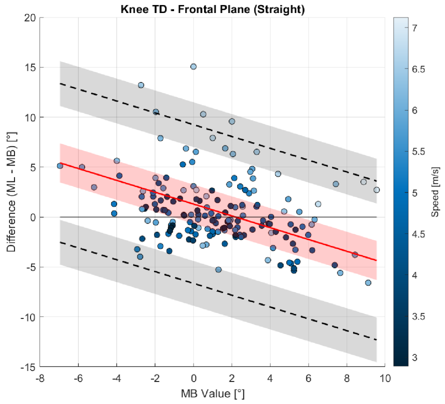

Supplementary Figure 193: Extended BA Plot for TD knee angle - Frontal Plane (Straight)

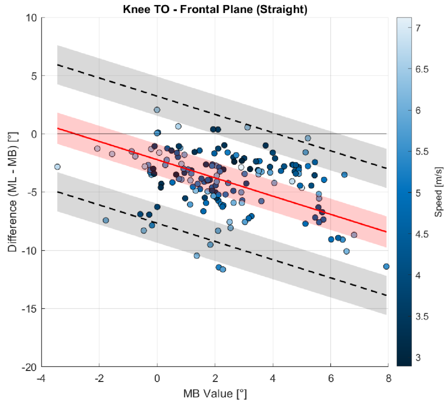

Supplementary Figure 194: Extended BA Plot for TO knee angle - Frontal Plane (Straight)

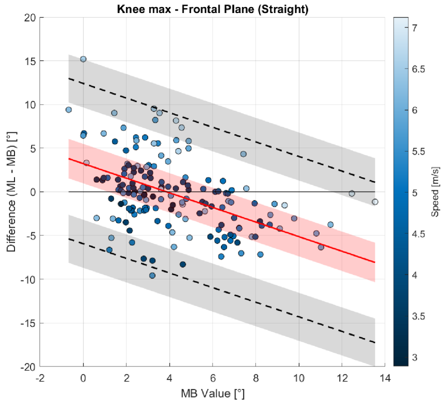

Supplementary Figure 195: Extended BA Plot for maximal knee angle - Frontal Plane (Straight)

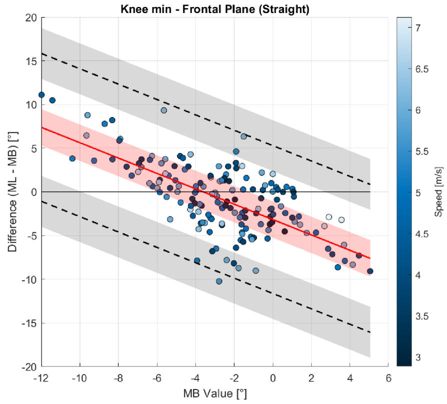

Supplementary Figure 196: Extended BA Plot for minimal knee angle - Frontal Plane (Straight)

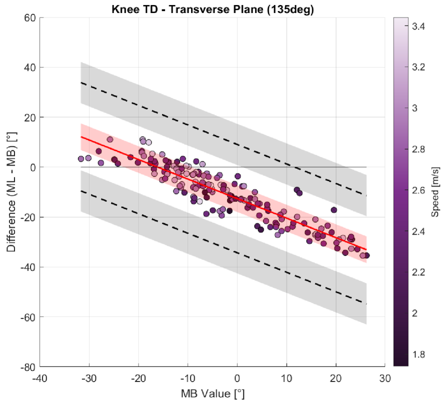

Supplementary Figure 197: Extended BA Plot for TD knee angle - Transverse Plane (135deg)

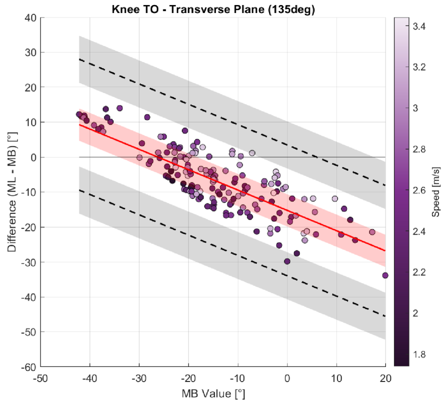

Supplementary Figure 198: Extended BA Plot for TO knee angle - Transverse Plane (135deg)

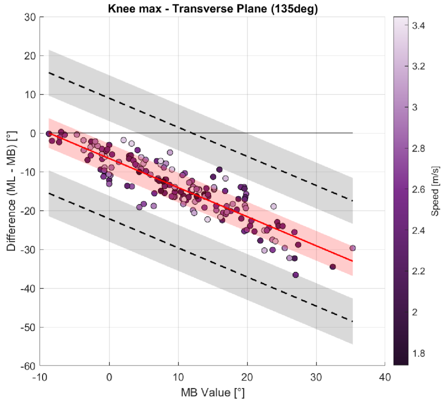

Supplementary Figure 199: Extended BA Plot for maximal knee angle - Transverse Plane (135deg)

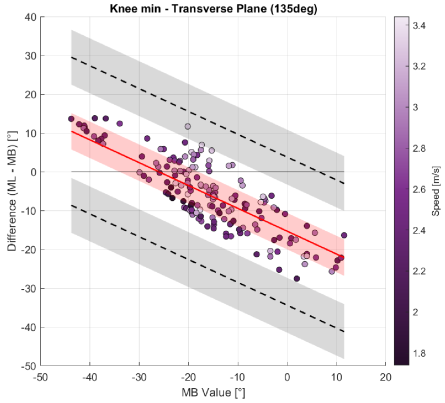

Supplementary Figure 200: Extended BA Plot for minimal knee angle - Transverse Plane (135deg)

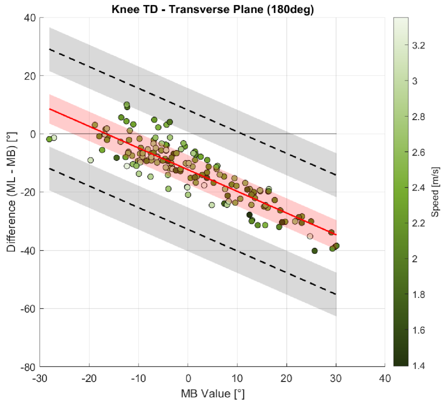

Supplementary Figure 201: Extended BA Plot for TD knee angle - Transverse Plane (180deg)

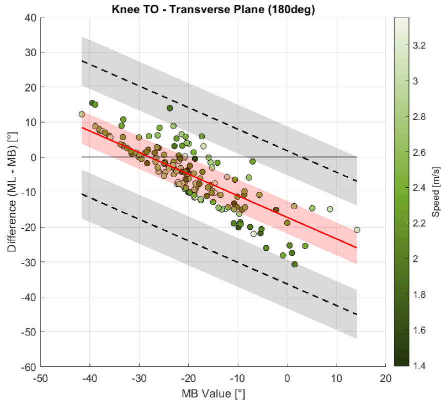

Supplementary Figure 202: Extended BA Plot for TO knee angle - Transverse Plane (180deg)

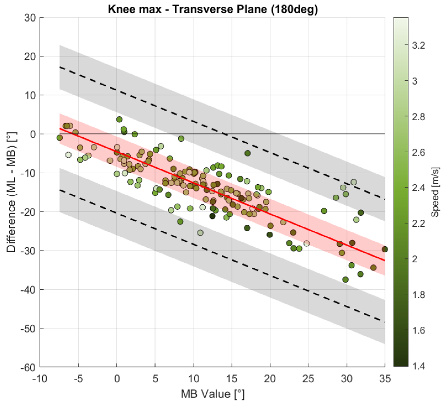

Supplementary Figure 203: Extended BA Plot for maximal knee angle - Transverse Plane (180deg)

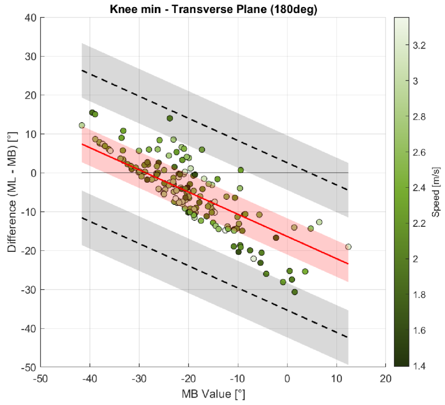

Supplementary Figure 204: Extended BA Plot for minimal knee angle - Transverse Plane (180deg)

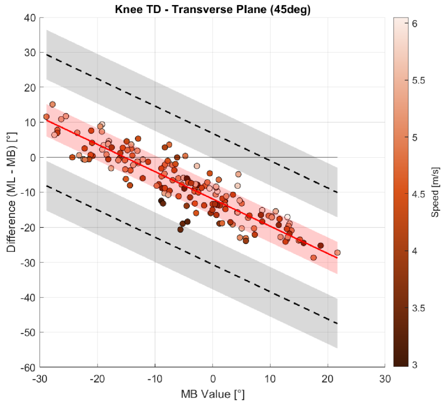

Supplementary Figure 205: Extended BA Plot for TD knee angle - Transverse Plane (45deg)

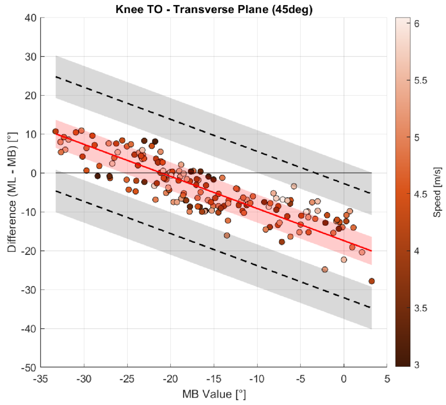

Supplementary Figure 206: Extended BA Plot for TO knee angle - Transverse Plane (45deg)

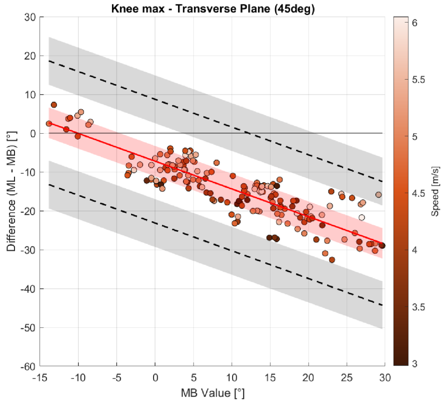

Supplementary Figure 207: Extended BA Plot for maximal knee angle - Transverse Plane (45deg)

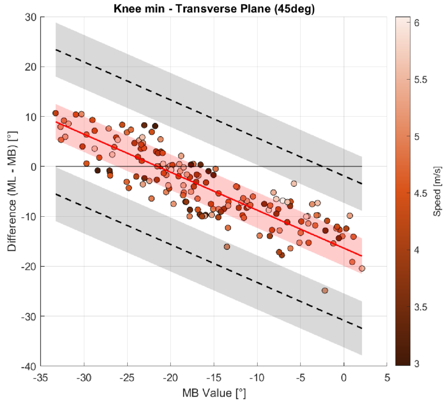

Supplementary Figure 208: Extended BA Plot for minimal knee angle - Transverse Plane (45deg)

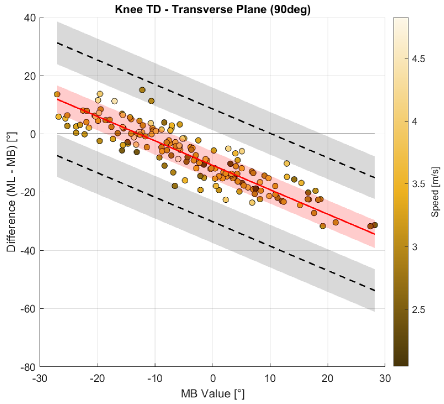

Supplementary Figure 209: Extended BA Plot for TD knee angle - Transverse Plane (90deg)

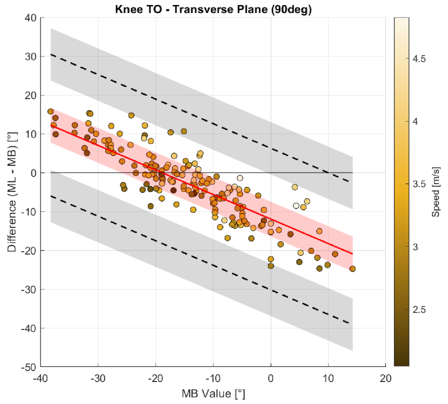

Supplementary Figure 210: Extended BA Plot for TO knee angle - Transverse Plane (90deg)

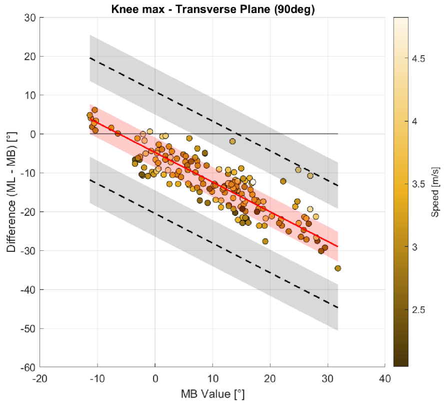

Supplementary Figure 211: Extended BA Plot for maximal knee angle - Transverse Plane (90deg)

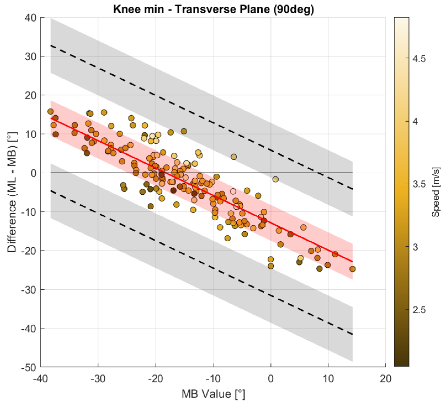

Supplementary Figure 212: Extended BA Plot for minimal knee angle - Transverse Plane (90deg)

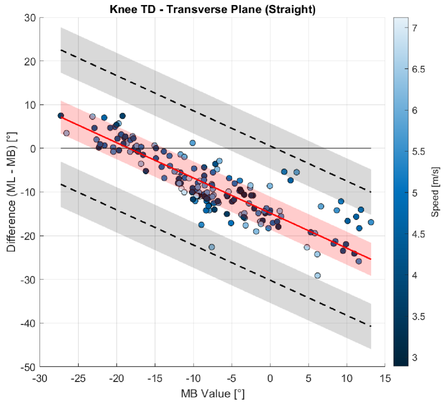

Supplementary Figure 213: Extended BA Plot for TD knee angle - Transverse Plane (Straight)

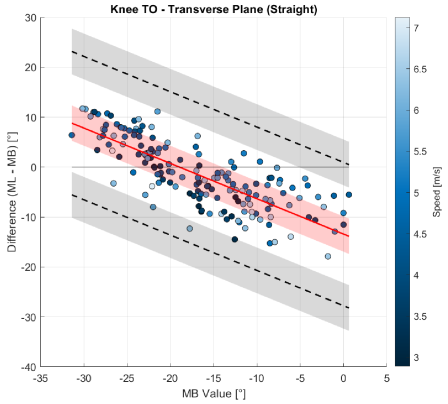

Supplementary Figure 214: Extended BA Plot for TO knee angle - Transverse Plane (Straight)

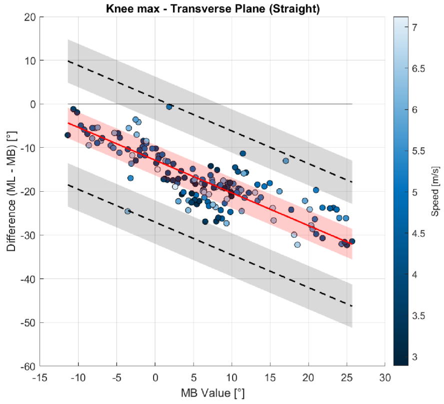

Supplementary Figure 215: Extended BA Plot for maximal knee angle - Transverse Plane (Straight)

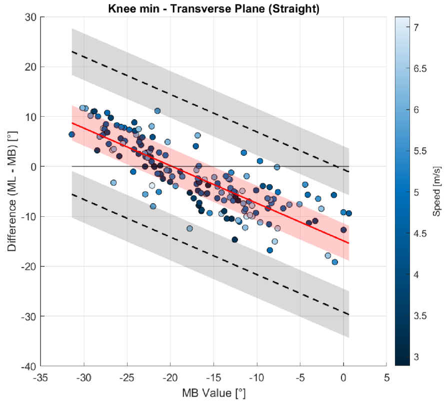

Supplementary Figure 216: Extended BA Plot for minimal knee angle - Transverse Plane (Straight)

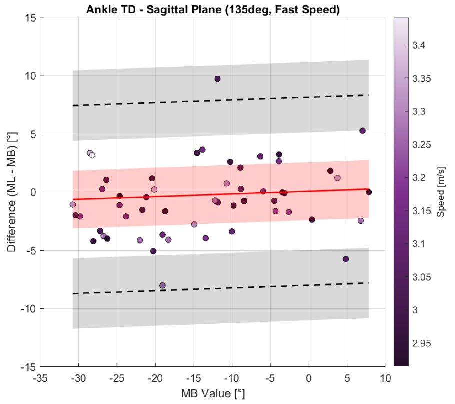

Supplementary Figure 217: Extended BA Plot for TD ankle angle - Sagittal Plane (135deg, Fast)

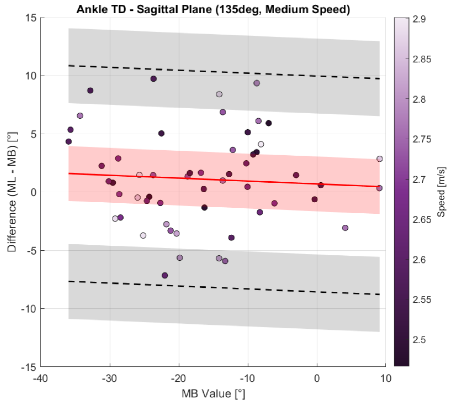

Supplementary Figure 218: Extended BA Plot for TD ankle angle - Sagittal Plane (135deg, Medium)

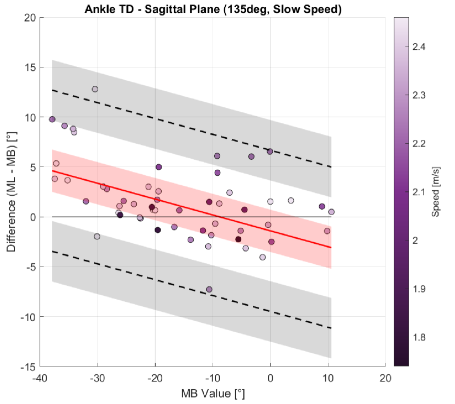

Supplementary Figure 219: Extended BA Plot for TD ankle angle - Sagittal Plane (135deg, Slow)

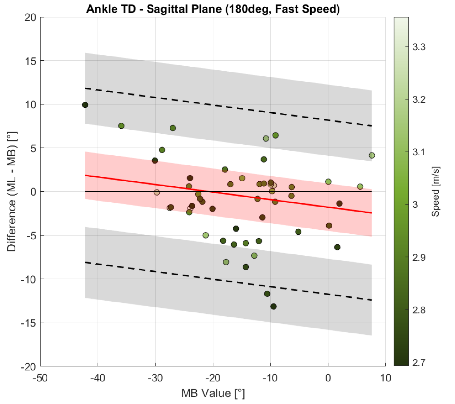

Supplementary Figure 220: Extended BA Plot for TD ankle angle - Sagittal Plane (180deg, Fast)

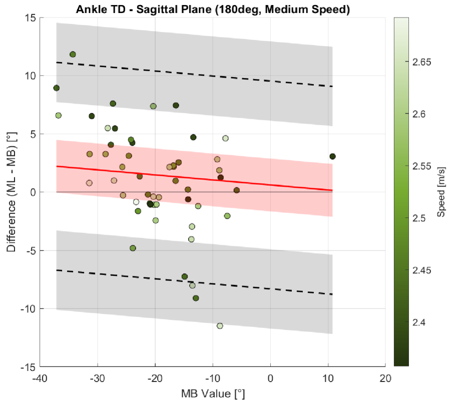

Supplementary Figure 221: Extended BA Plot for TD ankle angle - Sagittal Plane (180deg, Medium)

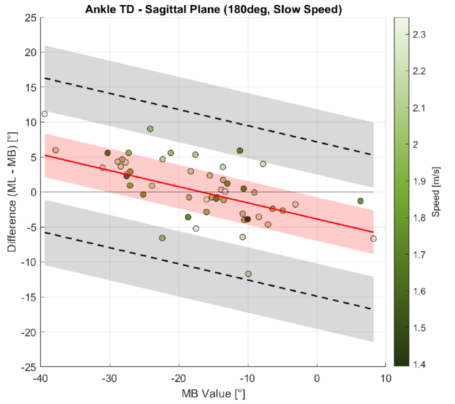

Supplementary Figure 222: Extended BA Plot for TD ankle angle - Sagittal Plane (180deg, Slow)

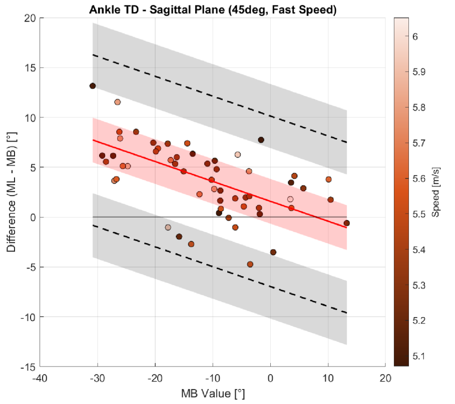

Supplementary Figure 223: Extended BA Plot for TD ankle angle - Sagittal Plane (45deg, Fast)

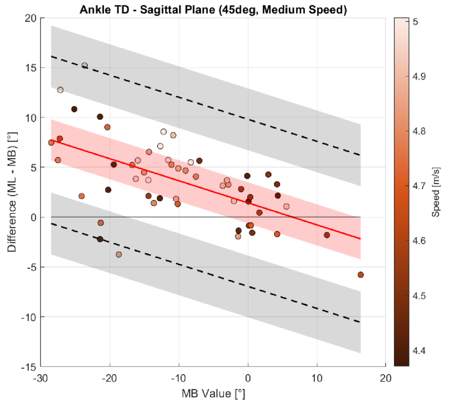

Supplementary Figure 224: Extended BA Plot for TD ankle angle - Sagittal Plane (45deg, Medium)

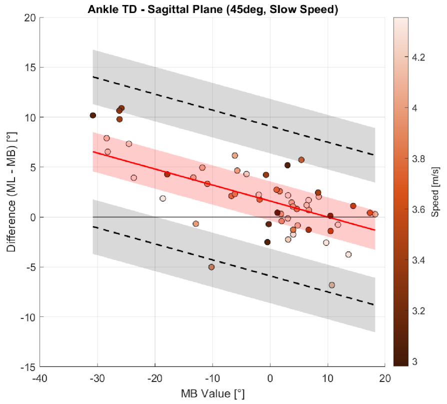

Supplementary Figure 225: Extended BA Plot for TD ankle angle - Sagittal Plane (45deg, Slow)

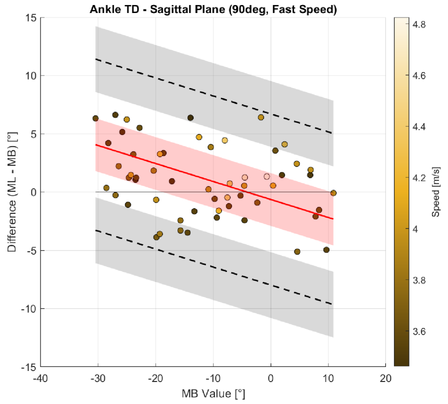

Supplementary Figure 226: Extended BA Plot for TD ankle angle - Sagittal Plane (90deg, Fast)

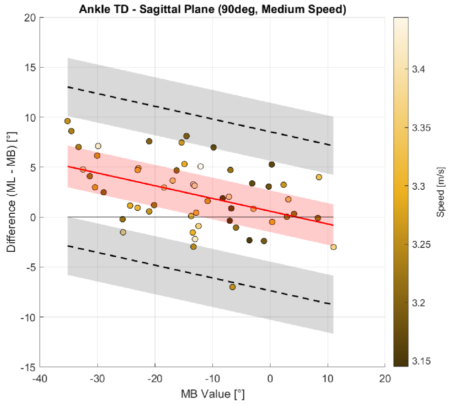

Supplementary Figure 227: Extended BA Plot for TD ankle angle - Sagittal Plane (90deg, Medium)

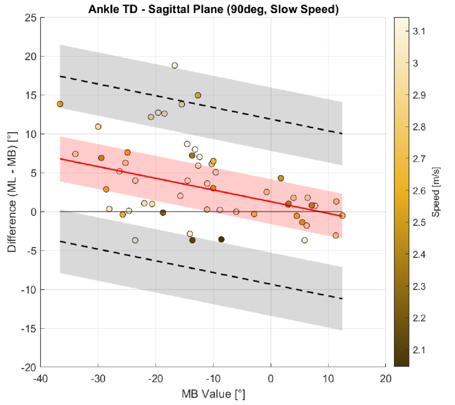

Supplementary Figure 228: Extended BA Plot for TD ankle angle - Sagittal Plane (90deg, Slow)

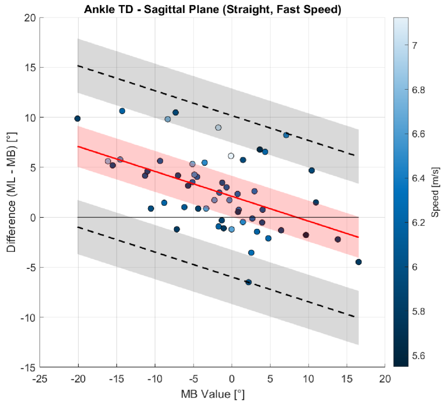

Supplementary Figure 229: Extended BA Plot for TD ankle angle - Sagittal Plane (Straight, Fast)

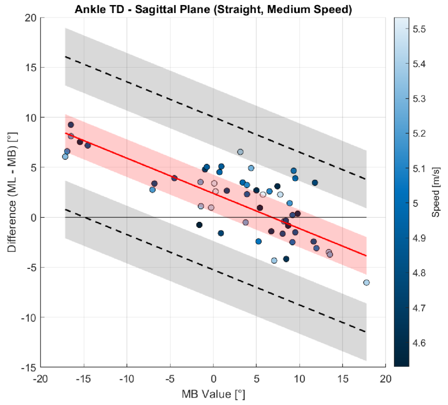

Supplementary Figure 230: Extended BA Plot for TD ankle angle - Sagittal Plane (Straight, Medium)

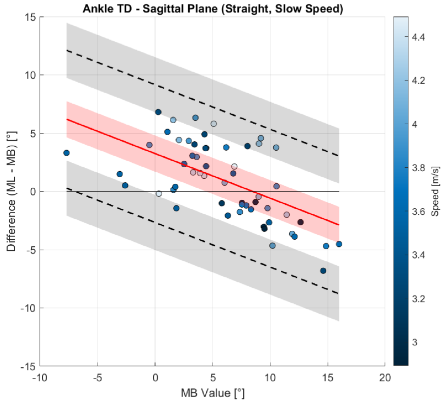

Supplementary Figure 231: Extended BA Plot for TD ankle angle - Sagittal Plane (Straight, Slow)

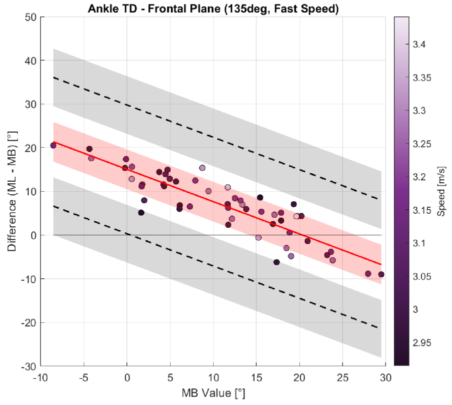

Supplementary Figure 232: Extended BA Plot for TD ankle angle - Frontal Plane (135deg, Fast)

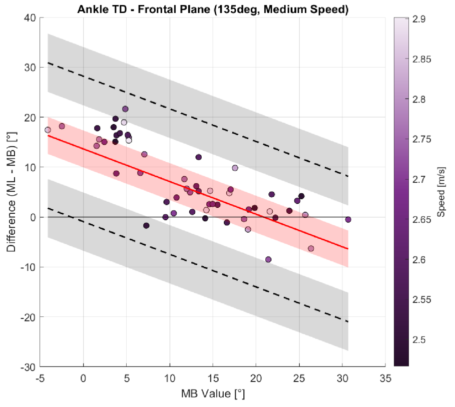

Supplementary Figure 233: Extended BA Plot for TD ankle angle - Frontal Plane (135deg, Medium)

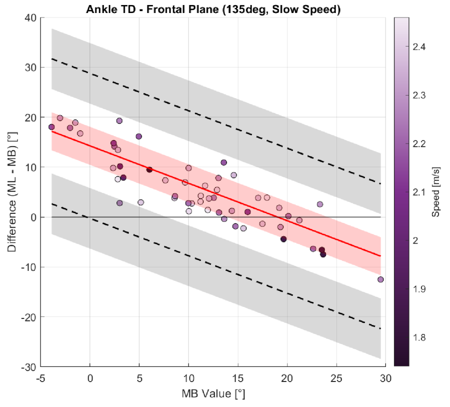

Supplementary Figure 234: Extended BA Plot for TD ankle angle - Frontal Plane (135deg, Slow)

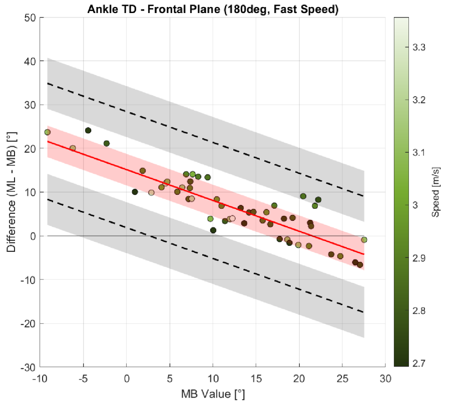

Supplementary Figure 235: Extended BA Plot for TD ankle angle - Frontal Plane (180deg, Fast)

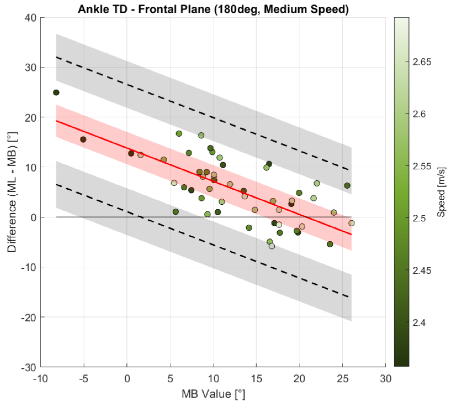

Supplementary Figure 236: Extended BA Plot for TD ankle angle - Frontal Plane (180deg, Medium)

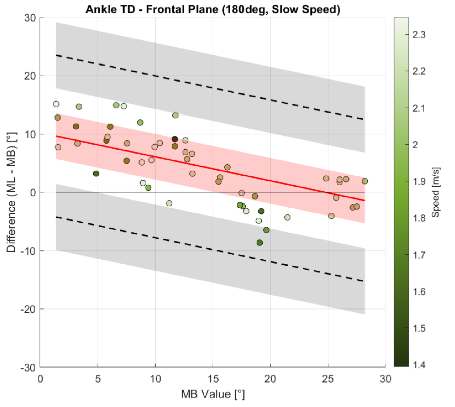

Supplementary Figure 237: Extended BA Plot for TD ankle angle - Frontal Plane (180deg, Slow)

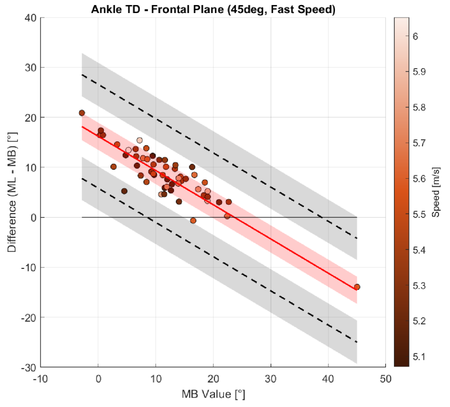

Supplementary Figure 238: Extended BA Plot for TD ankle angle - Frontal Plane (45deg, Fast)

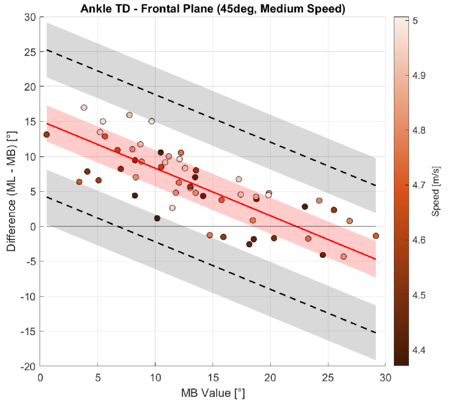

Supplementary Figure 239: Extended BA Plot for TD ankle angle - Frontal Plane (45deg, Medium)

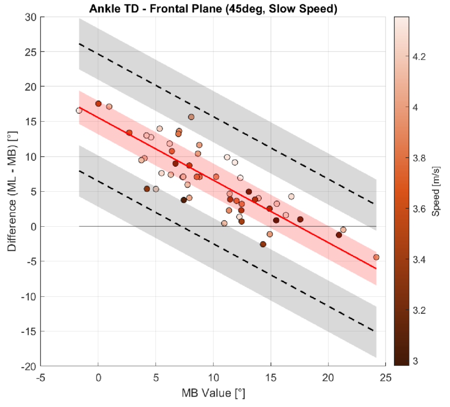

Supplementary Figure 240: Extended BA Plot for TD ankle angle - Frontal Plane (45deg, Slow)

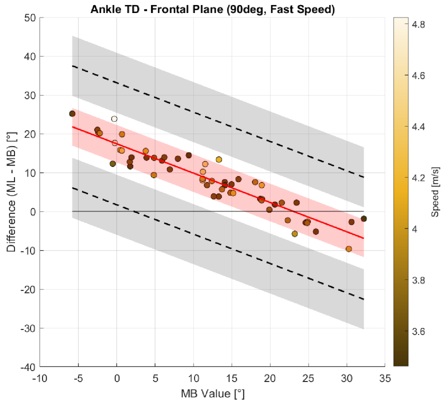

Supplementary Figure 241: Extended BA Plot for TD ankle angle - Frontal Plane (90deg, Fast)

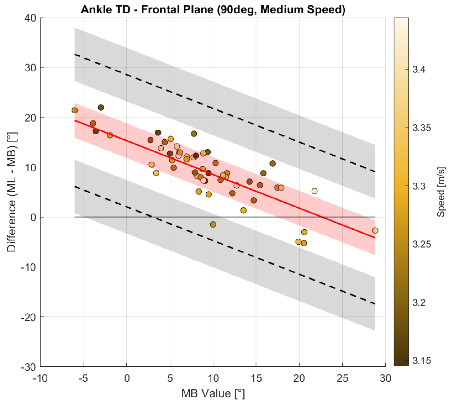

Supplementary Figure 242: Extended BA Plot for TD ankle angle - Frontal Plane (90deg, Medium)

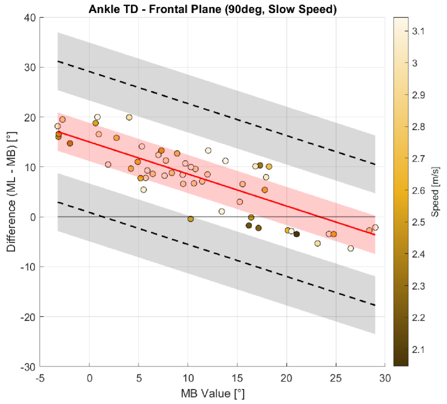

Supplementary Figure 243: Extended BA Plot for TD ankle angle - Frontal Plane (90deg, Slow)

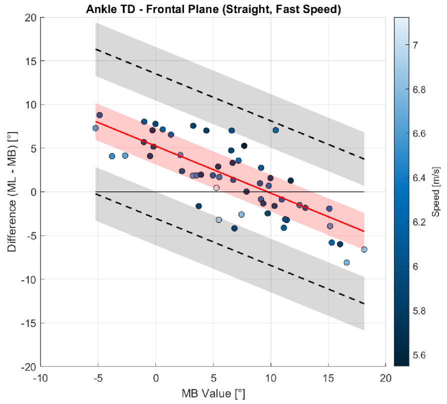

Supplementary Figure 244: Extended BA Plot for TD ankle angle - Frontal Plane (Straight, Fast)

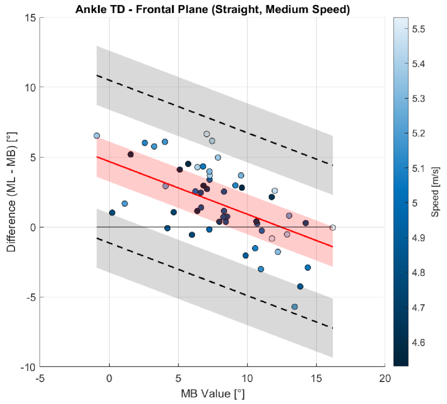

Supplementary Figure 245: Extended BA Plot for TD ankle angle - Frontal Plane (Straight, Medium)

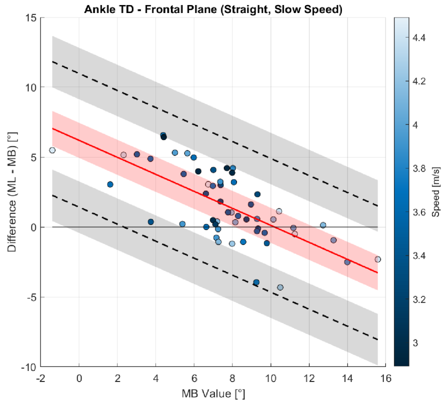

Supplementary Figure 246: Extended BA Plot for TD ankle angle - Frontal Plane (Straight, Slow)

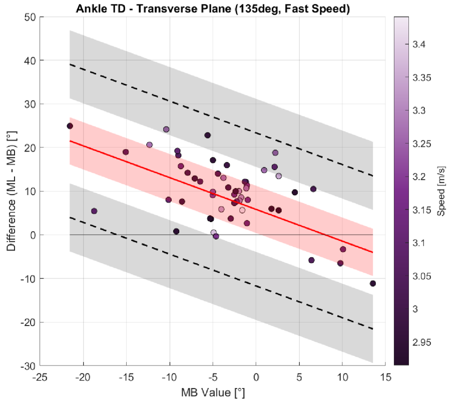

Supplementary Figure 247: Extended BA Plot for TD ankle angle - Transverse Plane (135deg, Fast)

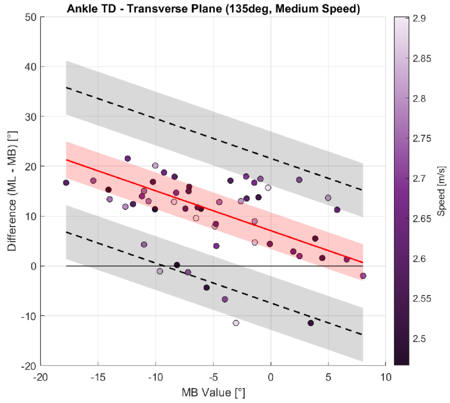

Supplementary Figure 248: Extended BA Plot for TD ankle angle - Transverse Plane (135deg, Medium)

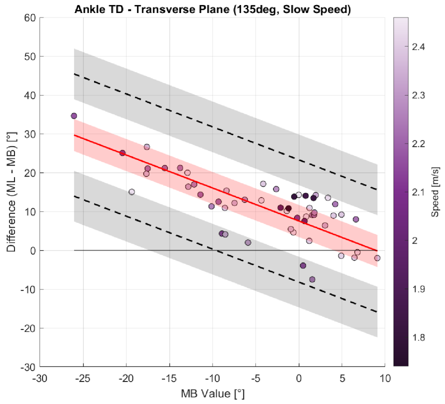

Supplementary Figure 249: Extended BA Plot for TD ankle angle - Transverse Plane (135deg, Slow)

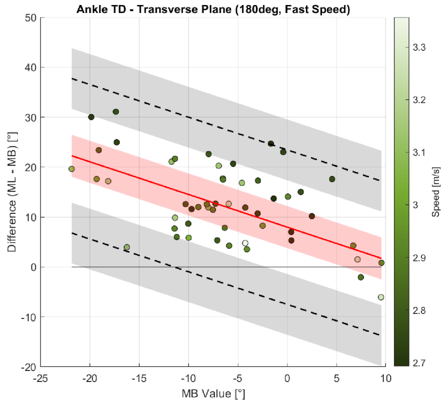

Supplementary Figure 250: Extended BA Plot for TD ankle angle - Transverse Plane (180deg, Fast)

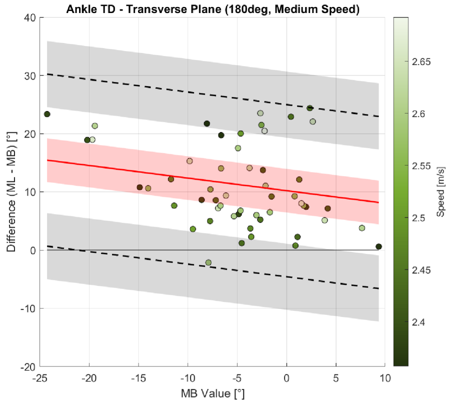

Supplementary Figure 251: Extended BA Plot for TD ankle angle - Transverse Plane (180deg, Medium)

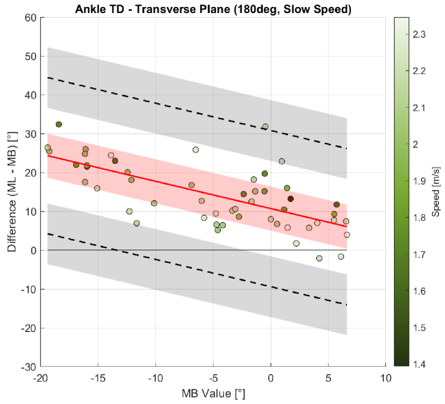

Supplementary Figure 252: Extended BA Plot for TD ankle angle - Transverse Plane (180deg, Slow)

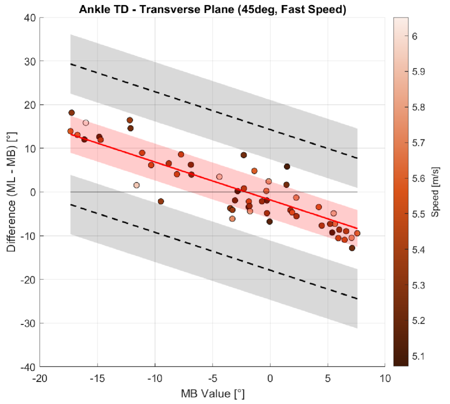

Supplementary Figure 253: Extended BA Plot for TD ankle angle - Transverse Plane (45deg, Fast)

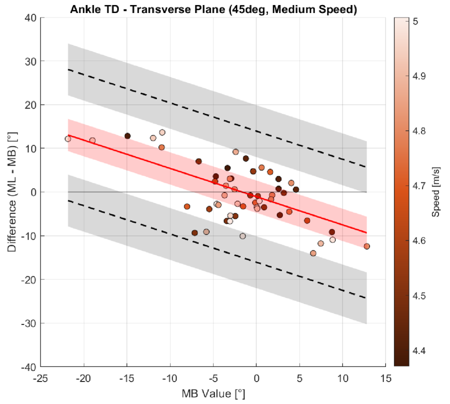

Supplementary Figure 254: Extended BA Plot for TD ankle angle - Transverse Plane (45deg, Medium)

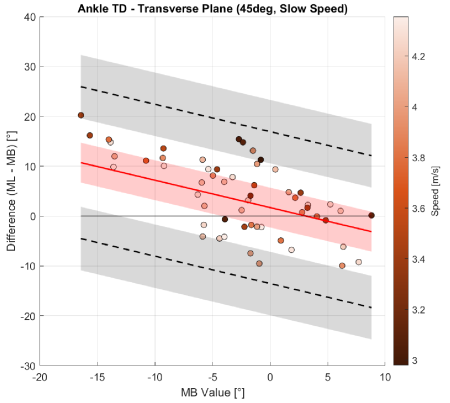

Supplementary Figure 255: Extended BA Plot for TD ankle angle - Transverse Plane (45deg, Slow)

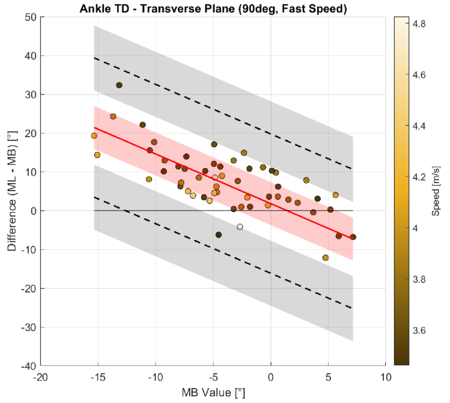

Supplementary Figure 256: Extended BA Plot for TD ankle angle - Transverse Plane (90deg, Fast)

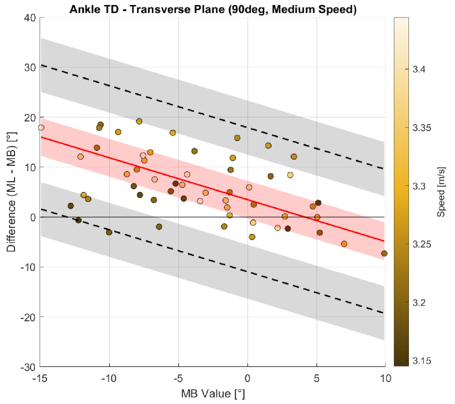

Supplementary Figure 257: Extended BA Plot for TD ankle angle - Transverse Plane (90deg, Medium)

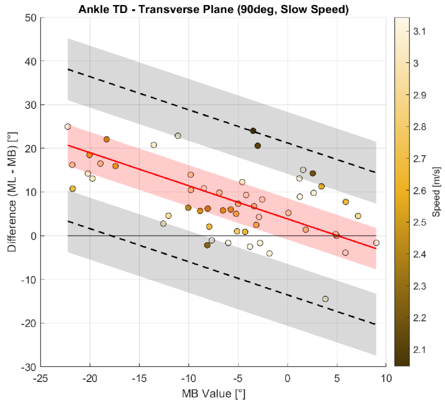

Supplementary Figure 258: Extended BA Plot for TD ankle angle - Transverse Plane (90deg, Slow)

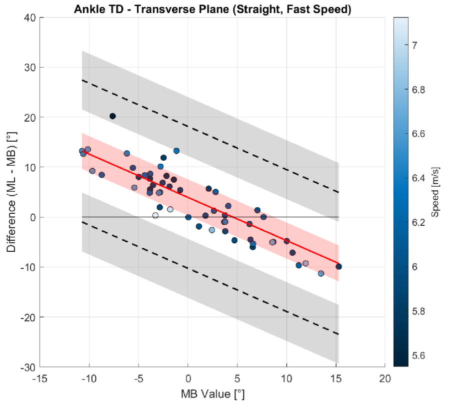

Supplementary Figure 259: Extended BA Plot for TD ankle angle - Transverse Plane (Straight, Fast)

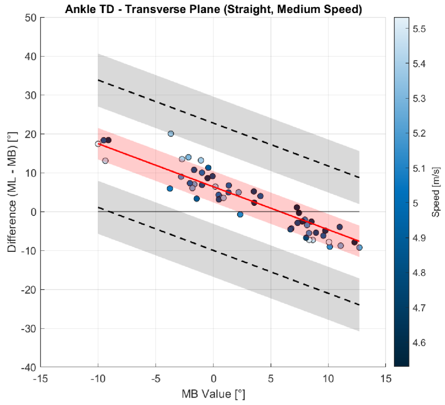

Supplementary Figure 260: Extended BA Plot for TD ankle angle - Transverse Plane (Straight, Medium)

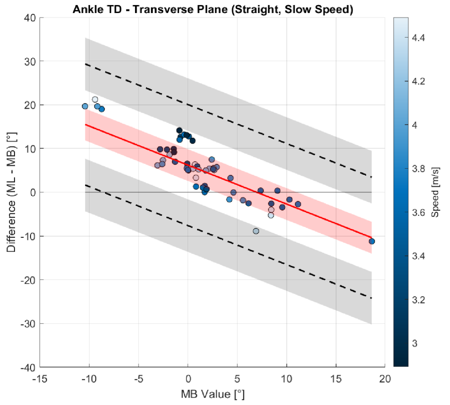

Supplementary Figure 261: Extended BA Plot for TD ankle angle - Transverse Plane (Straight, Slow)

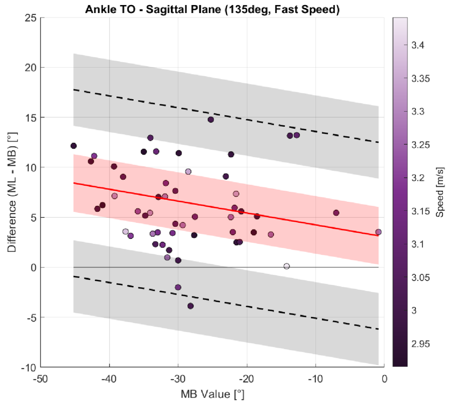

Supplementary Figure 262: Extended BA Plot for TO ankle angle - Sagittal Plane (135deg, Fast)

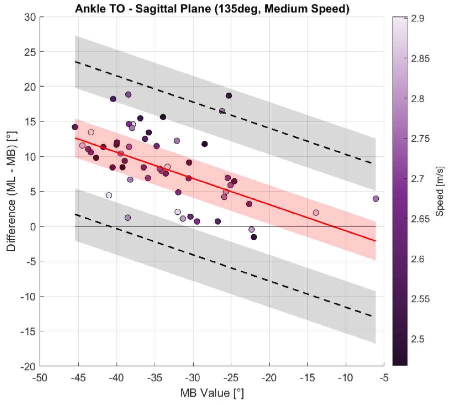

Supplementary Figure 263: Extended BA Plot for TO ankle angle - Sagittal Plane (135deg, Medium)

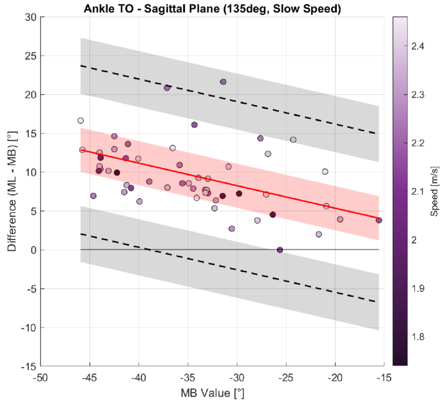

Supplementary Figure 264: Extended BA Plot for TO ankle angle - Sagittal Plane (135deg, Slow)

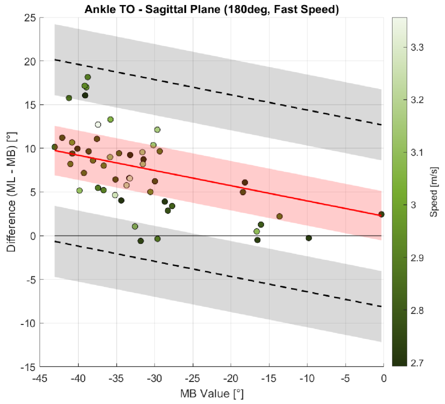

Supplementary Figure 265: Extended BA Plot for TO ankle angle - Sagittal Plane (180deg, Fast)

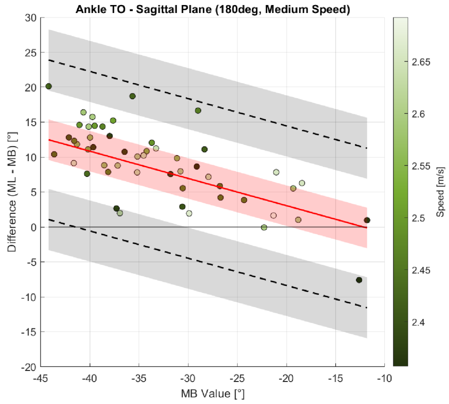

Supplementary Figure 266: Extended BA Plot for TO ankle angle - Sagittal Plane (180deg, Medium)

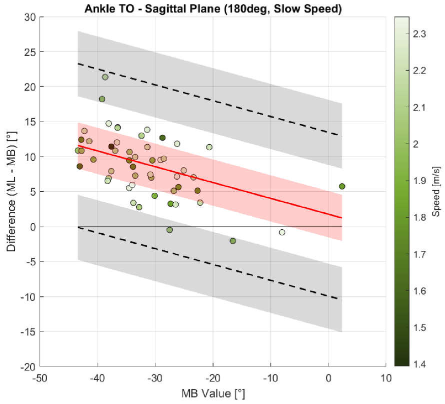

Supplementary Figure 267: Extended BA Plot for TO ankle angle - Sagittal Plane (180deg, Slow)

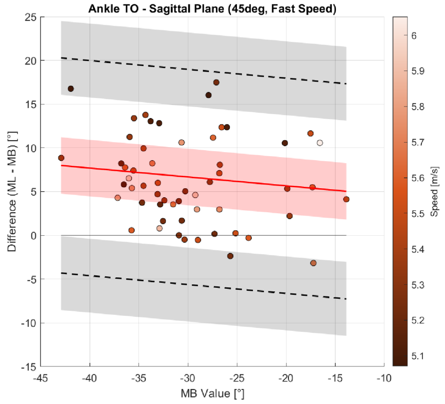

Supplementary Figure 268: Extended BA Plot for TO ankle angle - Sagittal Plane (45deg, Fast)

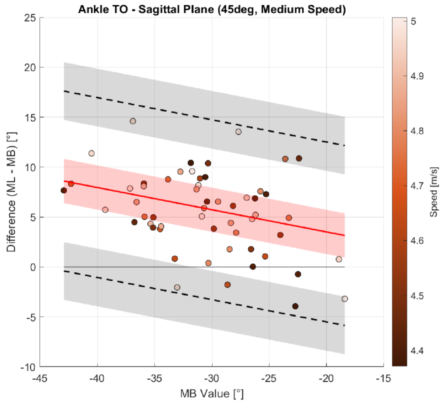

Supplementary Figure 269: Extended BA Plot for TO ankle angle - Sagittal Plane (45deg, Medium)

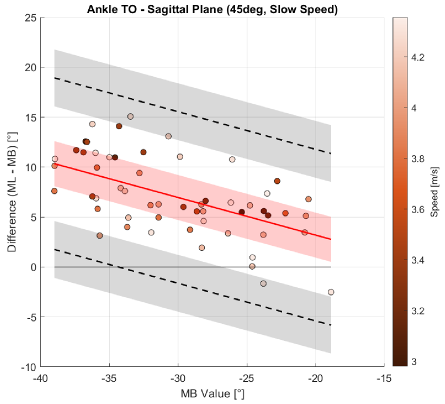

Supplementary Figure 270: Extended BA Plot for TO ankle angle - Sagittal Plane (45deg, Slow)

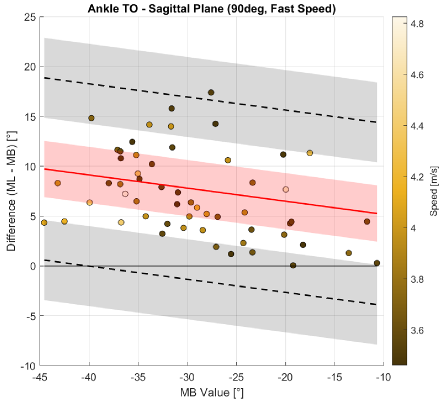

Supplementary Figure 271: Extended BA Plot for TO ankle angle - Sagittal Plane (90deg, Fast)

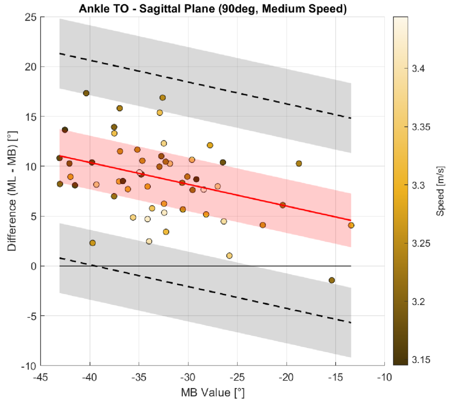

Supplementary Figure 272: Extended BA Plot for TO ankle angle - Sagittal Plane (90deg, Medium)

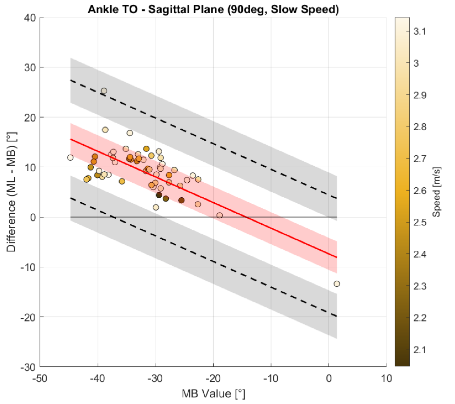

Supplementary Figure 273: Extended BA Plot for TO ankle angle - Sagittal Plane (90deg, Slow)

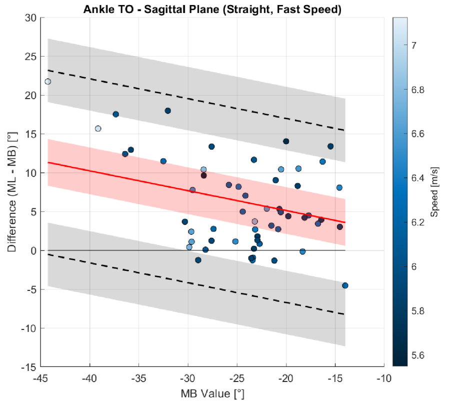

Supplementary Figure 274: Extended BA Plot for TO ankle angle - Sagittal Plane (Straight, Fast)

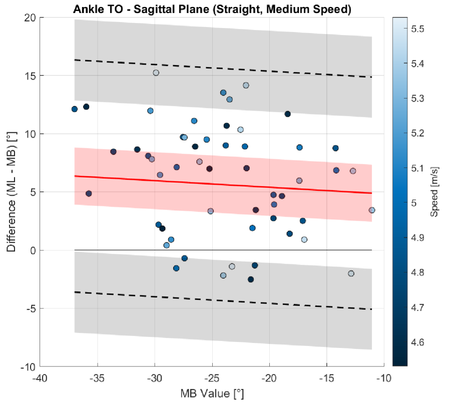

Supplementary Figure 275: Extended BA Plot for TO ankle angle - Sagittal Plane (Straight, Medium)

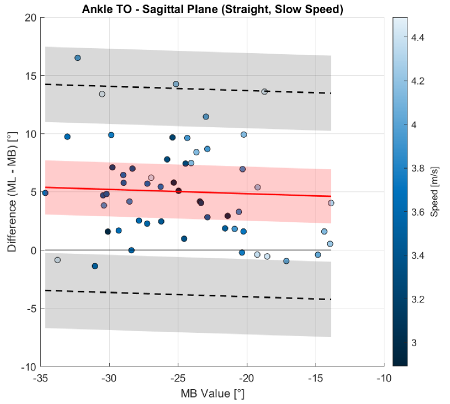

Supplementary Figure 276: Extended BA Plot for TO ankle angle - Sagittal Plane (Straight, Slow)

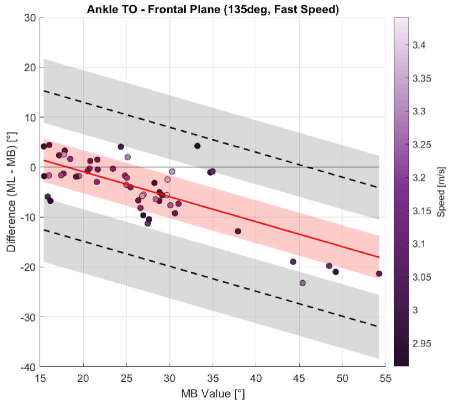

Supplementary Figure 277: Extended BA Plot for TO ankle angle - Frontal Plane (135deg, Fast)

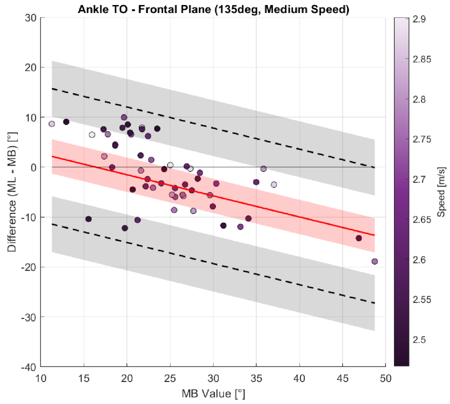

Supplementary Figure 278: Extended BA Plot for TO ankle angle - Frontal Plane (135deg, Medium)

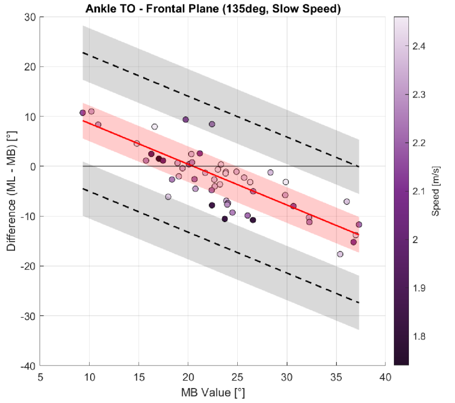

Supplementary Figure 279: Extended BA Plot for TO ankle angle - Frontal Plane (135deg, Slow)

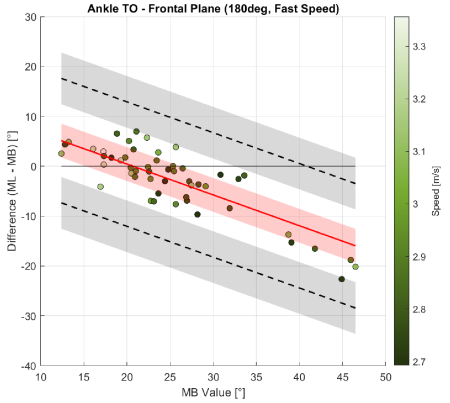

Supplementary Figure 280: Extended BA Plot for TO ankle angle - Frontal Plane (180deg, Fast)

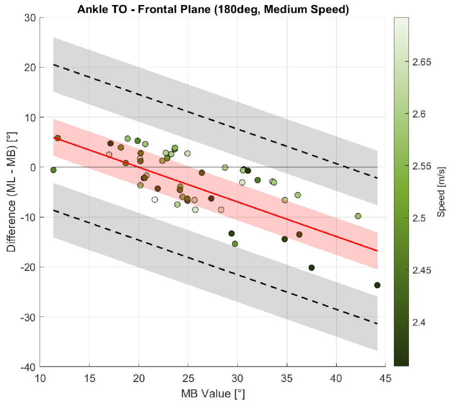

Supplementary Figure 281: Extended BA Plot for TO ankle angle - Frontal Plane (180deg, Medium)

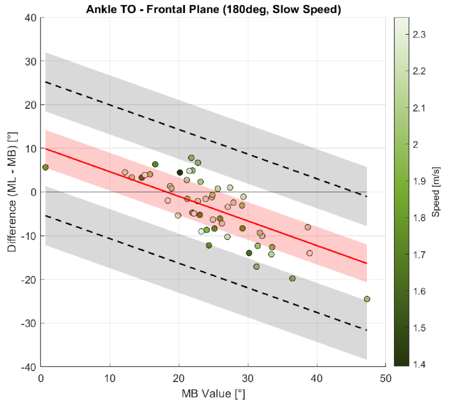

Supplementary Figure 282: Extended BA Plot for TO ankle angle - Frontal Plane (180deg, Slow)

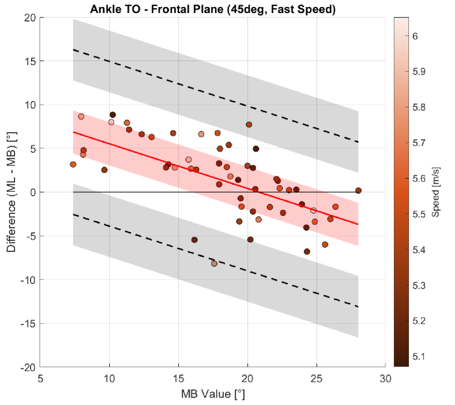

Supplementary Figure 283: Extended BA Plot for TO ankle angle - Frontal Plane (45deg, Fast)

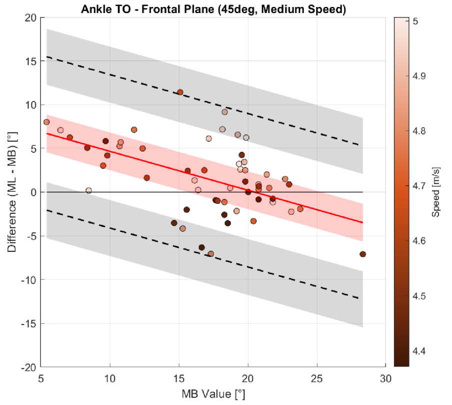

Supplementary Figure 284: Extended BA Plot for TO ankle angle - Frontal Plane (45deg, Medium)

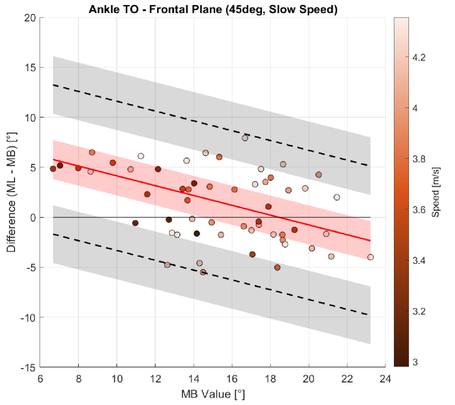

Supplementary Figure 285: Extended BA Plot for TO ankle angle - Frontal Plane (45deg, Slow)

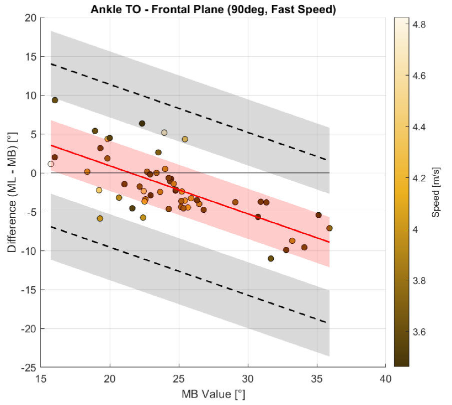

Supplementary Figure 286: Extended BA Plot for TO ankle angle - Frontal Plane (90deg, Fast)

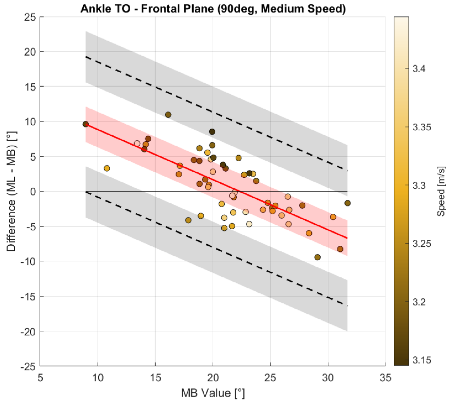

Supplementary Figure 287: Extended BA Plot for TO ankle angle - Frontal Plane (90deg, Medium)

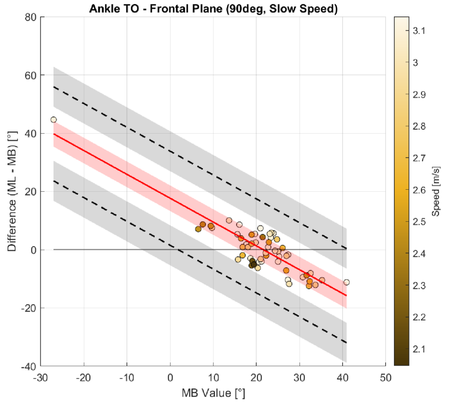

Supplementary Figure 288: Extended BA Plot for TO ankle angle - Frontal Plane (90deg, Slow)

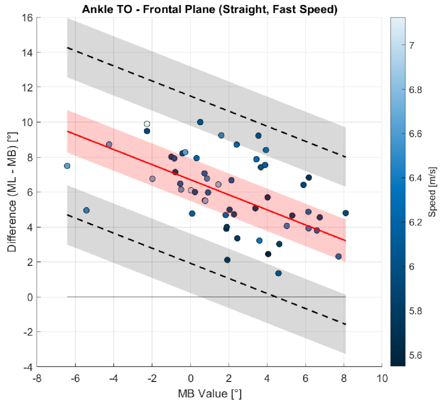

Supplementary Figure 289: Extended BA Plot for TO ankle angle - Frontal Plane (Straight, Fast)

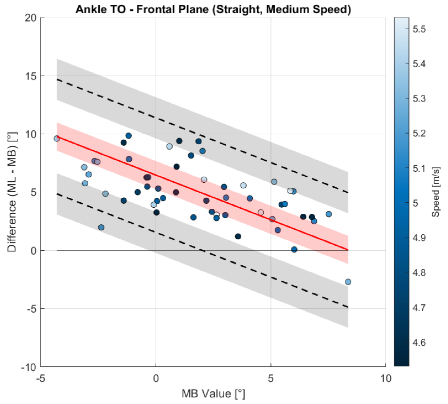

Supplementary Figure 290: Extended BA Plot for TO ankle angle - Frontal Plane (Straight, Medium)

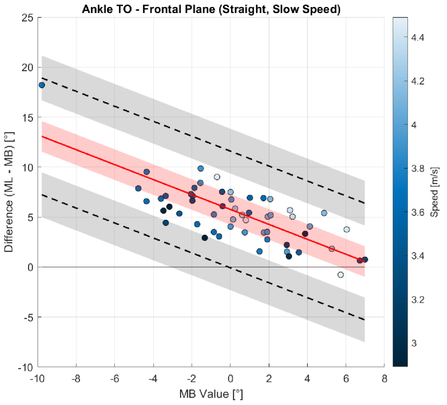

Supplementary Figure 291: Extended BA Plot for TO ankle angle - Frontal Plane (Straight, Slow)

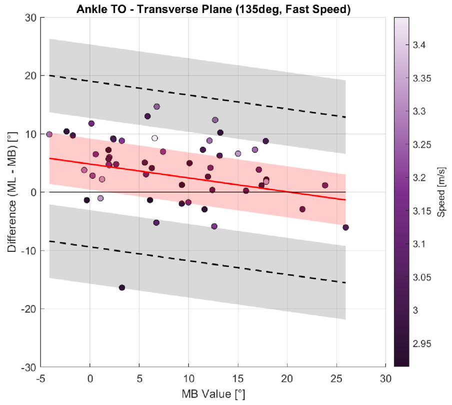

Supplementary Figure 292: Extended BA Plot for TO ankle angle - Transverse Plane (135deg, Fast)

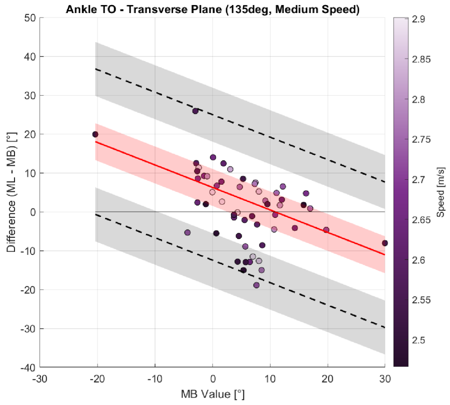

Supplementary Figure 293: Extended BA Plot for TO ankle angle - Transverse Plane (135deg, Medium)

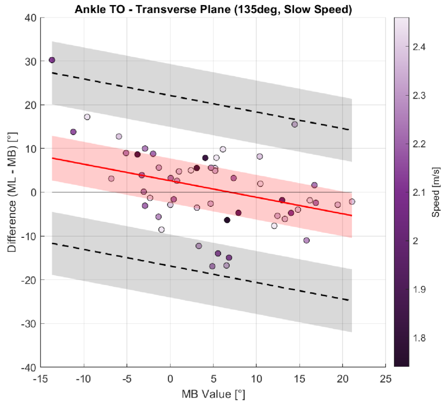

Supplementary Figure 294: Extended BA Plot for TO ankle angle - Transverse Plane (135deg, Slow)

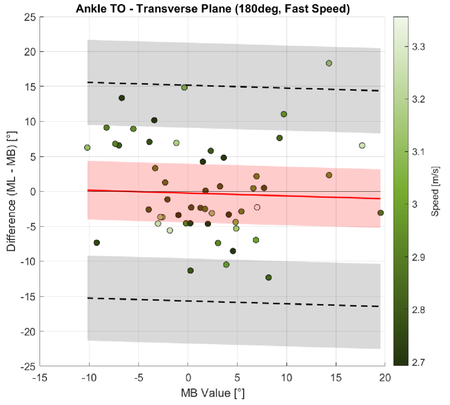

Supplementary Figure 295: Extended BA Plot for TO ankle angle - Transverse Plane (180deg, Fast)

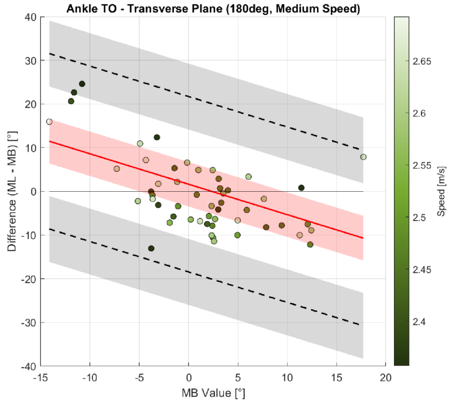

Supplementary Figure 296: Extended BA Plot for TO ankle angle - Transverse Plane (180deg, Medium)

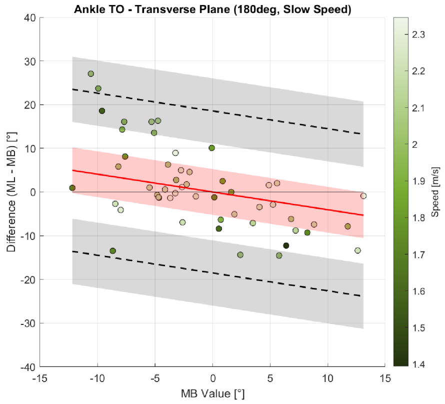

Supplementary Figure 297: Extended BA Plot for TO ankle angle - Transverse Plane (180deg, Slow)

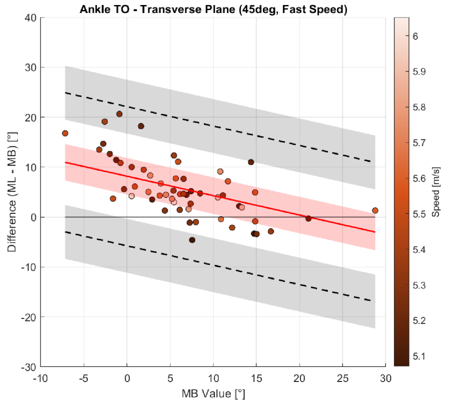

Supplementary Figure 298: Extended BA Plot for TO ankle angle - Transverse Plane (45deg, Fast)

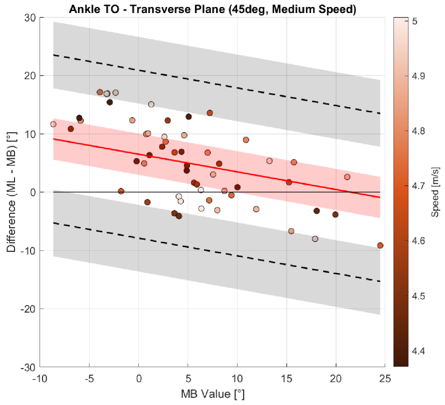

Supplementary Figure 299: Extended BA Plot for TO ankle angle - Transverse Plane (45deg, Medium)

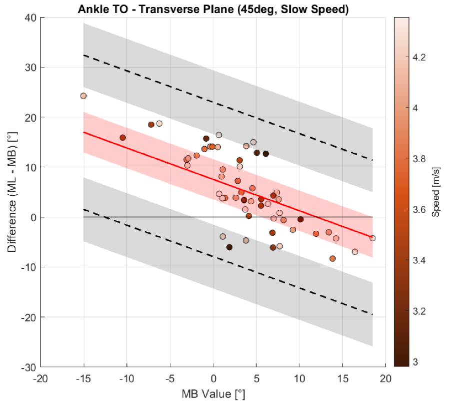

Supplementary Figure 300: Extended BA Plot for TO ankle angle - Transverse Plane (45deg, Slow)

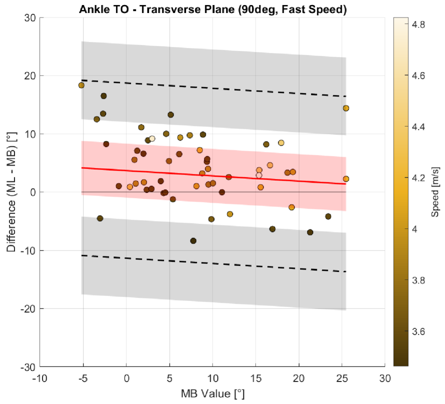

Supplementary Figure 301: Extended BA Plot for TO ankle angle - Transverse Plane (90deg, Fast)

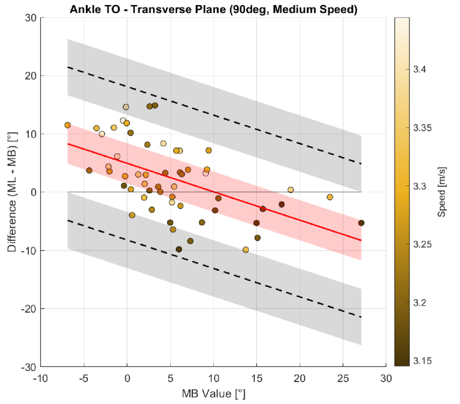

Supplementary Figure 302: Extended BA Plot for TO ankle angle - Transverse Plane (90deg, Medium)

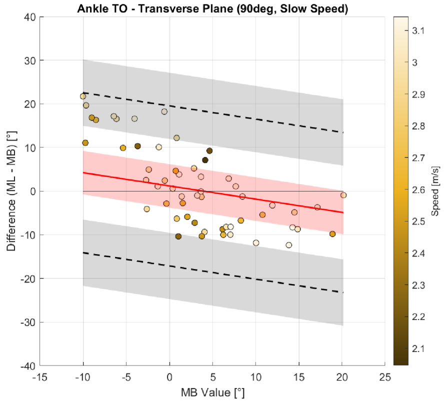

Supplementary Figure 303: Extended BA Plot for TO ankle angle - Transverse Plane (90deg, Slow)

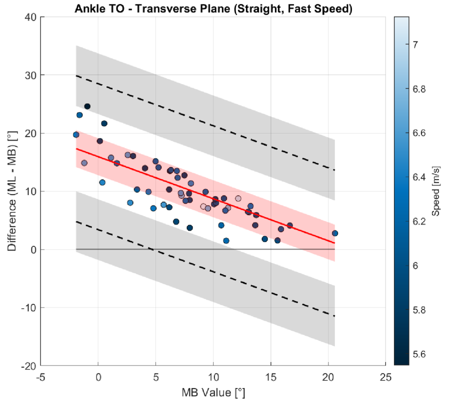

Supplementary Figure 304: Extended BA Plot for TO ankle angle - Transverse Plane (Straight, Fast)

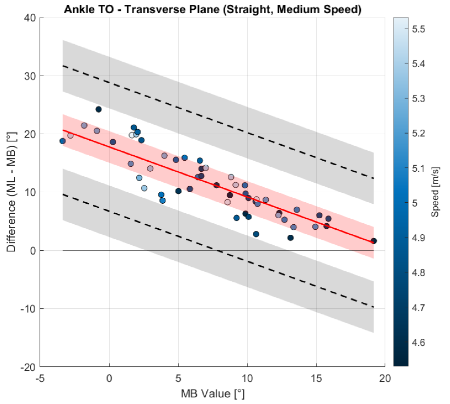

Supplementary Figure 305: Extended BA Plot for TO ankle angle - Transverse Plane (Straight, Medium)

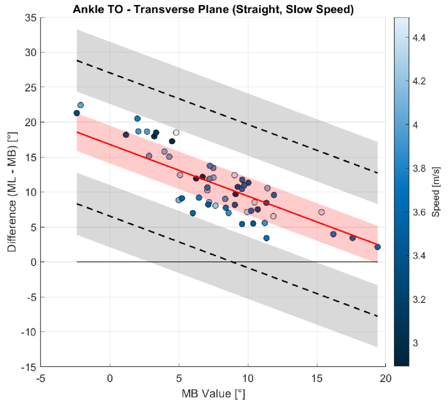

Supplementary Figure 306: Extended BA Plot for TO ankle angle - Transverse Plane (Straight, Slow)

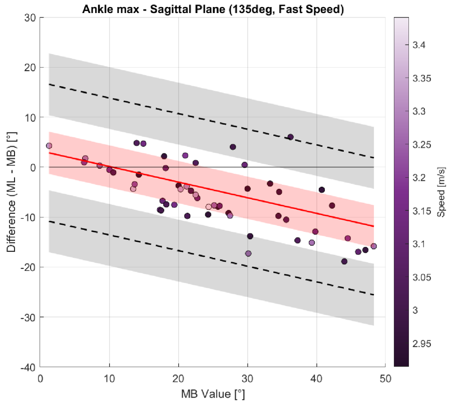

Supplementary Figure 307: Extended BA Plot for maximal ankle angle - Sagittal Plane (135deg, Fast)

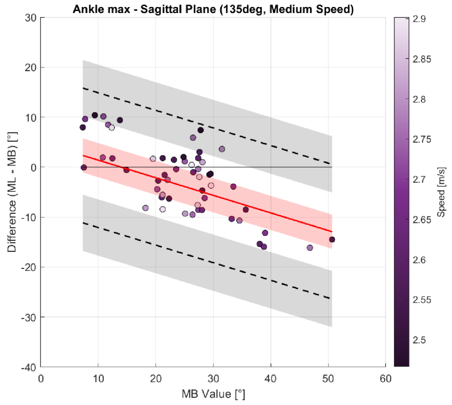

Supplementary Figure 308: Extended BA Plot for maximal ankle angle - Sagittal Plane (135deg, Medium)

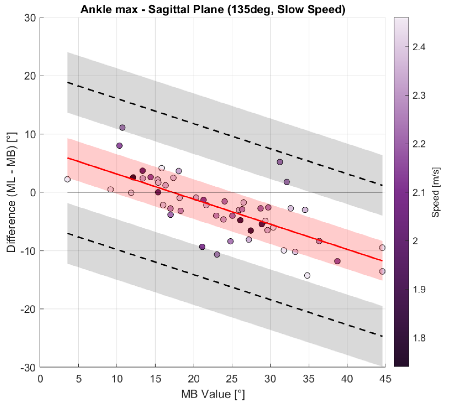

Supplementary Figure 309: Extended BA Plot for maximal ankle angle - Sagittal Plane (135deg, Slow)

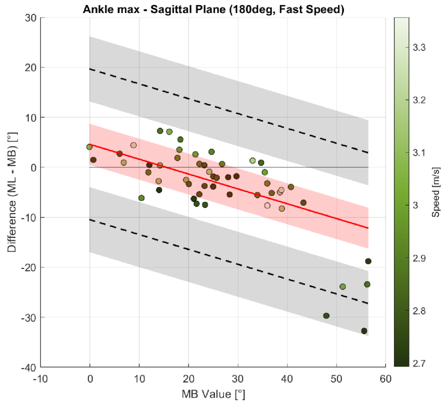

Supplementary Figure 310: Extended BA Plot for maximal ankle angle - Sagittal Plane (180deg, Fast)

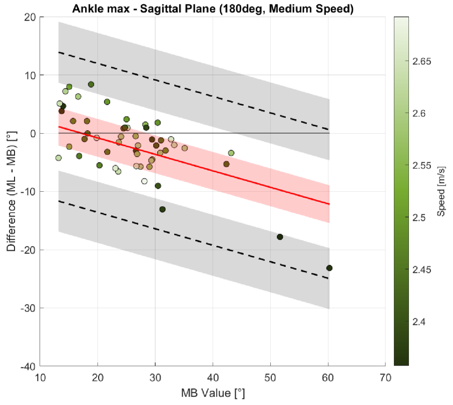

Supplementary Figure 311: Extended BA Plot for maximal ankle angle - Sagittal Plane (180deg, Medium)

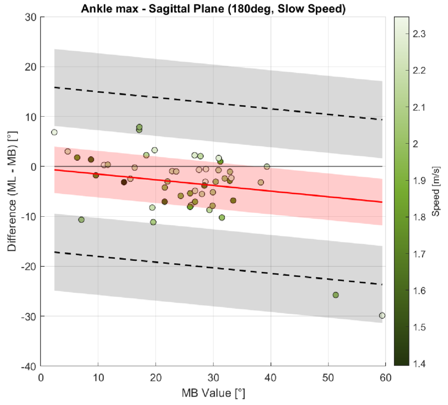

Supplementary Figure 312: Extended BA Plot for maximal ankle angle - Sagittal Plane (180deg, Slow)

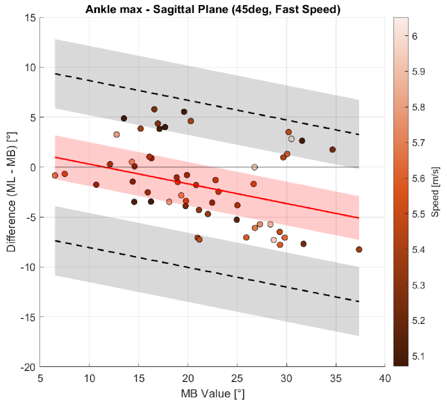

Supplementary Figure 313: Extended BA Plot for maximal ankle angle - Sagittal Plane (45deg, Fast)

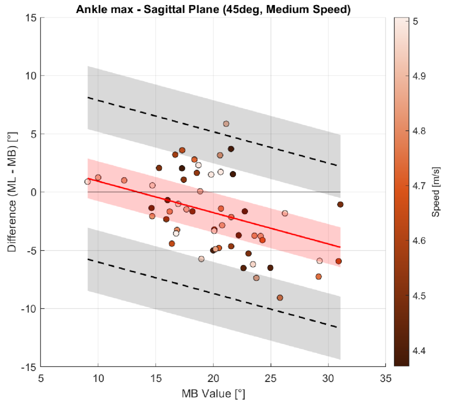

Supplementary Figure 314: Extended BA Plot for maximal ankle angle - Sagittal Plane (45deg, Medium)

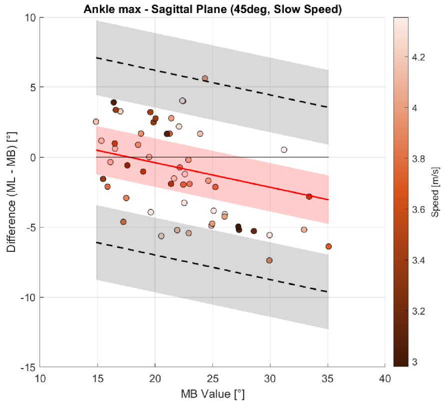

Supplementary Figure 315: Extended BA Plot for maximal ankle angle - Sagittal Plane (45deg, Slow)

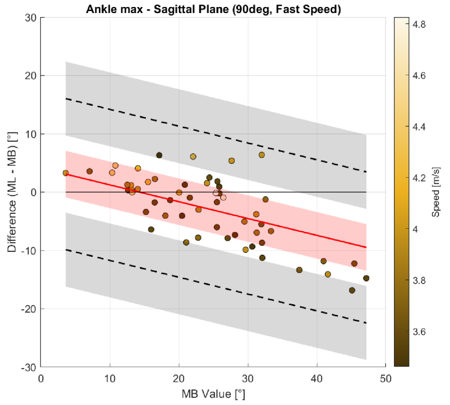

Supplementary Figure 316: Extended BA Plot for maximal ankle angle - Sagittal Plane (90deg, Fast)

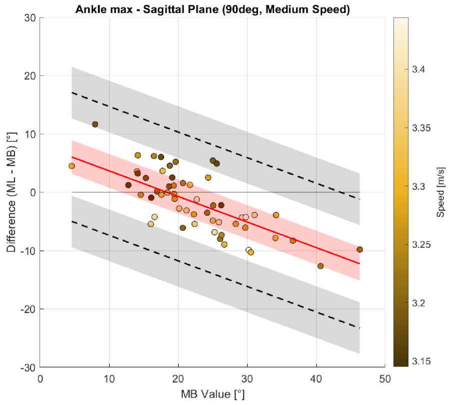

Supplementary Figure 317: Extended BA Plot for maximal ankle angle - Sagittal Plane (90deg, Medium)

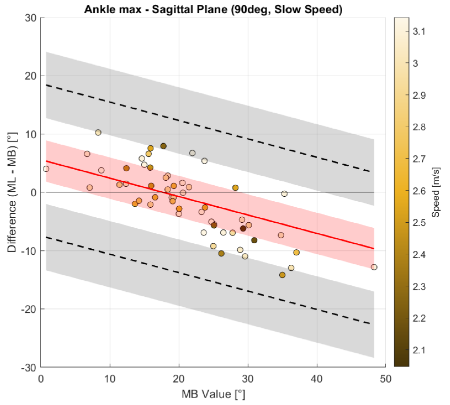

Supplementary Figure 318: Extended BA Plot for maximal ankle angle - Sagittal Plane (90deg, Slow)

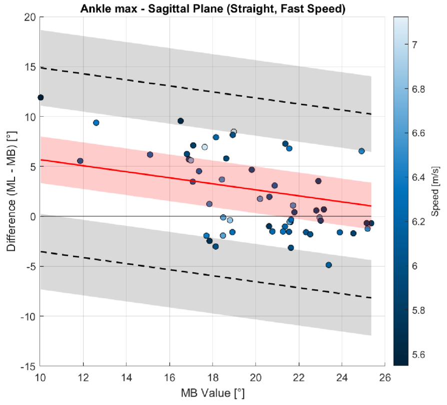

Supplementary Figure 319: Extended BA Plot for maximal ankle angle - Sagittal Plane (Straight, Fast)

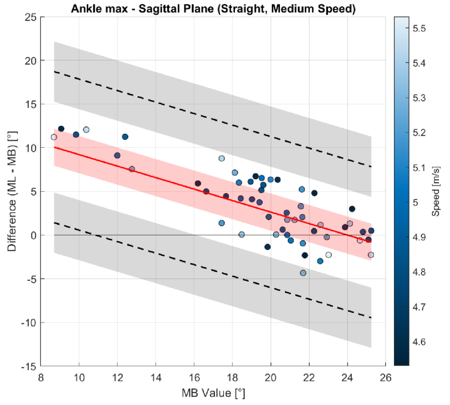

Supplementary Figure 320: Extended BA Plot for maximal ankle angle - Sagittal Plane (Straight, Medium)

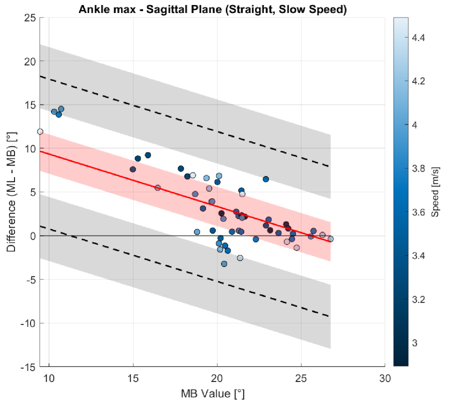

Supplementary Figure 321: Extended BA Plot for maximal ankle angle - Sagittal Plane (Straight, Slow)

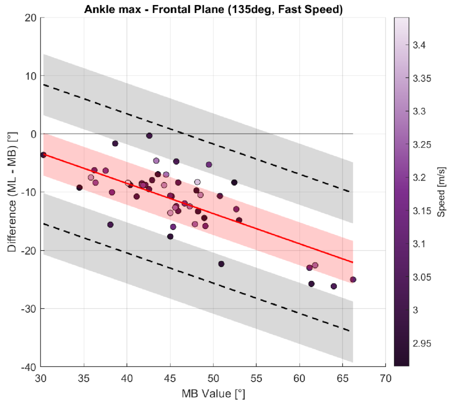

Supplementary Figure 322: Extended BA Plot for maximal ankle angle - Frontal Plane (135deg, Fast)

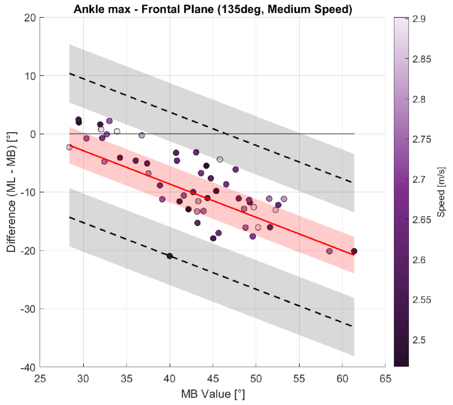

Supplementary Figure 323: Extended BA Plot for maximal ankle angle - Frontal Plane (135deg, Medium)

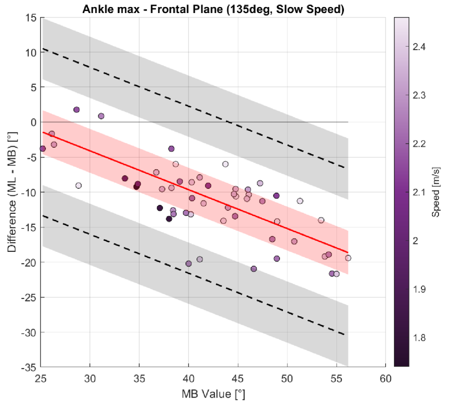

Supplementary Figure 324: Extended BA Plot for maximal ankle angle - Frontal Plane (135deg, Slow)

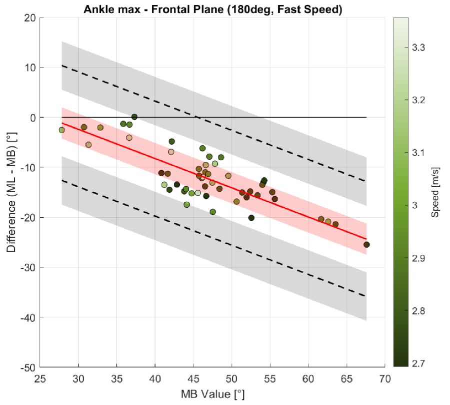

Supplementary Figure 325: Extended BA Plot for maximal ankle angle - Frontal Plane (180deg, Fast)

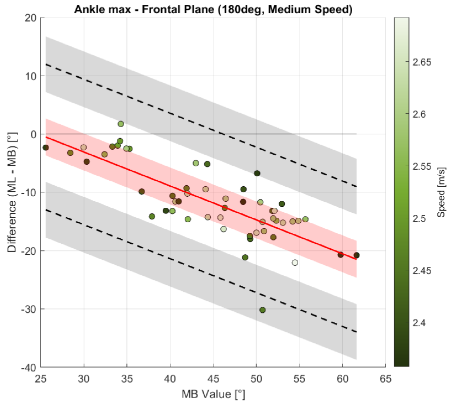

Supplementary Figure 326: Extended BA Plot for maximal ankle angle - Frontal Plane (180deg, Medium)

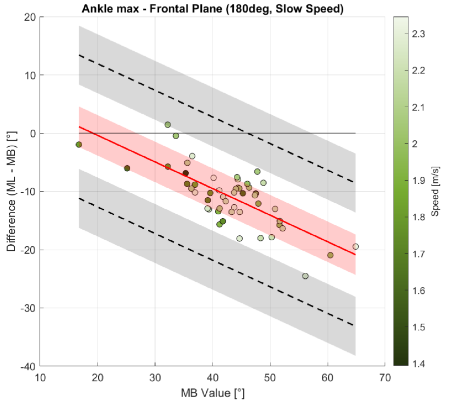

Supplementary Figure 327: Extended BA Plot for maximal ankle angle - Frontal Plane (180deg, Slow)

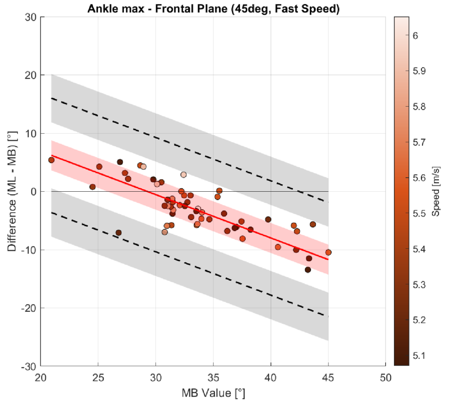

Supplementary Figure 328: Extended BA Plot for maximal ankle angle - Frontal Plane (45deg, Fast)

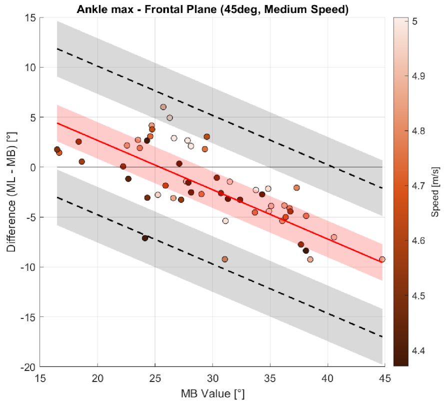

Supplementary Figure 329: Extended BA Plot for maximal ankle angle - Frontal Plane (45deg, Medium)

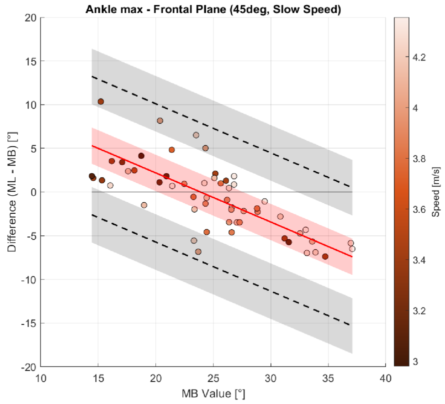

Supplementary Figure 330: Extended BA Plot for maximal ankle angle - Frontal Plane (45deg, Slow)

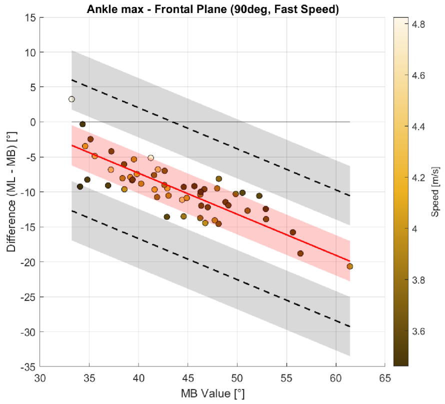

Supplementary Figure 331: Extended BA Plot for maximal ankle angle - Frontal Plane (90deg, Fast)

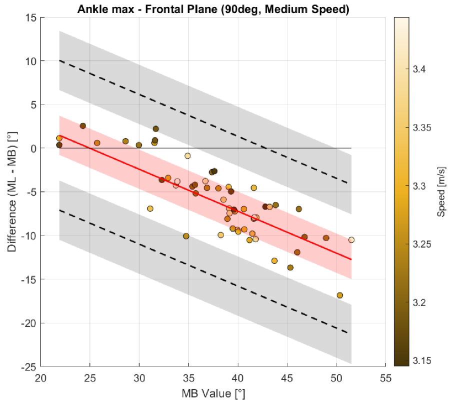

Supplementary Figure 332: Extended BA Plot for maximal ankle angle - Frontal Plane (90deg, Medium)

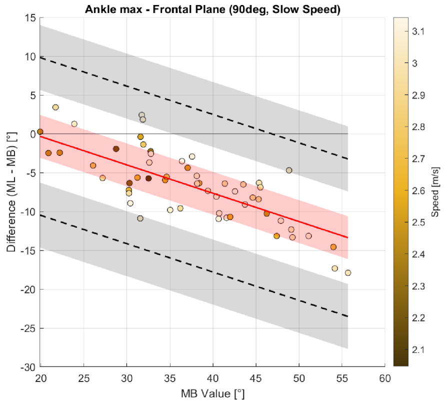

Supplementary Figure 333: Extended BA Plot for maximal ankle angle - Frontal Plane (90deg, Slow)

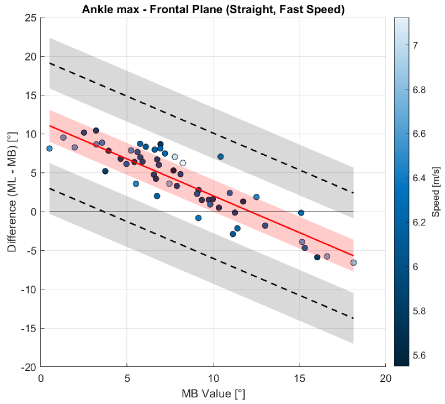

Supplementary Figure 334: Extended BA Plot for maximal ankle angle - Frontal Plane (Straight, Fast)

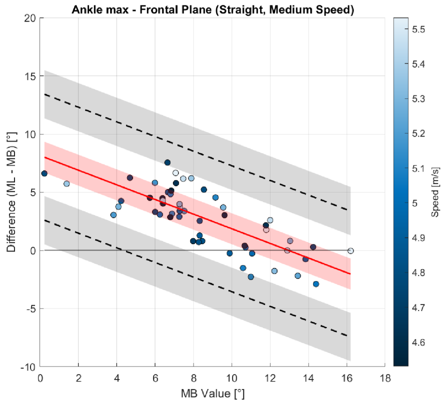

Supplementary Figure 335: Extended BA Plot for maximal ankle angle - Frontal Plane (Straight, Medium)

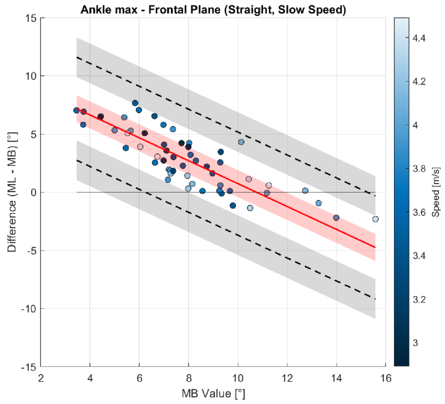

Supplementary Figure 336: Extended BA Plot for maximal ankle angle - Frontal Plane (Straight, Slow)

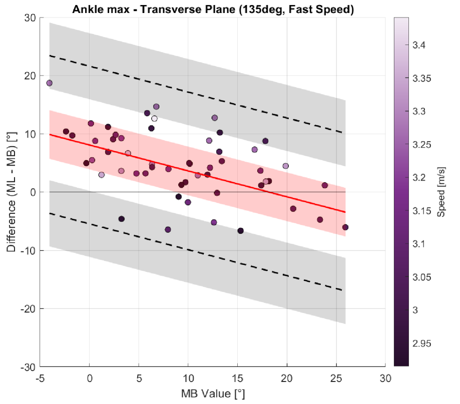

Supplementary Figure 337: Extended BA Plot for maximal ankle angle - Transverse Plane (135deg, Fast)

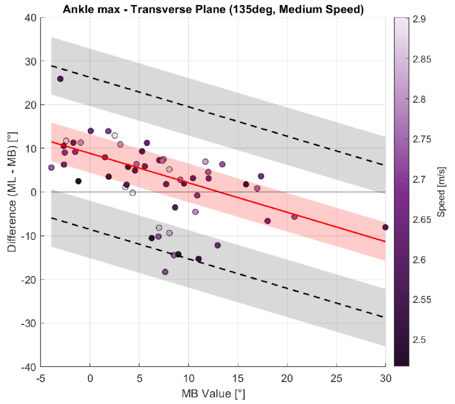

Supplementary Figure 338: Extended BA Plot for maximal ankle angle - Transverse Plane (135deg, Medium)

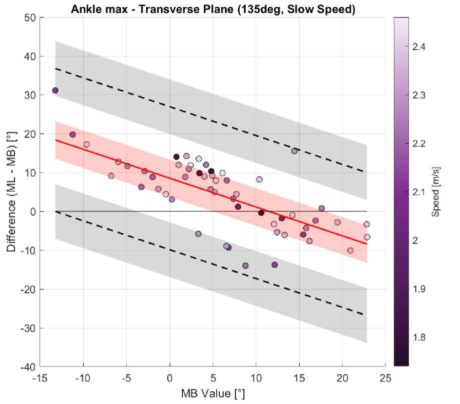

Supplementary Figure 339: Extended BA Plot for maximal ankle angle - Transverse Plane (135deg, Slow)

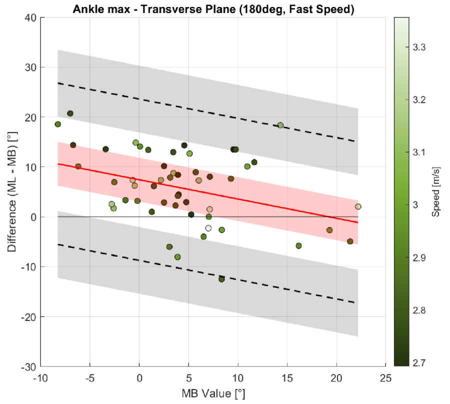

Supplementary Figure 340: Extended BA Plot for maximal ankle angle - Transverse Plane (180deg, Fast)

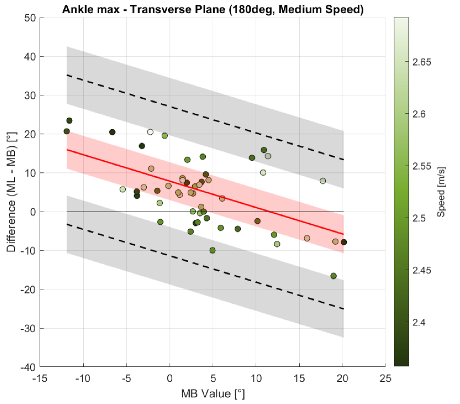

Supplementary Figure 341: Extended BA Plot for maximal ankle angle - Transverse Plane (180deg, Medium)

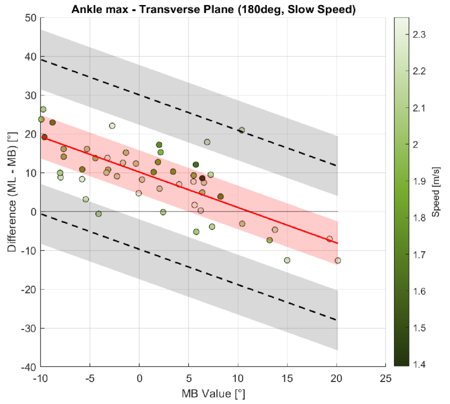

Supplementary Figure 342: Extended BA Plot for maximal ankle angle - Transverse Plane (180deg, Slow)

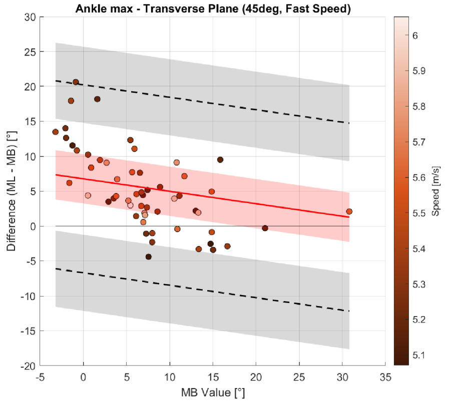

Supplementary Figure 343: Extended BA Plot for maximal ankle angle - Transverse Plane (45deg, Fast)

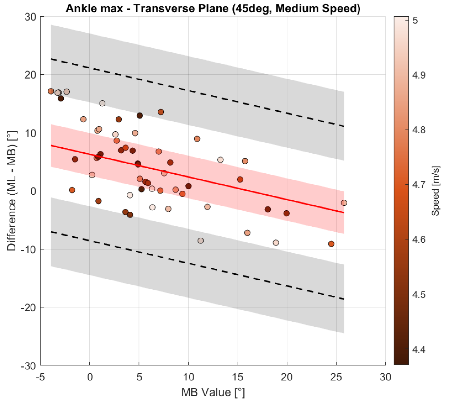

Supplementary Figure 344: Extended BA Plot for maximal ankle angle - Transverse Plane (45deg, Medium)

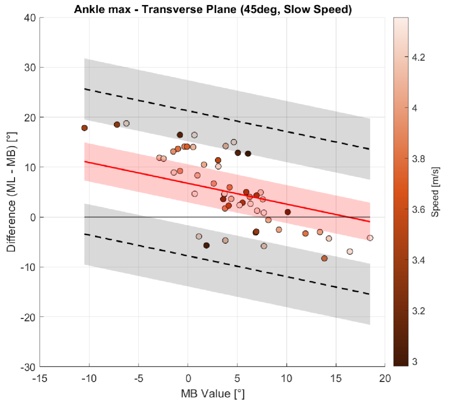

Supplementary Figure 345: Extended BA Plot for maximal ankle angle - Transverse Plane (45deg, Slow)

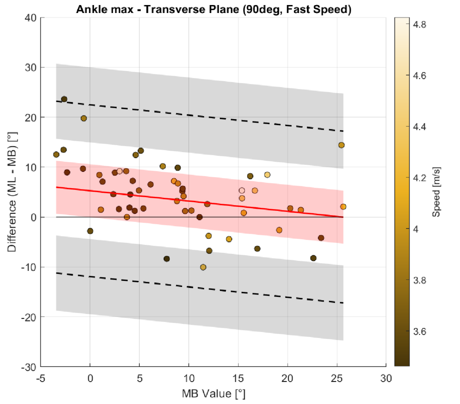

Supplementary Figure 346: Extended BA Plot for maximal ankle angle - Transverse Plane (90deg, Fast)

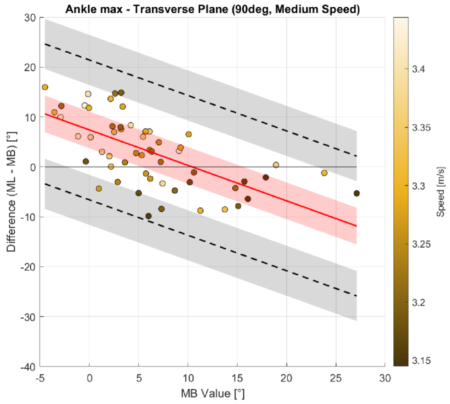

Supplementary Figure 347: Extended BA Plot for maximal ankle angle - Transverse Plane (90deg, Medium)

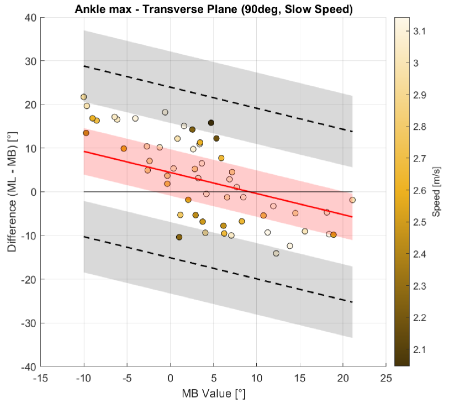

Supplementary Figure 348: Extended BA Plot for maximal ankle angle - Transverse Plane (90deg, Slow)

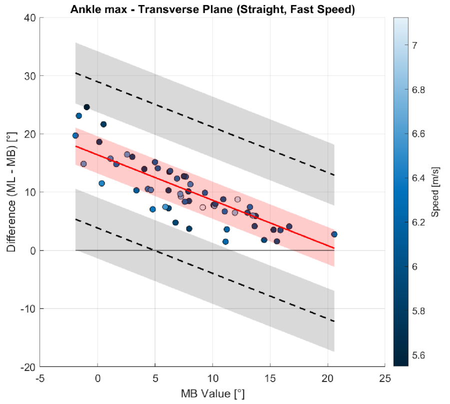

Supplementary Figure 349: Extended BA Plot for maximal ankle angle - Transverse Plane (Straight, Fast)

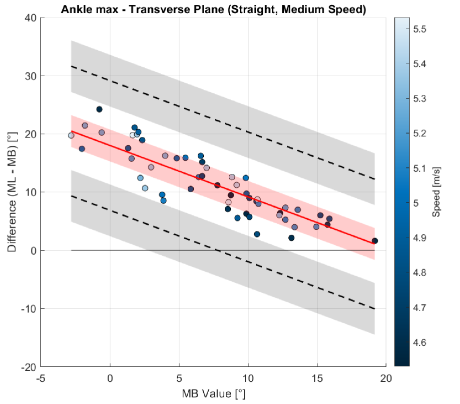

Supplementary Figure 350: Extended BA Plot for maximal ankle angle - Transverse Plane (Straight, Medium)

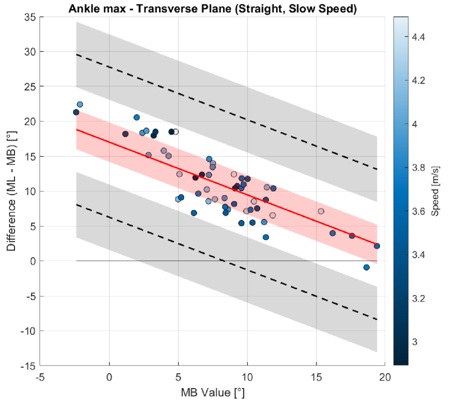

Supplementary Figure 351: Extended BA Plot for maximal ankle angle - Transverse Plane (Straight, Slow)

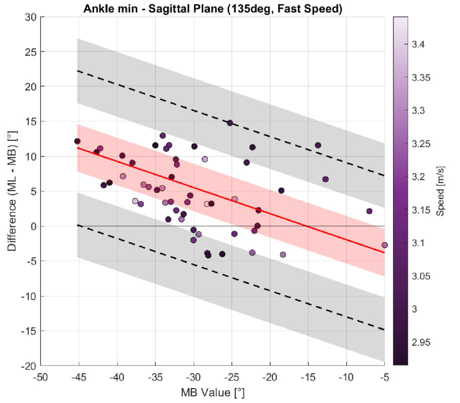

Supplementary Figure 352: Extended BA Plot for minimal ankle angle - Sagittal Plane (135deg, Fast)

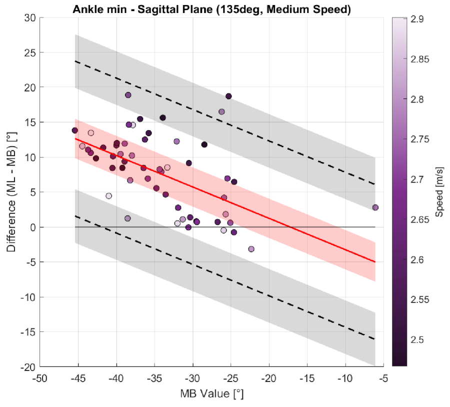

Supplementary Figure 353: Extended BA Plot for minimal ankle angle - Sagittal Plane (135deg, Medium)

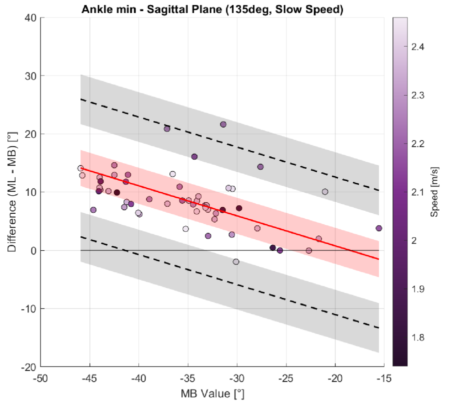

Supplementary Figure 354: Extended BA Plot for minimal ankle angle - Sagittal Plane (135deg, Slow)

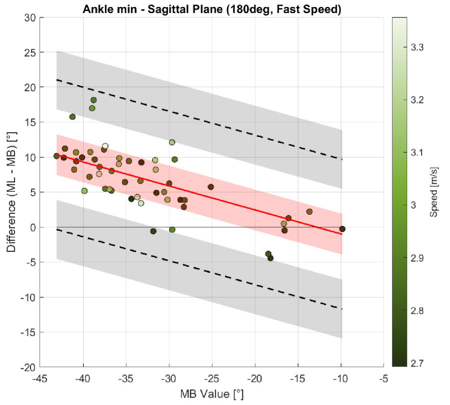

Supplementary Figure 355: Extended BA Plot for minimal ankle angle - Sagittal Plane (180deg, Fast)

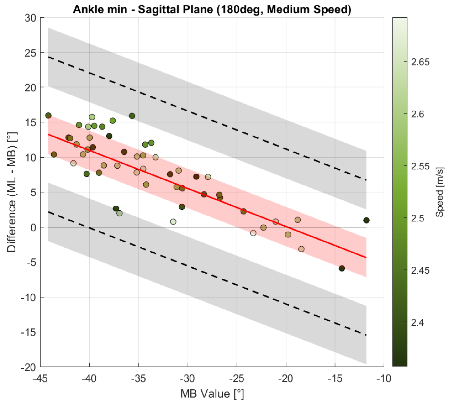

Supplementary Figure 356: Extended BA Plot for minimal ankle angle - Sagittal Plane (180deg, Medium)

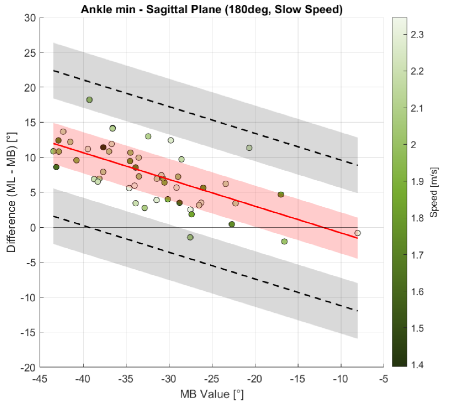

Supplementary Figure 357: Extended BA Plot for minimal ankle angle - Sagittal Plane (180deg, Slow)

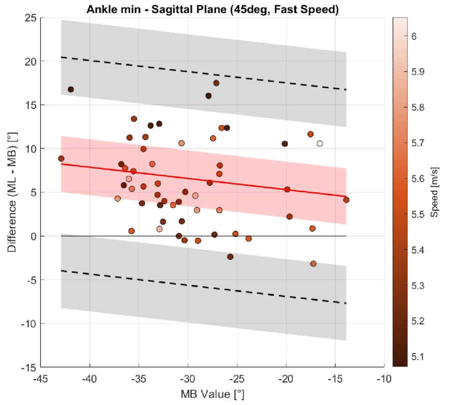

Supplementary Figure 358: Extended BA Plot for minimal ankle angle - Sagittal Plane (45deg, Fast)

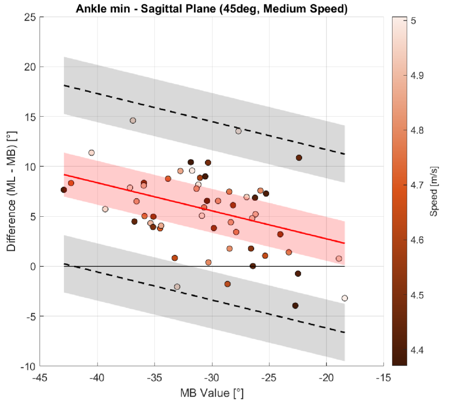

Supplementary Figure 359: Extended BA Plot for minimal ankle angle - Sagittal Plane (45deg, Medium)

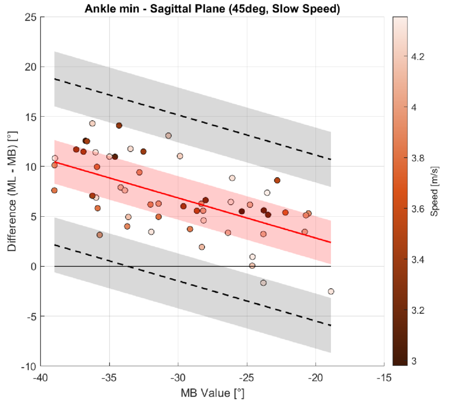

Supplementary Figure 360: Extended BA Plot for minimal ankle angle - Sagittal Plane (45deg, Slow)

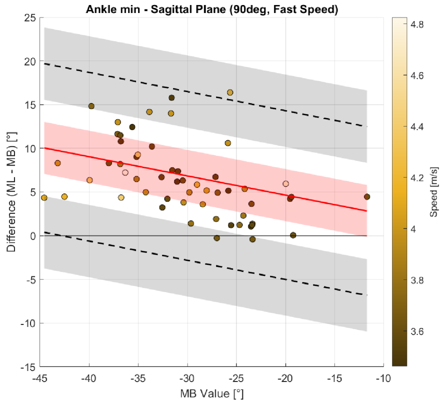

Supplementary Figure 361: Extended BA Plot for minimal ankle angle - Sagittal Plane (90deg, Fast)

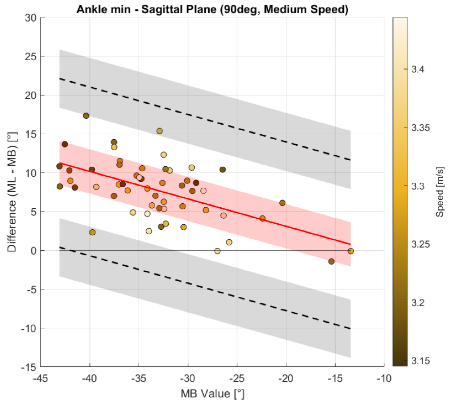

Supplementary Figure 362: Extended BA Plot for minimal ankle angle - Sagittal Plane (90deg, Medium)

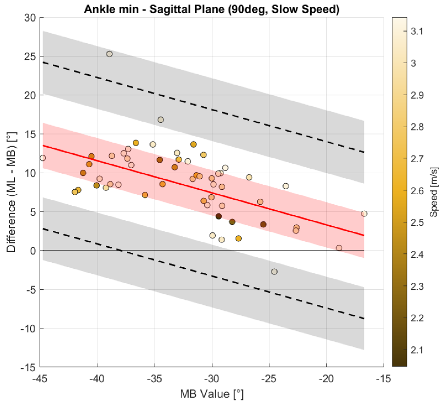

Supplementary Figure 363: Extended BA Plot for minimal ankle angle - Sagittal Plane (90deg, Slow)

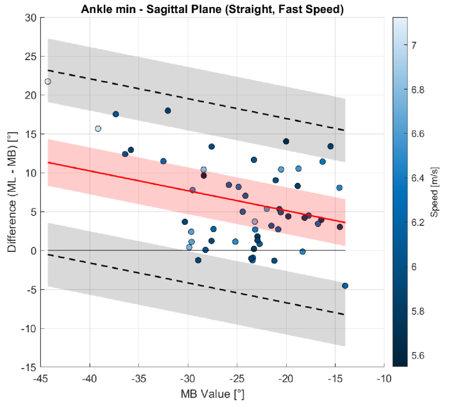

Supplementary Figure 364: Extended BA Plot for minimal ankle angle - Sagittal Plane (Straight, Fast)

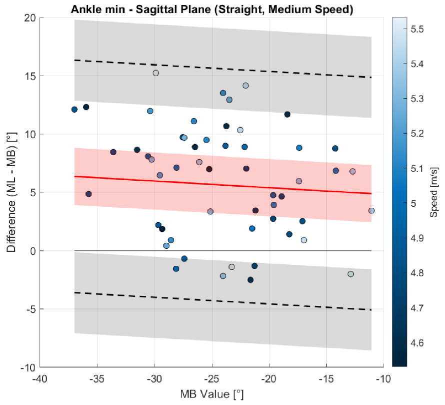

Supplementary Figure 365: Extended BA Plot for minimal ankle angle - Sagittal Plane (Straight, Medium)

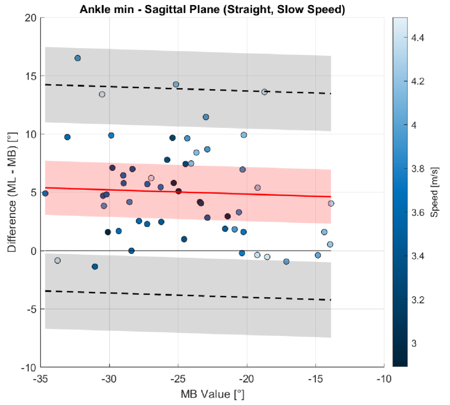

Supplementary Figure 366: Extended BA Plot for minimal ankle angle - Sagittal Plane (Straight, Slow)

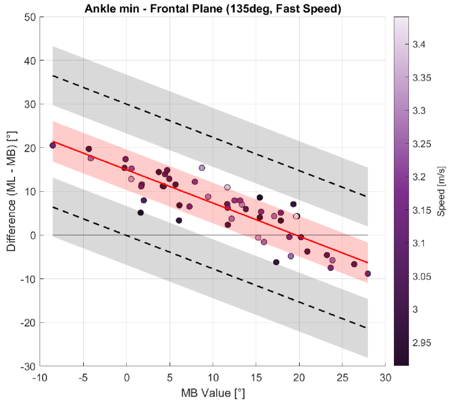

Supplementary Figure 367: Extended BA Plot for minimal ankle angle - Frontal Plane (135deg, Fast)

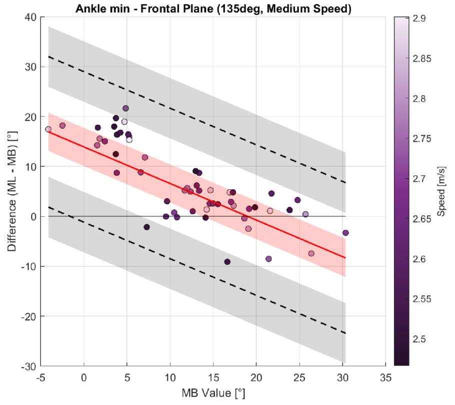

Supplementary Figure 368: Extended BA Plot for minimal ankle angle - Frontal Plane (135deg, Medium)

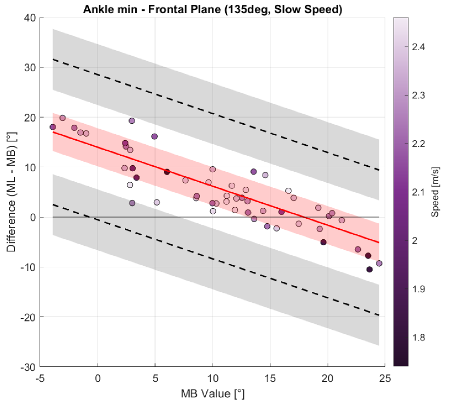

Supplementary Figure 369: Extended BA Plot for minimal ankle angle - Frontal Plane (135deg, Slow)

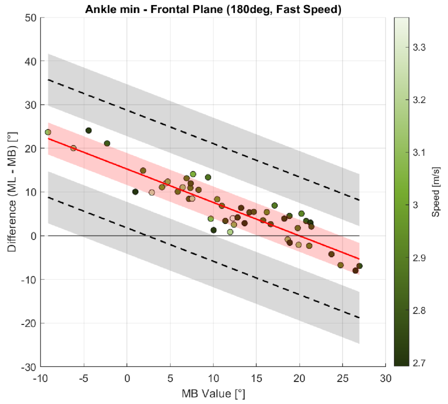

Supplementary Figure 370: Extended BA Plot for minimal ankle angle - Frontal Plane (180deg, Fast)

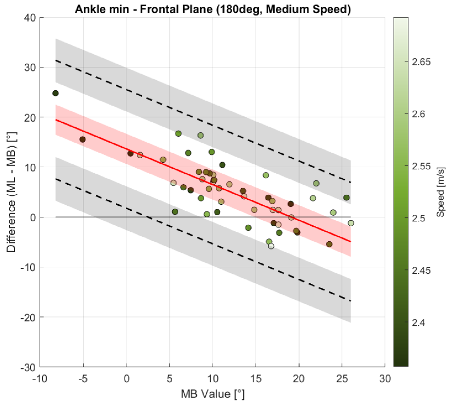

Supplementary Figure 371: Extended BA Plot for minimal ankle angle - Frontal Plane (180deg, Medium)

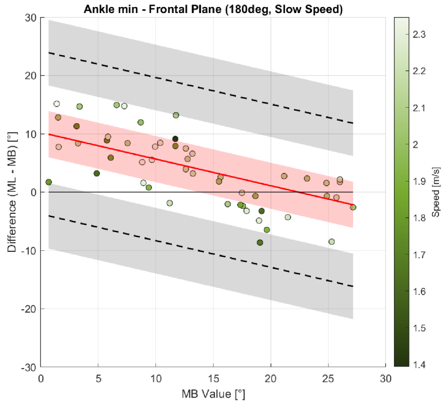

Supplementary Figure 372: Extended BA Plot for minimal ankle angle - Frontal Plane (180deg, Slow)

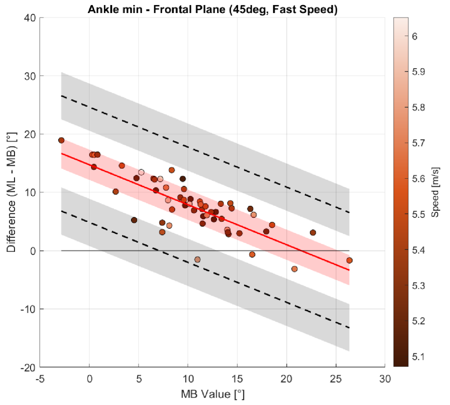

Supplementary Figure 373: Extended BA Plot for minimal ankle angle - Frontal Plane (45deg, Fast)

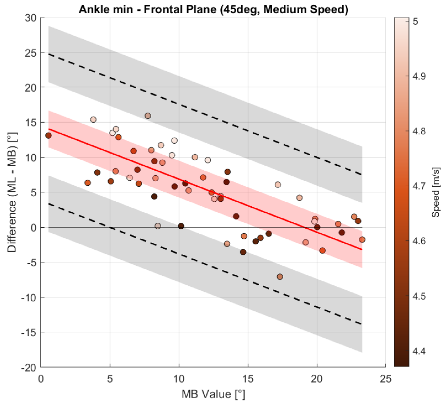

Supplementary Figure 374: Extended BA Plot for minimal ankle angle - Frontal Plane (45deg, Medium)

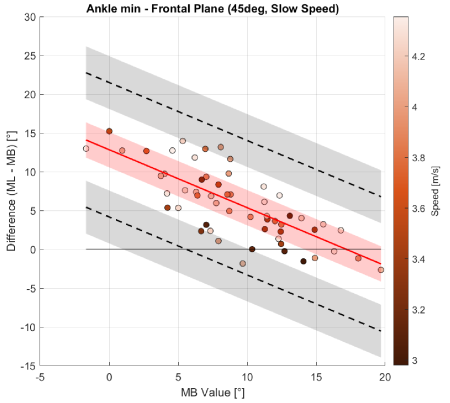

Supplementary Figure 375: Extended BA Plot for minimal ankle angle - Frontal Plane (45deg, Slow)

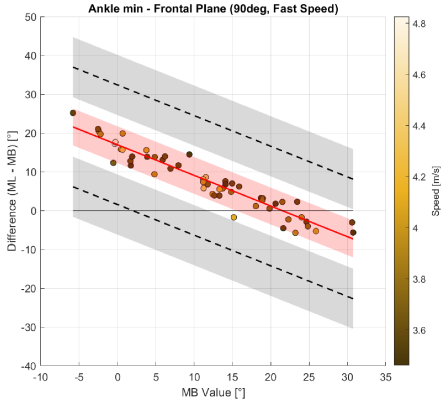

Supplementary Figure 376: Extended BA Plot for minimal ankle angle - Frontal Plane (90deg, Fast)

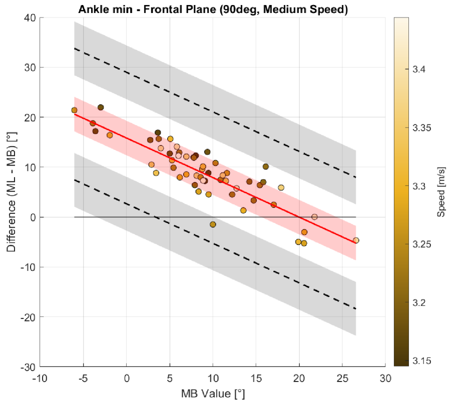

Supplementary Figure 377: Extended BA Plot for minimal ankle angle - Frontal Plane (90deg, Medium)

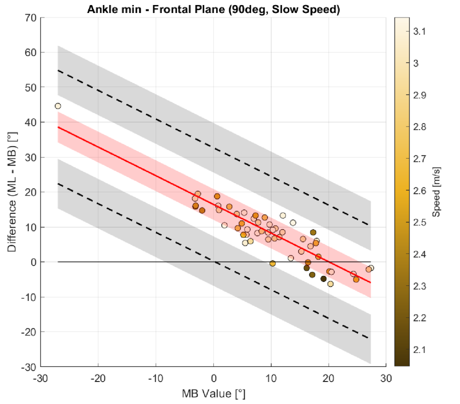

Supplementary Figure 378: Extended BA Plot for minimal ankle angle - Frontal Plane (90deg, Slow)

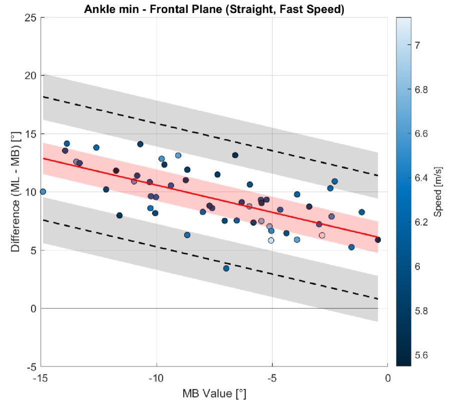

Supplementary Figure 379: Extended BA Plot for minimal ankle angle - Frontal Plane (Straight, Fast)

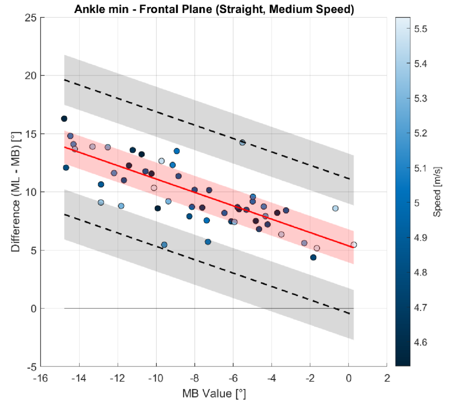

Supplementary Figure 380: Extended BA Plot for minimal ankle angle - Frontal Plane (Straight, Medium)

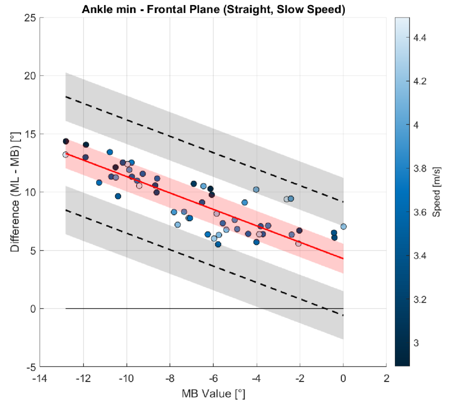

Supplementary Figure 381: Extended BA Plot for minimal ankle angle - Frontal Plane (Straight, Slow)

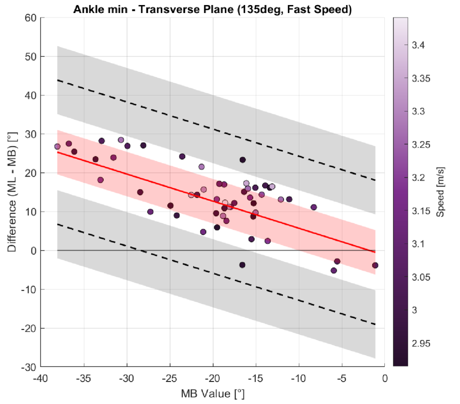

Supplementary Figure 382: Extended BA Plot for minimal ankle angle - Transverse Plane (135deg, Fast)

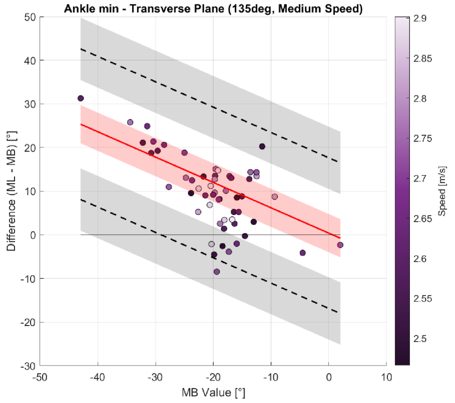

Supplementary Figure 383: Extended BA Plot for minimal ankle angle - Transverse Plane (135deg, Medium)

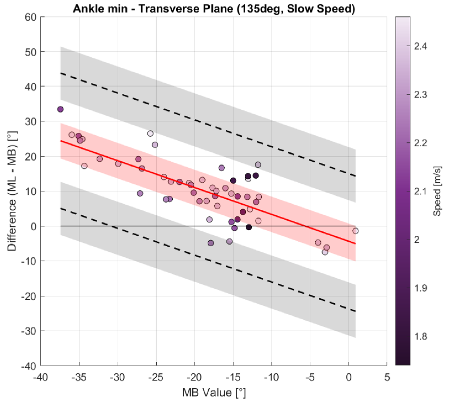

Supplementary Figure 384: Extended BA Plot for minimal ankle angle - Transverse Plane (135deg, Slow)

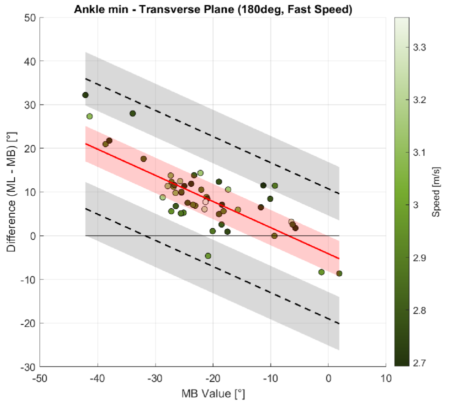

Supplementary Figure 385: Extended BA Plot for minimal ankle angle - Transverse Plane (180deg, Fast)

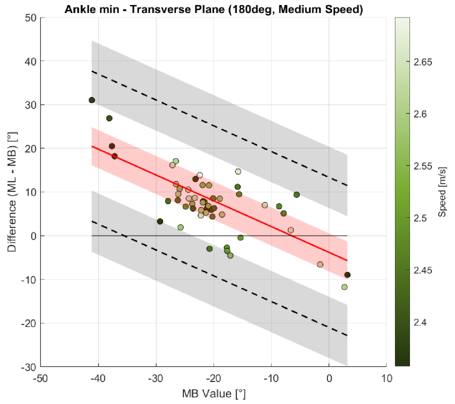

Supplementary Figure 386: Extended BA Plot for minimal ankle angle - Transverse Plane (180deg, Medium)

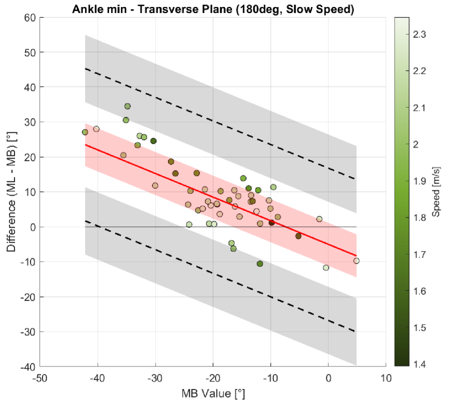

Supplementary Figure 387: Extended BA Plot for minimal ankle angle - Transverse Plane (180deg, Slow)

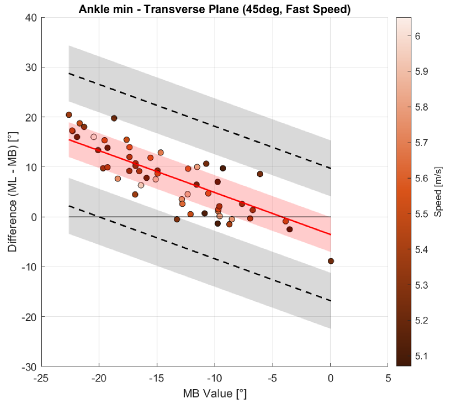

Supplementary Figure 388: Extended BA Plot for minimal ankle angle - Transverse Plane (45deg, Fast)

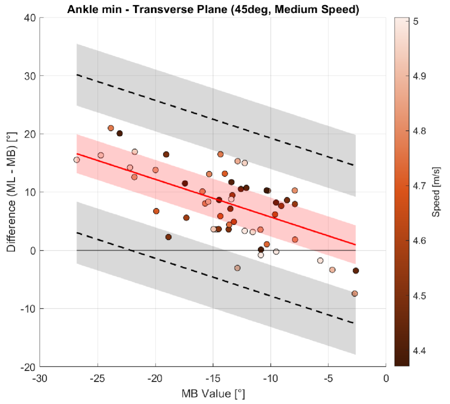

Supplementary Figure 389: Extended BA Plot for minimal ankle angle - Transverse Plane (45deg, Medium)

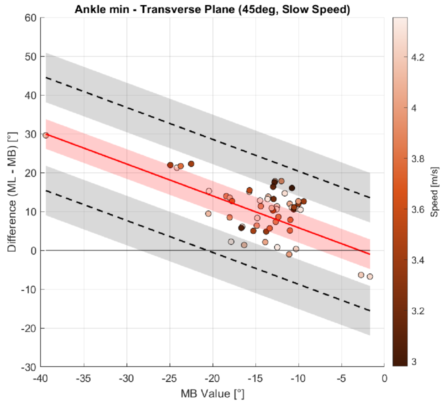

Supplementary Figure 390: Extended BA Plot for minimal ankle angle - Transverse Plane (45deg, Slow)

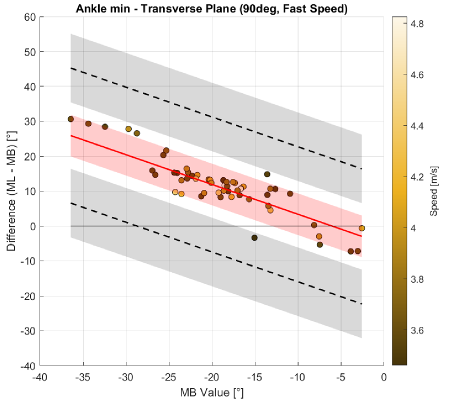

Supplementary Figure 391: Extended BA Plot for minimal ankle angle - Transverse Plane (90deg, Fast)

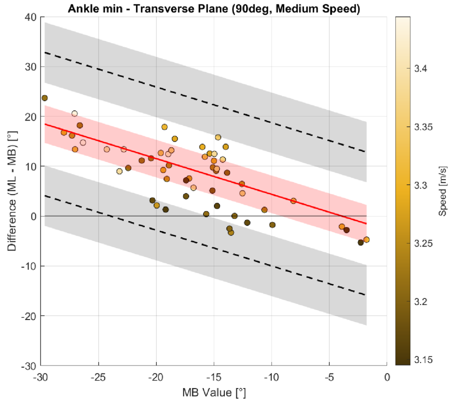

Supplementary Figure 392: Extended BA Plot for minimal ankle angle - Transverse Plane (90deg, Medium)

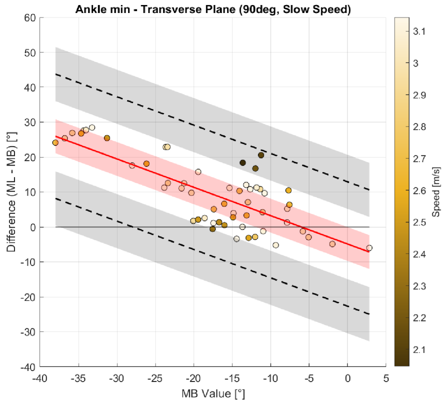

Supplementary Figure 393: Extended BA Plot for minimal ankle angle - Transverse Plane (90deg, Slow)

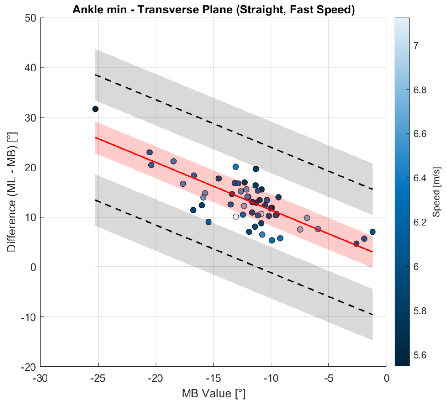

Supplementary Figure 394: Extended BA Plot for minimal ankle angle - Transverse Plane (Straight, Fast)

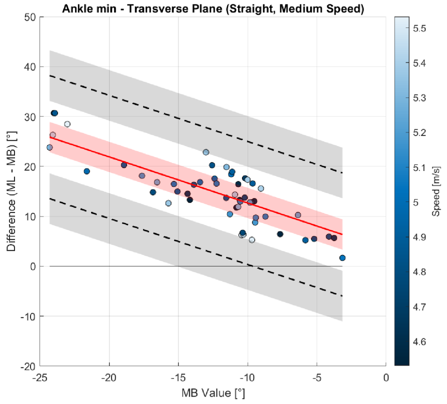

Supplementary Figure 395: Extended BA Plot for minimal ankle angle - Transverse Plane (Straight, Medium)

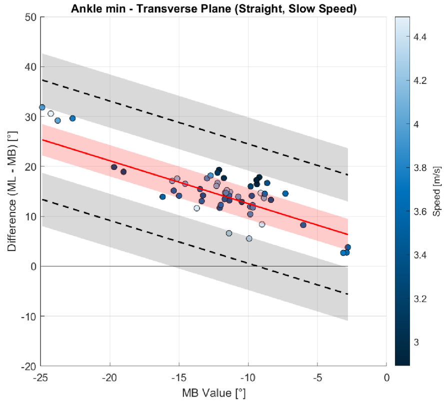

Supplementary Figure 396: Extended BA Plot for minimal ankle angle - Transverse Plane (Straight, Slow)

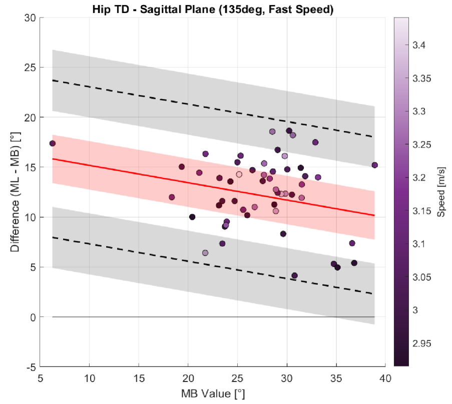

Supplementary Figure 397: Extended BA Plot for TD hip angle - Sagittal Plane (135deg, Fast)

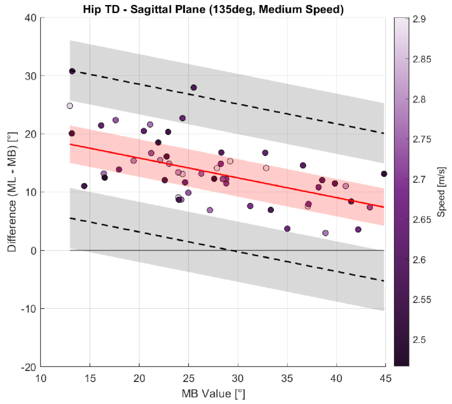

Supplementary Figure 398: Extended BA Plot for TD hip angle - Sagittal Plane (135deg, Medium)

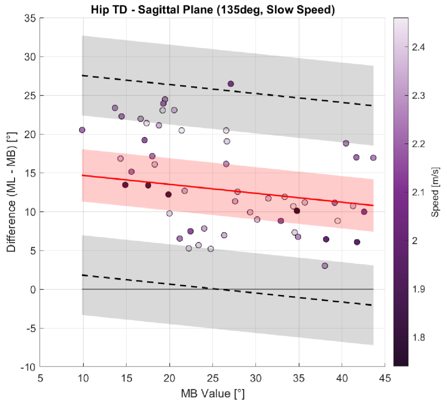

Supplementary Figure 399: Extended BA Plot for TD hip angle - Sagittal Plane (135deg, Slow)

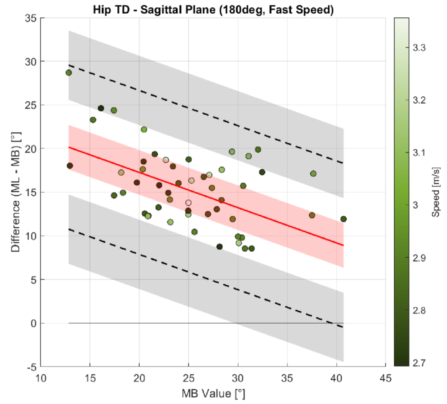

Supplementary Figure 400: Extended BA Plot for TD hip angle - Sagittal Plane (180deg, Fast)

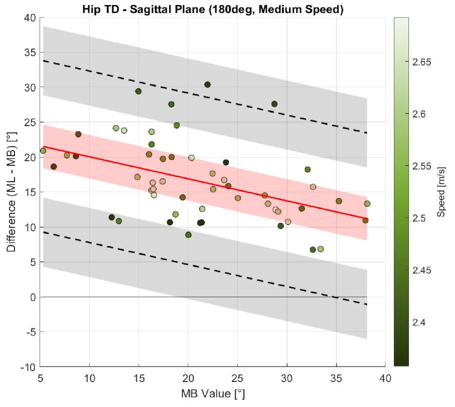

Supplementary Figure 401: Extended BA Plot for TD hip angle - Sagittal Plane (180deg, Medium)

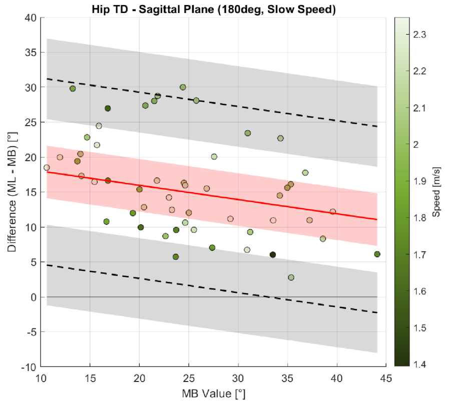

Supplementary Figure 402: Extended BA Plot for TD hip angle - Sagittal Plane (180deg, Slow)

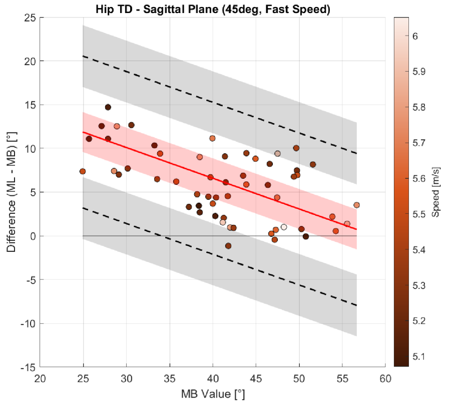

Supplementary Figure 403: Extended BA Plot for TD hip angle - Sagittal Plane (45deg, Fast)

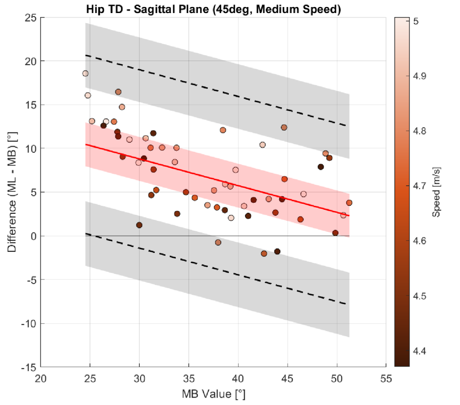

Supplementary Figure 404: Extended BA Plot for TD hip angle - Sagittal Plane (45deg, Medium)

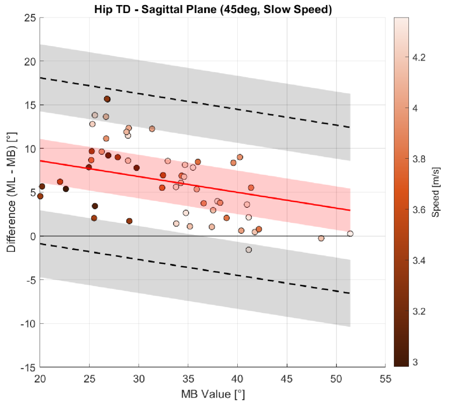

Supplementary Figure 405: Extended BA Plot for TD hip angle - Sagittal Plane (45deg, Slow)

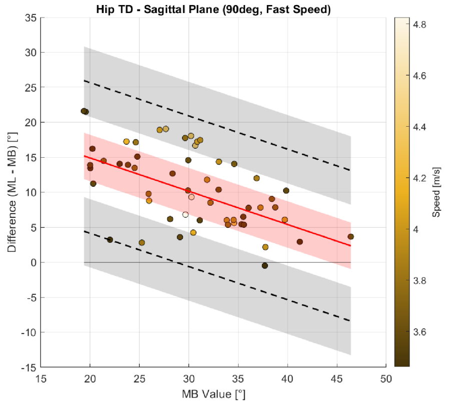

Supplementary Figure 406: Extended BA Plot for TD hip angle - Sagittal Plane (90deg, Fast)

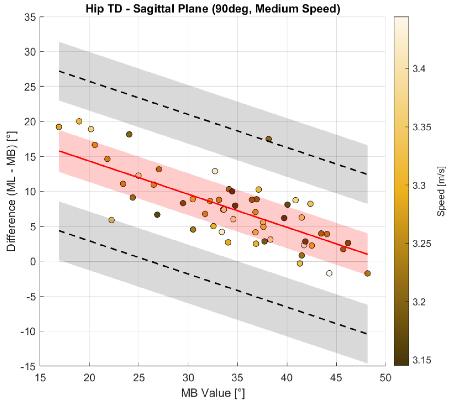

Supplementary Figure 407: Extended BA Plot for TD hip angle - Sagittal Plane (90deg, Medium)

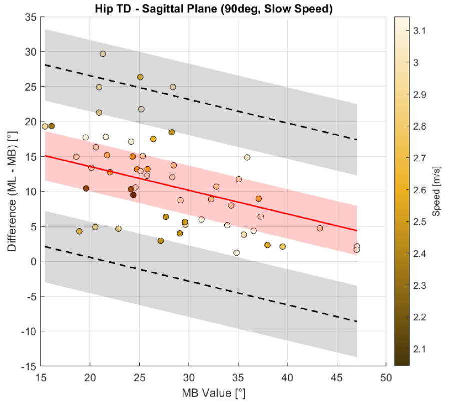

Supplementary Figure 408: Extended BA Plot for TD hip angle - Sagittal Plane (90deg, Slow)

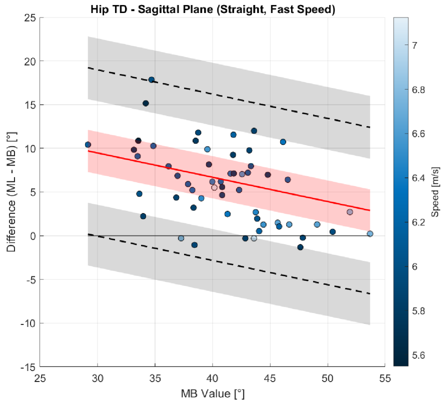

Supplementary Figure 409: Extended BA Plot for TD hip angle - Sagittal Plane (Straight, Fast)

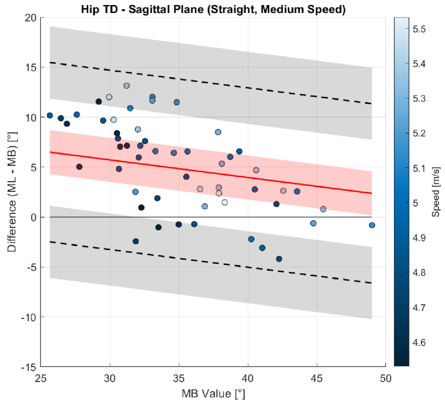

Supplementary Figure 410: Extended BA Plot for TD hip angle - Sagittal Plane (Straight, Medium)

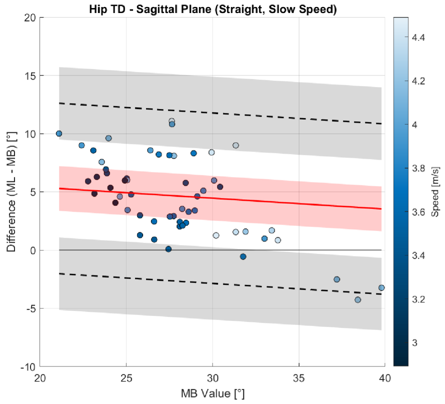

Supplementary Figure 411: Extended BA Plot for TD hip angle - Sagittal Plane (Straight, Slow)

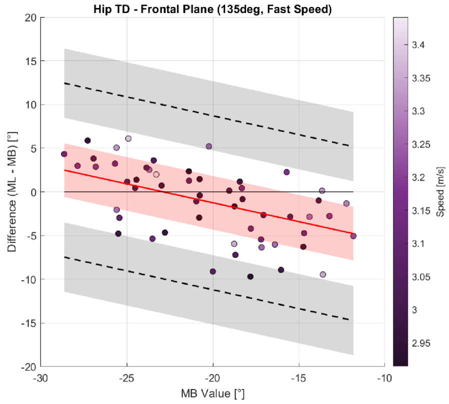

Supplementary Figure 412: Extended BA Plot for TD hip angle - Frontal Plane (135deg, Fast)

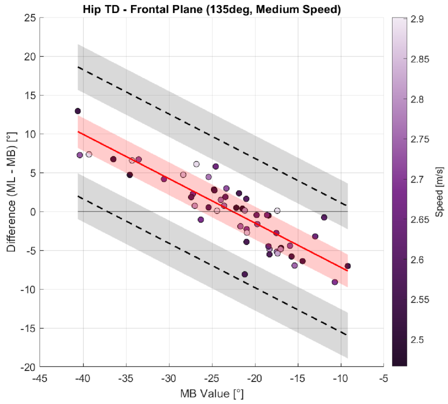

Supplementary Figure 413: Extended BA Plot for TD hip angle - Frontal Plane (135deg, Medium)

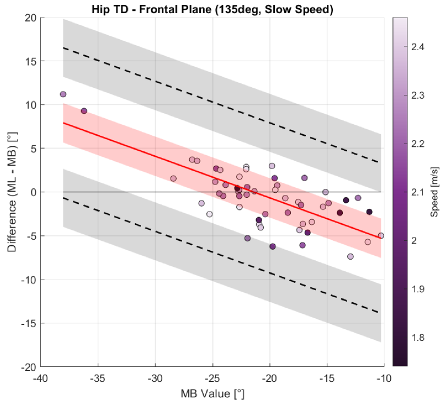

Supplementary Figure 414: Extended BA Plot for TD hip angle - Frontal Plane (135deg, Slow)

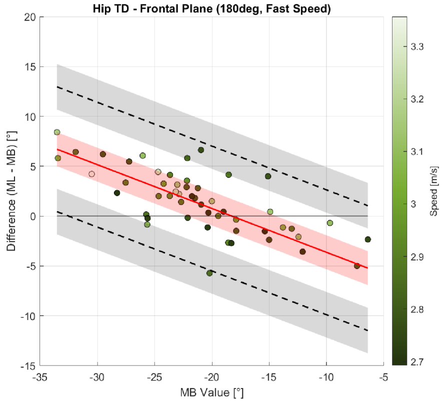

Supplementary Figure 415: Extended BA Plot for TD hip angle - Frontal Plane (180deg, Fast)

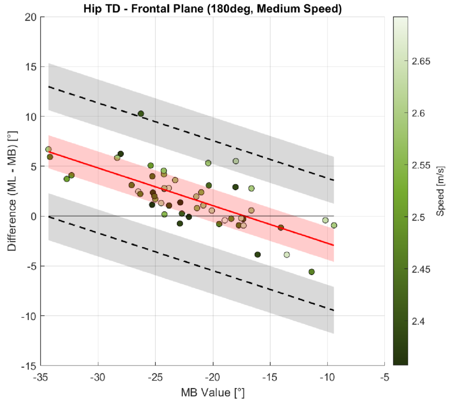

Supplementary Figure 416: Extended BA Plot for TD hip angle - Frontal Plane (180deg, Medium)

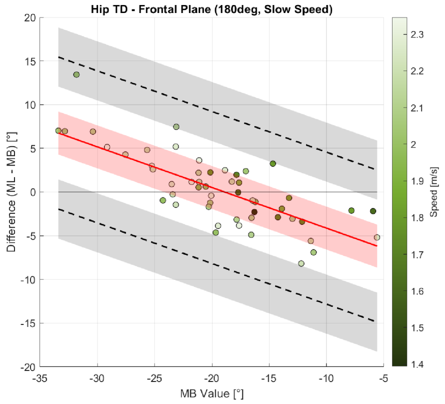

Supplementary Figure 417: Extended BA Plot for TD hip angle - Frontal Plane (180deg, Slow)

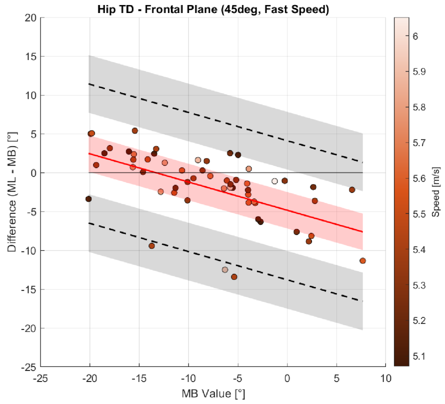

Supplementary Figure 418: Extended BA Plot for TD hip angle - Frontal Plane (45deg, Fast)

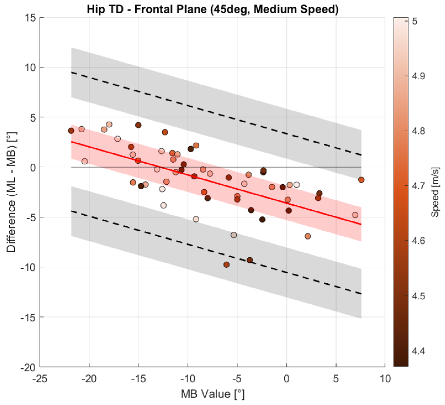

Supplementary Figure 419: Extended BA Plot for TD hip angle - Frontal Plane (45deg, Medium)

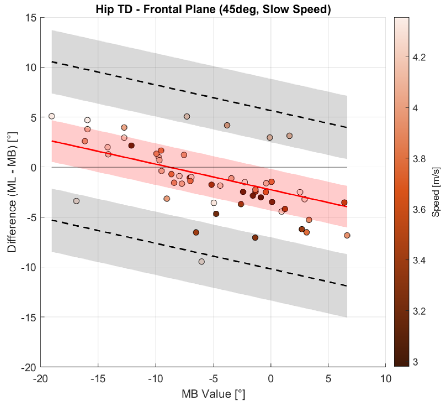

Supplementary Figure 420: Extended BA Plot for TD hip angle - Frontal Plane (45deg, Slow)

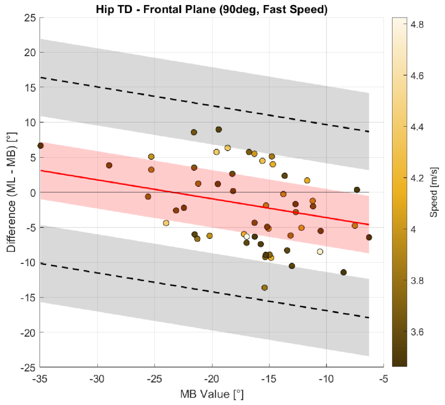

Supplementary Figure 421: Extended BA Plot for TD hip angle - Frontal Plane (90deg, Fast)

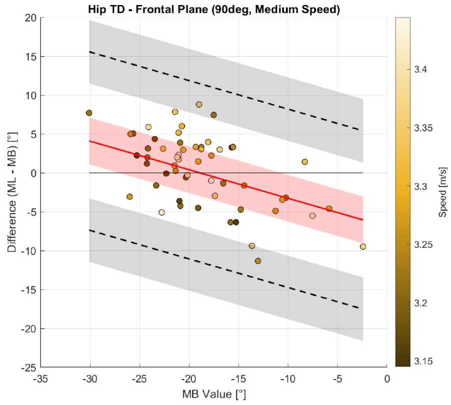

Supplementary Figure 422: Extended BA Plot for TD hip angle - Frontal Plane (90deg, Medium)

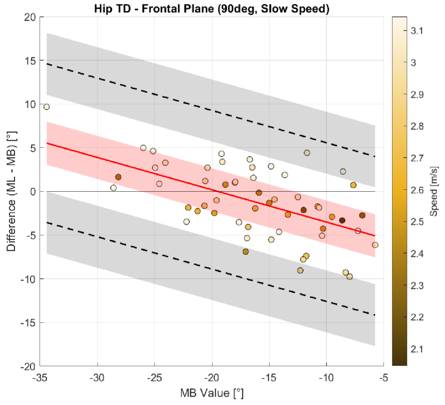

Supplementary Figure 423: Extended BA Plot for TD hip angle - Frontal Plane (90deg, Slow)

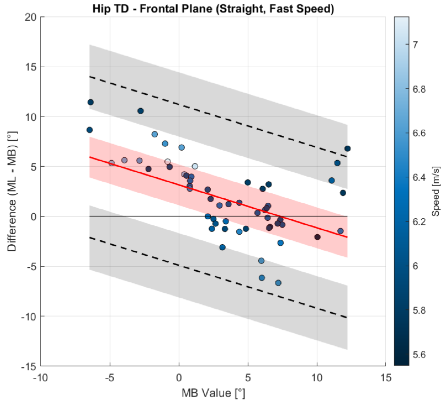

Supplementary Figure 424: Extended BA Plot for TD hip angle - Frontal Plane (Straight, Fast)

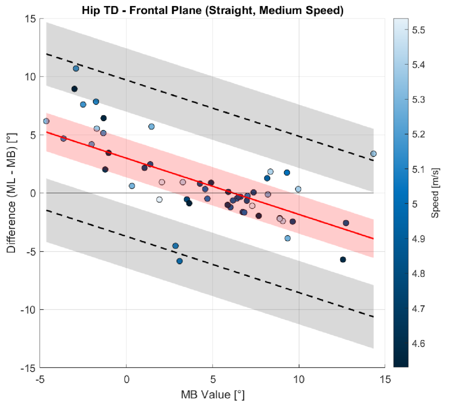

Supplementary Figure 425: Extended BA Plot for TD hip angle - Frontal Plane (Straight, Medium)

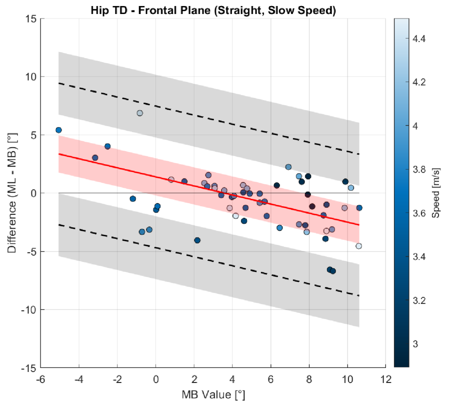

Supplementary Figure 426: Extended BA Plot for TD hip angle - Frontal Plane (Straight, Slow)

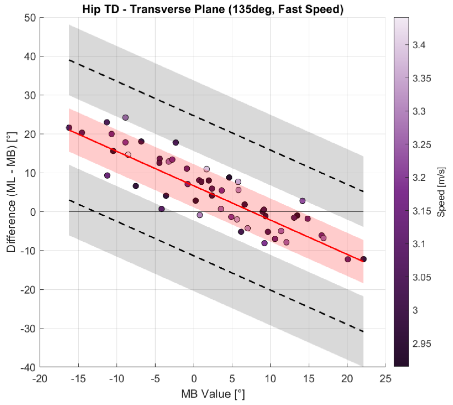

Supplementary Figure 427: Extended BA Plot for TD hip angle - Transverse Plane (135deg, Fast)

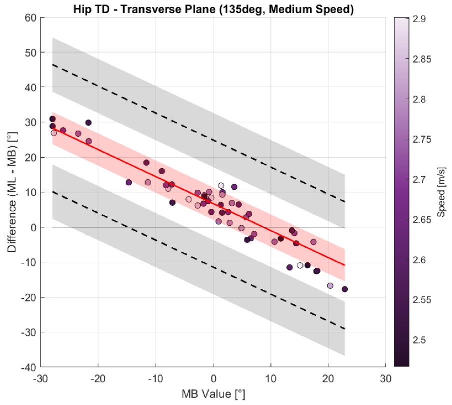

Supplementary Figure 428: Extended BA Plot for TD hip angle - Transverse Plane (135deg, Medium)

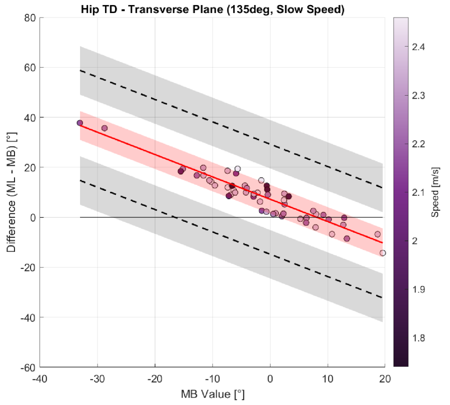

Supplementary Figure 429: Extended BA Plot for TD hip angle - Transverse Plane (135deg, Slow)

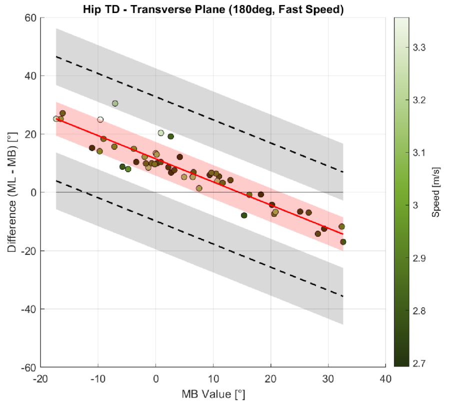

Supplementary Figure 430: Extended BA Plot for TD hip angle - Transverse Plane (180deg, Fast)

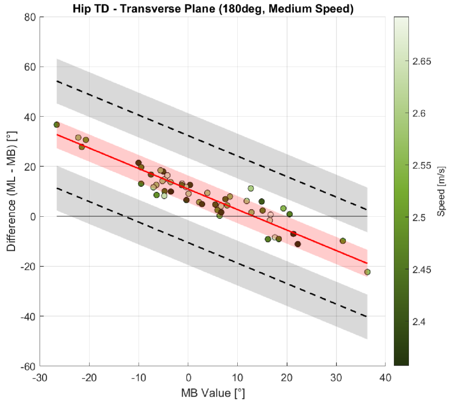

Supplementary Figure 431: Extended BA Plot for TD hip angle - Transverse Plane (180deg, Medium)

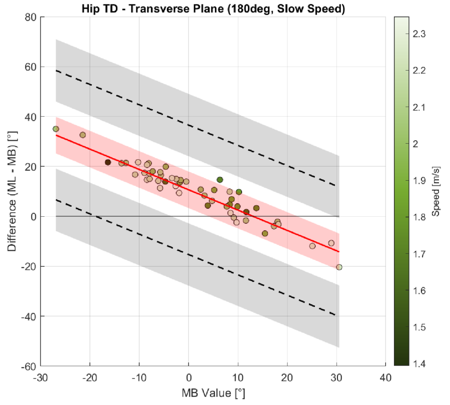

Supplementary Figure 432: Extended BA Plot for TD hip angle - Transverse Plane (180deg, Slow)

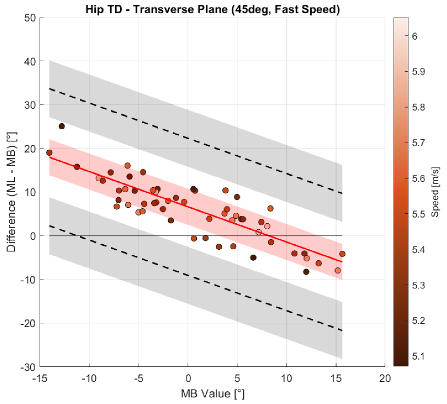

Supplementary Figure 433: Extended BA Plot for TD hip angle - Transverse Plane (45deg, Fast)

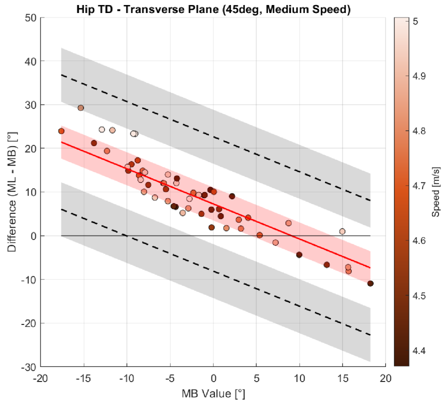

Supplementary Figure 434: Extended BA Plot for TD hip angle - Transverse Plane (45deg, Medium)

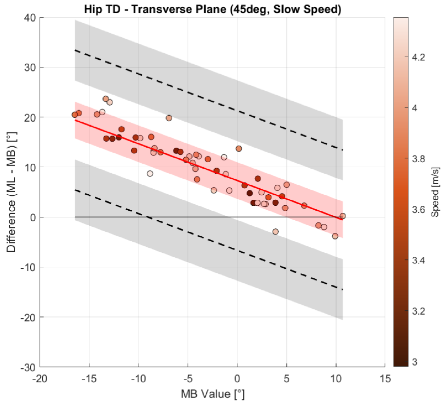

Supplementary Figure 435: Extended BA Plot for TD hip angle - Transverse Plane (45deg, Slow)

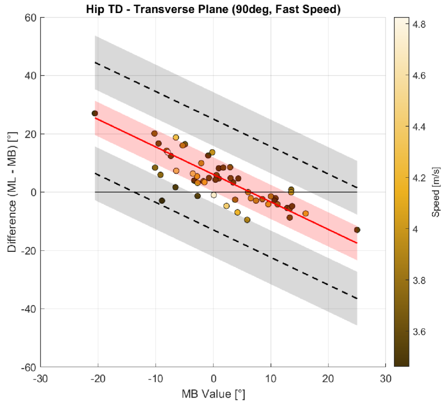

Supplementary Figure 436: Extended BA Plot for TD hip angle - Transverse Plane (90deg, Fast)

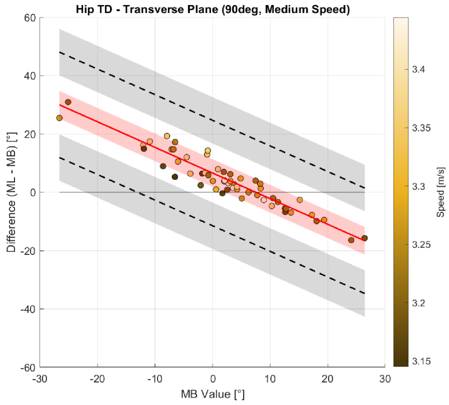

Supplementary Figure 437: Extended BA Plot for TD hip angle - Transverse Plane (90deg, Medium)

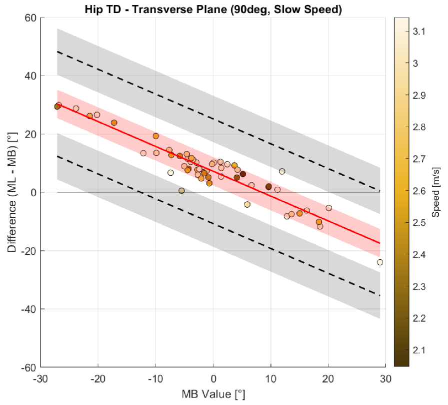

Supplementary Figure 438: Extended BA Plot for TD hip angle - Transverse Plane (90deg, Slow)

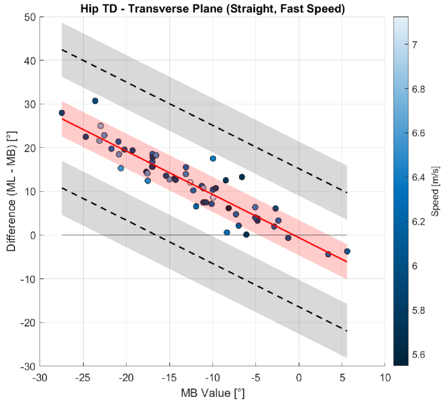

Supplementary Figure 439: Extended BA Plot for TD hip angle - Transverse Plane (Straight, Fast)

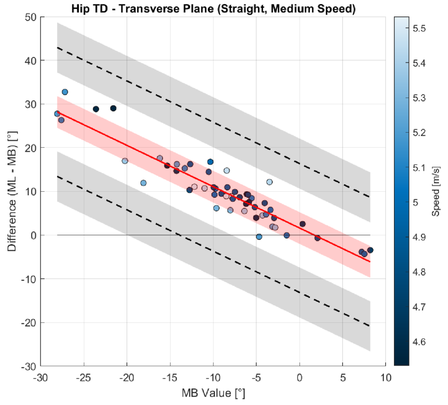

Supplementary Figure 440: Extended BA Plot for TD hip angle - Transverse Plane (Straight, Medium)

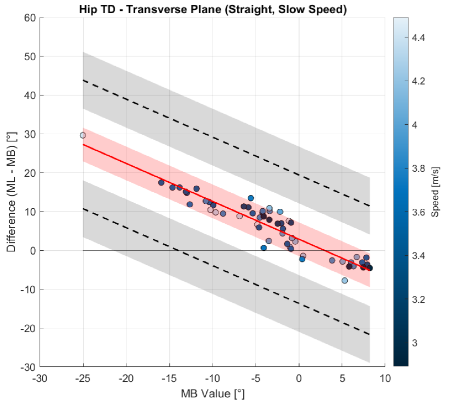

Supplementary Figure 441: Extended BA Plot for TD hip angle - Transverse Plane (Straight, Slow)

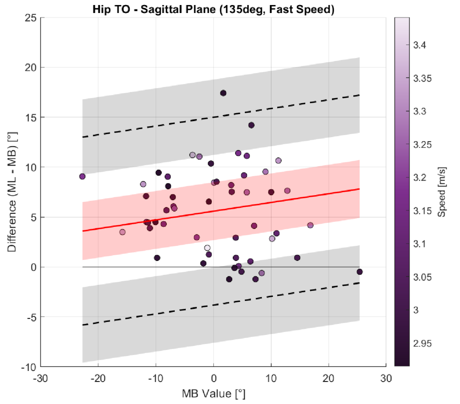

Supplementary Figure 442: Extended BA Plot for TO hip angle - Sagittal Plane (135deg, Fast)

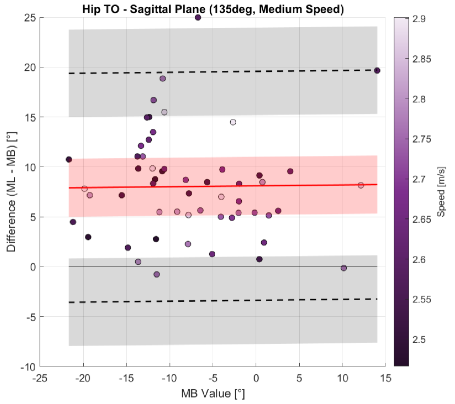

Supplementary Figure 443: Extended BA Plot for TO hip angle - Sagittal Plane (135deg, Medium)

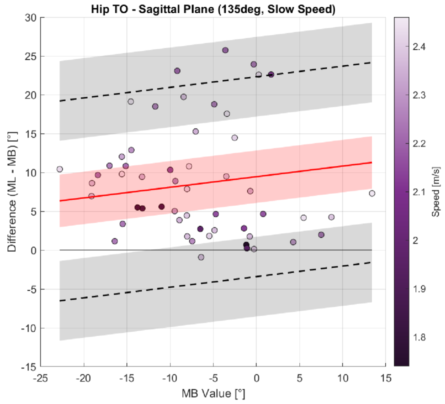

Supplementary Figure 444: Extended BA Plot for TO hip angle - Sagittal Plane (135deg, Slow)

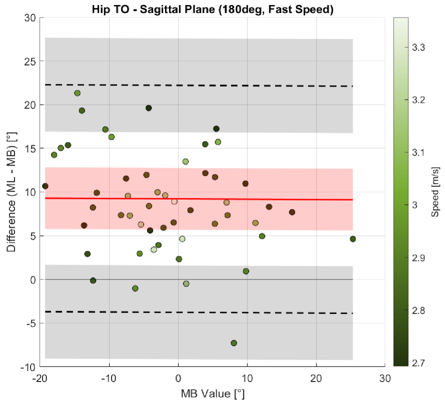

Supplementary Figure 445: Extended BA Plot for TO hip angle - Sagittal Plane (180deg, Fast)

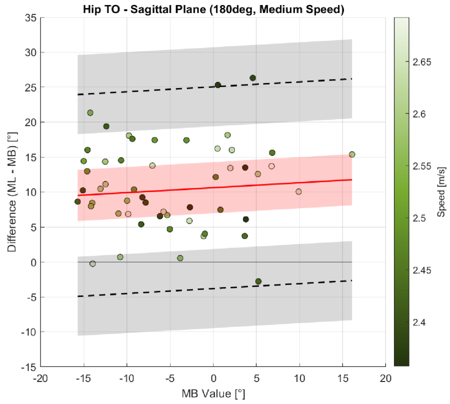

Supplementary Figure 446: Extended BA Plot for TO hip angle - Sagittal Plane (180deg, Medium)

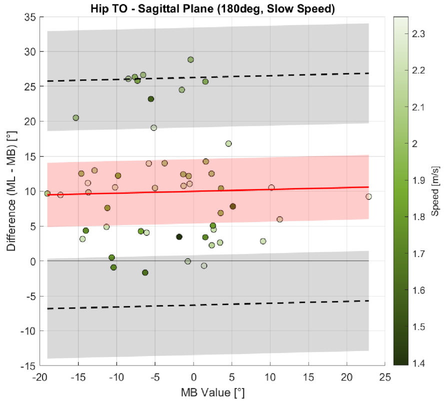

Supplementary Figure 447: Extended BA Plot for TO hip angle - Sagittal Plane (180deg, Slow)

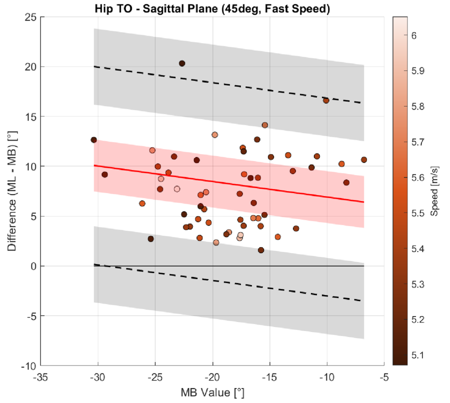

Supplementary Figure 448: Extended BA Plot for TO hip angle - Sagittal Plane (45deg, Fast)

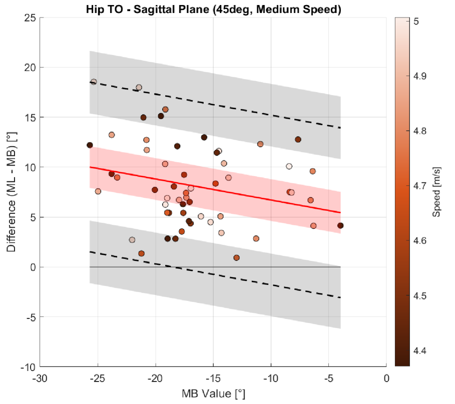

Supplementary Figure 449: Extended BA Plot for TO hip angle - Sagittal Plane (45deg, Medium)

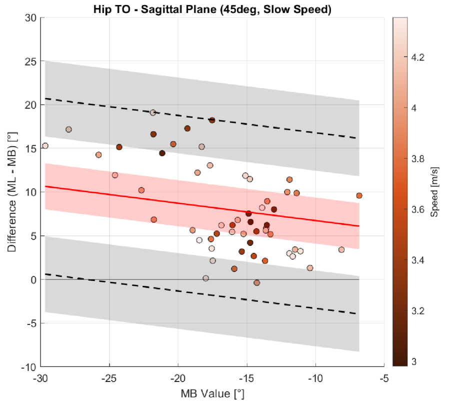

Supplementary Figure 450: Extended BA Plot for TO hip angle - Sagittal Plane (45deg, Slow)

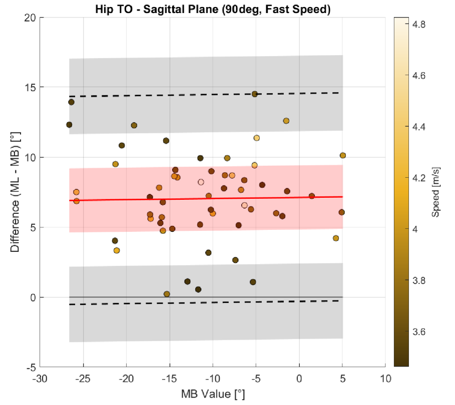

Supplementary Figure 451: Extended BA Plot for TO hip angle - Sagittal Plane (90deg, Fast)

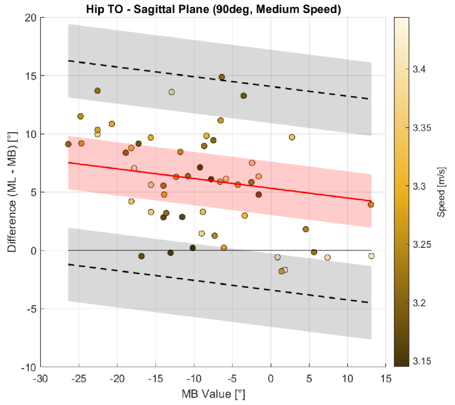

Supplementary Figure 452: Extended BA Plot for TO hip angle - Sagittal Plane (90deg, Medium)

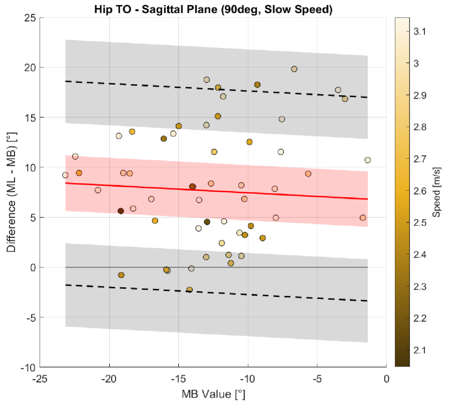

Supplementary Figure 453: Extended BA Plot for TO hip angle - Sagittal Plane (90deg, Slow)

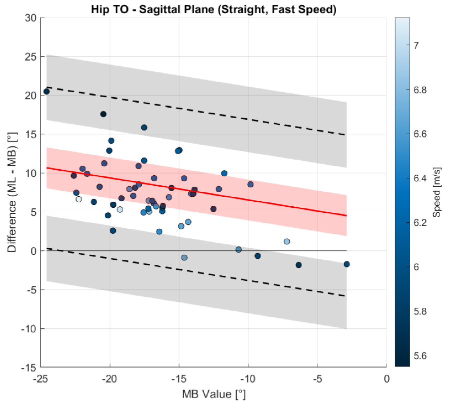

Supplementary Figure 454: Extended BA Plot for TO hip angle - Sagittal Plane (Straight, Fast)

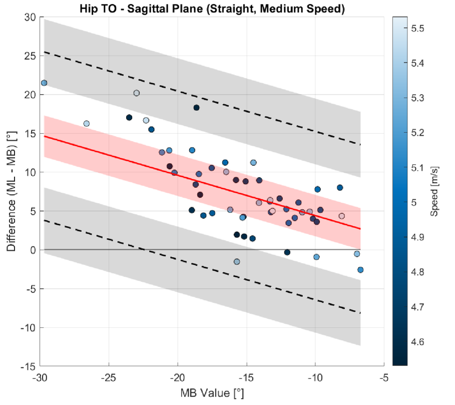

Supplementary Figure 455: Extended BA Plot for TO hip angle - Sagittal Plane (Straight, Medium)

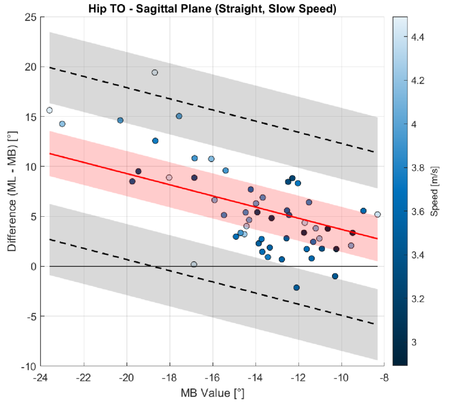

Supplementary Figure 456: Extended BA Plot for TO hip angle - Sagittal Plane (Straight, Slow)

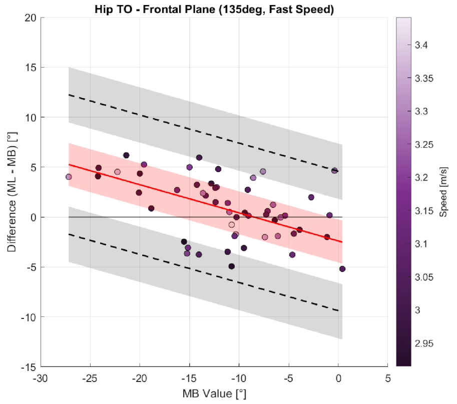

Supplementary Figure 457: Extended BA Plot for TO hip angle - Frontal Plane (135deg, Fast)

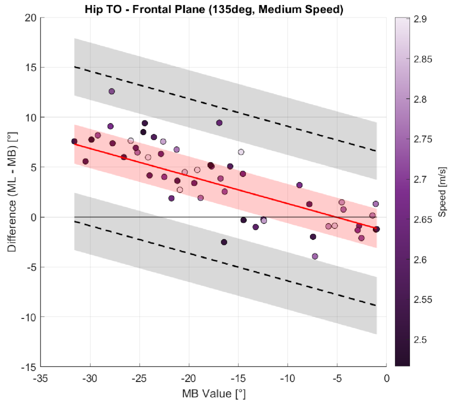

Supplementary Figure 458: Extended BA Plot for TO hip angle - Frontal Plane (135deg, Medium)

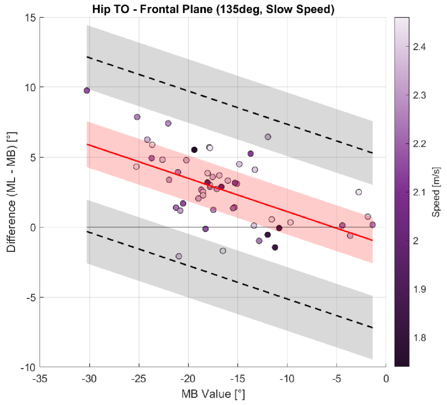

Supplementary Figure 459: Extended BA Plot for TO hip angle - Frontal Plane (135deg, Slow)

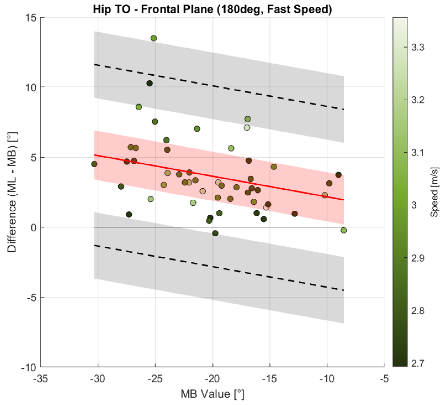

Supplementary Figure 460: Extended BA Plot for TO hip angle - Frontal Plane (180deg, Fast)

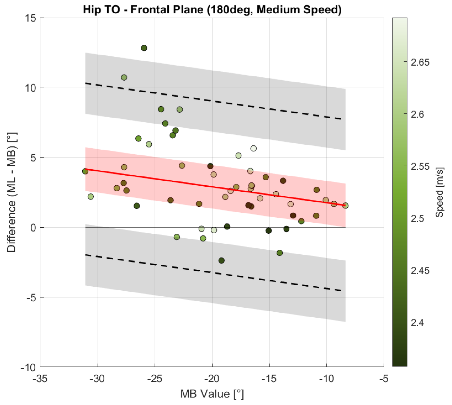

Supplementary Figure 461: Extended BA Plot for TO hip angle - Frontal Plane (180deg, Medium)

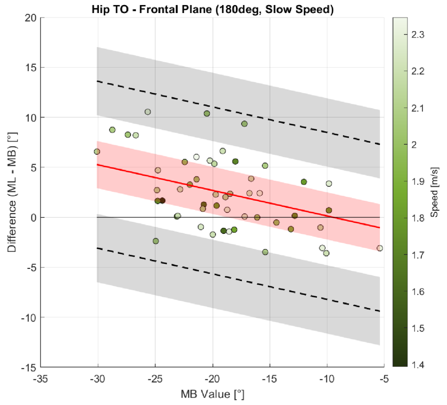

Supplementary Figure 462: Extended BA Plot for TO hip angle - Frontal Plane (180deg, Slow)

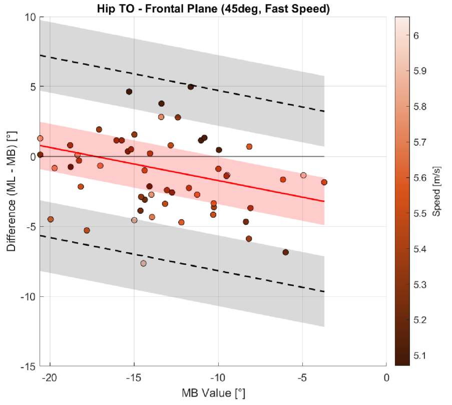

Supplementary Figure 463: Extended BA Plot for TO hip angle - Frontal Plane (45deg, Fast)

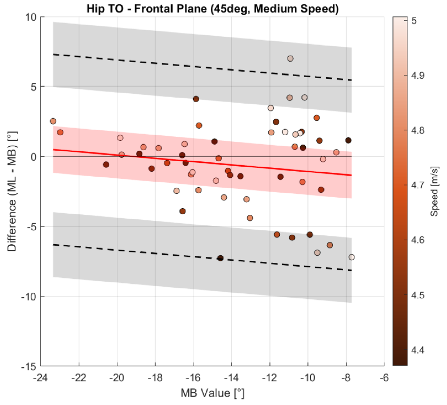

Supplementary Figure 464: Extended BA Plot for TO hip angle - Frontal Plane (45deg, Medium)

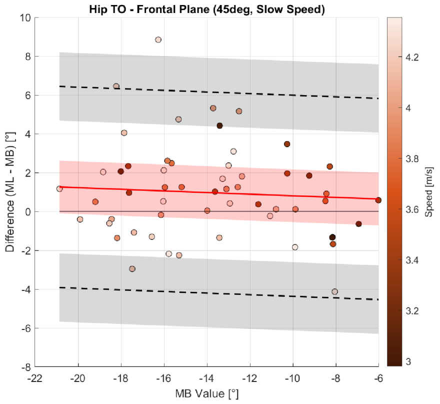

Supplementary Figure 465: Extended BA Plot for TO hip angle - Frontal Plane (45deg, Slow)

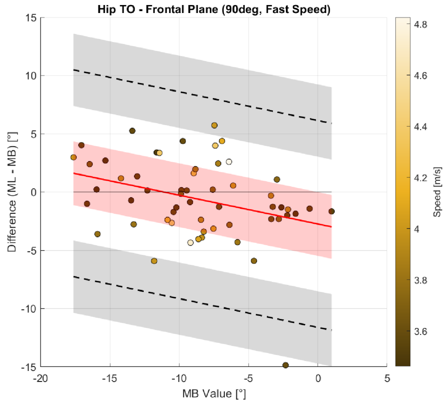

Supplementary Figure 466: Extended BA Plot for TO hip angle - Frontal Plane (90deg, Fast)

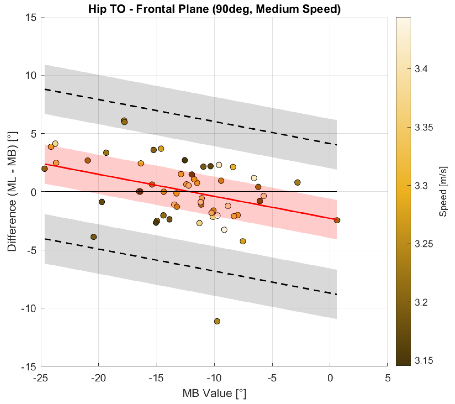

Supplementary Figure 467: Extended BA Plot for TO hip angle - Frontal Plane (90deg, Medium)

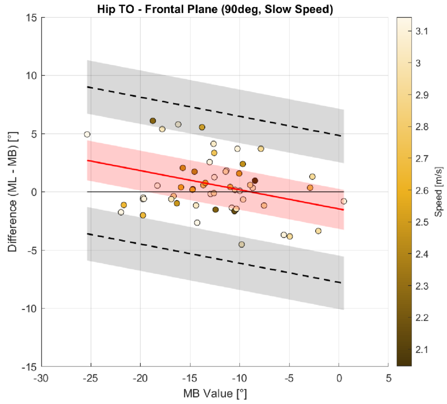

Supplementary Figure 468: Extended BA Plot for TO hip angle - Frontal Plane (90deg, Slow)

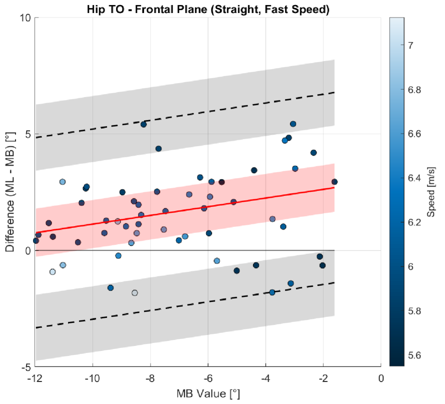

Supplementary Figure 469: Extended BA Plot for TO hip angle - Frontal Plane (Straight, Fast)

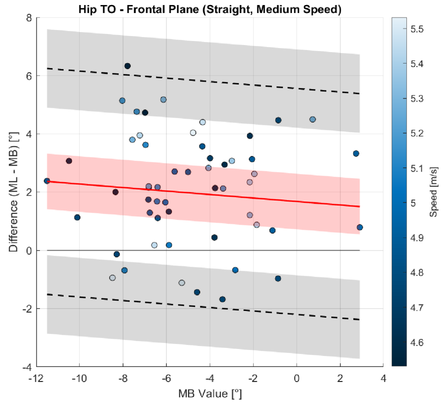

Supplementary Figure 470: Extended BA Plot for TO hip angle - Frontal Plane (Straight, Medium)

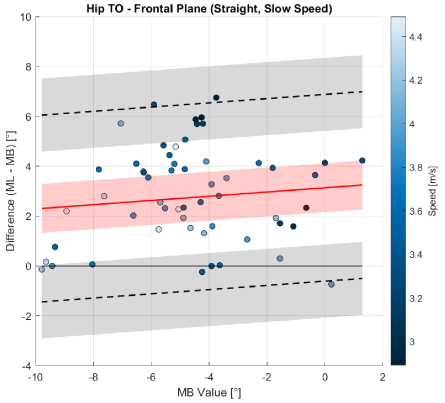

Supplementary Figure 471: Extended BA Plot for TO hip angle - Frontal Plane (Straight, Slow)

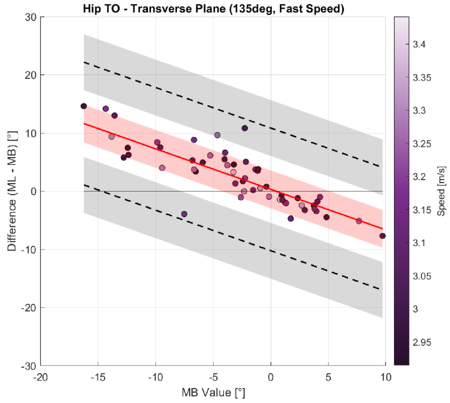

Supplementary Figure 472: Extended BA Plot for TO hip angle - Transverse Plane (135deg, Fast)

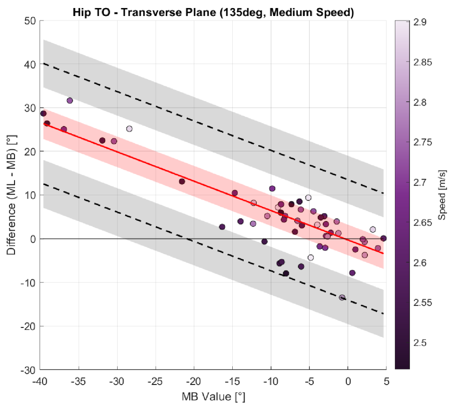

Supplementary Figure 473: Extended BA Plot for TO hip angle - Transverse Plane (135deg, Medium)

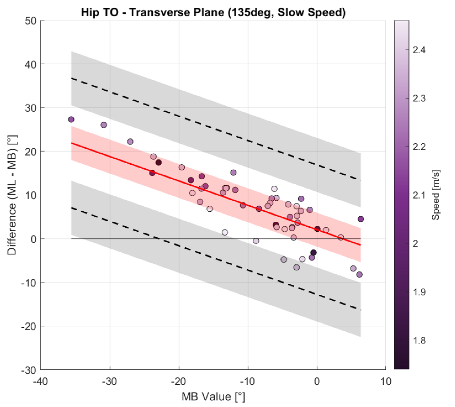

Supplementary Figure 474: Extended BA Plot for TO hip angle - Transverse Plane (135deg, Slow)

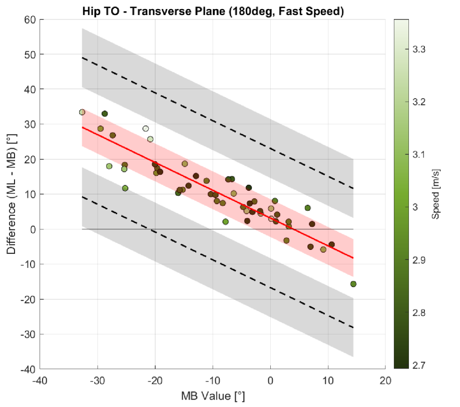

Supplementary Figure 475: Extended BA Plot for TO hip angle - Transverse Plane (180deg, Fast)

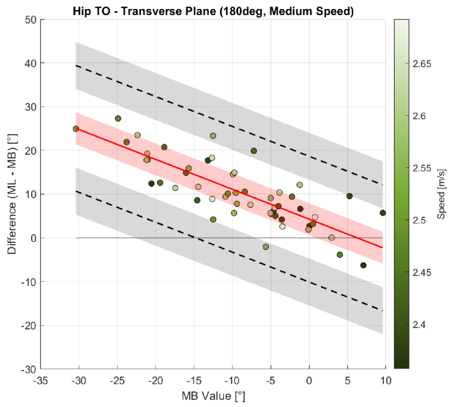

Supplementary Figure 476: Extended BA Plot for TO hip angle - Transverse Plane (180deg, Medium)

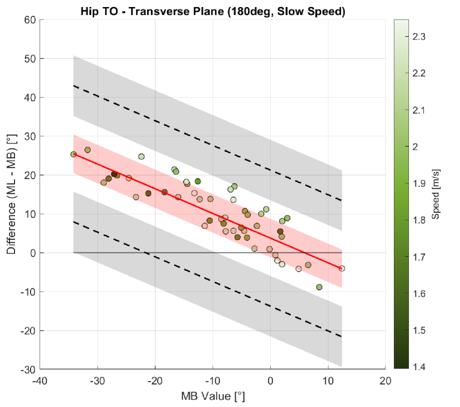

Supplementary Figure 477: Extended BA Plot for TO hip angle - Transverse Plane (180deg, Slow)

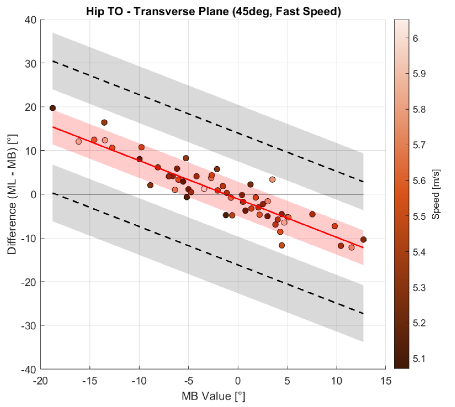

Supplementary Figure 478: Extended BA Plot for TO hip angle - Transverse Plane (45deg, Fast)

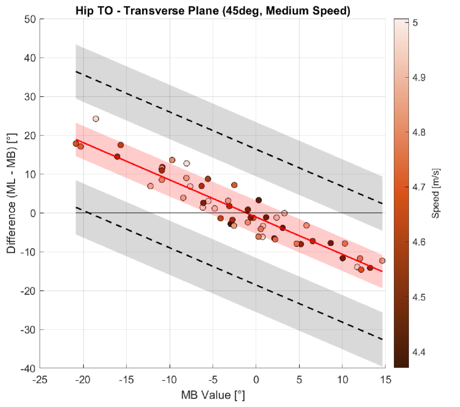

Supplementary Figure 479: Extended BA Plot for TO hip angle - Transverse Plane (45deg, Medium)

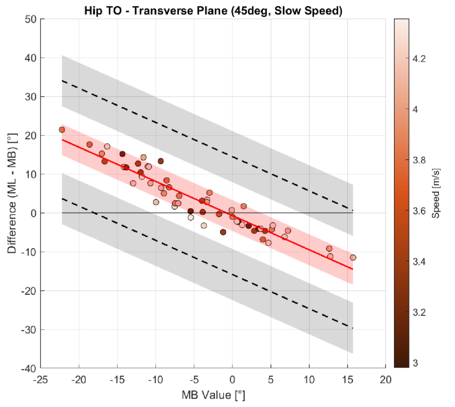

Supplementary Figure 480: Extended BA Plot for TO hip angle - Transverse Plane (45deg, Slow)

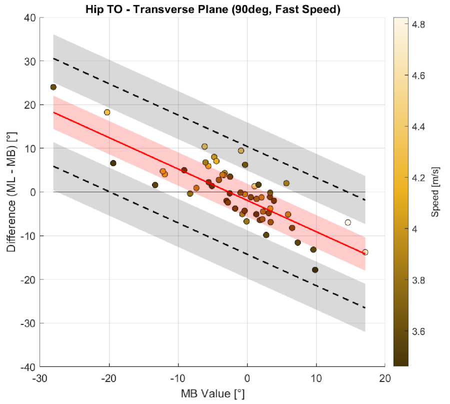

Supplementary Figure 481: Extended BA Plot for TO hip angle - Transverse Plane (90deg, Fast)

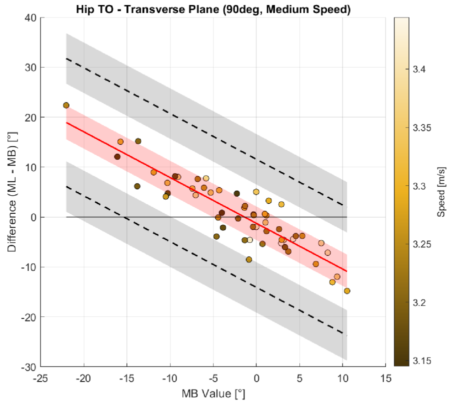

Supplementary Figure 482: Extended BA Plot for TO hip angle - Transverse Plane (90deg, Medium)

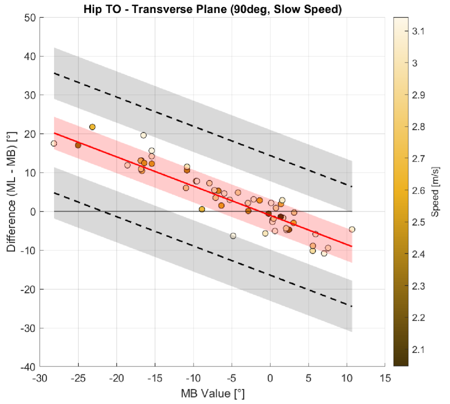

Supplementary Figure 483: Extended BA Plot for TO hip angle - Transverse Plane (90deg, Slow)

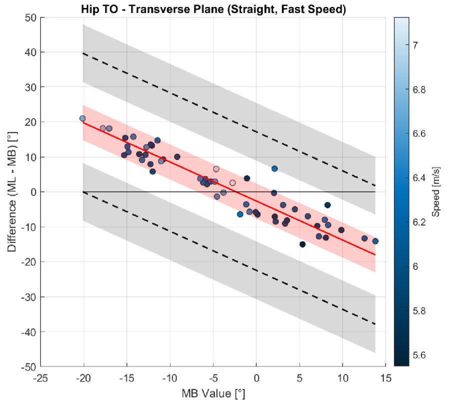

Supplementary Figure 484: Extended BA Plot for TO hip angle - Transverse Plane (Straight, Fast)

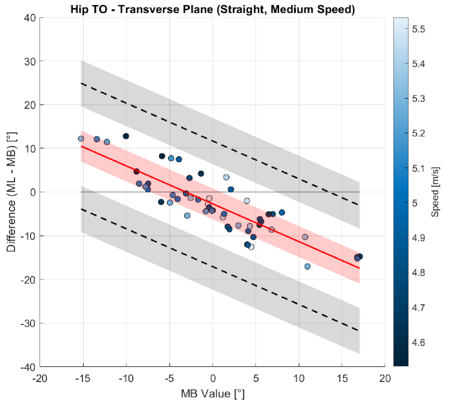

Supplementary Figure 485: Extended BA Plot for TO hip angle - Transverse Plane (Straight, Medium)

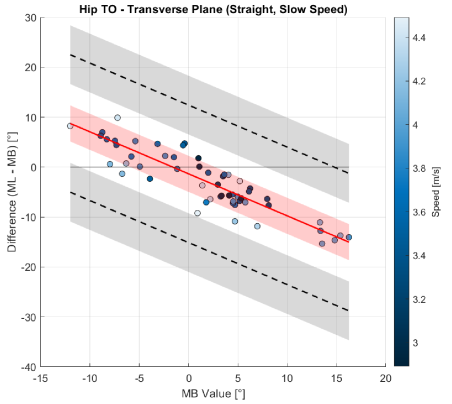

Supplementary Figure 486: Extended BA Plot for TO hip angle - Transverse Plane (Straight, Slow)

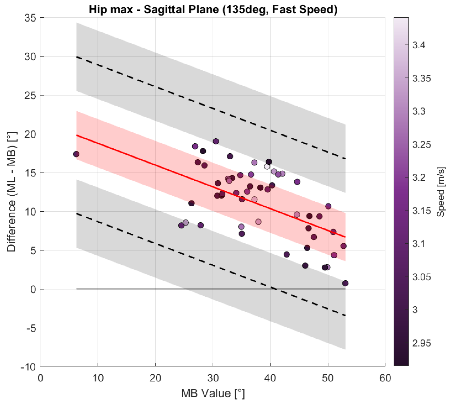

Supplementary Figure 487: Extended BA Plot for maximal hip angle - Sagittal Plane (135deg, Fast)

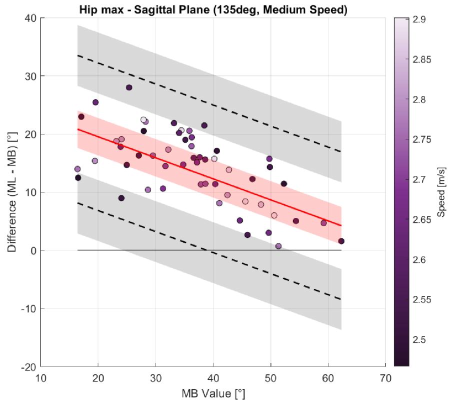

Supplementary Figure 488: Extended BA Plot for maximal hip angle - Sagittal Plane (135deg, Medium)

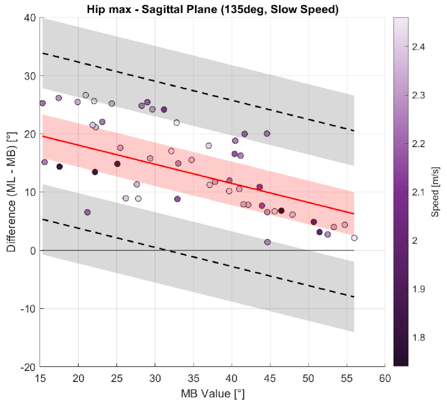

Supplementary Figure 489: Extended BA Plot for maximal hip angle - Sagittal Plane (135deg, Slow)

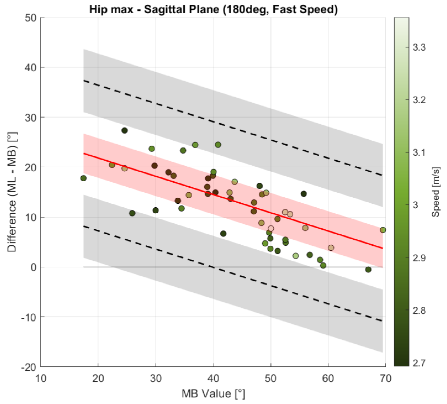

Supplementary Figure 490: Extended BA Plot for maximal hip angle - Sagittal Plane (180deg, Fast)

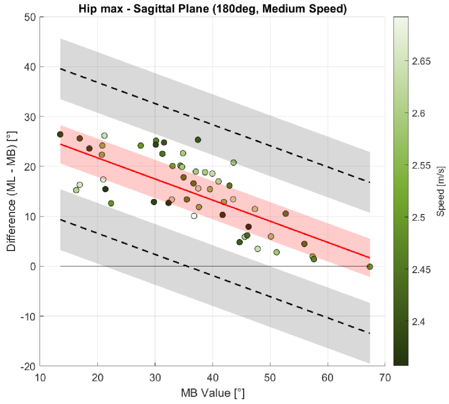

Supplementary Figure 491: Extended BA Plot for maximal hip angle - Sagittal Plane (180deg, Medium)

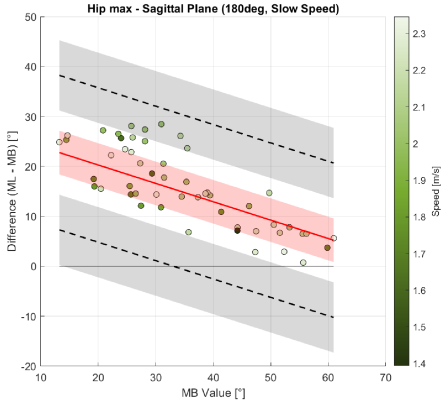

Supplementary Figure 492: Extended BA Plot for maximal hip angle - Sagittal Plane (180deg, Slow)

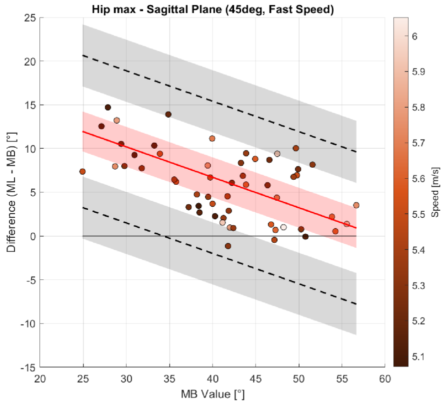

Supplementary Figure 493: Extended BA Plot for maximal hip angle - Sagittal Plane (45deg, Fast)

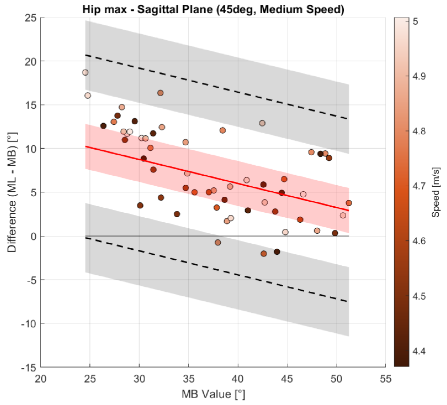

Supplementary Figure 494: Extended BA Plot for maximal hip angle - Sagittal Plane (45deg, Medium)

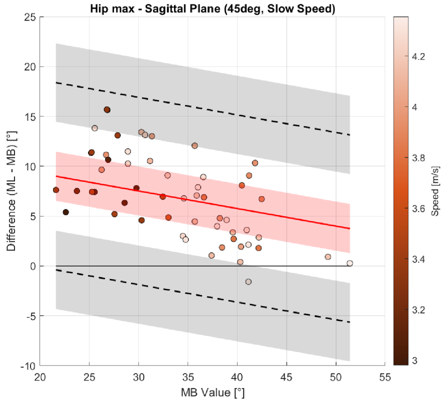

Supplementary Figure 495: Extended BA Plot for maximal hip angle - Sagittal Plane (45deg, Slow)

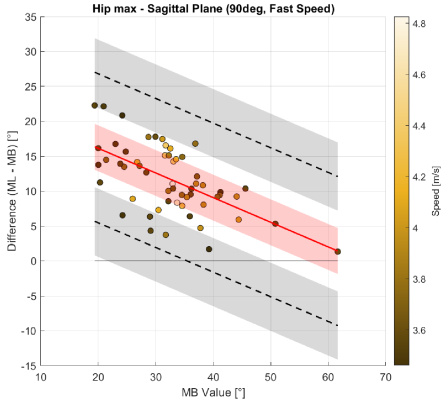

Supplementary Figure 496: Extended BA Plot for maximal hip angle - Sagittal Plane (90deg, Fast)

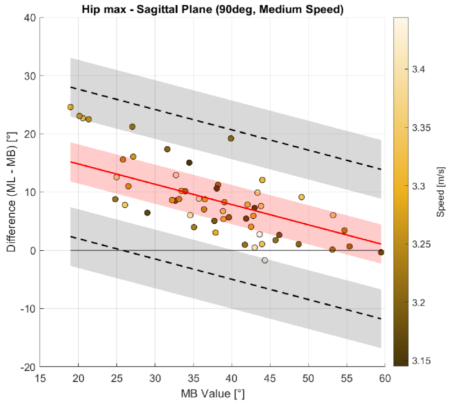

Supplementary Figure 497: Extended BA Plot for maximal hip angle - Sagittal Plane (90deg, Medium)

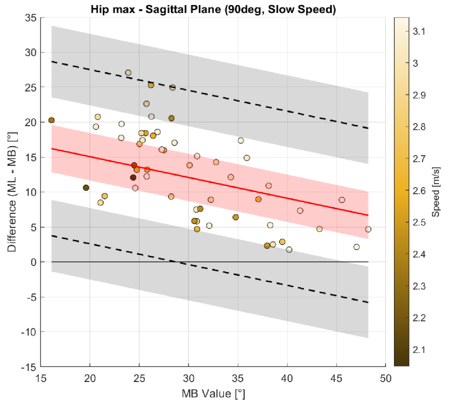

Supplementary Figure 498: Extended BA Plot for maximal hip angle - Sagittal Plane (90deg, Slow)

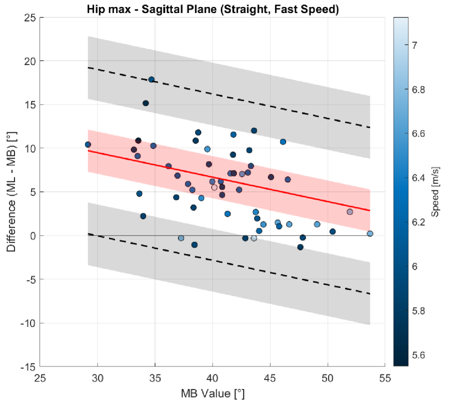

Supplementary Figure 499: Extended BA Plot for maximal hip angle - Sagittal Plane (Straight, Fast)

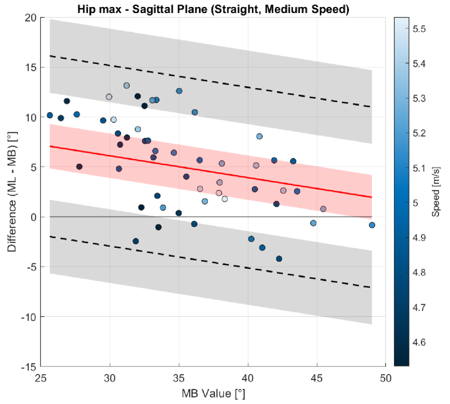

Supplementary Figure 500: Extended BA Plot for maximal hip angle - Sagittal Plane (Straight, Medium)

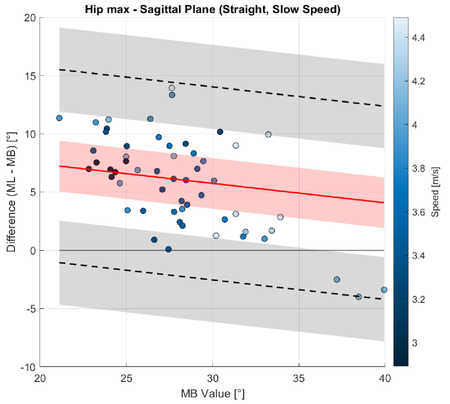

Supplementary Figure 501: Extended BA Plot for maximal hip angle - Sagittal Plane (Straight, Slow)

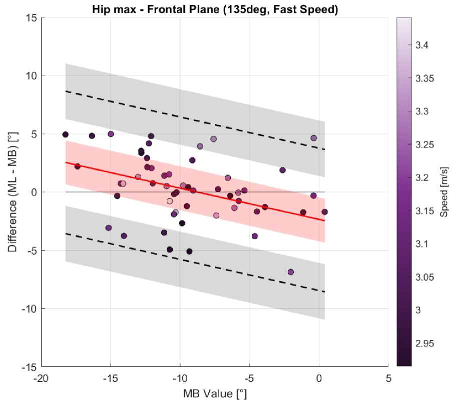

Supplementary Figure 502: Extended BA Plot for maximal hip angle - Frontal Plane (135deg, Fast)

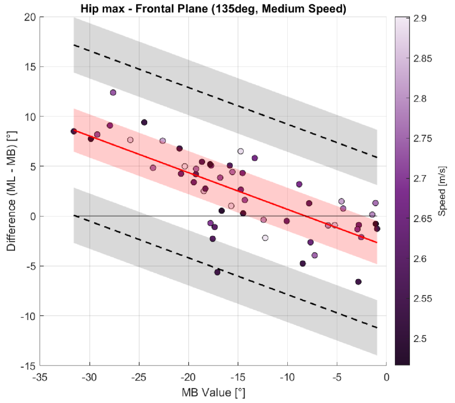

Supplementary Figure 503: Extended BA Plot for maximal hip angle - Frontal Plane (135deg, Medium)

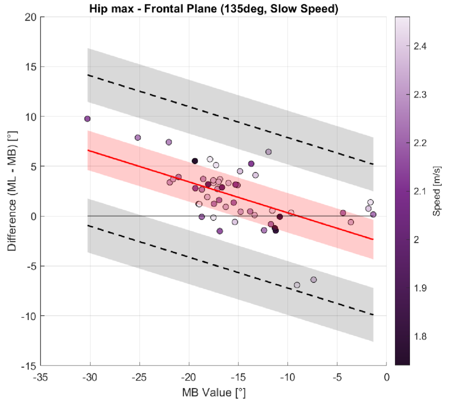

Supplementary Figure 504: Extended BA Plot for maximal hip angle - Frontal Plane (135deg, Slow)

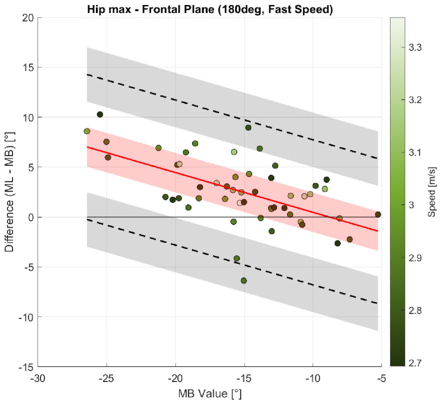

Supplementary Figure 505: Extended BA Plot for maximal hip angle - Frontal Plane (180deg, Fast)

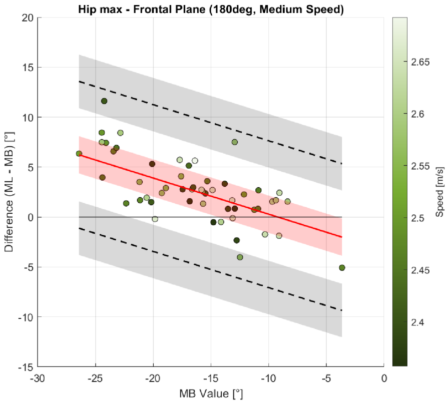

Supplementary Figure 506: Extended BA Plot for maximal hip angle - Frontal Plane (180deg, Medium)

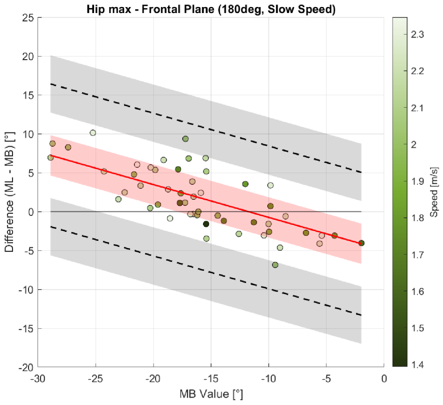

Supplementary Figure 507: Extended BA Plot for maximal hip angle - Frontal Plane (180deg, Slow)

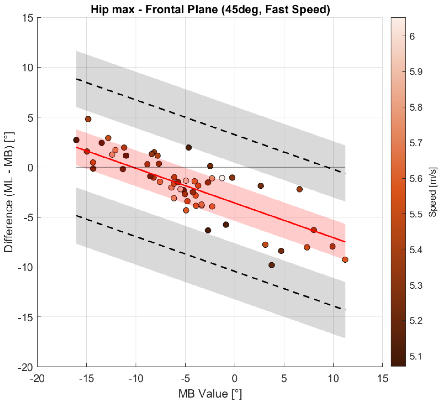

Supplementary Figure 508: Extended BA Plot for maximal hip angle - Frontal Plane (45deg, Fast)

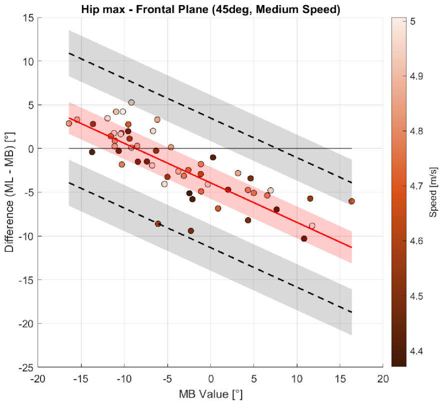

Supplementary Figure 509: Extended BA Plot for maximal hip angle - Frontal Plane (45deg, Medium)

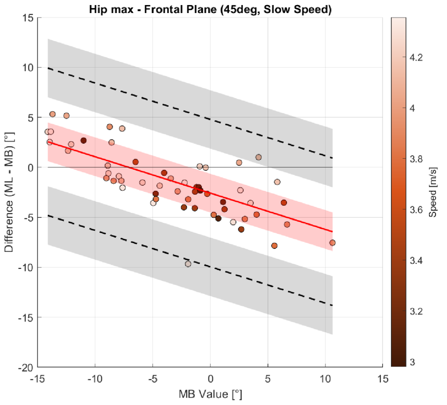

Supplementary Figure 510: Extended BA Plot for maximal hip angle - Frontal Plane (45deg, Slow)

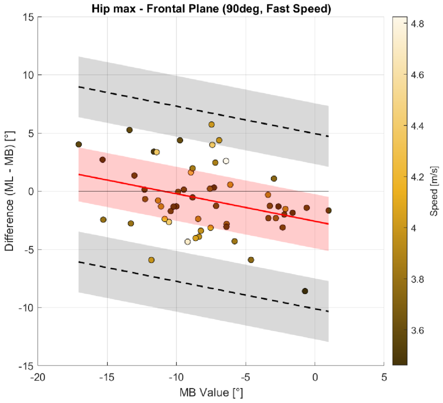

Supplementary Figure 511: Extended BA Plot for maximal hip angle - Frontal Plane (90deg, Fast)

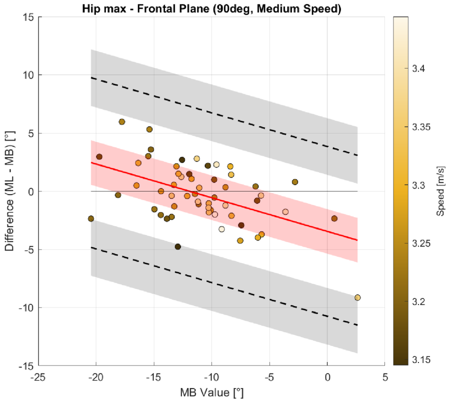

Supplementary Figure 512: Extended BA Plot for maximal hip angle - Frontal Plane (90deg, Medium)

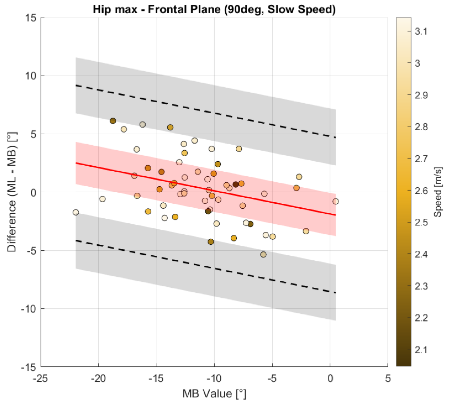

Supplementary Figure 513: Extended BA Plot for maximal hip angle - Frontal Plane (90deg, Slow)

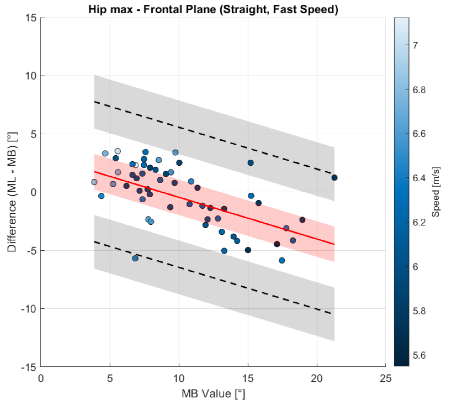

Supplementary Figure 514: Extended BA Plot for maximal hip angle - Frontal Plane (Straight, Fast)

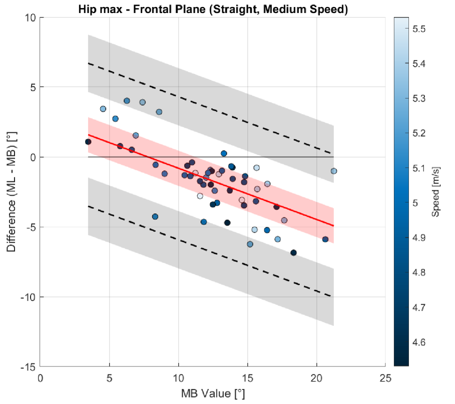

Supplementary Figure 515: Extended BA Plot for maximal hip angle - Frontal Plane (Straight, Medium)

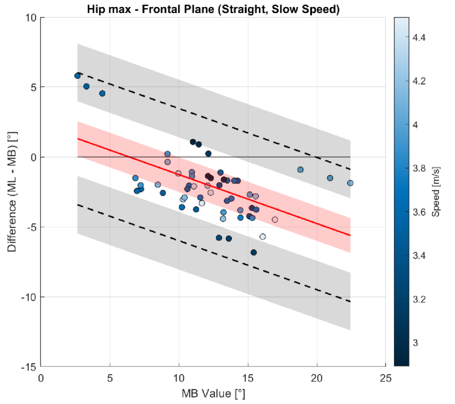

Supplementary Figure 516: Extended BA Plot for maximal hip angle - Frontal Plane (Straight, Slow)

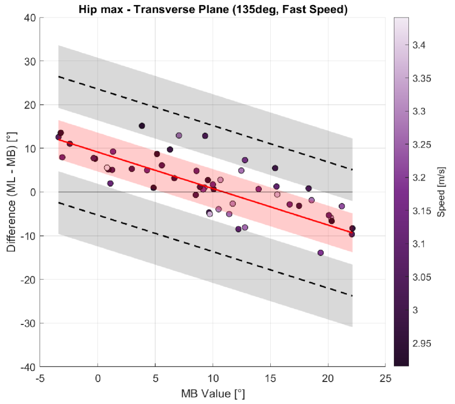

Supplementary Figure 517: Extended BA Plot for maximal hip angle - Transverse Plane (135deg, Fast)

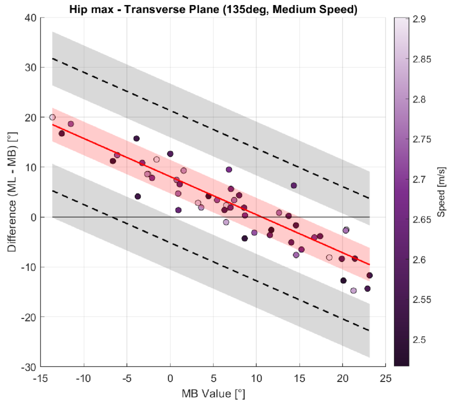

Supplementary Figure 518: Extended BA Plot for maximal hip angle - Transverse Plane (135deg, Medium)

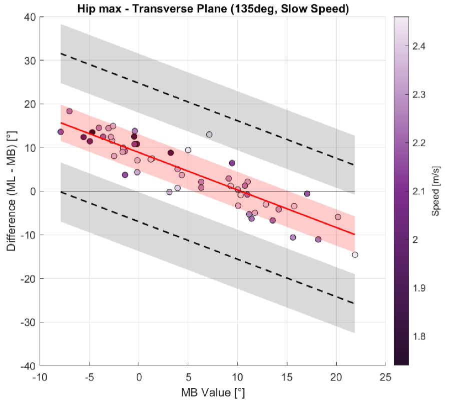

Supplementary Figure 519: Extended BA Plot for maximal hip angle - Transverse Plane (135deg, Slow)

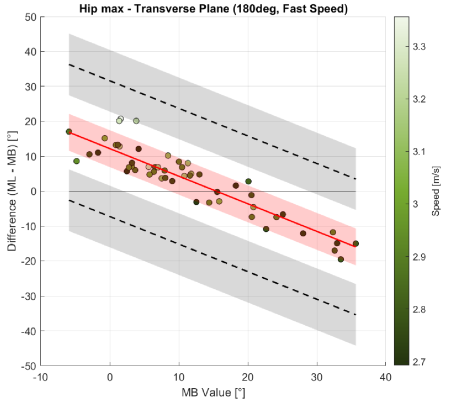

Supplementary Figure 520: Extended BA Plot for maximal hip angle - Transverse Plane (180deg, Fast)

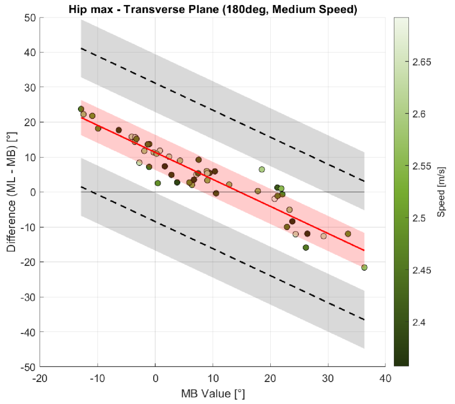

Supplementary Figure 521: Extended BA Plot for maximal hip angle - Transverse Plane (180deg, Medium)

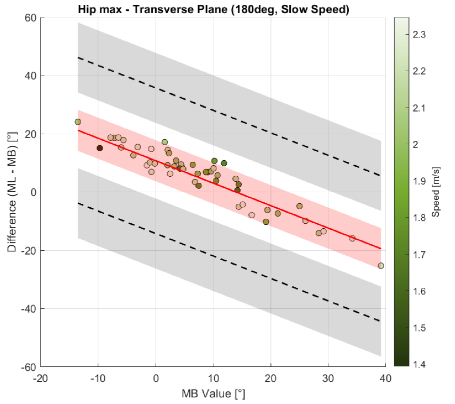

Supplementary Figure 522: Extended BA Plot for maximal hip angle - Transverse Plane (180deg, Slow)

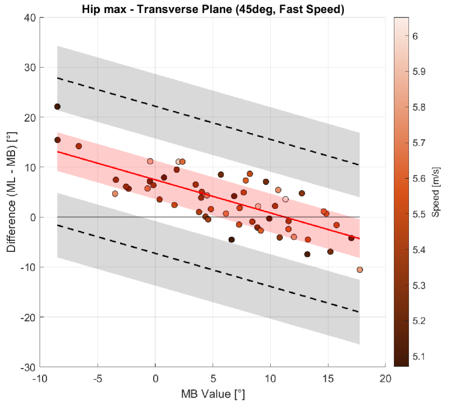

Supplementary Figure 523: Extended BA Plot for maximal hip angle - Transverse Plane (45deg, Fast)

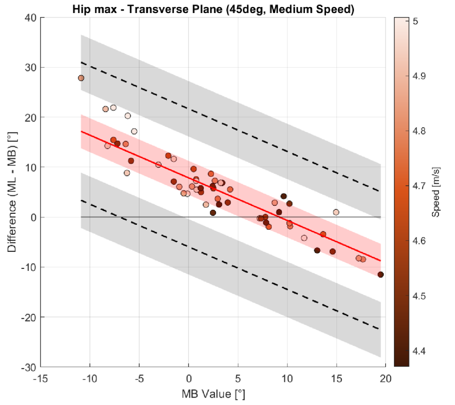

Supplementary Figure 524: Extended BA Plot for maximal hip angle - Transverse Plane (45deg, Medium)

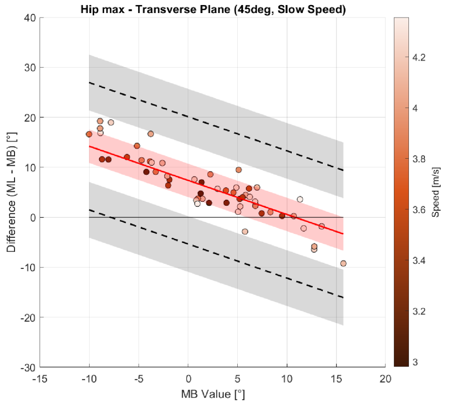

Supplementary Figure 525: Extended BA Plot for maximal hip angle - Transverse Plane (45deg, Slow)

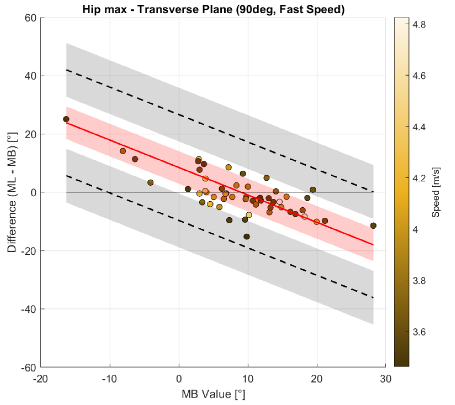

Supplementary Figure 526: Extended BA Plot for maximal hip angle - Transverse Plane (90deg, Fast)

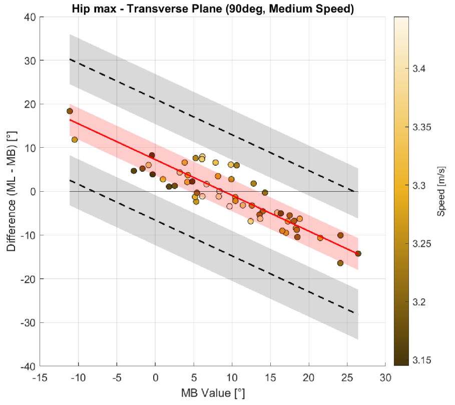

Supplementary Figure 527: Extended BA Plot for maximal hip angle - Transverse Plane (90deg, Medium)

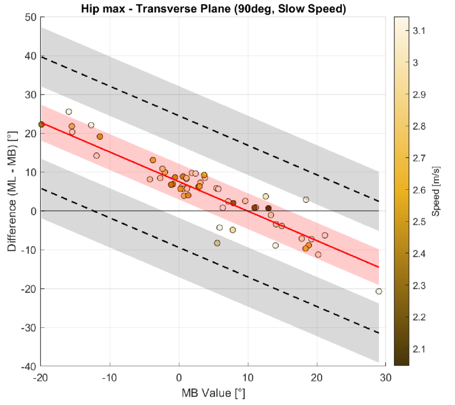

Supplementary Figure 528: Extended BA Plot for maximal hip angle - Transverse Plane (90deg, Slow)

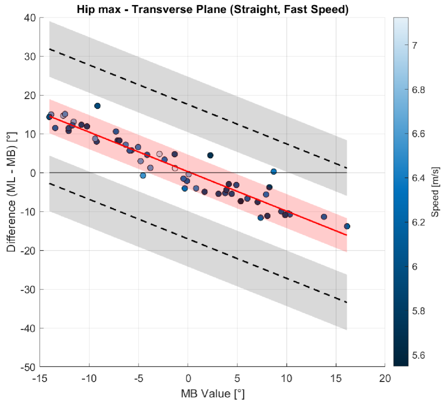

Supplementary Figure 529: Extended BA Plot for maximal hip angle - Transverse Plane (Straight, Fast)

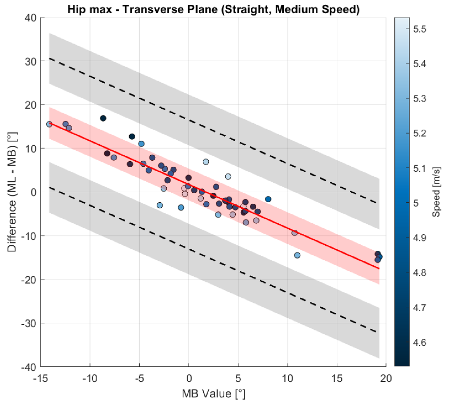

Supplementary Figure 530: Extended BA Plot for maximal hip angle - Transverse Plane (Straight, Medium)

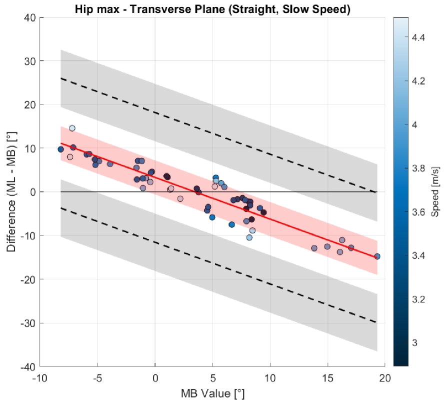

Supplementary Figure 531: Extended BA Plot for maximal hip angle - Transverse Plane (Straight, Slow)

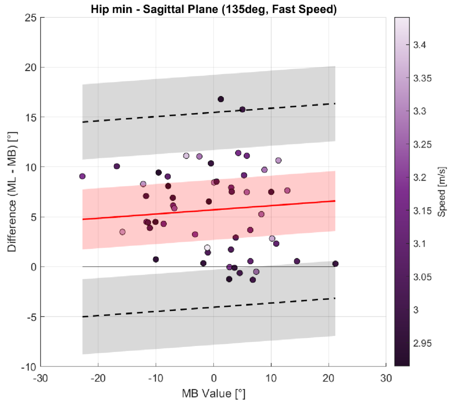

Supplementary Figure 532: Extended BA Plot for minimal hip angle - Sagittal Plane (135deg, Fast)

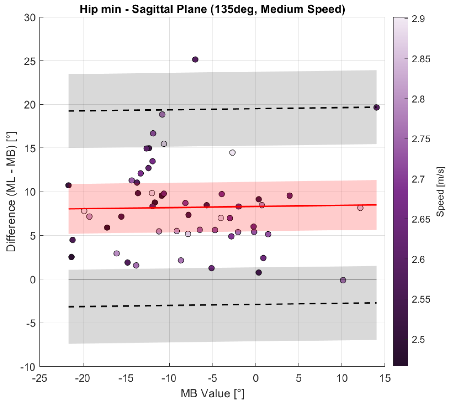

Supplementary Figure 533: Extended BA Plot for minimal hip angle - Sagittal Plane (135deg, Medium)

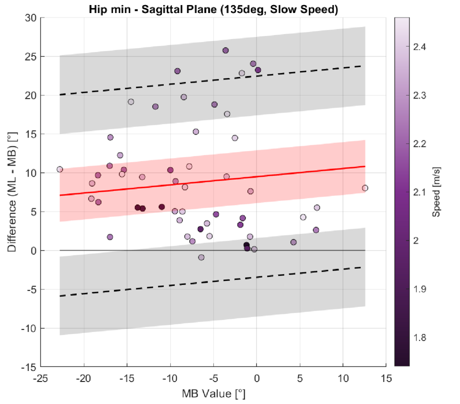

Supplementary Figure 534: Extended BA Plot for minimal hip angle - Sagittal Plane (135deg, Slow)

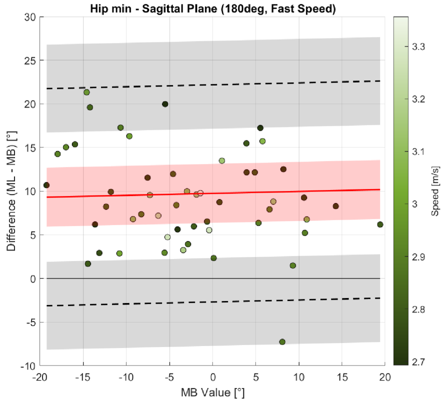

Supplementary Figure 535: Extended BA Plot for minimal hip angle - Sagittal Plane (180deg, Fast)

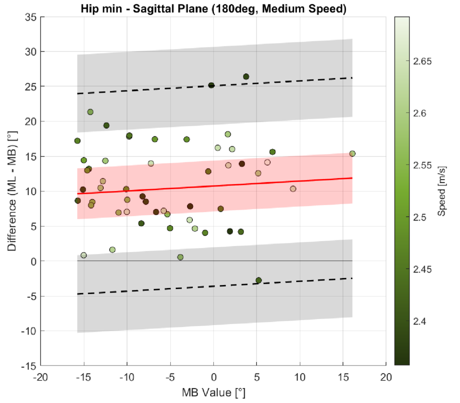

Supplementary Figure 536: Extended BA Plot for minimal hip angle - Sagittal Plane (180deg, Medium)

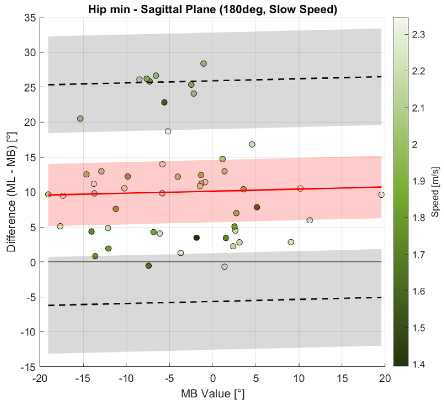

Supplementary Figure 537: Extended BA Plot for minimal hip angle - Sagittal Plane (180deg, Slow)

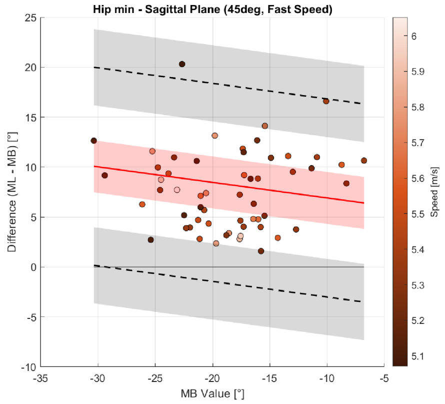

Supplementary Figure 538: Extended BA Plot for minimal hip angle - Sagittal Plane (45deg, Fast)

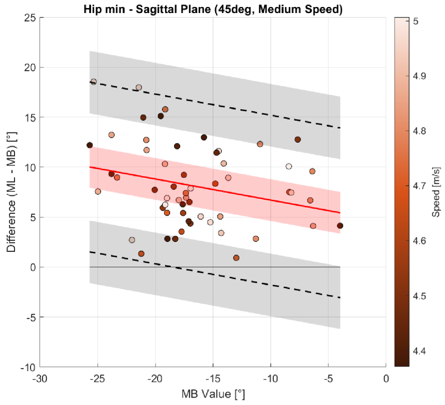

Supplementary Figure 539: Extended BA Plot for minimal hip angle - Sagittal Plane (45deg, Medium)

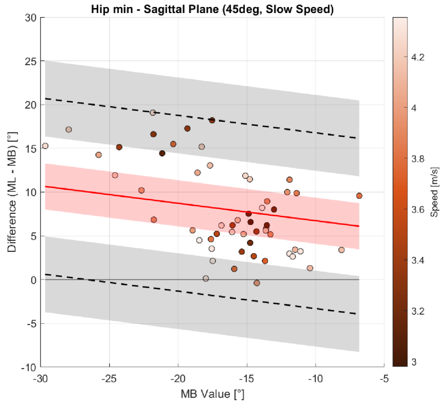

Supplementary Figure 540: Extended BA Plot for minimal hip angle - Sagittal Plane (45deg, Slow)

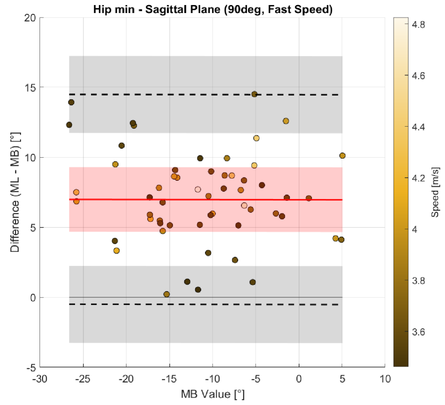

Supplementary Figure 541: Extended BA Plot for minimal hip angle - Sagittal Plane (90deg, Fast)

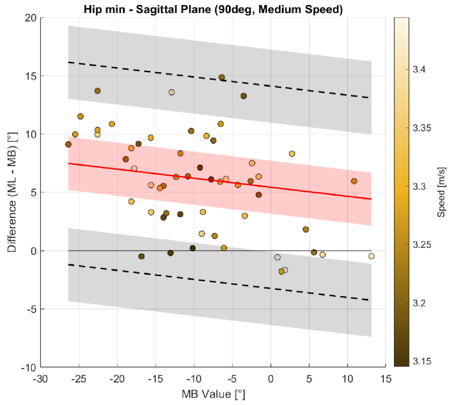

Supplementary Figure 542: Extended BA Plot for minimal hip angle - Sagittal Plane (90deg, Medium)

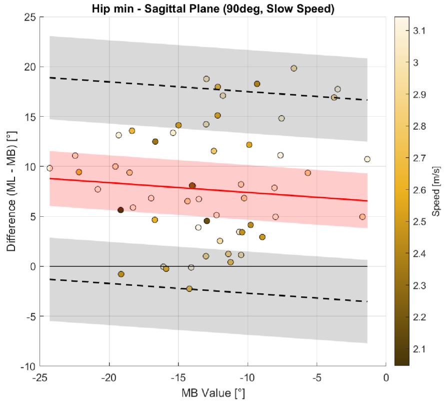

Supplementary Figure 543: Extended BA Plot for minimal hip angle - Sagittal Plane (90deg, Slow)

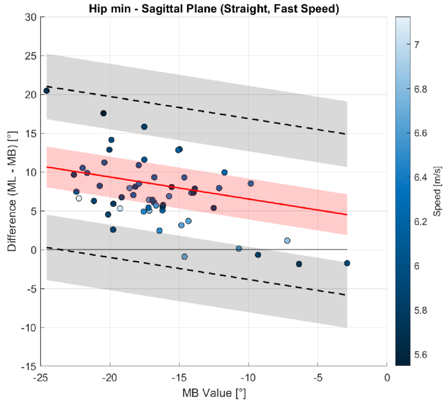

Supplementary Figure 544: Extended BA Plot for minimal hip angle - Sagittal Plane (Straight, Fast)

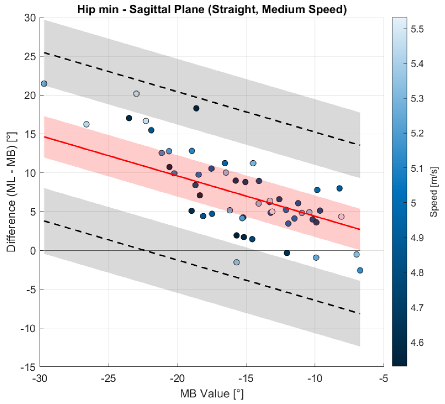

Supplementary Figure 545: Extended BA Plot for minimal hip angle - Sagittal Plane (Straight, Medium)

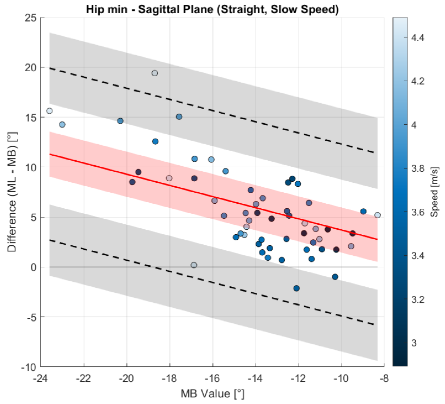

Supplementary Figure 546: Extended BA Plot for minimal hip angle - Sagittal Plane (Straight, Slow)

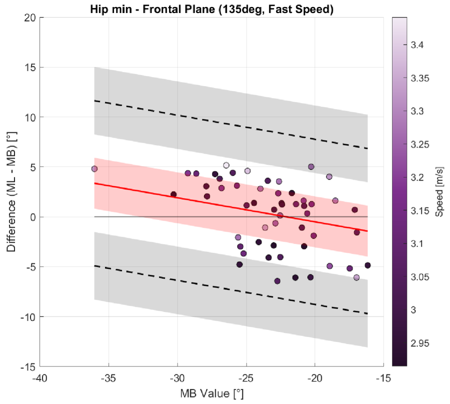

Supplementary Figure 547: Extended BA Plot for minimal hip angle - Frontal Plane (135deg, Fast)

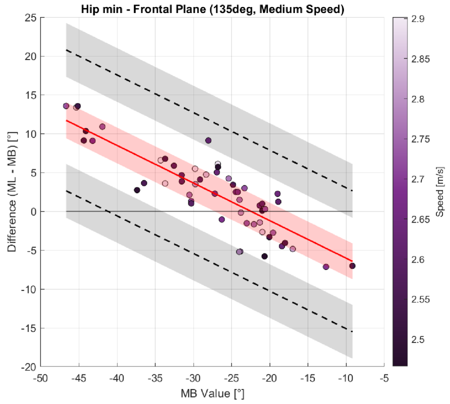

Supplementary Figure 548: Extended BA Plot for minimal hip angle - Frontal Plane (135deg, Medium)

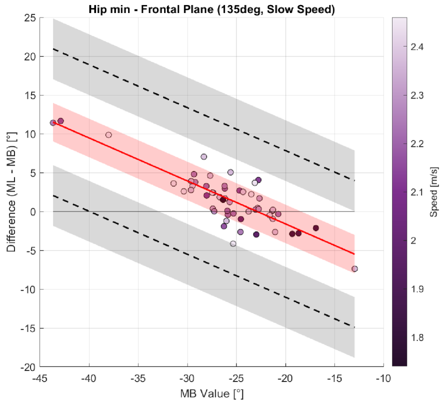

Supplementary Figure 549: Extended BA Plot for minimal hip angle - Frontal Plane (135deg, Slow)

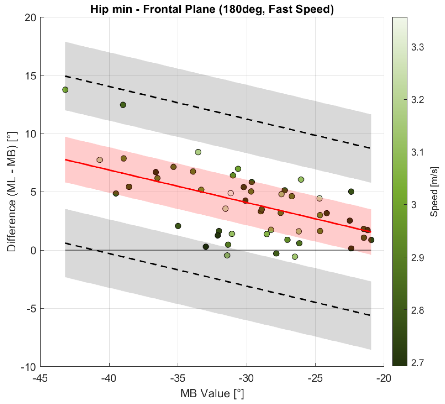

Supplementary Figure 550: Extended BA Plot for minimal hip angle - Frontal Plane (180deg, Fast)

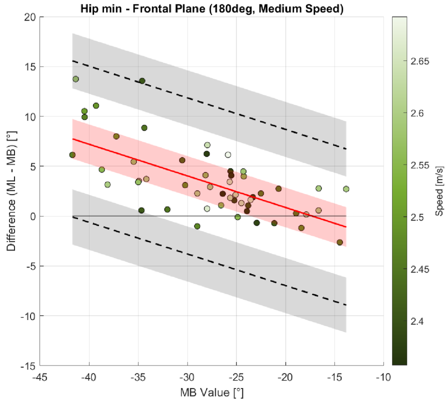

Supplementary Figure 551: Extended BA Plot for minimal hip angle - Frontal Plane (180deg, Medium)

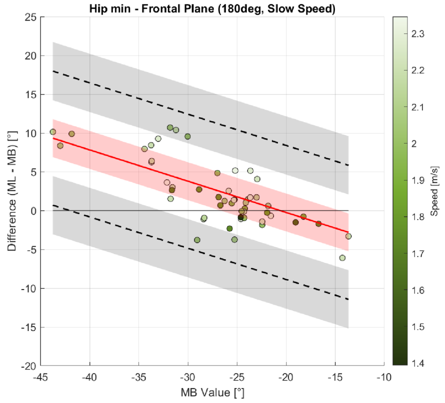

Supplementary Figure 552: Extended BA Plot for minimal hip angle - Frontal Plane (180deg, Slow)

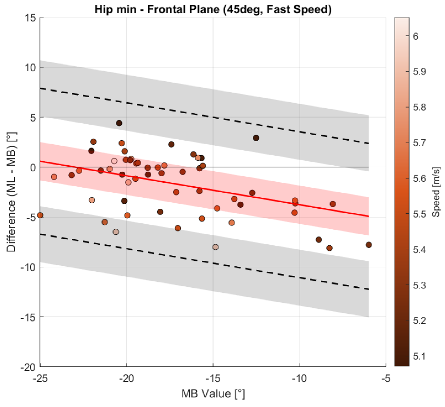

Supplementary Figure 553: Extended BA Plot for minimal hip angle - Frontal Plane (45deg, Fast)

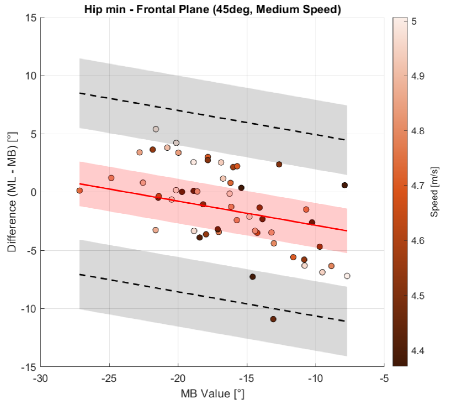

Supplementary Figure 554: Extended BA Plot for minimal hip angle - Frontal Plane (45deg, Medium)

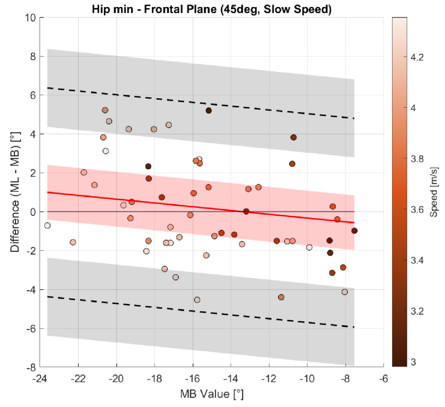

Supplementary Figure 555: Extended BA Plot for minimal hip angle - Frontal Plane (45deg, Slow)

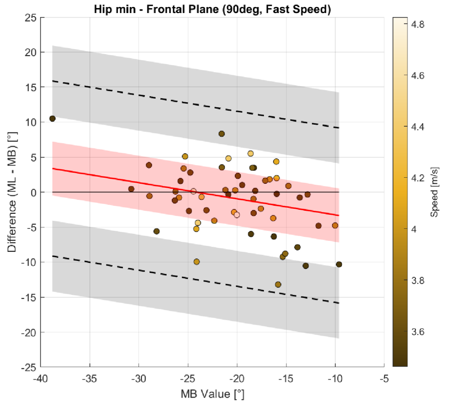

Supplementary Figure 556: Extended BA Plot for minimal hip angle - Frontal Plane (90deg, Fast)

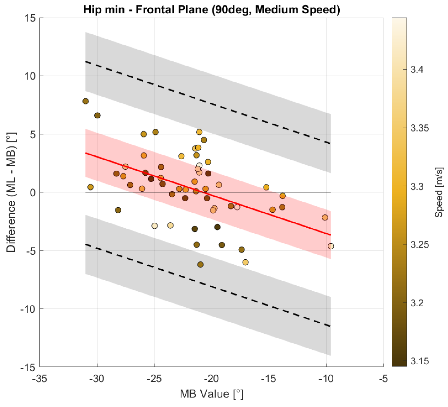

Supplementary Figure 557: Extended BA Plot for minimal hip angle - Frontal Plane (90deg, Medium)

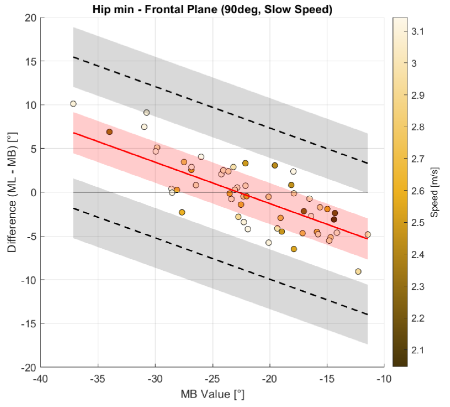

Supplementary Figure 558: Extended BA Plot for minimal hip angle - Frontal Plane (90deg, Slow)

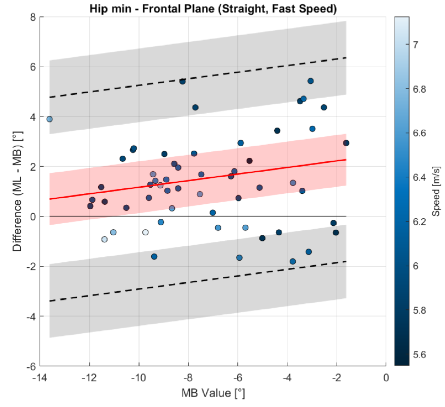

Supplementary Figure 559: Extended BA Plot for minimal hip angle - Frontal Plane (Straight, Fast)

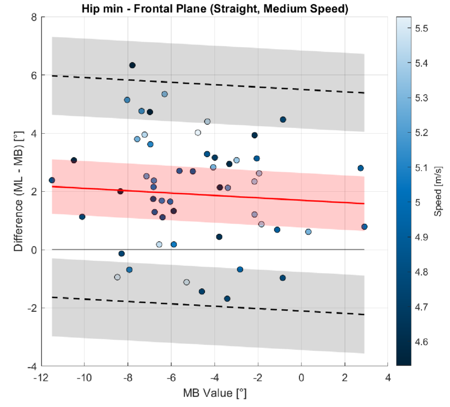

Supplementary Figure 560: Extended BA Plot for minimal hip angle - Frontal Plane (Straight, Medium)

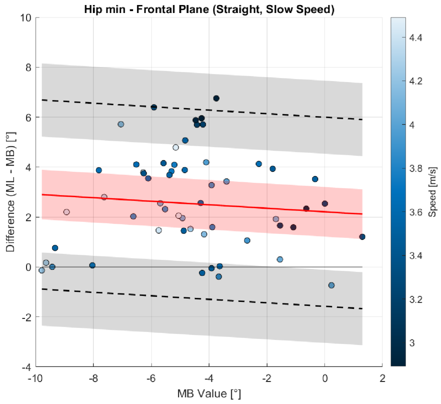

Supplementary Figure 561: Extended BA Plot for minimal hip angle - Frontal Plane (Straight, Slow)

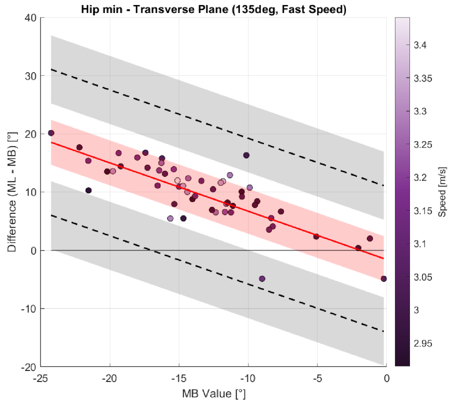

Supplementary Figure 562: Extended BA Plot for minimal hip angle - Transverse Plane (135deg, Fast)

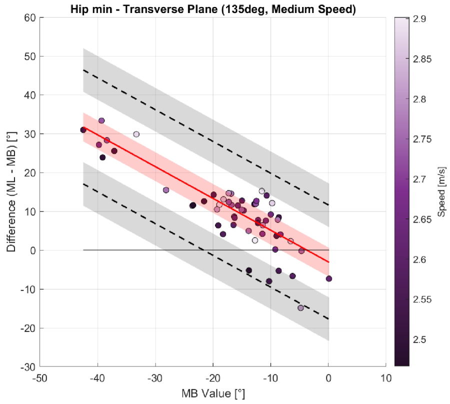

Supplementary Figure 563: Extended BA Plot for minimal hip angle - Transverse Plane (135deg, Medium)

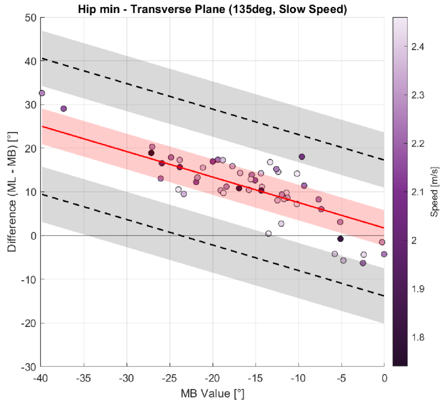

Supplementary Figure 564: Extended BA Plot for minimal hip angle - Transverse Plane (135deg, Slow)

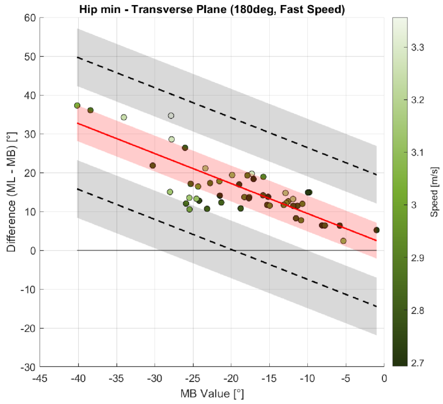

Supplementary Figure 565: Extended BA Plot for minimal hip angle - Transverse Plane (180deg, Fast)

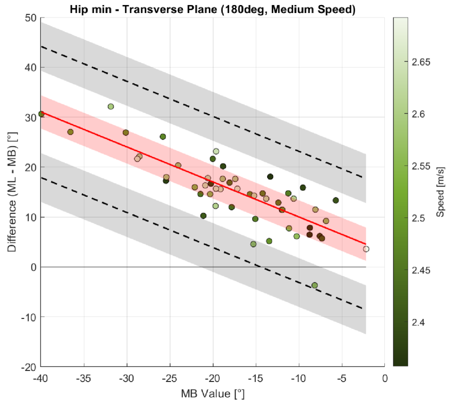

Supplementary Figure 566: Extended BA Plot for minimal hip angle - Transverse Plane (180deg, Medium)

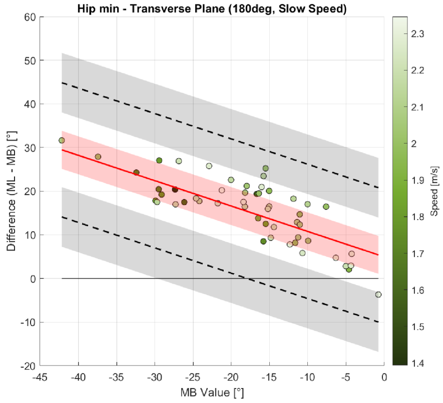

Supplementary Figure 567: Extended BA Plot for minimal hip angle - Transverse Plane (180deg, Slow)

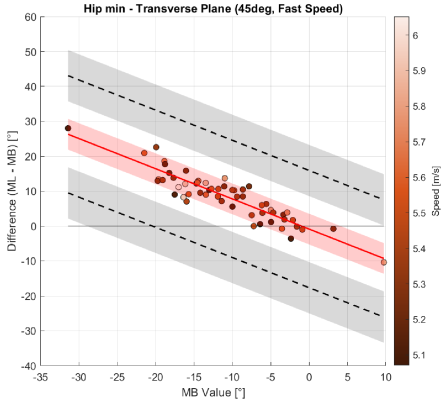

Supplementary Figure 568: Extended BA Plot for minimal hip angle - Transverse Plane (45deg, Fast)

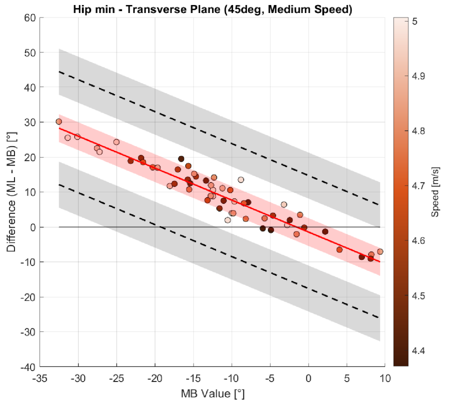

Supplementary Figure 569: Extended BA Plot for minimal hip angle - Transverse Plane (45deg, Medium)

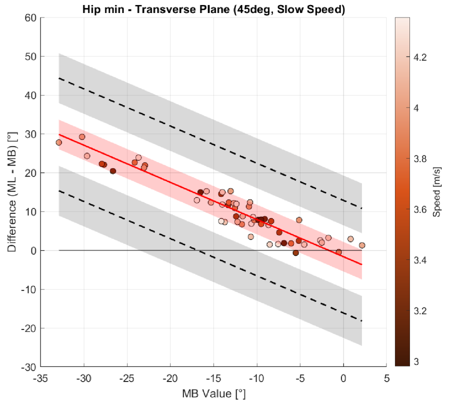

Supplementary Figure 570: Extended BA Plot for minimal hip angle - Transverse Plane (45deg, Slow)

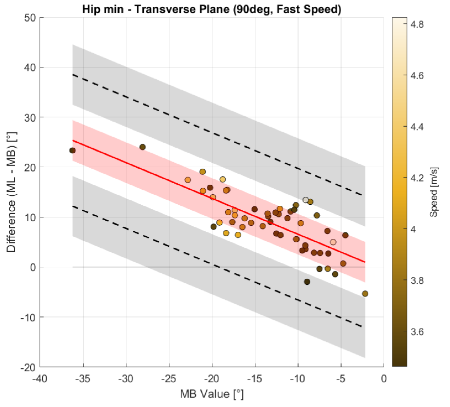

Supplementary Figure 571: Extended BA Plot for minimal hip angle - Transverse Plane (90deg, Fast)

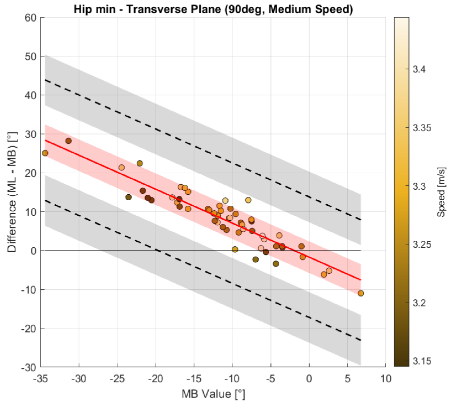

Supplementary Figure 572: Extended BA Plot for minimal hip angle - Transverse Plane (90deg, Medium)

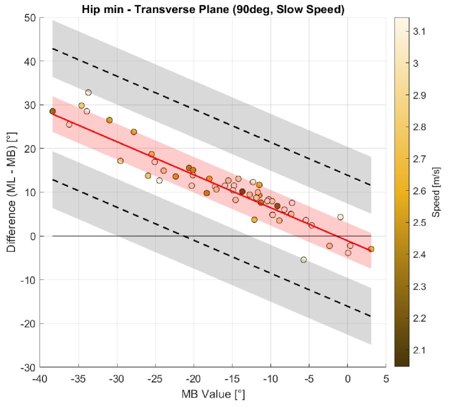

Supplementary Figure 573: Extended BA Plot for minimal hip angle - Transverse Plane (90deg, Slow)

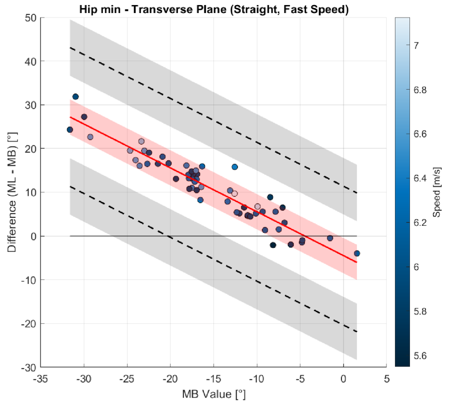

Supplementary Figure 574: Extended BA Plot for minimal hip angle - Transverse Plane (Straight, Fast)

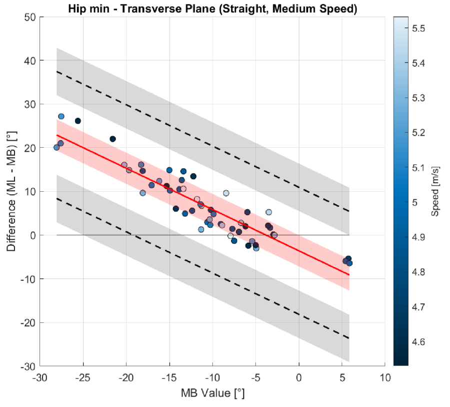

Supplementary Figure 575: Extended BA Plot for minimal hip angle - Transverse Plane (Straight, Medium)

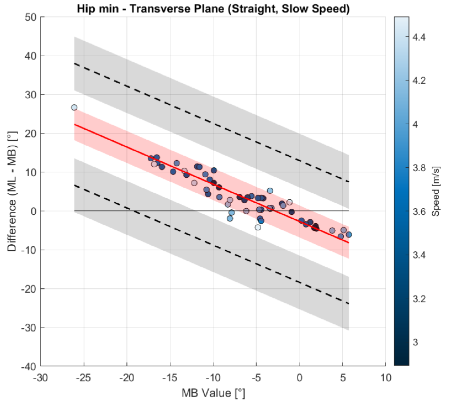

Supplementary Figure 576: Extended BA Plot for minimal hip angle - Transverse Plane (Straight, Slow)

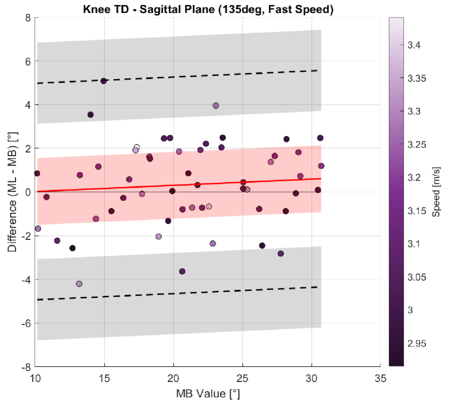

Supplementary Figure 577: Extended BA Plot for TD knee angle - Sagittal Plane (135deg, Fast)

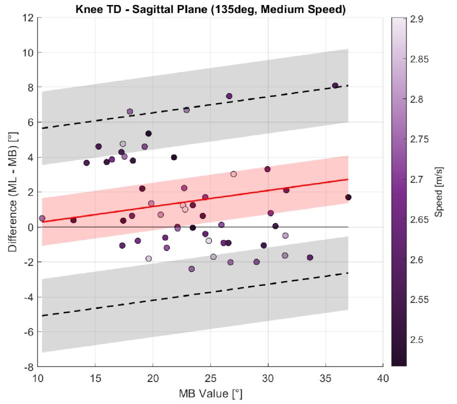

Supplementary Figure 578: Extended BA Plot for TD knee angle - Sagittal Plane (135deg, Medium)

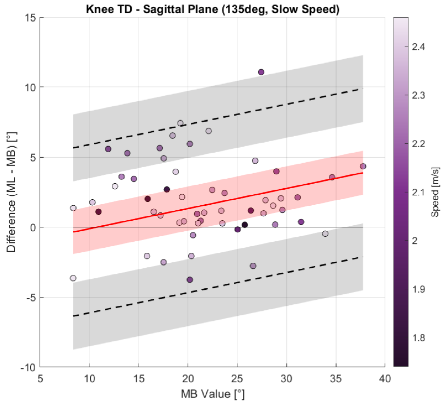

Supplementary Figure 579: Extended BA Plot for TD knee angle - Sagittal Plane (135deg, Slow)

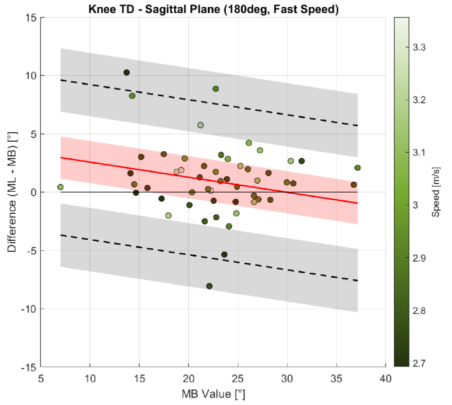

Supplementary Figure 580: Extended BA Plot for TD knee angle - Sagittal Plane (180deg, Fast)

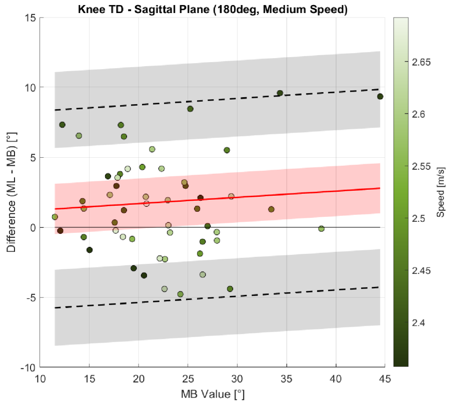

Supplementary Figure 581: Extended BA Plot for TD knee angle - Sagittal Plane (180deg, Medium)

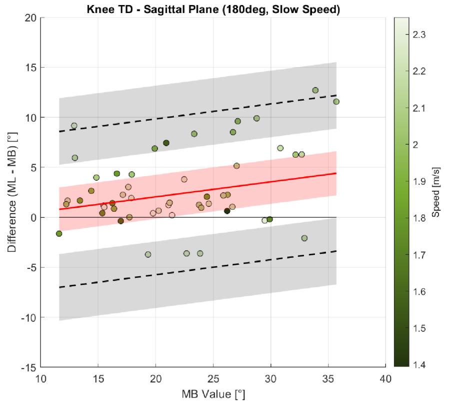

Supplementary Figure 582: Extended BA Plot for TD knee angle - Sagittal Plane (180deg, Slow)

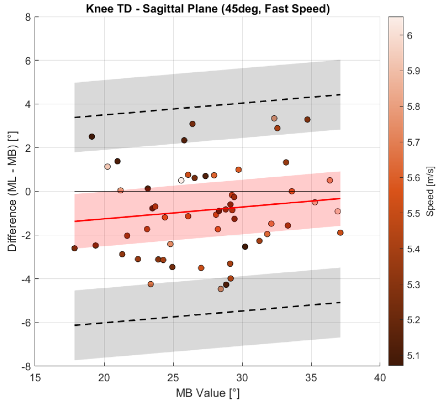

Supplementary Figure 583: Extended BA Plot for TD knee angle - Sagittal Plane (45deg, Fast)

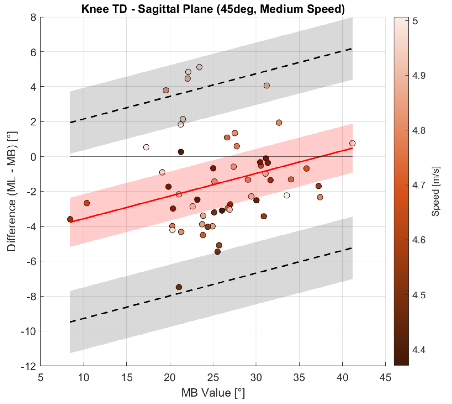

Supplementary Figure 584: Extended BA Plot for TD knee angle - Sagittal Plane (45deg, Medium)

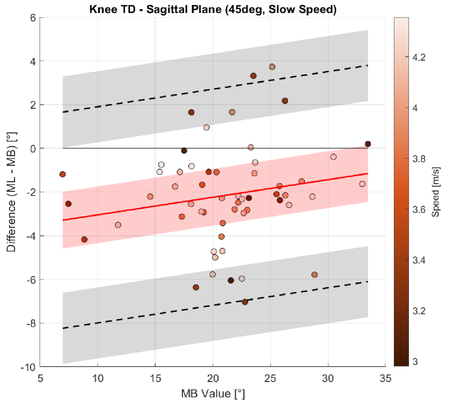

Supplementary Figure 585: Extended BA Plot for TD knee angle - Sagittal Plane (45deg, Slow)

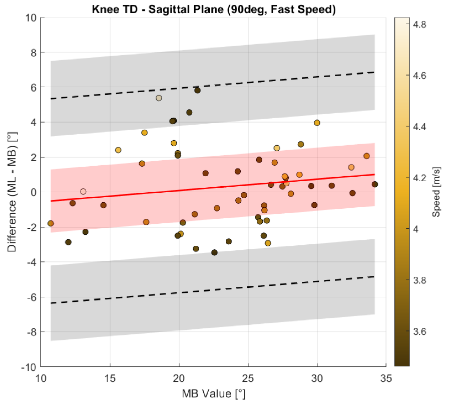

Supplementary Figure 586: Extended BA Plot for TD knee angle - Sagittal Plane (90deg, Fast)

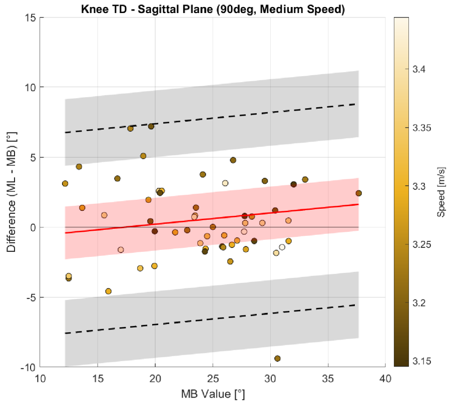

Supplementary Figure 587: Extended BA Plot for TD knee angle - Sagittal Plane (90deg, Medium)

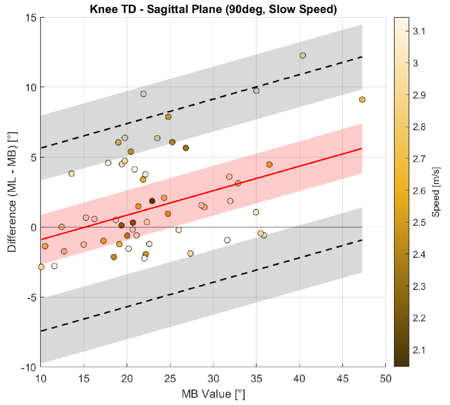

Supplementary Figure 588: Extended BA Plot for TD knee angle - Sagittal Plane (90deg, Slow)

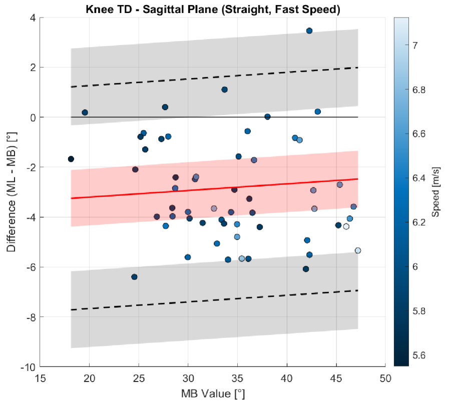

Supplementary Figure 589: Extended BA Plot for TD knee angle - Sagittal Plane (Straight, Fast)

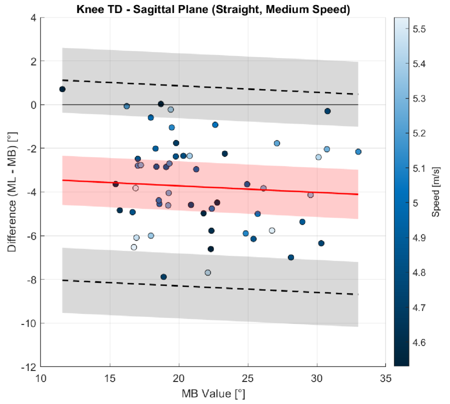

Supplementary Figure 590: Extended BA Plot for TD knee angle - Sagittal Plane (Straight, Medium)

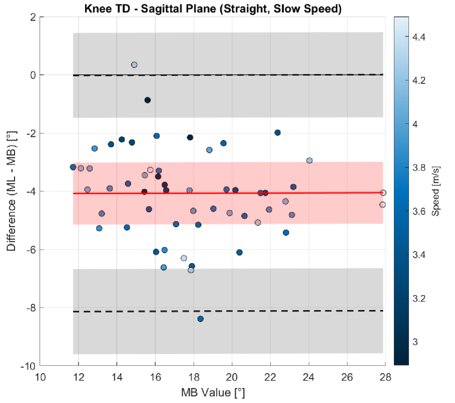

Supplementary Figure 591: Extended BA Plot for TD knee angle - Sagittal Plane (Straight, Slow)

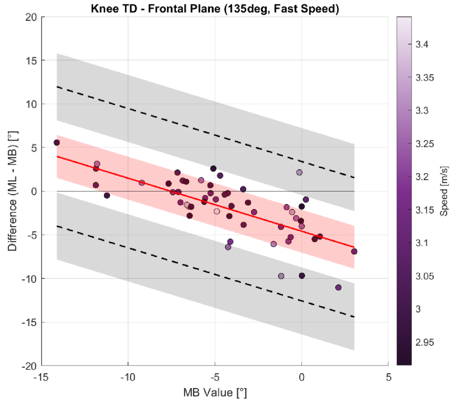

Supplementary Figure 592: Extended BA Plot for TD knee angle - Frontal Plane (135deg, Fast)

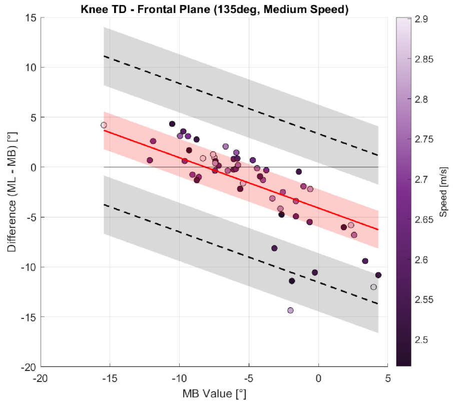

Supplementary Figure 593: Extended BA Plot for TD knee angle - Frontal Plane (135deg, Medium)

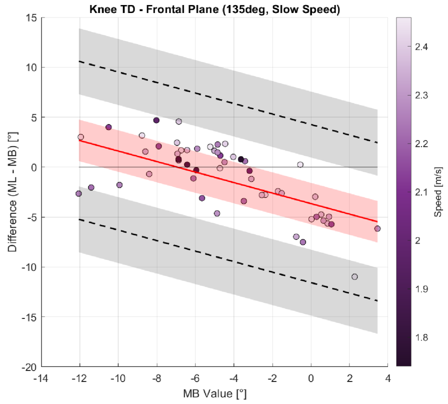

Supplementary Figure 594: Extended BA Plot for TD knee angle - Frontal Plane (135deg, Slow)

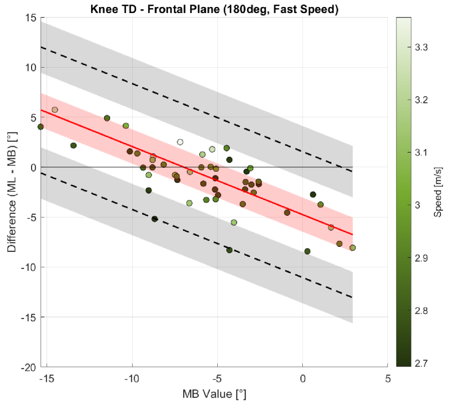

Supplementary Figure 595: Extended BA Plot for TD knee angle - Frontal Plane (180deg, Fast)

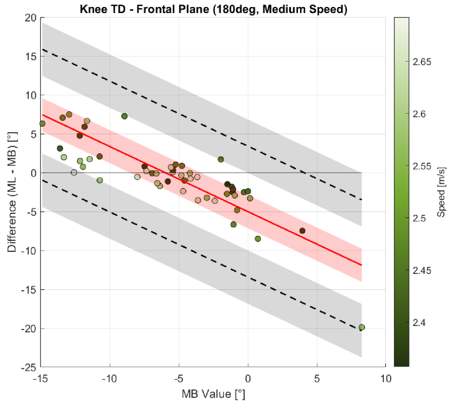

Supplementary Figure 596: Extended BA Plot for TD knee angle - Frontal Plane (180deg, Medium)

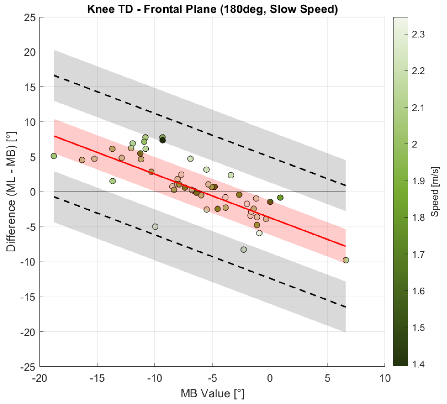

Supplementary Figure 597: Extended BA Plot for TD knee angle - Frontal Plane (180deg, Slow)

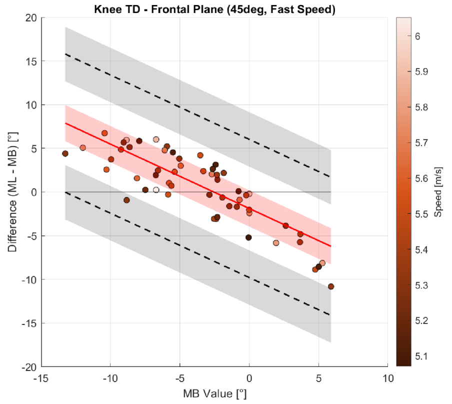

Supplementary Figure 598: Extended BA Plot for TD knee angle - Frontal Plane (45deg, Fast)

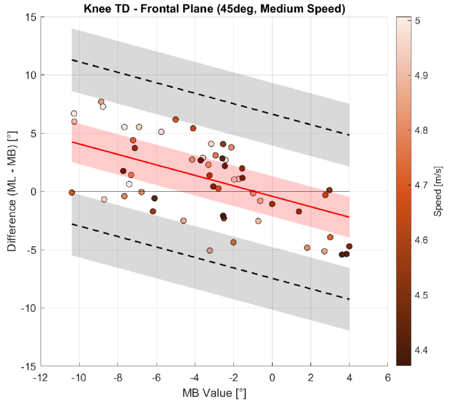

Supplementary Figure 599: Extended BA Plot for TD knee angle - Frontal Plane (45deg, Medium)

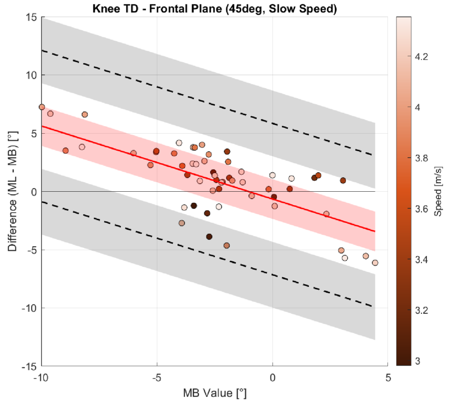

Supplementary Figure 600: Extended BA Plot for TD knee angle - Frontal Plane (45deg, Slow)

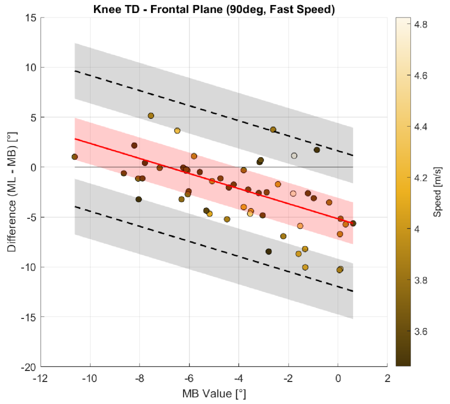

Supplementary Figure 601: Extended BA Plot for TD knee angle - Frontal Plane (90deg, Fast)

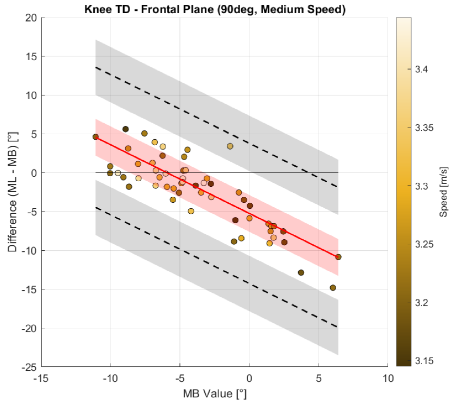

Supplementary Figure 602: Extended BA Plot for TD knee angle - Frontal Plane (90deg, Medium)

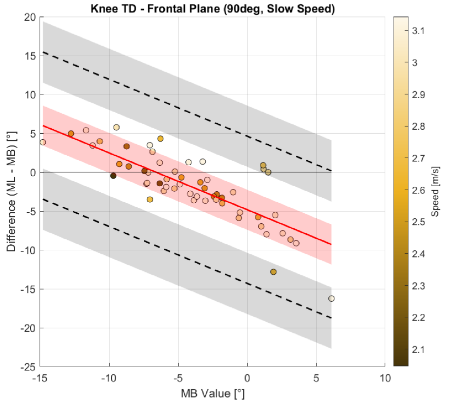

Supplementary Figure 603: Extended BA Plot for TD knee angle - Frontal Plane (90deg, Slow)

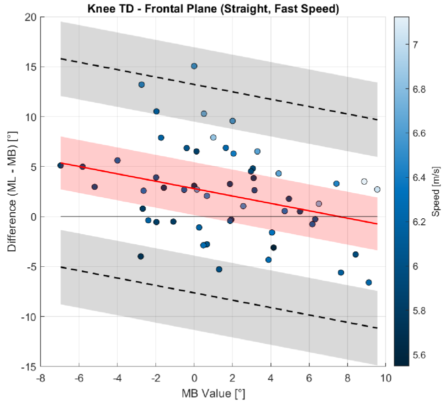

Supplementary Figure 604: Extended BA Plot for TD knee angle - Frontal Plane (Straight, Fast)

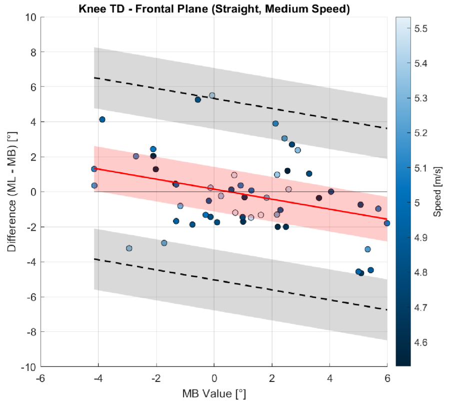

Supplementary Figure 605: Extended BA Plot for TD knee angle - Frontal Plane (Straight, Medium)

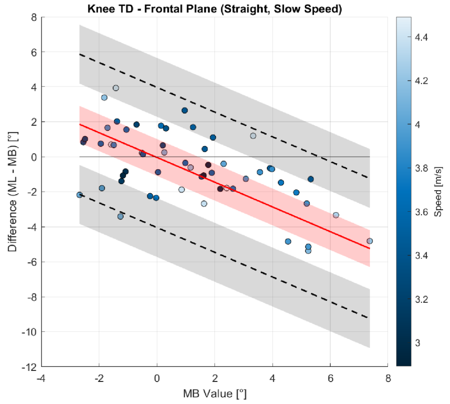

Supplementary Figure 606: Extended BA Plot for TD knee angle - Frontal Plane (Straight, Slow)

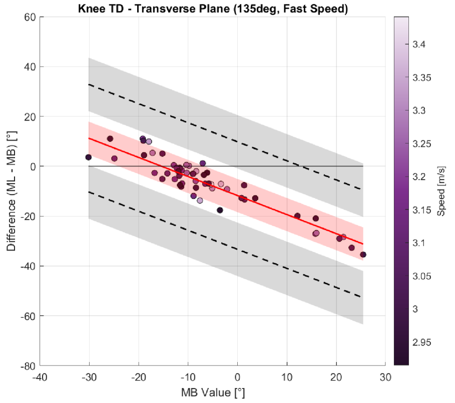

Supplementary Figure 607: Extended BA Plot for TD knee angle - Transverse Plane (135deg, Fast)

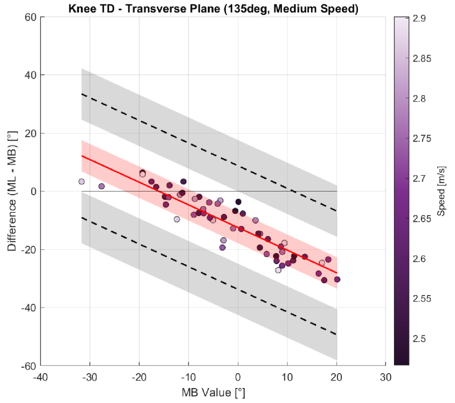

Supplementary Figure 608: Extended BA Plot for TD knee angle - Transverse Plane (135deg, Medium)

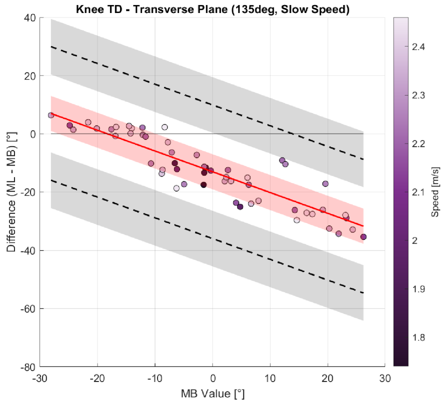

Supplementary Figure 609: Extended BA Plot for TD knee angle - Transverse Plane (135deg, Slow)

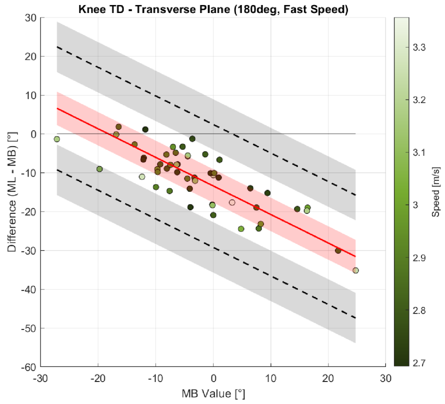

Supplementary Figure 610: Extended BA Plot for TD knee angle - Transverse Plane (180deg, Fast)

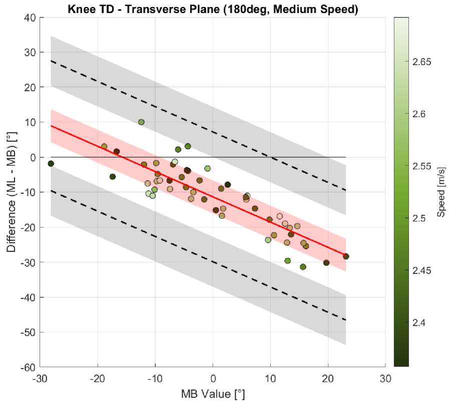

Supplementary Figure 611: Extended BA Plot for TD knee angle - Transverse Plane (180deg, Medium)

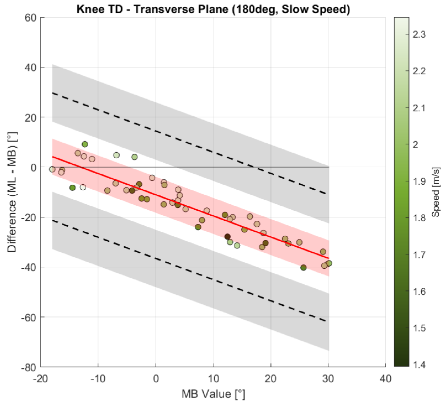

Supplementary Figure 612: Extended BA Plot for TD knee angle - Transverse Plane (180deg, Slow)

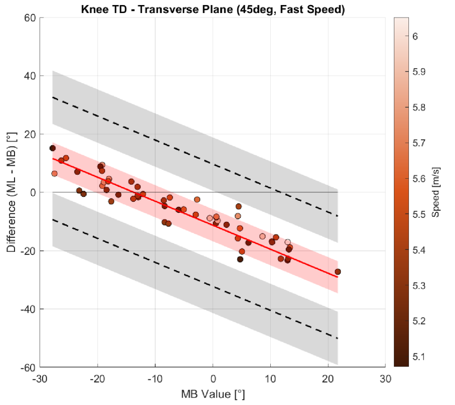

Supplementary Figure 613: Extended BA Plot for TD knee angle - Transverse Plane (45deg, Fast)

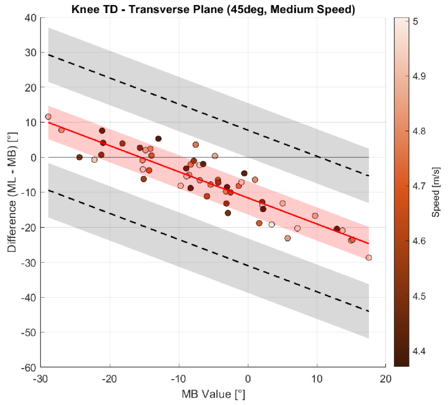

Supplementary Figure 614: Extended BA Plot for TD knee angle - Transverse Plane (45deg, Medium)

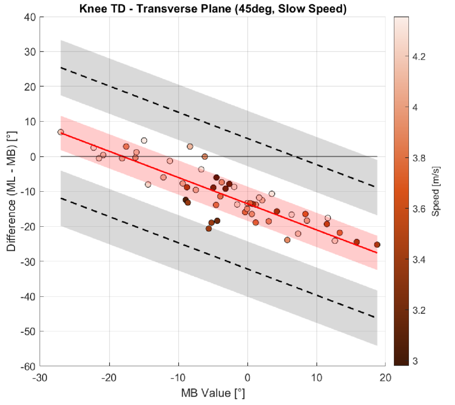

Supplementary Figure 615: Extended BA Plot for TD knee angle - Transverse Plane (45deg, Slow)

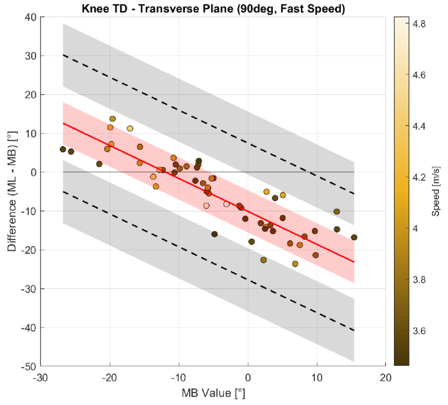

Supplementary Figure 616: Extended BA Plot for TD knee angle - Transverse Plane (90deg, Fast)

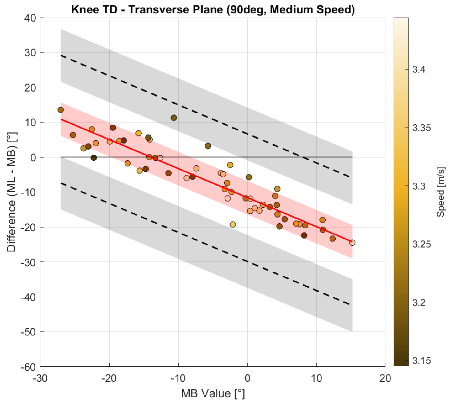

Supplementary Figure 617: Extended BA Plot for TD knee angle - Transverse Plane (90deg, Medium)

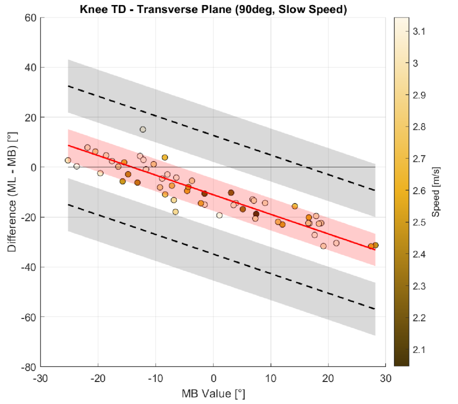

Supplementary Figure 618: Extended BA Plot for TD knee angle - Transverse Plane (90deg, Slow)

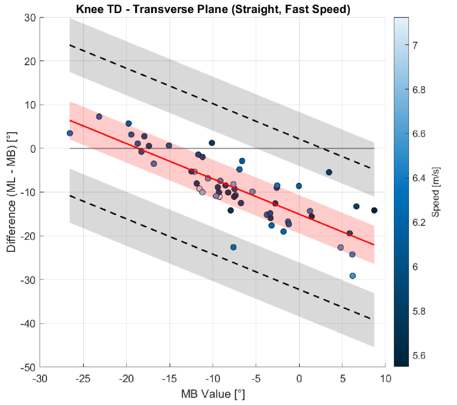

Supplementary Figure 619: Extended BA Plot for TD knee angle - Transverse Plane (Straight, Fast)

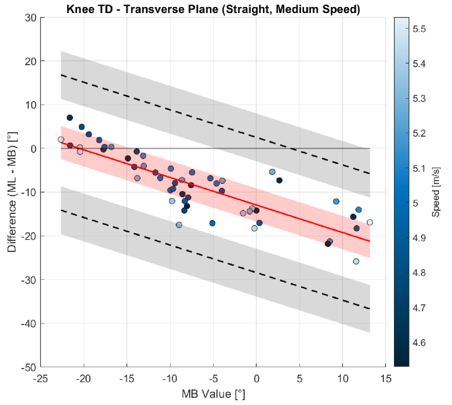

Supplementary Figure 620: Extended BA Plot for TD knee angle - Transverse Plane (Straight, Medium)

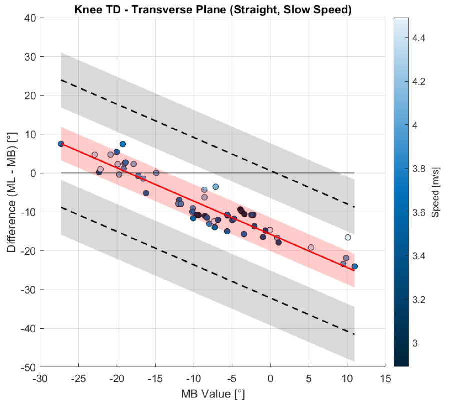

Supplementary Figure 621: Extended BA Plot for TD knee angle - Transverse Plane (Straight, Slow)

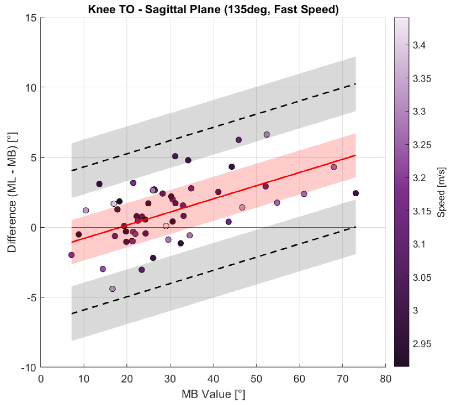

Supplementary Figure 622: Extended BA Plot for TO knee angle - Sagittal Plane (135deg, Fast)

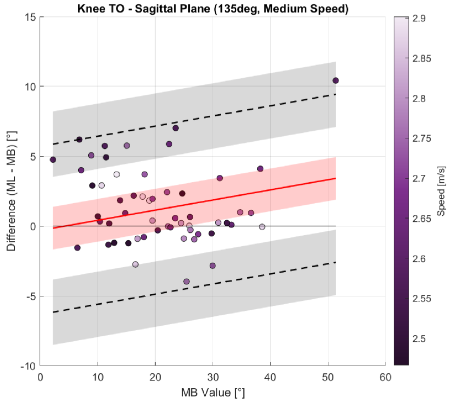

Supplementary Figure 623: Extended BA Plot for TO knee angle - Sagittal Plane (135deg, Medium)

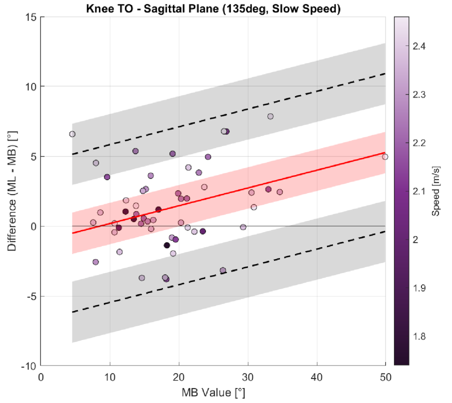

Supplementary Figure 624: Extended BA Plot for TO knee angle - Sagittal Plane (135deg, Slow)

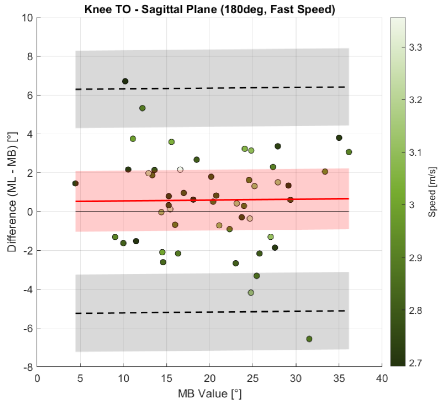

Supplementary Figure 625: Extended BA Plot for TO knee angle - Sagittal Plane (180deg, Fast)

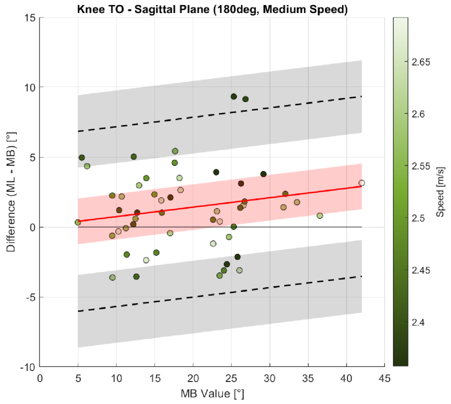

Supplementary Figure 626: Extended BA Plot for TO knee angle - Sagittal Plane (180deg, Medium)

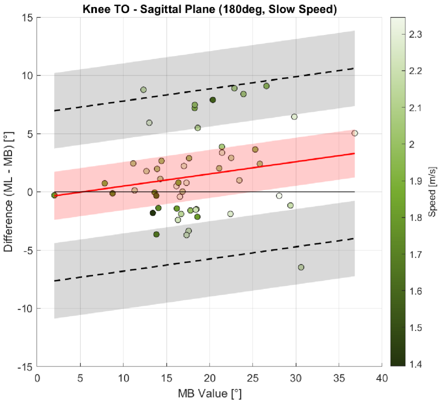

Supplementary Figure 627: Extended BA Plot for TO knee angle - Sagittal Plane (180deg, Slow)

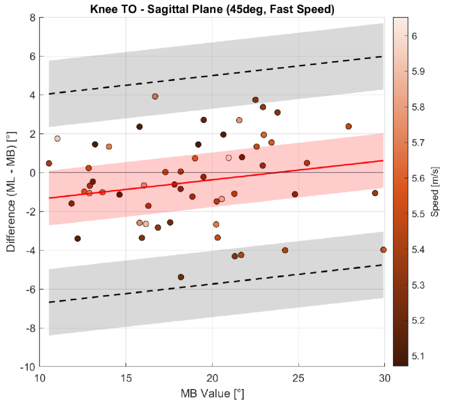

Supplementary Figure 628: Extended BA Plot for TO knee angle - Sagittal Plane (45deg, Fast)

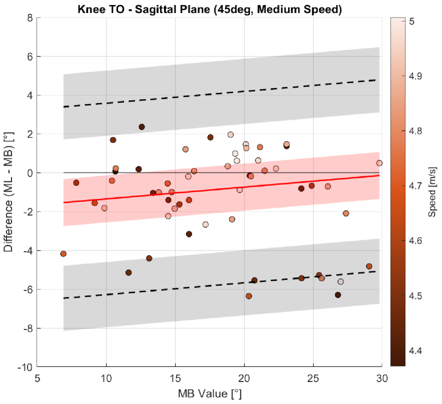

Supplementary Figure 629: Extended BA Plot for TO knee angle - Sagittal Plane (45deg, Medium)

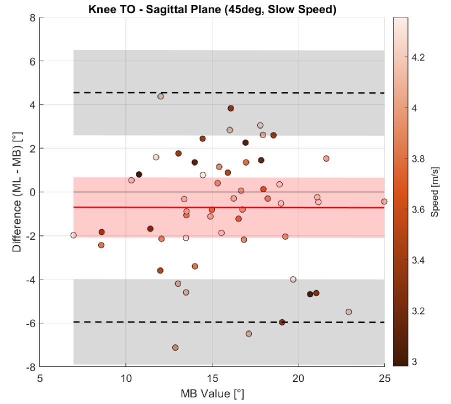

Supplementary Figure 630: Extended BA Plot for TO knee angle - Sagittal Plane (45deg, Slow)

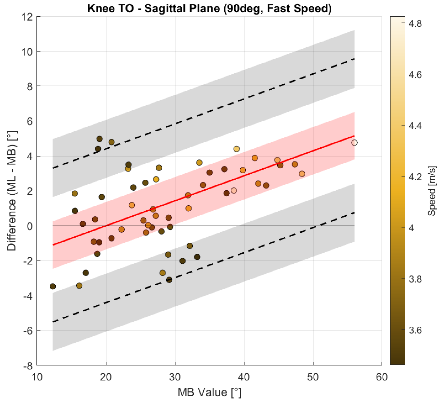

Supplementary Figure 631: Extended BA Plot for TO knee angle - Sagittal Plane (90deg, Fast)

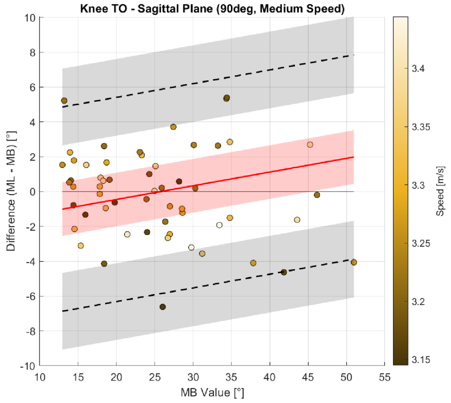

Supplementary Figure 632: Extended BA Plot for TO knee angle - Sagittal Plane (90deg, Medium)

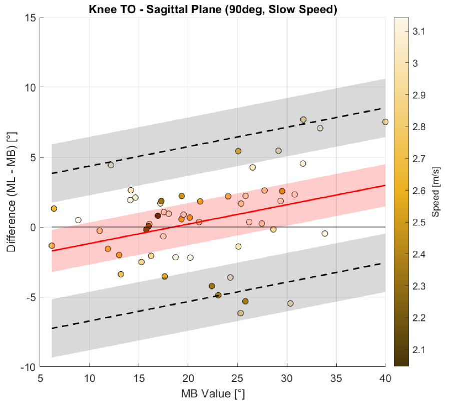

Supplementary Figure 633: Extended BA Plot for TO knee angle - Sagittal Plane (90deg, Slow)

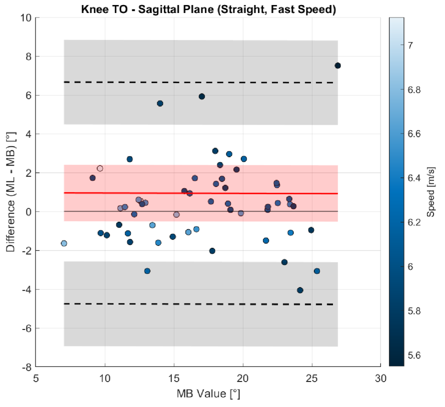

Supplementary Figure 634: Extended BA Plot for TO knee angle - Sagittal Plane (Straight, Fast)

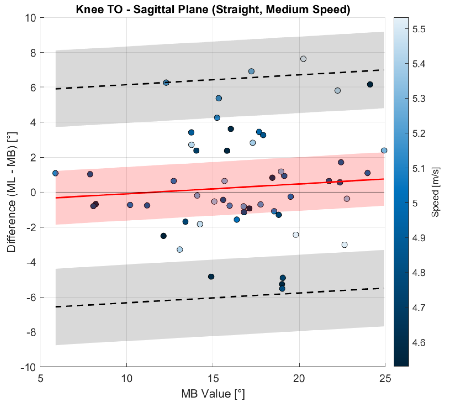

Supplementary Figure 635: Extended BA Plot for TO knee angle - Sagittal Plane (Straight, Medium)

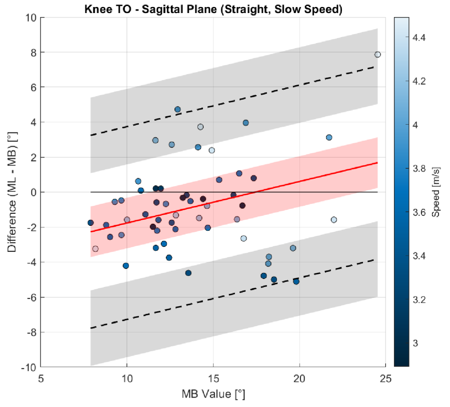

Supplementary Figure 636: Extended BA Plot for TO knee angle - Sagittal Plane (Straight, Slow)

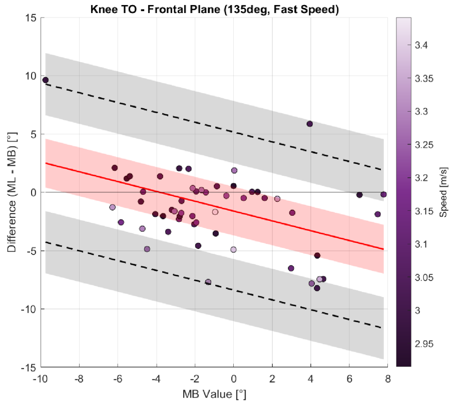

Supplementary Figure 637: Extended BA Plot for TO knee angle - Frontal Plane (135deg, Fast)

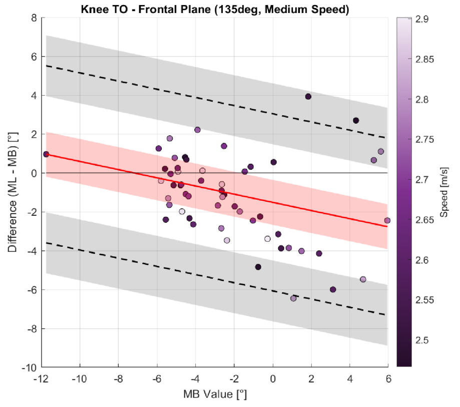

Supplementary Figure 638: Extended BA Plot for TO knee angle - Frontal Plane (135deg, Medium)

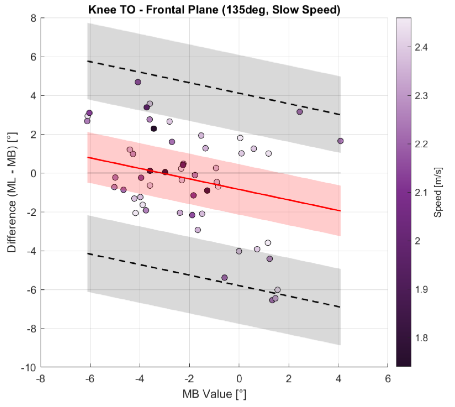

Supplementary Figure 639: Extended BA Plot for TO knee angle - Frontal Plane (135deg, Slow)

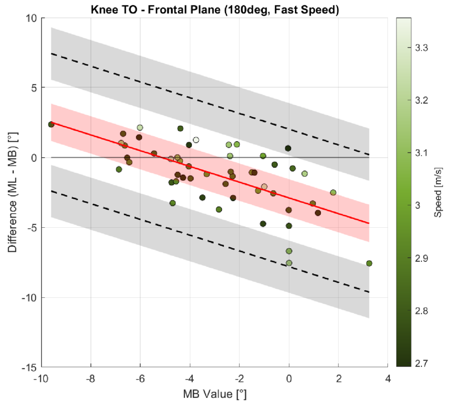

Supplementary Figure 640: Extended BA Plot for TO knee angle - Frontal Plane (180deg, Fast)

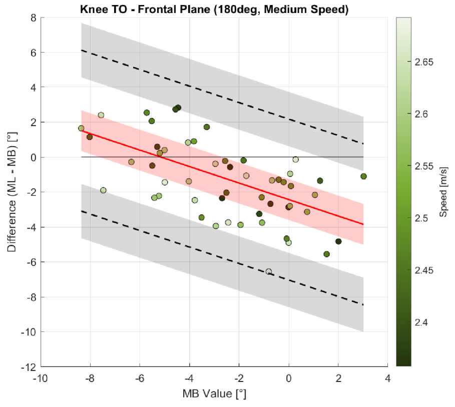

Supplementary Figure 641: Extended BA Plot for TO knee angle - Frontal Plane (180deg, Medium)

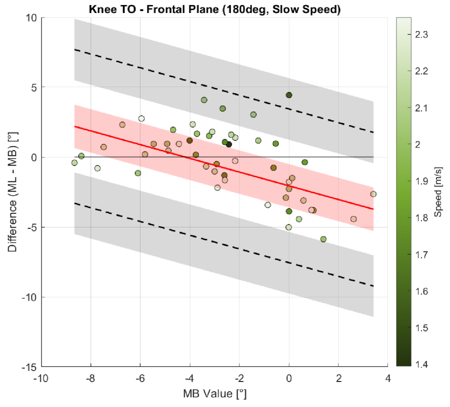

Supplementary Figure 642: Extended BA Plot for TO knee angle - Frontal Plane (180deg, Slow)

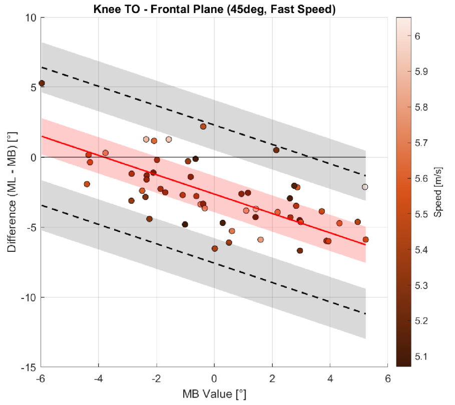

Supplementary Figure 643: Extended BA Plot for TO knee angle - Frontal Plane (45deg, Fast)

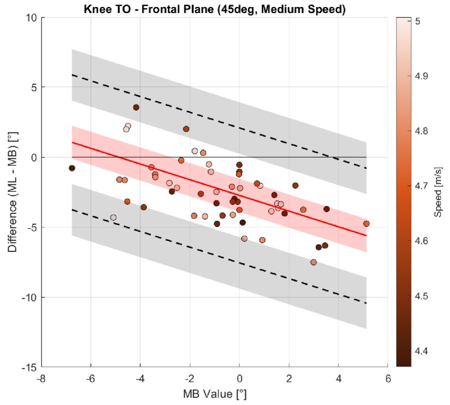

Supplementary Figure 644: Extended BA Plot for TO knee angle - Frontal Plane (45deg, Medium)

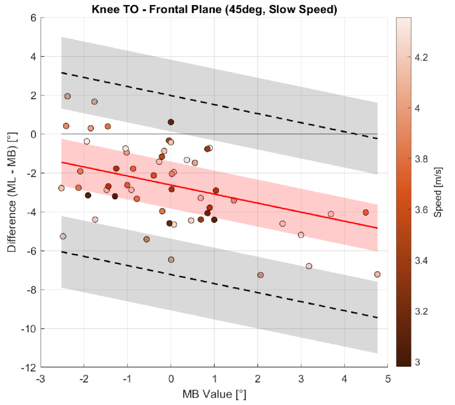

Supplementary Figure 645: Extended BA Plot for TO knee angle - Frontal Plane (45deg, Slow)

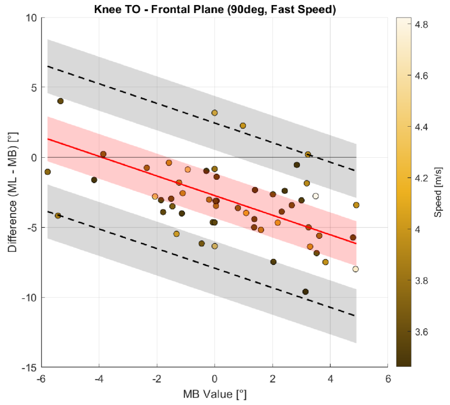

Supplementary Figure 646: Extended BA Plot for TO knee angle - Frontal Plane (90deg, Fast)

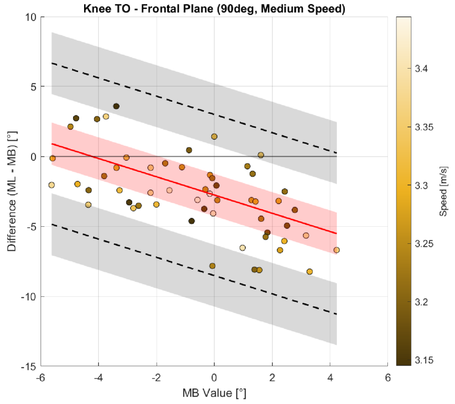

Supplementary Figure 647: Extended BA Plot for TO knee angle - Frontal Plane (90deg, Medium)

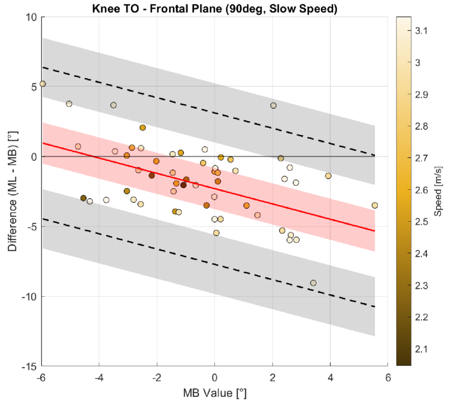

Supplementary Figure 648: Extended BA Plot for TO knee angle - Frontal Plane (90deg, Slow)

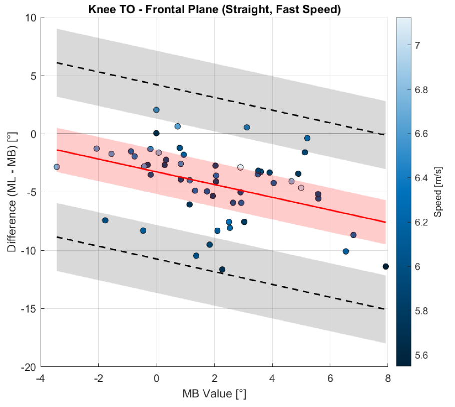

Supplementary Figure 649: Extended BA Plot for TO knee angle - Frontal Plane (Straight, Fast)

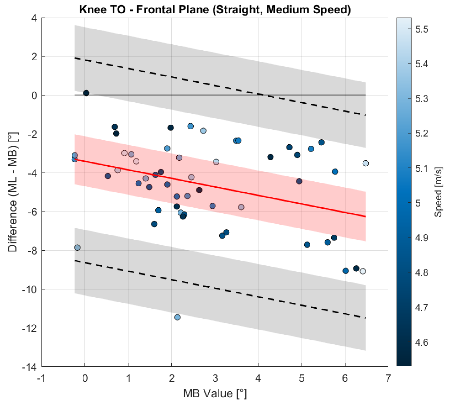

Supplementary Figure 650: Extended BA Plot for TO knee angle - Frontal Plane (Straight, Medium)

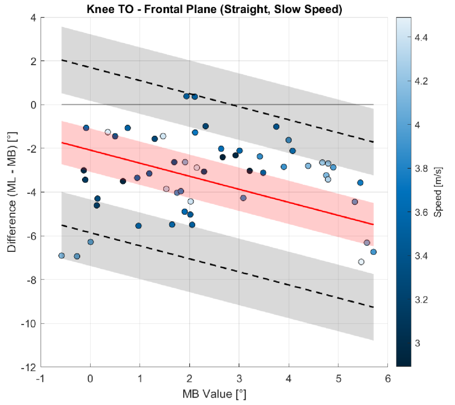

Supplementary Figure 651: Extended BA Plot for TO knee angle - Frontal Plane (Straight, Slow)

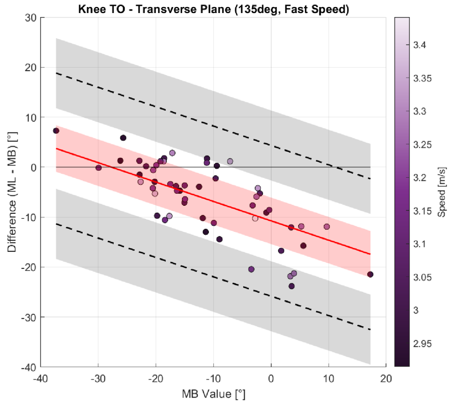

Supplementary Figure 652: Extended BA Plot for TO knee angle - Transverse Plane (135deg, Fast)

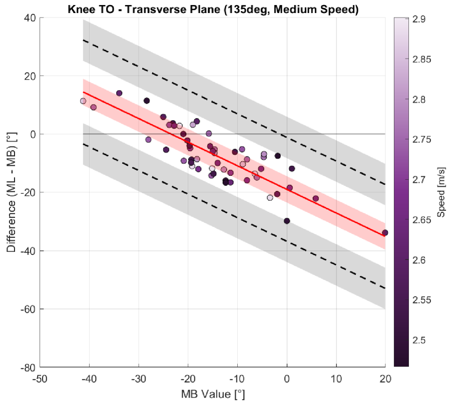

Supplementary Figure 653: Extended BA Plot for TO knee angle - Transverse Plane (135deg, Medium)

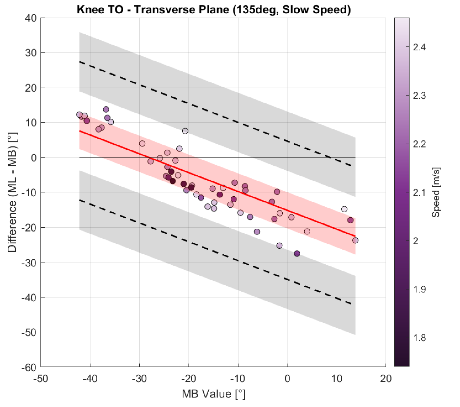

Supplementary Figure 654: Extended BA Plot for TO knee angle - Transverse Plane (135deg, Slow)

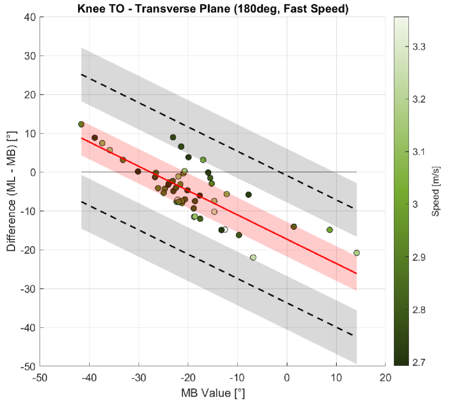

Supplementary Figure 655: Extended BA Plot for TO knee angle - Transverse Plane (180deg, Fast)

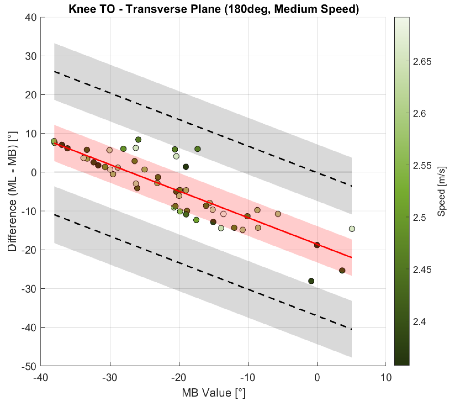

Supplementary Figure 656: Extended BA Plot for TO knee angle - Transverse Plane (180deg, Medium)

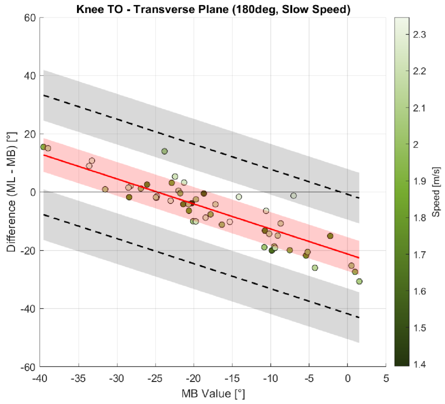

Supplementary Figure 657: Extended BA Plot for TO knee angle - Transverse Plane (180deg, Slow)

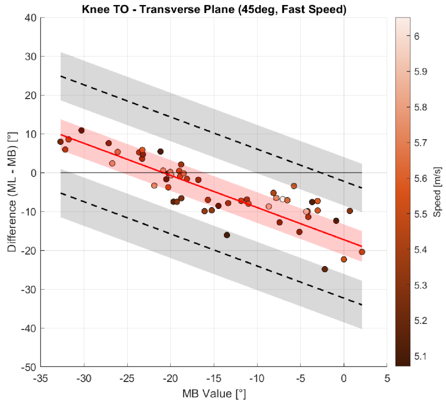

Supplementary Figure 658: Extended BA Plot for TO knee angle - Transverse Plane (45deg, Fast)

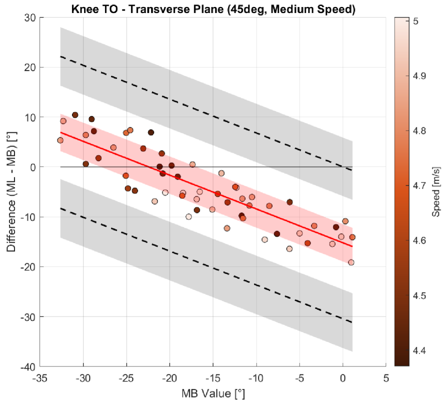

Supplementary Figure 659: Extended BA Plot for TO knee angle - Transverse Plane (45deg, Medium)

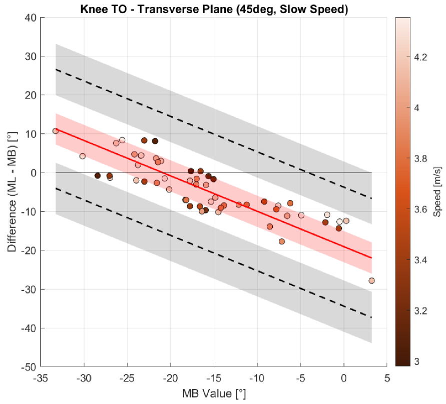

Supplementary Figure 660: Extended BA Plot for TO knee angle - Transverse Plane (45deg, Slow)

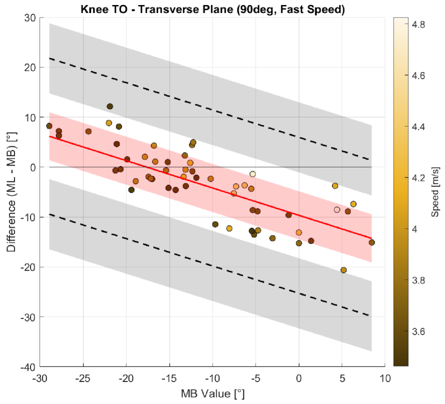

Supplementary Figure 661: Extended BA Plot for TO knee angle - Transverse Plane (90deg, Fast)

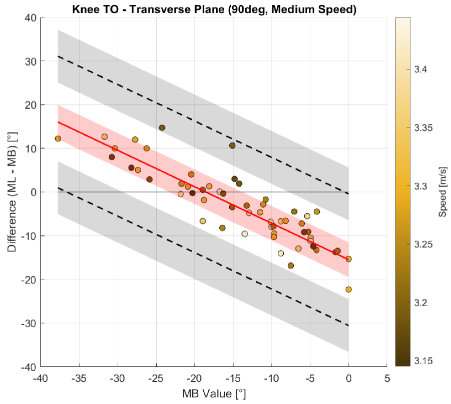

Supplementary Figure 662: Extended BA Plot for TO knee angle - Transverse Plane (90deg, Medium)

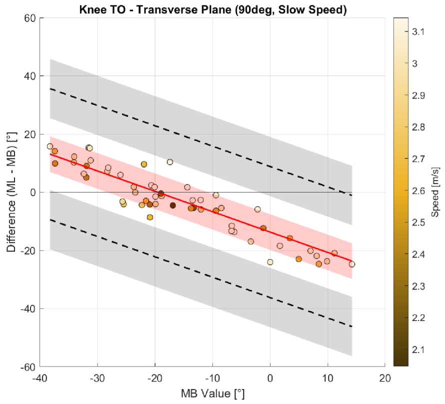

Supplementary Figure 663: Extended BA Plot for TO knee angle - Transverse Plane (90deg, Slow)

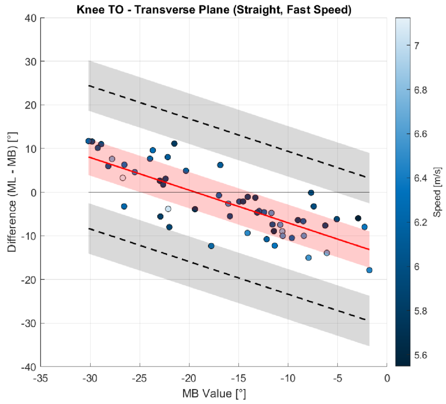

Supplementary Figure 664: Extended BA Plot for TO knee angle - Transverse Plane (Straight, Fast)

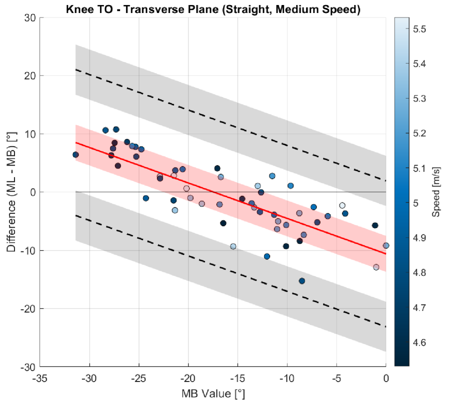

Supplementary Figure 665: Extended BA Plot for TO knee angle - Transverse Plane (Straight, Medium)

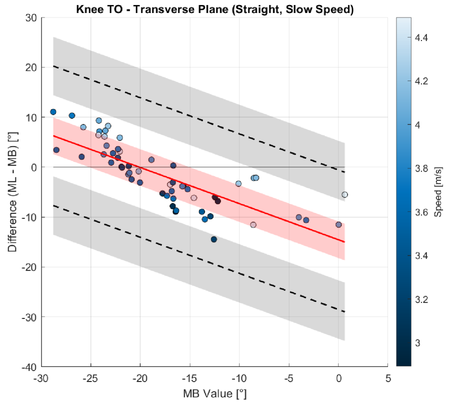

Supplementary Figure 666: Extended BA Plot for TO knee angle - Transverse Plane (Straight, Slow)

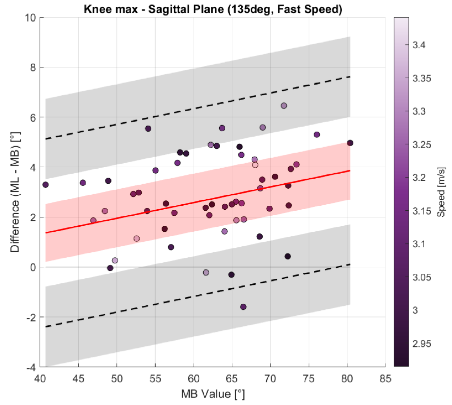

Supplementary Figure 667: Extended BA Plot for maximal knee angle - Sagittal Plane (135deg, Fast)

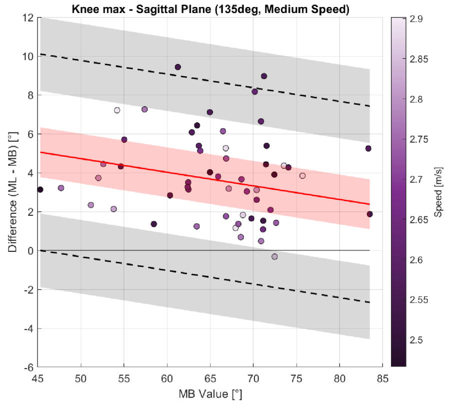

Supplementary Figure 668: Extended BA Plot for maximal knee angle - Sagittal Plane (135deg, Medium)

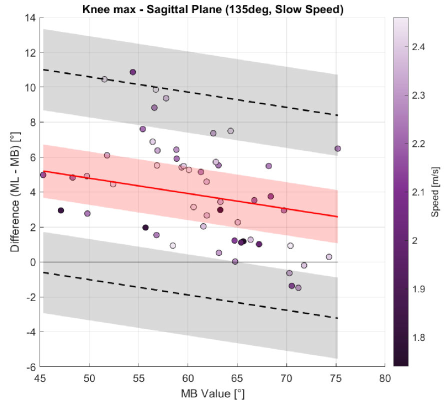

Supplementary Figure 669: Extended BA Plot for maximal knee angle - Sagittal Plane (135deg, Slow)

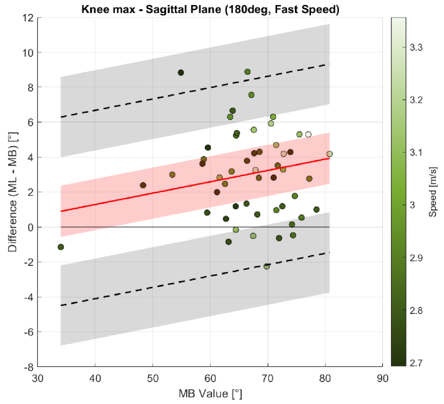

Supplementary Figure 670: Extended BA Plot for maximal knee angle - Sagittal Plane (180deg, Fast)

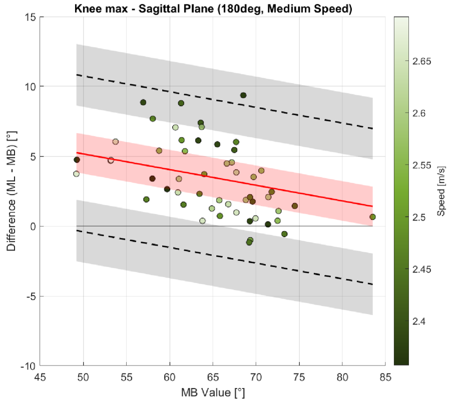

Supplementary Figure 671: Extended BA Plot for maximal knee angle - Sagittal Plane (180deg, Medium)

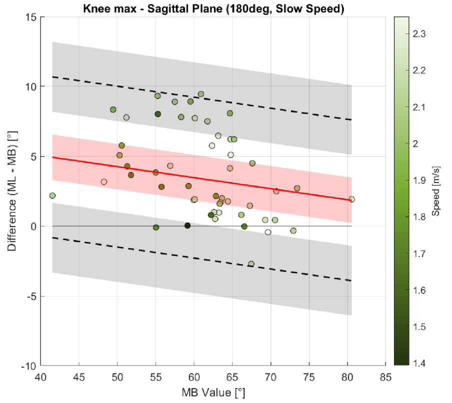

Supplementary Figure 672: Extended BA Plot for maximal knee angle - Sagittal Plane (180deg, Slow)

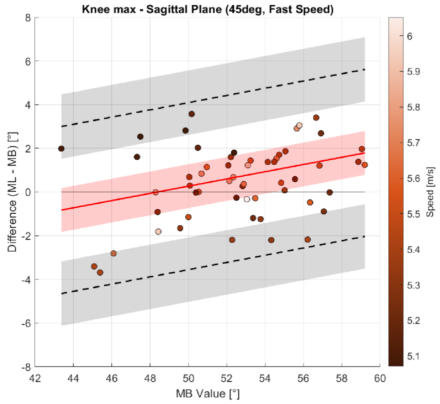

Supplementary Figure 673: Extended BA Plot for maximal knee angle - Sagittal Plane (45deg, Fast)

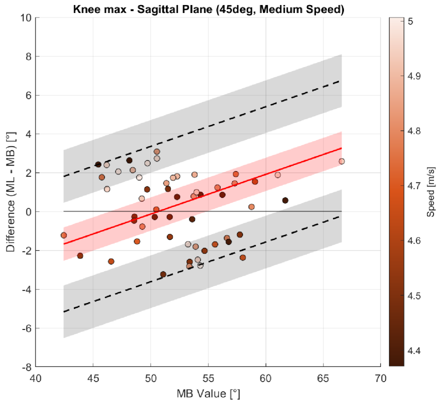

Supplementary Figure 674: Extended BA Plot for maximal knee angle - Sagittal Plane (45deg, Medium)

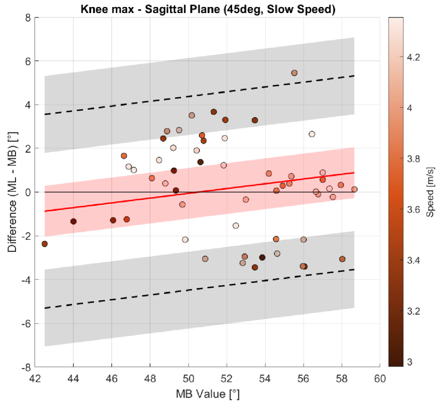

Supplementary Figure 675: Extended BA Plot for maximal knee angle - Sagittal Plane (45deg, Slow)

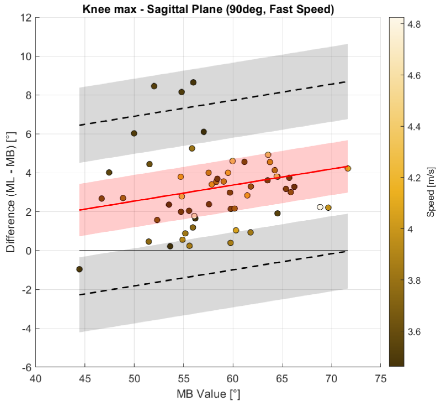

Supplementary Figure 676: Extended BA Plot for maximal knee angle - Sagittal Plane (90deg, Fast)

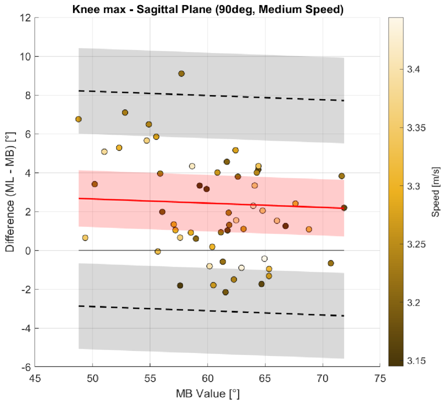

Supplementary Figure 677: Extended BA Plot for maximal knee angle - Sagittal Plane (90deg, Medium)

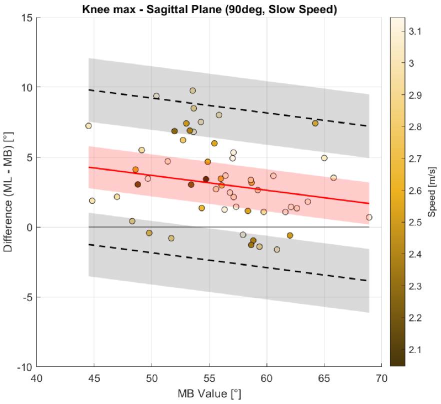

Supplementary Figure 678: Extended BA Plot for maximal knee angle - Sagittal Plane (90deg, Slow)

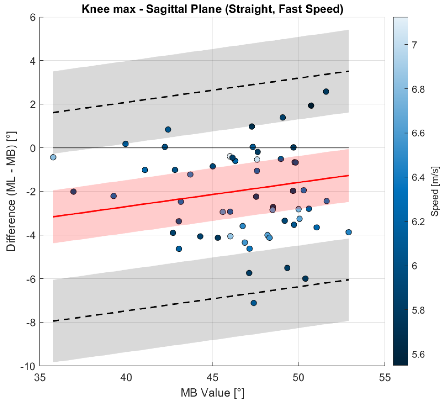

Supplementary Figure 679: Extended BA Plot for maximal knee angle - Sagittal Plane (Straight, Fast)

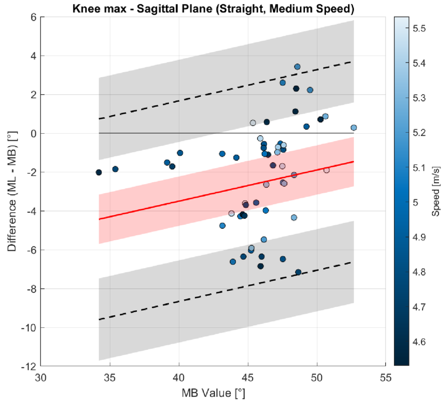

Supplementary Figure 680: Extended BA Plot for maximal knee angle - Sagittal Plane (Straight, Medium)

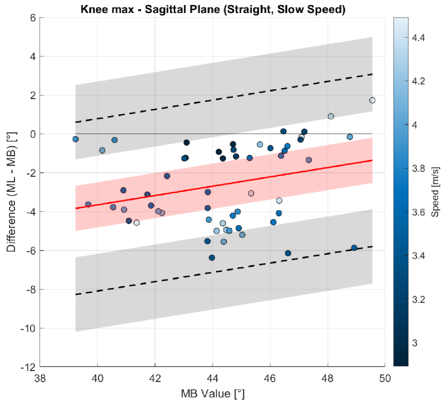

Supplementary Figure 681: Extended BA Plot for maximal knee angle - Sagittal Plane (Straight, Slow)

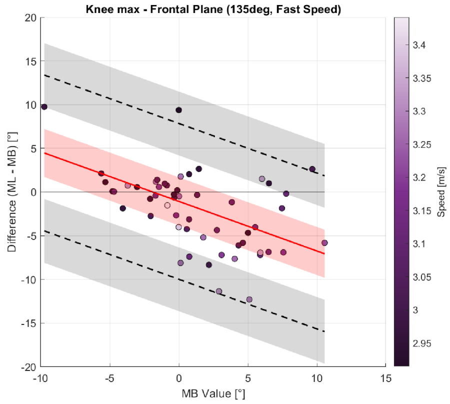

Supplementary Figure 682: Extended BA Plot for maximal knee angle - Frontal Plane (135deg, Fast)

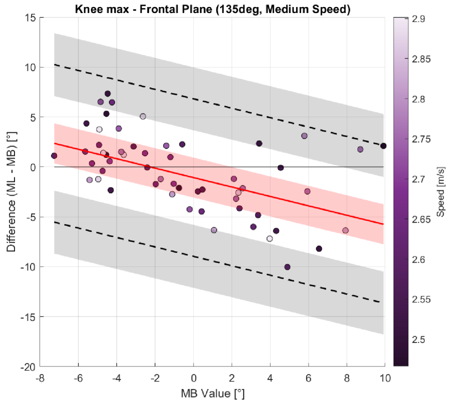

Supplementary Figure 683: Extended BA Plot for maximal knee angle - Frontal Plane (135deg, Medium)

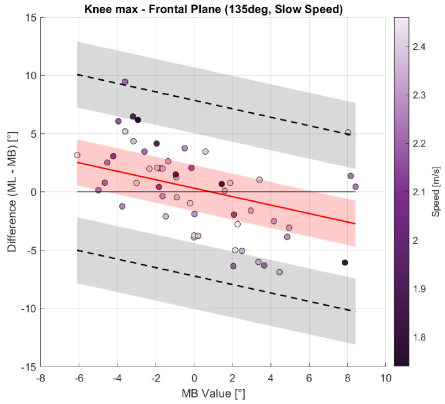

Supplementary Figure 684: Extended BA Plot for maximal knee angle - Frontal Plane (135deg, Slow)

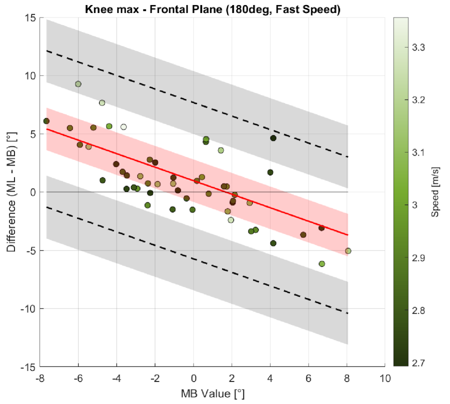

Supplementary Figure 685: Extended BA Plot for maximal knee angle - Frontal Plane (180deg, Fast)

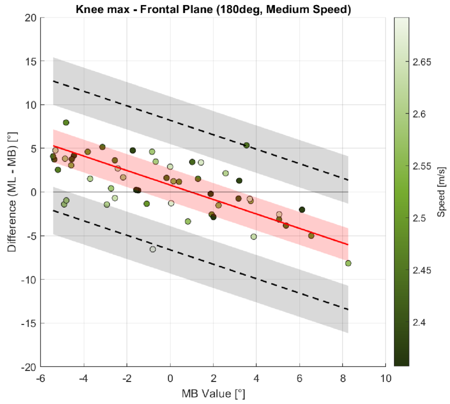

Supplementary Figure 686: Extended BA Plot for maximal knee angle - Frontal Plane (180deg, Medium)

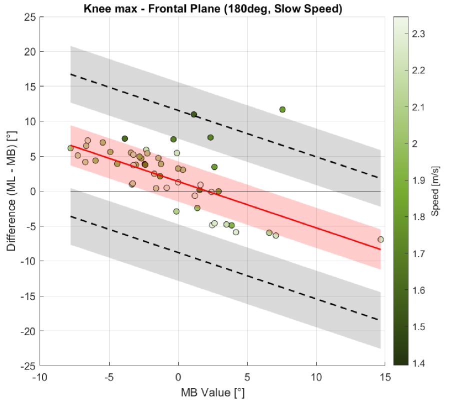

Supplementary Figure 687: Extended BA Plot for maximal knee angle - Frontal Plane (180deg, Slow)

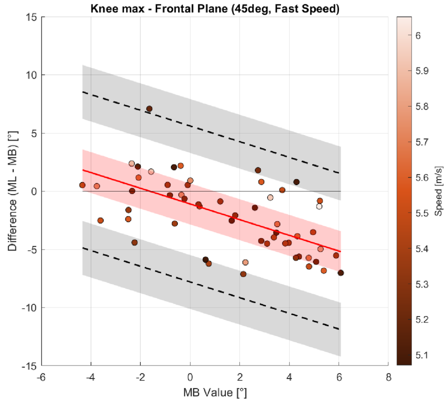

Supplementary Figure 688: Extended BA Plot for maximal knee angle - Frontal Plane (45deg, Fast)

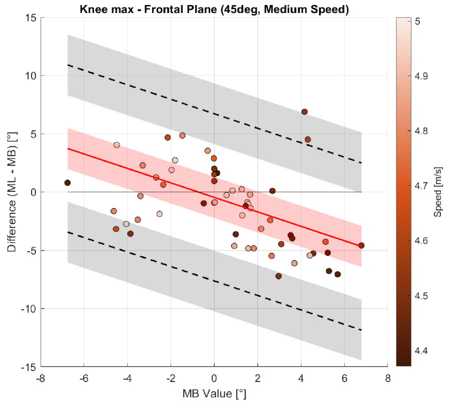

Supplementary Figure 689: Extended BA Plot for maximal knee angle - Frontal Plane (45deg, Medium)

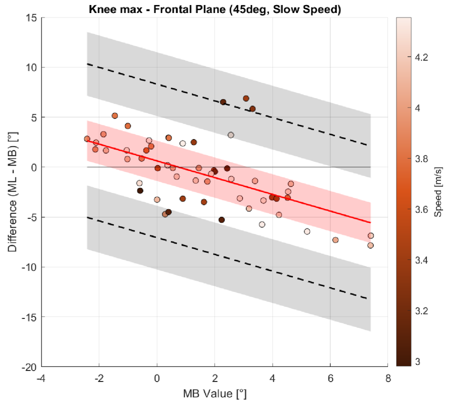

Supplementary Figure 690: Extended BA Plot for maximal knee angle - Frontal Plane (45deg, Slow)

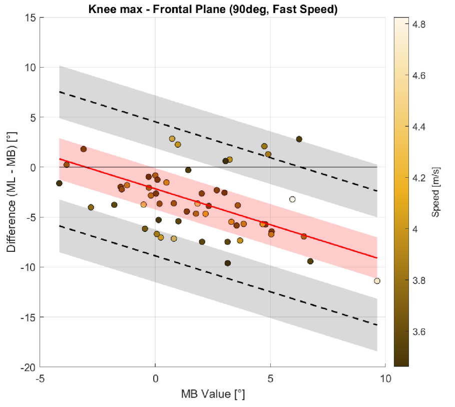

Supplementary Figure 691: Extended BA Plot for maximal knee angle - Frontal Plane (90deg, Fast)

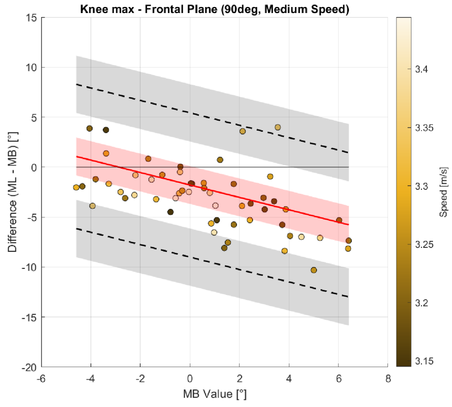

Supplementary Figure 692: Extended BA Plot for maximal knee angle - Frontal Plane (90deg, Medium)

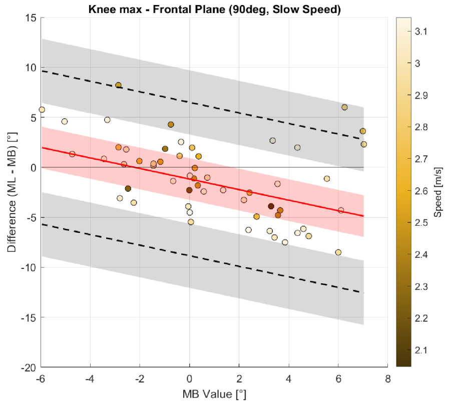

Supplementary Figure 693: Extended BA Plot for maximal knee angle - Frontal Plane (90deg, Slow)

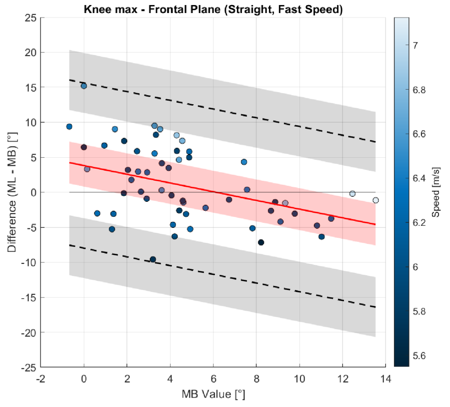

Supplementary Figure 694: Extended BA Plot for maximal knee angle - Frontal Plane (Straight, Fast)

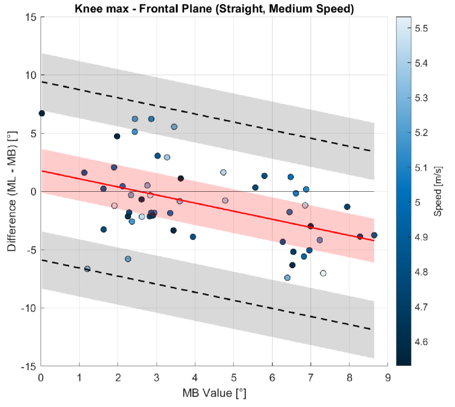

Supplementary Figure 695: Extended BA Plot for maximal knee angle - Frontal Plane (Straight, Medium)

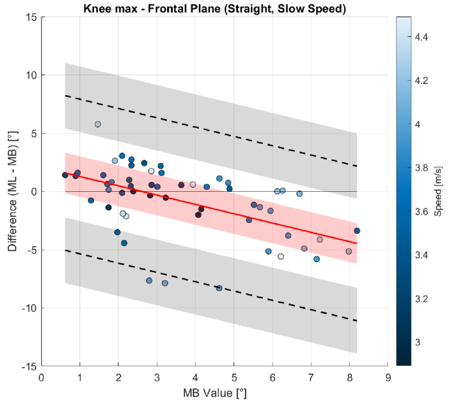

Supplementary Figure 696: Extended BA Plot for maximal knee angle - Frontal Plane (Straight, Slow)

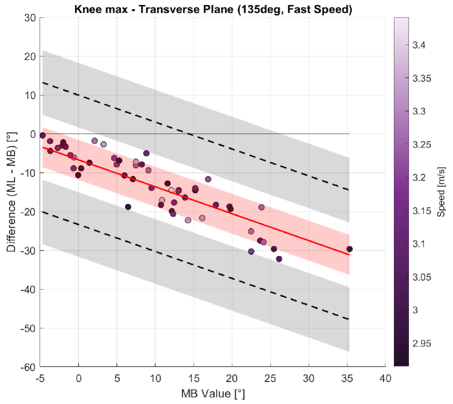

Supplementary Figure 697: Extended BA Plot for maximal knee angle - Transverse Plane (135deg, Fast)

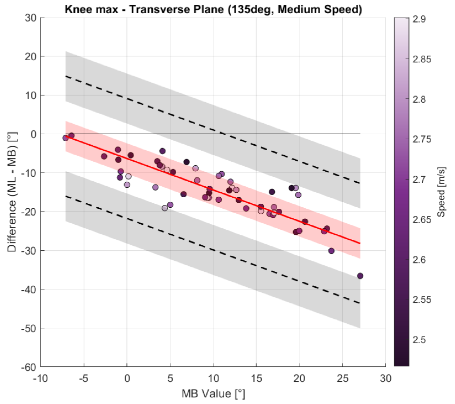

Supplementary Figure 698: Extended BA Plot for maximal knee angle - Transverse Plane (135deg, Medium)

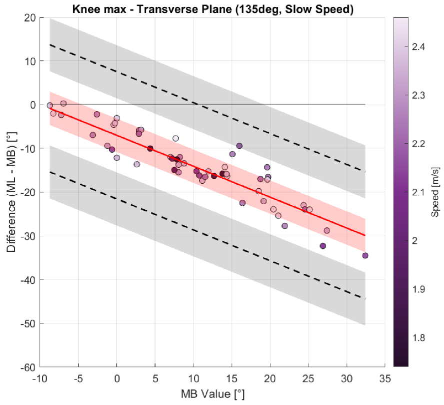

Supplementary Figure 699: Extended BA Plot for maximal knee angle - Transverse Plane (135deg, Slow)

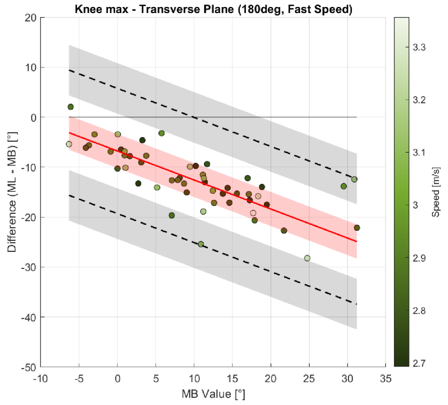

Supplementary Figure 700: Extended BA Plot for maximal knee angle - Transverse Plane (180deg, Fast)

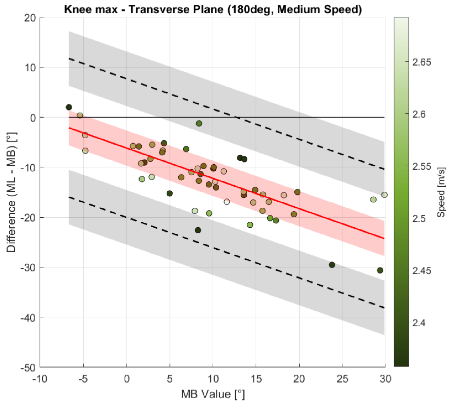

Supplementary Figure 701: Extended BA Plot for maximal knee angle - Transverse Plane (180deg, Medium)

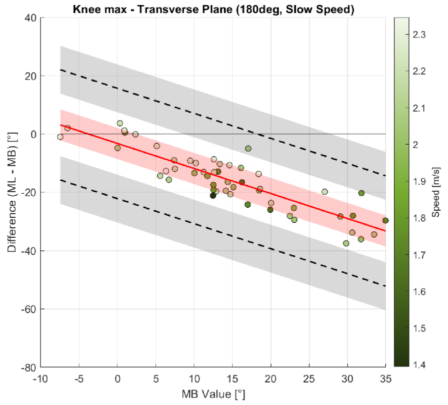

Supplementary Figure 702: Extended BA Plot for maximal knee angle - Transverse Plane (180deg, Slow)

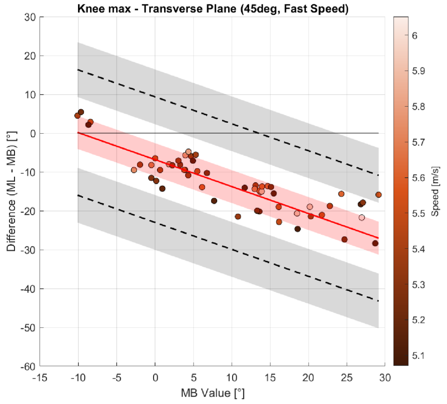

Supplementary Figure 703: Extended BA Plot for maximal knee angle - Transverse Plane (45deg, Fast)

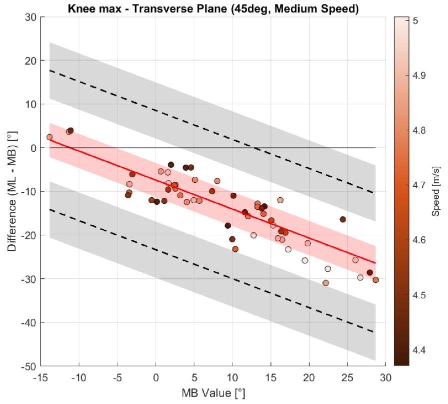

Supplementary Figure 704: Extended BA Plot for maximal knee angle - Transverse Plane (45deg, Medium)

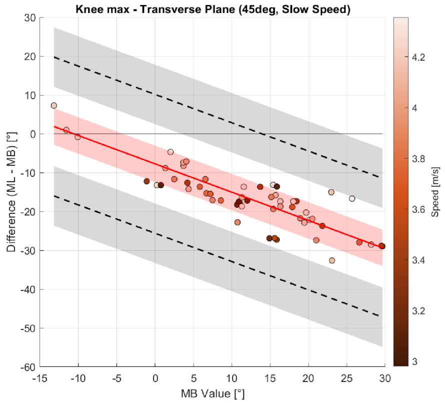

Supplementary Figure 705: Extended BA Plot for maximal knee angle - Transverse Plane (45deg, Slow)

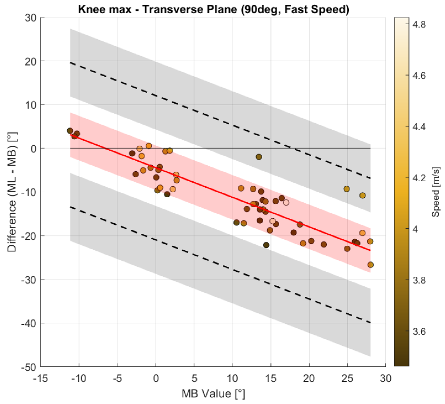

Supplementary Figure 706: Extended BA Plot for maximal knee angle - Transverse Plane (90deg, Fast)

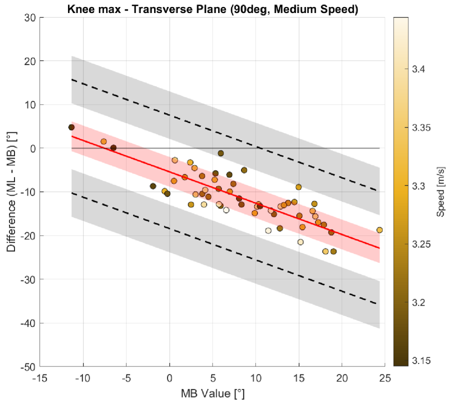

Supplementary Figure 707: Extended BA Plot for maximal knee angle - Transverse Plane (90deg, Medium)

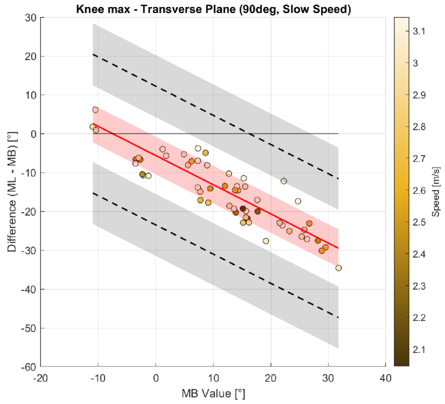

Supplementary Figure 708: Extended BA Plot for maximal knee angle - Transverse Plane (90deg, Slow)

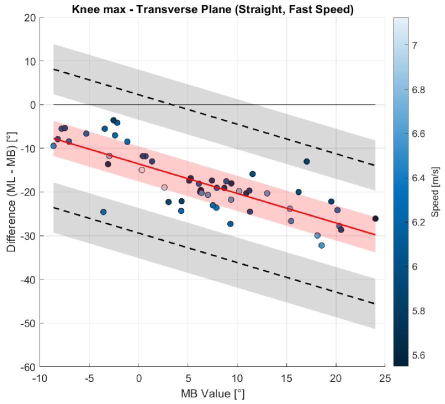

Supplementary Figure 709: Extended BA Plot for maximal knee angle - Transverse Plane (Straight, Fast)

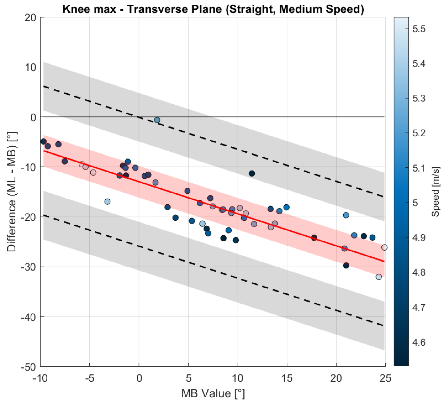

Supplementary Figure 710: Extended BA Plot for maximal knee angle - Transverse Plane (Straight, Medium)

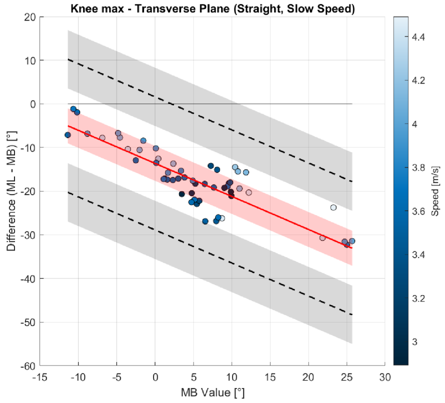

Supplementary Figure 711: Extended BA Plot for maximal knee angle - Transverse Plane (Straight, Slow)

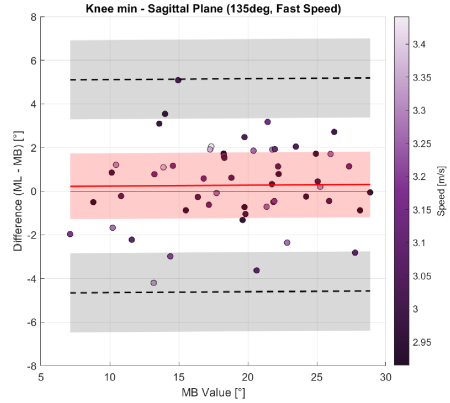

Supplementary Figure 712: Extended BA Plot for minimal knee angle - Sagittal Plane (135deg, Fast)

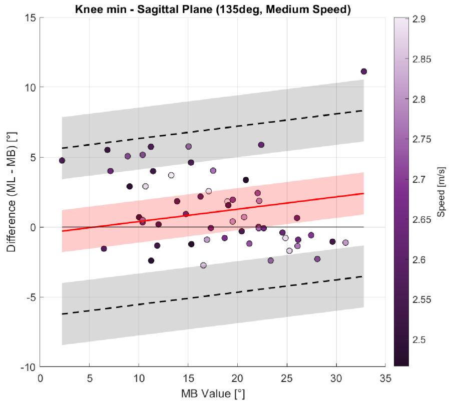

Supplementary Figure 713: Extended BA Plot for minimal knee angle - Sagittal Plane (135deg, Medium)

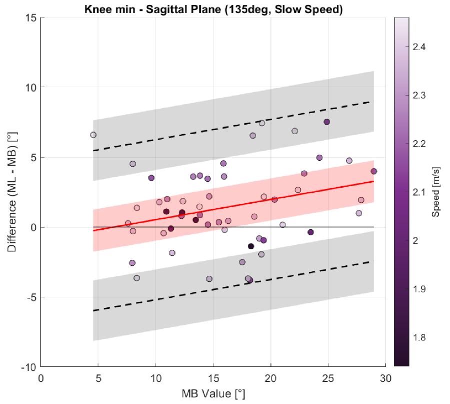

Supplementary Figure 714: Extended BA Plot for minimal knee angle - Sagittal Plane (135deg, Slow)

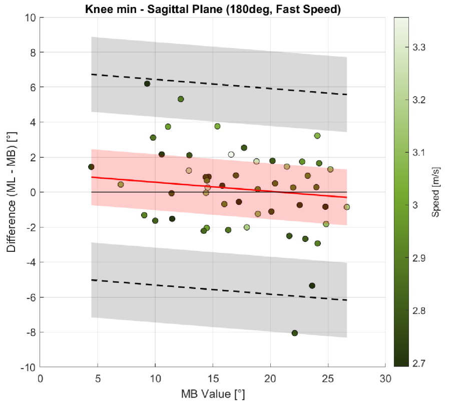

Supplementary Figure 715: Extended BA Plot for minimal knee angle - Sagittal Plane (180deg, Fast)

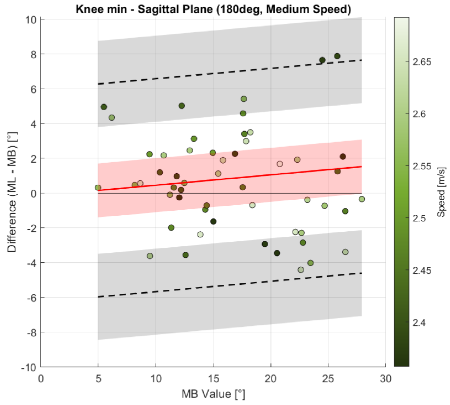

Supplementary Figure 716: Extended BA Plot for minimal knee angle - Sagittal Plane (180deg, Medium)

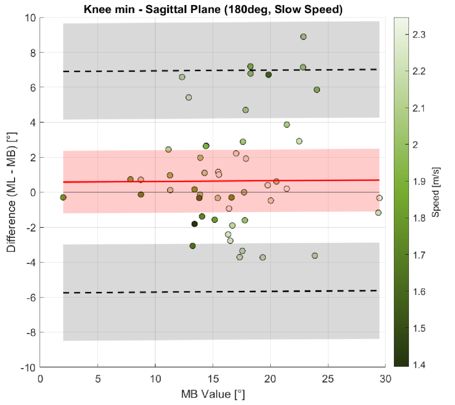

Supplementary Figure 717: Extended BA Plot for minimal knee angle - Sagittal Plane (180deg, Slow)

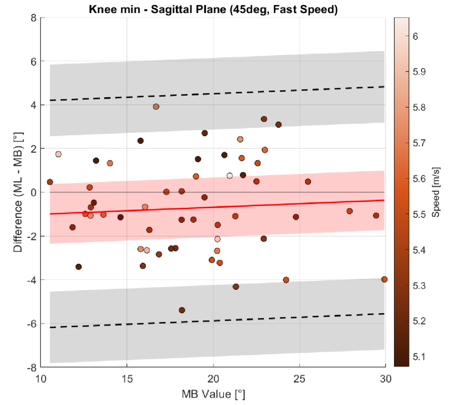

Supplementary Figure 718: Extended BA Plot for minimal knee angle - Sagittal Plane (45deg, Fast)

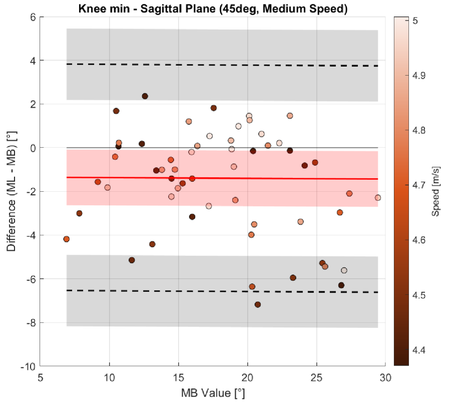

Supplementary Figure 719: Extended BA Plot for minimal knee angle - Sagittal Plane (45deg, Medium)

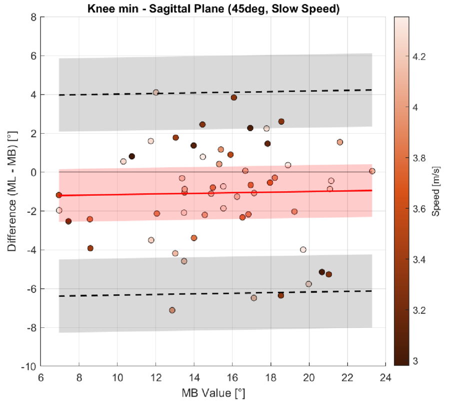

Supplementary Figure 720: Extended BA Plot for minimal knee angle - Sagittal Plane (45deg, Slow)

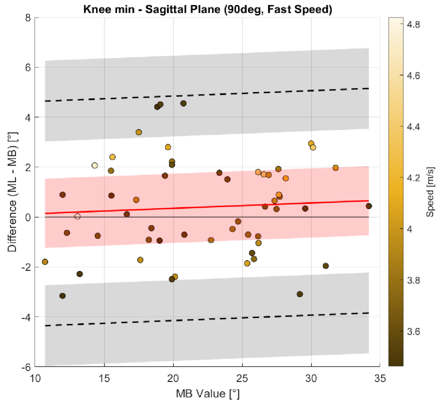

Supplementary Figure 721: Extended BA Plot for minimal knee angle - Sagittal Plane (90deg, Fast)

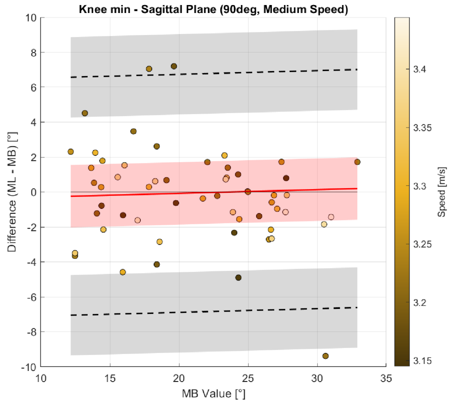

Supplementary Figure 722: Extended BA Plot for minimal knee angle - Sagittal Plane (90deg, Medium)

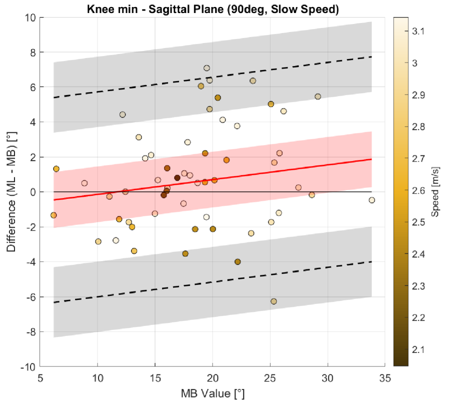

Supplementary Figure 723: Extended BA Plot for minimal knee angle - Sagittal Plane (90deg, Slow)

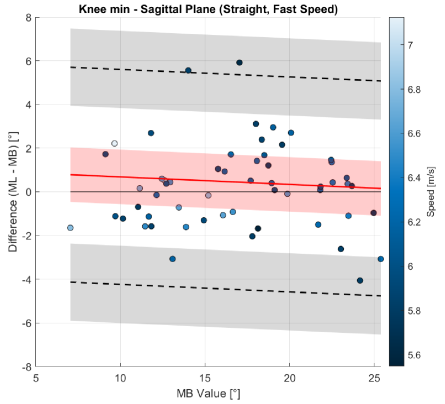

Supplementary Figure 724: Extended BA Plot for minimal knee angle - Sagittal Plane (Straight, Fast)

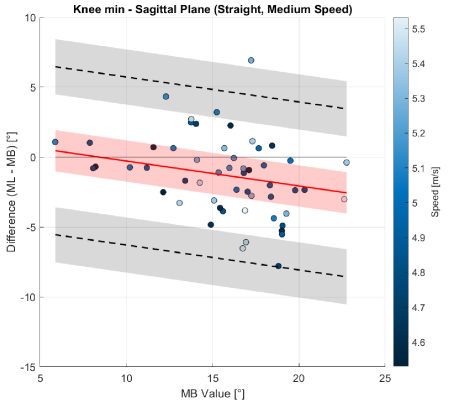

Supplementary Figure 725: Extended BA Plot for minimal knee angle - Sagittal Plane (Straight, Medium)

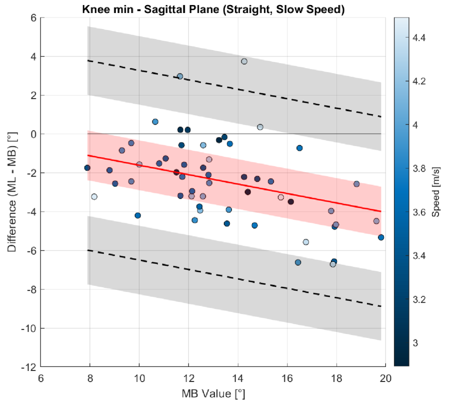

Supplementary Figure 726: Extended BA Plot for minimal knee angle - Sagittal Plane (Straight, Slow)

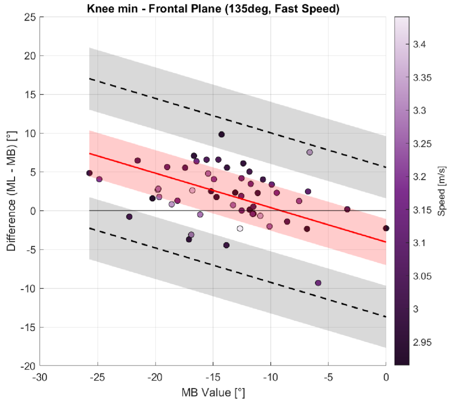

Supplementary Figure 727: Extended BA Plot for minimal knee angle - Frontal Plane (135deg, Fast)

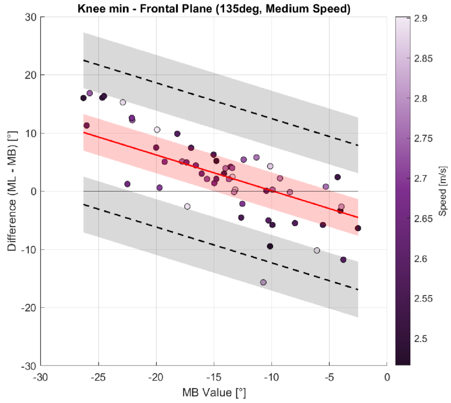

Supplementary Figure 728: Extended BA Plot for minimal knee angle - Frontal Plane (135deg, Medium)

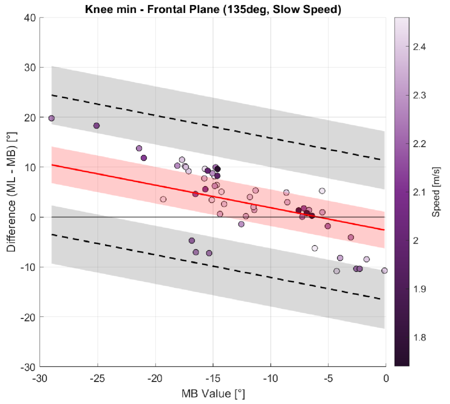

Supplementary Figure 729: Extended BA Plot for minimal knee angle - Frontal Plane (135deg, Slow)

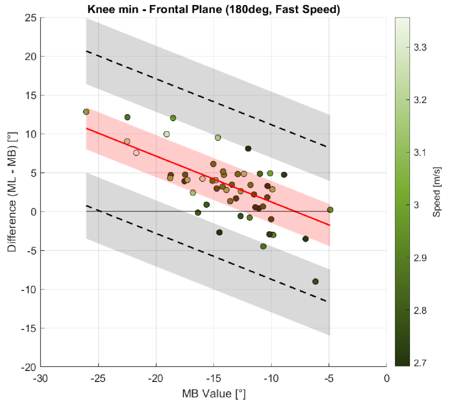

Supplementary Figure 730: Extended BA Plot for minimal knee angle - Frontal Plane (180deg, Fast)

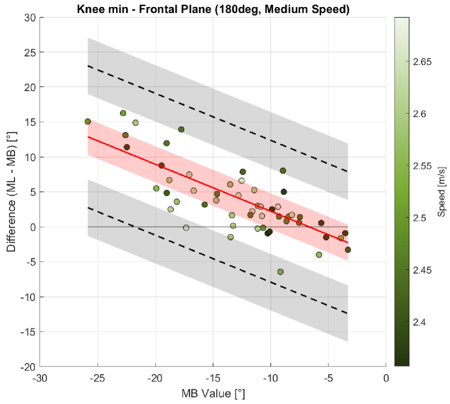

Supplementary Figure 731: Extended BA Plot for minimal knee angle - Frontal Plane (180deg, Medium)

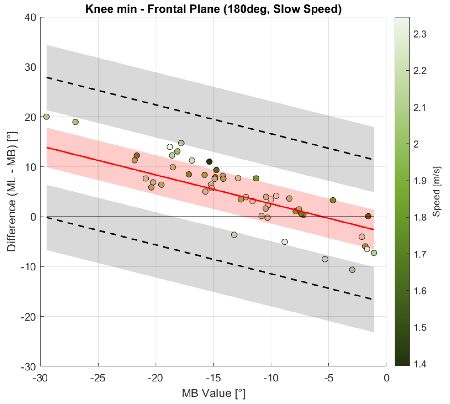

Supplementary Figure 732: Extended BA Plot for minimal knee angle - Frontal Plane (180deg, Slow)

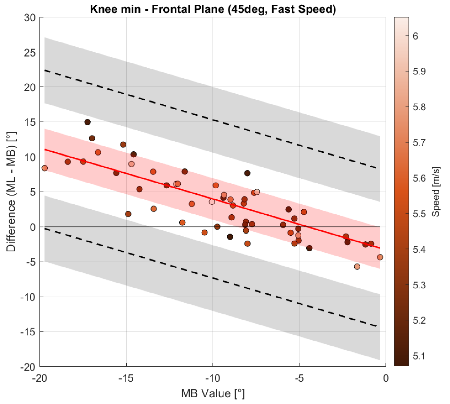

Supplementary Figure 733: Extended BA Plot for minimal knee angle - Frontal Plane (45deg, Fast)

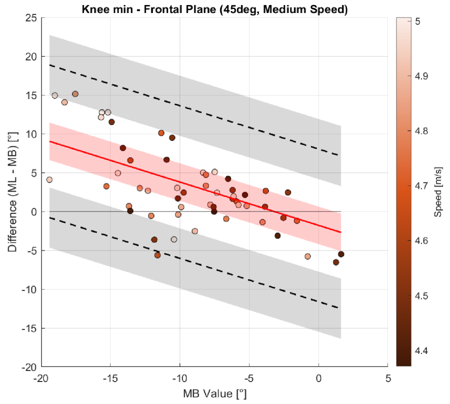

Supplementary Figure 734: Extended BA Plot for minimal knee angle - Frontal Plane (45deg, Medium)

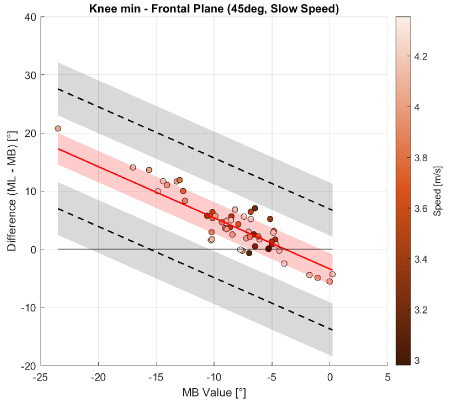

Supplementary Figure 735: Extended BA Plot for minimal knee angle - Frontal Plane (45deg, Slow)

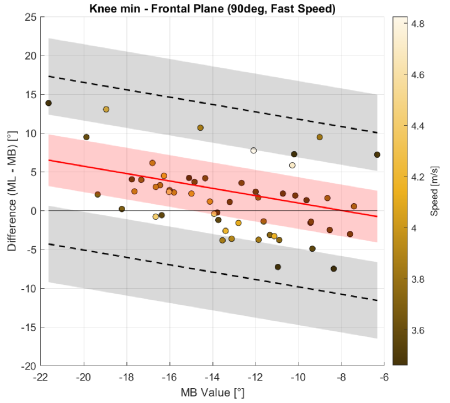

Supplementary Figure 736: Extended BA Plot for minimal knee angle - Frontal Plane (90deg, Fast)

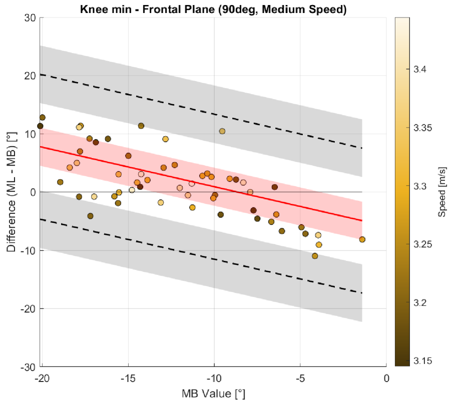

Supplementary Figure 737: Extended BA Plot for minimal knee angle - Frontal Plane (90deg, Medium)

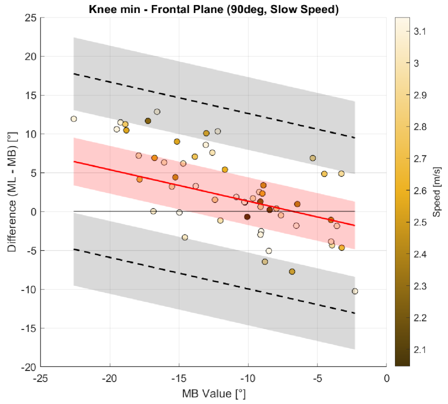

Supplementary Figure 738: Extended BA Plot for minimal knee angle - Frontal Plane (90deg, Slow)

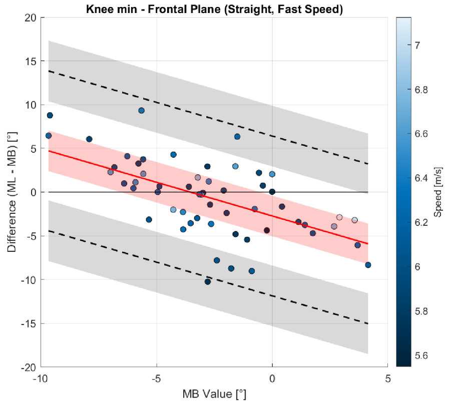

Supplementary Figure 739: Extended BA Plot for minimal knee angle - Frontal Plane (Straight, Fast)

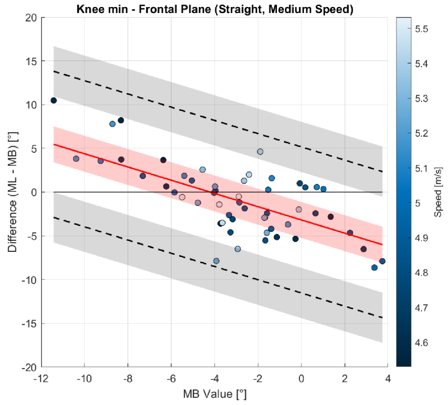

Supplementary Figure 740: Extended BA Plot for minimal knee angle - Frontal Plane (Straight, Medium)

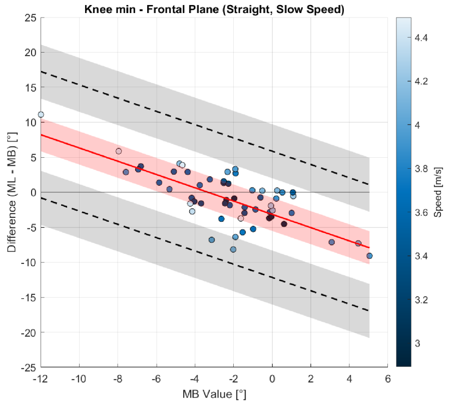

Supplementary Figure 741: Extended BA Plot for minimal knee angle - Frontal Plane (Straight, Slow)

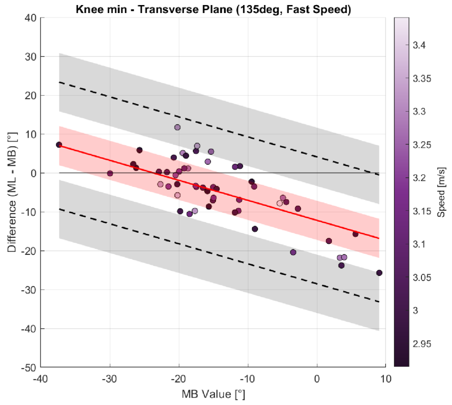

Supplementary Figure 742: Extended BA Plot for minimal knee angle - Transverse Plane (135deg, Fast)

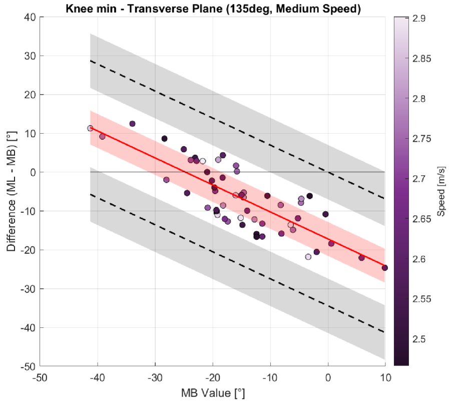

Supplementary Figure 743: Extended BA Plot for minimal knee angle - Transverse Plane (135deg, Medium)

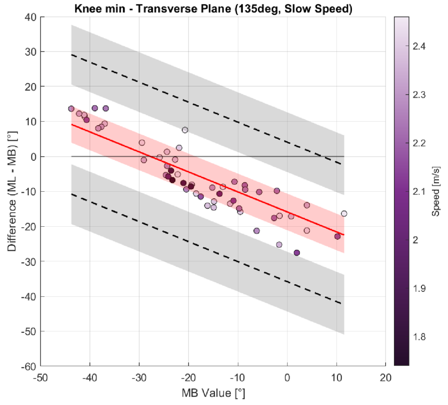

Supplementary Figure 744: Extended BA Plot for minimal knee angle - Transverse Plane (135deg, Slow)

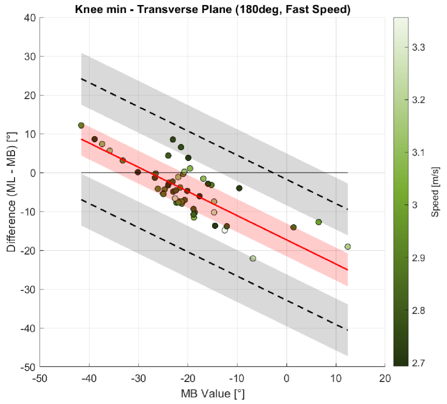

Supplementary Figure 745: Extended BA Plot for minimal knee angle - Transverse Plane (180deg, Fast)

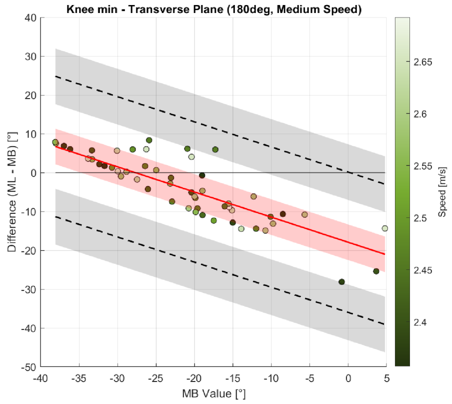

Supplementary Figure 746: Extended BA Plot for minimal knee angle - Transverse Plane (180deg, Medium)

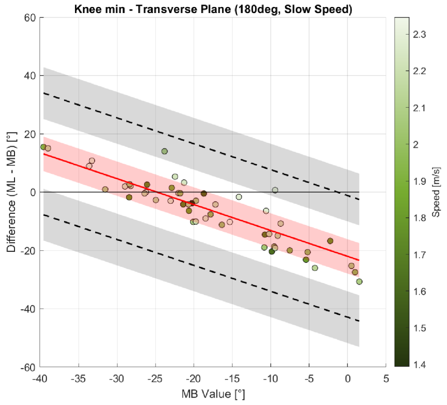

Supplementary Figure 747: Extended BA Plot for minimal knee angle - Transverse Plane (180deg, Slow)

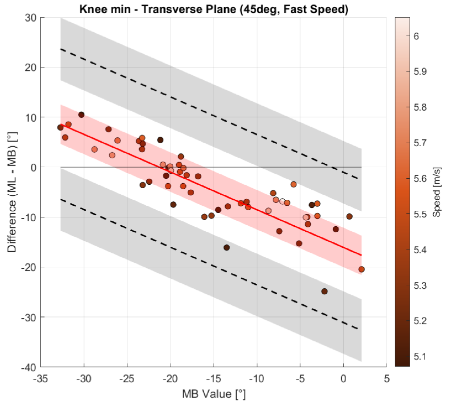

Supplementary Figure 748: Extended BA Plot for minimal knee angle - Transverse Plane (45deg, Fast)

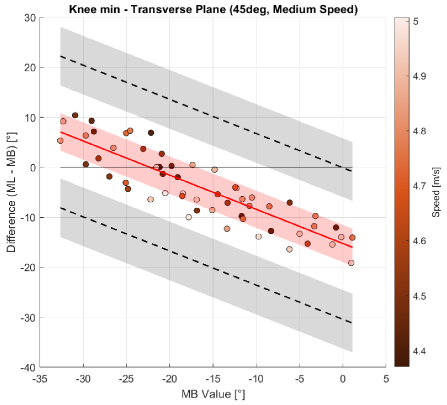

Supplementary Figure 749: Extended BA Plot for minimal knee angle - Transverse Plane (45deg, Medium)

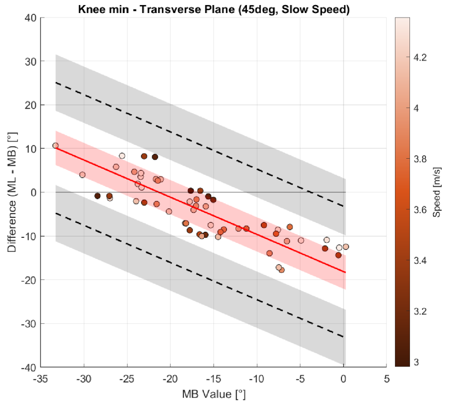

Supplementary Figure 750: Extended BA Plot for minimal knee angle - Transverse Plane (45deg, Slow)

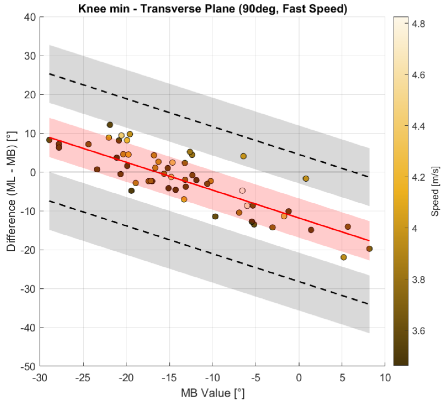

Supplementary Figure 751: Extended BA Plot for minimal knee angle - Transverse Plane (90deg, Fast)

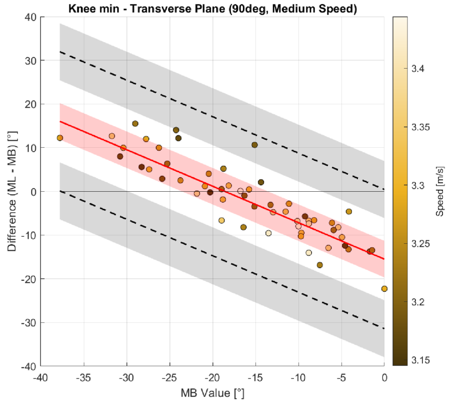

Supplementary Figure 752: Extended BA Plot for minimal knee angle - Transverse Plane (90deg, Medium)

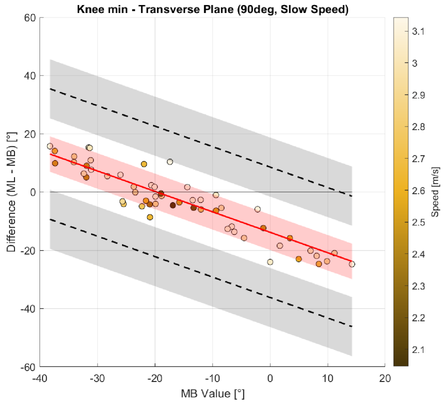

Supplementary Figure 753: Extended BA Plot for minimal knee angle - Transverse Plane (90deg, Slow)

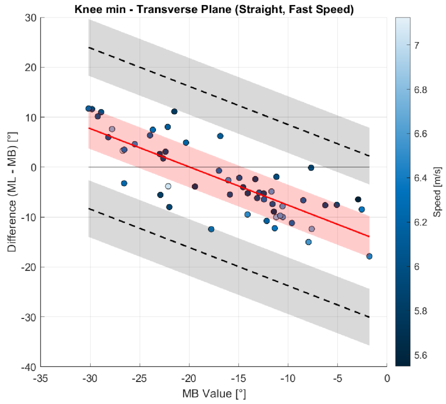

Supplementary Figure 754: Extended BA Plot for minimal knee angle - Transverse Plane (Straight, Fast)

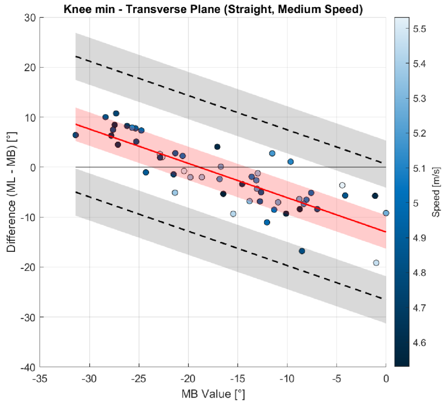

Supplementary Figure 755: Extended BA Plot for minimal knee angle - Transverse Plane (Straight, Medium)

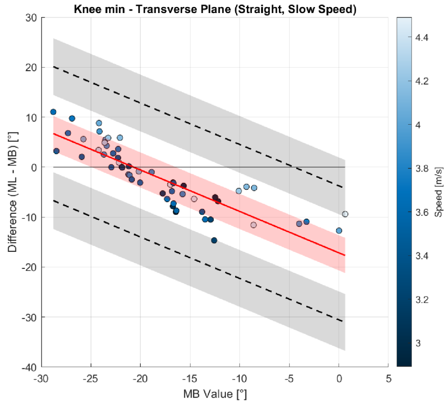

Supplementary Figure 756: Extended BA Plot for minimal knee angle - Transverse Plane (Straight, Slow)
